# Photocatalytic regioselective four-component radical relay carbonylation for α-aminoketones synthesis

**DOI:** 10.1039/d5sc04120a

**Published:** 2025-08-05

**Authors:** Mao-Lin Yang, Xiao-Feng Wu

**Affiliations:** a Leibniz-Institut für Katalyse e.V. Albert-Einstein-Str. 29a 18059 Rostock Germany xiao-feng.wu@catalysis.de; b Dalian National Laboratory for Clean Energy, Dalian Institute of Chemical Physics, Chinese Academy of Sciences 116023 Liaoning China

## Abstract

Regioselective transformation is among the long-standing challenges in organic synthesis, particularly involving gas trapping. We present here a novel photocatalytic strategy for the regioselective carbonylation reaction toward α-aminoketones. Experimental studies reveal that protonic acids dissociated from protonated amines facilitate the reactions of low-reactive aldehydes and enhance the electrophilicity of imines, thereby promoting the efficient coupling of less nucleophilic acyl radicals. This approach introduces a reliable framework for controlling regioselectivity in photocatalytic multi-radical-coupled CO trapping reactions, broadens the chemical space of α-aminoketones, and advances carbonylation reactions for sustainable development.

## Abstract 2

A novel photocatalytic strategy for the regioselective carbonylation reaction toward α-aminoketones has been developed.

## Graphical abstract

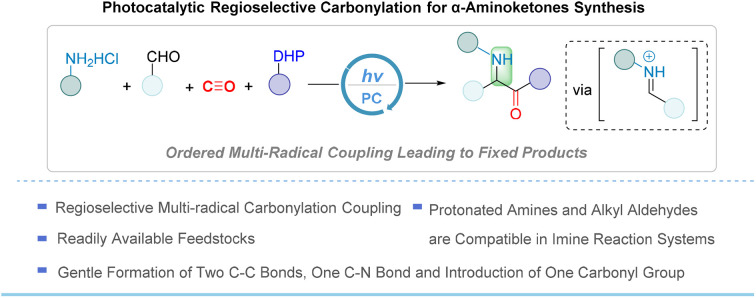

## Introduction

**Fig. 1:**
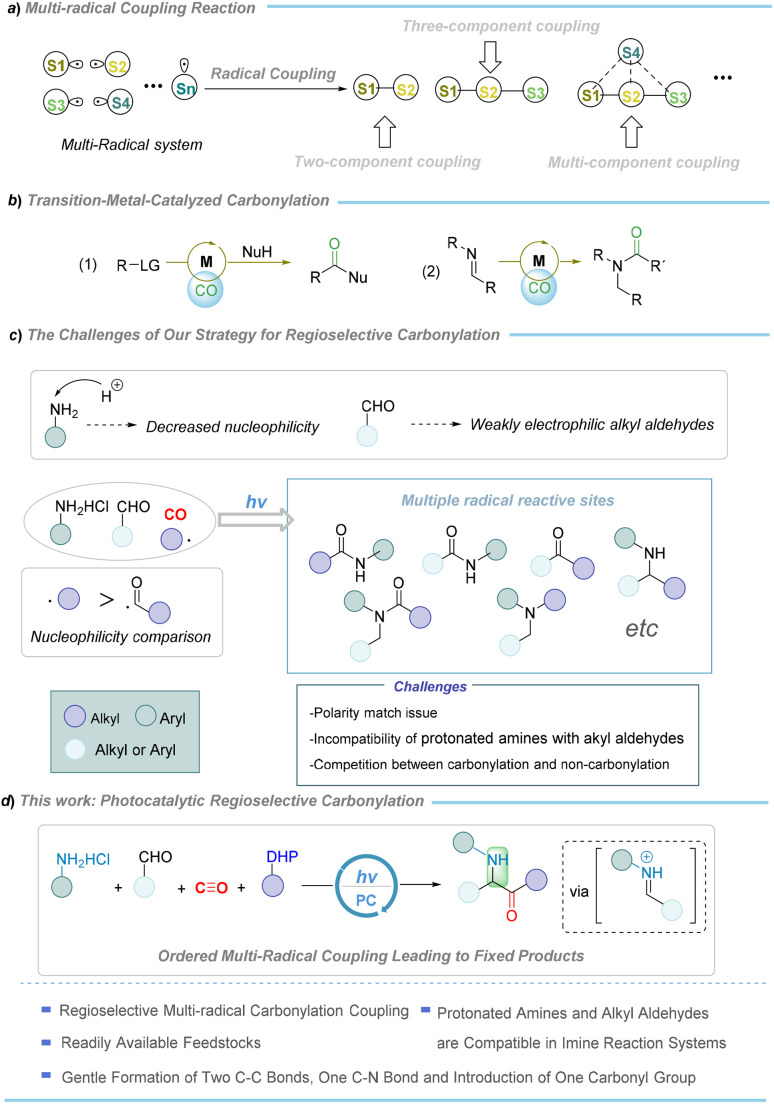
Background of regioselective multi-radical reaction and our design.

Designing highly regioselective and mild strategies to facilitate the orderly coupling of radicals with multiple reactive sites, thereby enabling the precise construction of target organic molecules, is highly desired in organic synthesis and sustainable chemistry, but still presents a significant challenge.^1,2^ As shown in Fig. 1a, multi-radical coupling reactions offer immense potential for constructing complex molecular frameworks. However, the presence of multiple possible coupling pathways between radicals, as well as the inherent susceptibility of radicals to quenching, creates substantial barriers to achieving precise radical coupling in multi-radical systems. Achieving high regioselectivity in multi-radical coupling reactions, innovative strategies are required, along with an in-depth understanding of the factors governing radical reactivity, stability, lifetime, and potential competitive side reactions. Furthermore, this challenge is exacerbated when highly reactive side reactions dominate, and when the required radical intermediates are unstable, especially when precise orchestration of the radical coupling process is essential. Tackling these challenges offers the potential for transformative advancements in drug discovery, materials science, and beyond, laying the foundation for novel advancements in synthetic chemistry. Among the possibilities, ionic pairing interacted coupling reactions, which rely on cationic and anionic reaction mechanisms, offering the potential for efficient regioselectivity control through variations in catalysts, solvents, or ligands, enabling the precise synthesis of the target molecules.^3–5^

Carbon monoxide (CO) has been recognized as an abundant, cost-effective, and versatile C1 feedstock.^6–11^ The advancement of its innovative transformations has been successfully achieved, and the significance of these developments was highlighted by the 2021 Nobel Prize in Chemistry.^12^ Since the middle of 20th century, transition metal-catalyzed carbonylation reactions have been enabled by a series of landmark developments, including the Mond–Langer process,^13^ the Gattermann–Koch reaction,^14^ Roelen's oxo process,^15^ the Monsanto/Cativa process,^16^ and Heck's palladium-catalyzed methodology^17^ (Fig. 1b(1)). While transition metal-catalyzed carbonylation has become a fundamental method for producing carbonylated compounds,^18–25^ challenges persist in achieving green and sustainable chemistry, including dependence on precious metal catalysts, the need for high-energy radiation, the requirement of exogenous additives or ligands, *etc.*

In recent decades, photocatalytic single-electron transfer (SET)-mediated radical coupling reaction has emerged as a powerful strategy in organic synthesis, offering an environmentally benign approach for the construction of complex molecular frameworks.^26–32^ As a promising alternative to conventional two-electron transition metal catalysis, photocatalytic SET-mediated carbonylation of CO has garnered significant attention, owing to its alignment with sustainable chemistry principles. However, this strategy faces significant challenges, particularly in controlling regioselectivity in multi-radical systems, where competition between carbonylative and non-carbonylative pathways poses a major hurdle. Therefore, the development of regioselective carbonylative radical coupling methods is crucial.

Among the various unsaturated chemical bonds, imines serve as pivotal intermediates in the synthesis of bioactive molecules,^33–36^ with widespread application in both academia and industry, which was also highlighted by the Nobel Prize contributions of Knowles and Noyori.^37^ However, the instability of imines presents substantial challenges for their synthetic applications, particularly when protonated amines or alkyl aldehydes are employed as reactants. Protonation introduces a positive charge on the nitrogen atom of the amine, which reduces its nucleophilicity and consequently lowers its ability to effectively nucleophilic attack the carbonyl carbon of the aldehyde. Moreover, the condensation between protonated amines and aldehydes involves a proton transfer process, which is controlled by the equilibrium between proton donors, potentially further slowing the rate of imine formation. In contrast to aryl aldehydes, alkyl aldehydes lack a conjugated system, rendering their carbonyl carbons less electrophilic and thereby making nucleophilic attack by amines more challenging. Therefore, developing mild and efficient synthetic strategies to facilitate the imine formation from protonated amines or alkyl aldehydes and enable their subsequent transformations is of considerable importance.

α-Aminoketones as valuable synthons and key constituents of pharmaceuticals and bioactive molecules,^38^ make the carbonylation of imines to their synthesis highly valuable. However, research in this area is still rare, likely the instability of imines and poor compatibility with carbon monoxide.^39–44^ In general, the reaction site is usually at the nitrogen atom in the conventional metal-catalyzed carbonylation with imines (Fig. 1b(2)). This is due to the nitrogen's lone pair favors σ-coordination with metals,^41–44^ thus hindering the formation of α-aminoketones. In contrast, mild radical coupling carbonylation strategies hold great potential for overcoming these challenges. Nevertheless, imine carbonylation *via* radical coupling remains largely unexplored, primarily due to the uncontrolled radical couplings in multi-component systems. Thus, developing a mild, regioselective radical-mediated imine carbonylation strategy, especially for advanced multi-component variants, is highly desirable. Such transformation represents a highly appealing approach, offering several distinct advantages: (1) introducing a novel and highly regioselective approach to multi-radical coupling carbonylation reactions; (2) broadening the synthetic utility of imines and their applications by enabling the efficient transformation of protonated amines and alkyl aldehydes, which typically show low reactivity; (3) comparing to using imines as starting materials, *in situ* generation of imine intermediates for subsequent reactions significantly mitigates the detrimental impact of imine instability on the reaction progress; (4) this system represents the first photocatalysis strategy for the carbonylative transformation of protonated amines and aldehydes into aminoketones, marking a significant milestone in sustainable carbonylation processes. However, the challenges for such transformation are obvious (Fig. 1c). First, the protonation of amines reduces their nucleophilicity, hindering the efficiency of imine formation with aldehydes. Second, alkyl aldehydes, with their weak nucleophilicity and steric hindrance, are less likely to form stable imines with amines. Third, the alkyl radical used to capture CO is more nucleophilic than the acyl radical, leading to a main competitive non-carbonylative coupling reaction. Fourth, the instability of imine and favored decarbonylation of acyl radical require careful optimization of reaction conditions. Additionally, incompatibilities between the imine formation process and carbonylation may disrupt the SET relay, thereby impeding the overall catalytic.

Based on the above considerations and our ongoing interests in the exploration of sustainable carbonylative transformations,^45–51^ we designed a new photocatalytic carbonylation catalytic system. This system leverages the high regioselective photocatalytic carbonylation of protonated amines and aldehydes to α-aminoketones. In this process, protonated amines slow down the reaction, allowing alkyl radicals to capture CO and forming acyl radicals which could minimize non-carbonylative coupling of alkyl radicals. On the other hand, the proton increases imine electrophilicity and enhances the reactivity of alkyl aldehydes,^52^ aiding the coupling with acyl radicals. This photocatalytic process operates in tandem, with each step complementing the other to enable an efficient CO-inclusive four-component radical coupling reaction, yielding a range of substituted α-aminoketones in good yields. The success of this transformation provides valuable insights into the application of highly regioselective and mild photocatalytic strategies, facilitating the orderly coupling of radicals with multiple reactive sites in complex reaction systems, and fostering the sustainable development of carbonylation processes.

## Results and discussion

To realize the above design of photocatalytic regioselective monoxide-inclusive four-component carbonylation of protonated amines and aldehydes to α-aminoketones (for more details, see the ESI), we initially employed benzaldehyde 1a, hydrochloride of aniline 2a, and Hantzsch ester 3a as model substrates under 400–500 nm irradiation, wherein we explored the carbonylation under 40 bar of CO, as shown in Table 1. At the beginning, Ir(dtbbpy)(ppy)_2_PF_6_ (PC-2, 1.5 mol%) was identified as a more effective photocatalyst than the alternatives (Table 1, entries 4–8), the optimal reaction temperature was found to be 30 °C (Table 1, entries 2 and 3), and the optimal reaction solvent was CHCl_3_ (Table 1, entries 4, 9–12). Of course, to achieve efficient CO conversion in this multi-component carbonylation system, we investigated the effects of substrate ratio and reaction time. The optimal substrate ratio was found to be 1 : 1 : 1.2 (1a/2a/3a), with the ideal reaction time being 24 h (Table 1, entries 13–15). Additional control experiments underscored the essential roles of light and the photocatalyst, in achieving a successful transformation (Table 1, entries 16). Finally, the optimal reaction conditions are shown in Table 1, entries 1 (1a/2a/3a = 1 : 1 : 1.2, CHCl_3_ (0.1 M), CO (40 bar), PC-2 (1.5 mol%), under 400–500 nm irradiation, at 30 °C for 24 h). It is also worth to mention that none carbon monoxide insertion (non-carbonylation) product can be detected as the main by-product during the optimization process in case of low yield.

**Table 1:** Optimization of the reaction conditions^a^

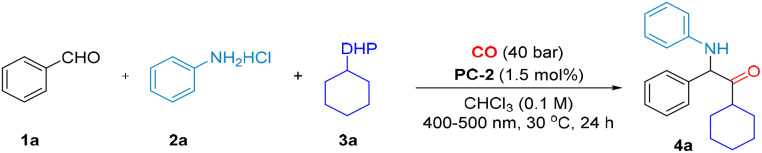		
Entry	Deviation from standard conditions	Yield (%)
1^a,d^	None	89 (82)
2^a,d^	30 °C for 15 h	60
3^a,d^	50 °C instead of 30 °C, for 15 h	41
4^b,e^	PC-1 instead of PC-2, for 15 h	35
5^b^	15 h instead of 24 h	30^c^, 49^d^, 45^e^
6^b,e^	PC-3 instead of PC-2, for 15 h	25
7^b,e^	PC-4 instead of PC-2, for 15 h	—
8^b,e^	PC-5 instead of PC-2, for 15 h	30
9^b,d^	THF instead of CHCl_3_, for 15 h	32
10^b,d^	DCE instead of CHCl_3_, for 15 h	43
11^b,d^	DCM instead of CHCl_3_, for 15 h	40
12^b,d^	MeCN instead of CHCl_3_, for 15 h	Trace
13^a,d^	15 h instead of 24 h	55
14 ^a,d^	12 h instead of 24 h	40
15 ^a,d^	20 h instead of 24 h	72
16 ^a,d^	Without PC or light	—
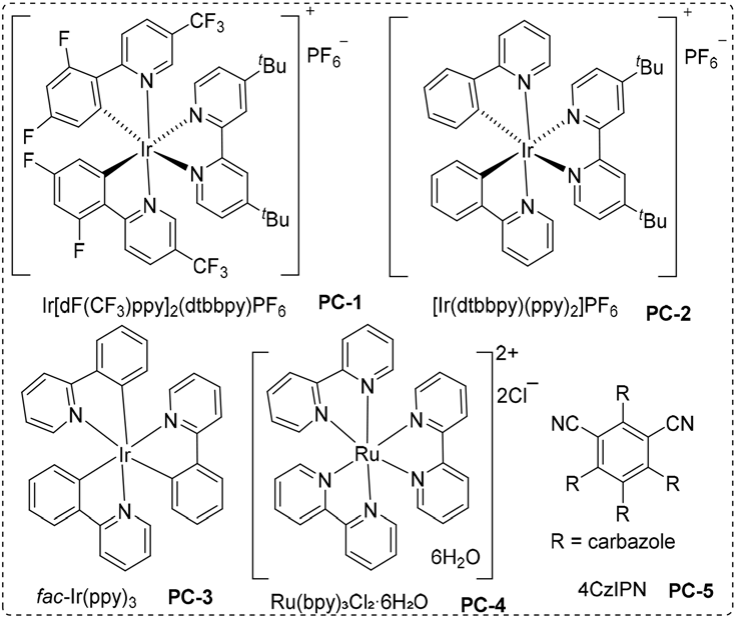		

a The reaction was conducted using 1a (0.1 mmol), 2a (0.1 mmol), 3a (0.12 mmol^a^, 0.15 mmol^b^), CHCl_3_ (1.0 mL), CO (40 bar) irradiation with blue light at 30 °C for 24 h. PC (1 mol%^c^, 1.5 mol%^d^, 2 mol%^e^). Determined by GC with hexadecane as internal standard. Isolated yield is shown in parentheses.

**Scheme 1:**
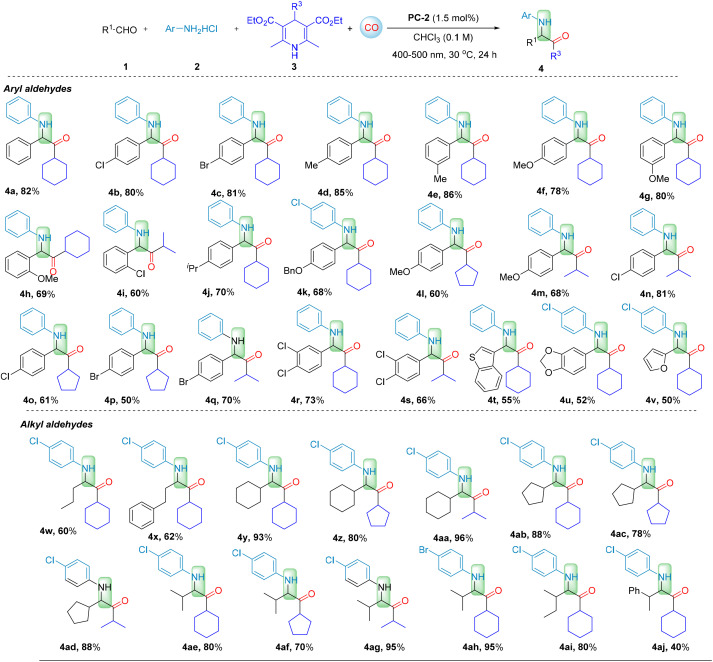
Reaction conditions: 1 (0.2 mmol), 2 (0.2 mmol), 3 (0.24 mmol), CHCl_3_ (2.0 mL), CO (40 bar), PC-2 (1.5 mol%), under 400–500 nm irradiation, at 30 °C for 24 h. Isolated yield.

**Scheme 2:**
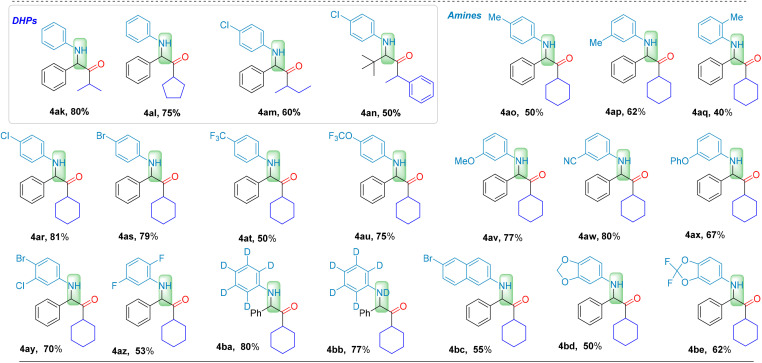
Reaction conditions: 1 (0.2 mmol), 2 (0.2 mmol), 3 (0.24 mmol), CHCl_3_ (2.0 mL), CO (40 bar), PC-2 (1.5 mol%), under 400–500 nm irradiation, at 30 °C for 24 h. Isolated yield.

**Scheme 3:**
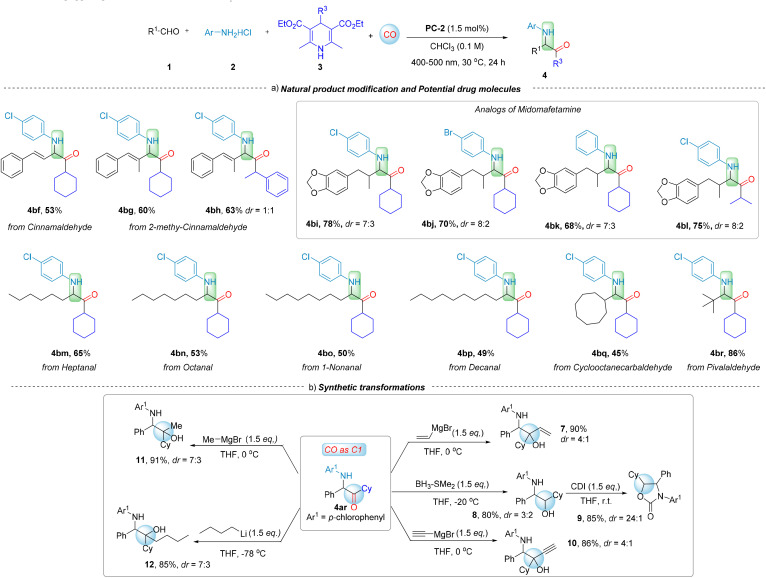
Reaction conditions: 1 (0.2 mmol), 2 (0.2 mmol), 3 (0.24 mmol), CHCl_3_ (2.0 mL), CO (40 bar), PC-2 (1.5 mol%), under 400–500 nm irradiation, at 30 °C for 24 h. Isolated yield.

Under the optimized conditions, we explored the applicability of this regioselective four-component carbonylation for the synthesis of various α-aminoketones (Schemes 1–3). A series of substituted arylaldehydes were tested at the first stage, and the carbonylated products α-aminoketones 4a–4v, were obtained in moderate to good yields (50–86%). Even *ortho*-substituted benzaldehydes with steric hindrance, resulted in carbonylation products in moderate yields (4h with 69%, and 4i with 60%). Benzo[*b*]thiophene-3-carbaldehyde gave the desired product 4t in 52%. Additionally, benzo[*d*][1,3]dioxole-5-carbaldehyde and furan-2-carbaldehyde afforded products 4u and 4v in 52% and 50% yields, respectively. Remarkably, alkyl aldehydes also participated in the reaction with high efficiency. Products such as 4w–4aj were obtained in moderate to good yields (40–96%), representing a significant breakthrough in challenging the traditional view that alkyl aldehydes are less favored in imine formation and their subsequent applications. Among them, secondary aldehydes exhibited the best reactivity (4y–4ai, 70–96% yields), with even bulky-substituted 2-phenylpropanal giving product 4aj in 40% yield. Primary aldehydes showed decreased reactivity, but still able to give the desired products 4w–4x in 60–62% yields. Surprisingly, even tertiary aldehyde with bulky substituent demonstrated excellent reactivity (4br, 86%). Interestingly, experimental results indicate that alkyl aldehydes exhibit excellent reactivity in our system. This observation contrasts with the conventional notion that alkyl aldehydes are less reactive due to the lack of conjugative stabilization, representing a notable advancement in the application of alkyl aldehydes in imine-based synthetic systems.

Furthermore, the substrate scope of arylamines hydrochloride and Hantzsch esters (DHP) were also examined. When investigating the arylamines functionalized with various substituents on the benzene ring, the influence of the electronic properties of the aromatic ring became apparent. Substituents with electron-donating and electron-withdrawing characteristics were found to significantly impact the reaction efficiency. Among these, electron-donating substituents decrease the yield. For instance, employing a methyl-substituted aniline hydrochloride as the substrate resulted in the formation of aminoketone products 4ao–4aq in 40–50% yields, especially with anilines with *para*-substitution or *ortho*-substitution. In contrast, the reactions involving anilines with electron-withdrawing *para*-substituents on the benzene ring resulted in higher yields of 4ar–4au (50–81%).

Fluorine-substituted aryl amines underwent carbonylation with moderate yields (4at with 50%; 4au with 75%). The moderate reactivity of *meta*-substituted aryl hydrochlorides is attributed to the minimal impact of *meta*-substituents on the electron density of the reaction substrate, as well as the steric hindrance during the reaction (4av–4ax, 67–80%). Additionally, double-substituted aryl amines can also be used to deliver the corresponding products (4ay with 70%; 4az with 53%). 6-Bromonaphthalen-2-amine hydrochloride yield the desired 4bc in 55% yield and benzo[*d*][1,3]dioxol-5-amine hydrochloride gave 4bd in 50% yield. Fluorine-substituted 2,2-difluorobenzo[*d*][1,3]dioxol-5-amine hydrochloride reacted smoothly to afford the corresponding 4be in 62% yield. Different substituted Hantzsch esters were also successfully employed for this conversion (4ak–4an, 50–80%). Notably, usually benzylic radicals are considered stable which making CO insertion difficult and the resulting acyl radicals prone to decarbonylation. However, in the current system, the formed acyl radical can be captured by the iminium salt promptly which then lead the desired carbonylation formed as the main product. These results underscore the broad substrate scope and excellent performance of this new regioselective carbonylation system for the synthesis of α-aminoketones.

To showcase the functional group tolerance of this new synthetic procedure, we set out to explore the generality of this protocol for the late-stage modification of different complex molecules (Scheme 3, eqn (a)). Aldehydes derived from natural products such as cinnamaldehyde (3bf), 2-methy-cinnamaldehyde (3bg and 3bh), heptanal (3bm), octanal (3bn), 1-nonanal (3bo) and decanal (3bp), as well as macrocyclic alkyl aldehydes such as cyclooctanaldehyde (3bq), can be used in this transformation smoothly, providing advanced α-aminoketones in moderate to good yields. Additionally, pivalaldehyde (3br) as an example of bulky alkyl aldehyde can participate in the reaction without any problem despite its steric hindrance, which represents a breakthrough in the synthesis of imines from large steric hindrance aldehydes under mild conditions. Compounds analogous to Midomafetamine can also be synthesized through Elional (3bi–3bl). These results highlight the potential of this method for late-stage modifications of natural compounds and drug molecules.

Next, the synthetic transformations of this produced carbonylation product were performed (Scheme 3, eqn (b)). The condensation of 4ar, facilitated by magnesium methyl bromide in tetrahydrofuran at 0 °C, resulted in the formation of 1-((4-chlorophenyl)amino)-2-cyclohexyl-1-phenylpropan-2-ol 11. Reactions of 4ar with *n*-butyllithium in tetrahydrofuran at −78 °C, yielded 1-((4-chlorophenyl)amino)-2-cyclohexyl-1-phenylhexan-2-ol 12. Reactions of 4ar with vinyl magnesium bromide or acetylenylmagnesium bromide in tetrahydrofuran at 0 °C led to the formation of 1-((4-chlorophenyl)amino)-2-cyclohexyl-1-phenylbut-3-en-2-ol 7 or 1-((4-chlorophenyl)amino)-2-cyclohexyl-1-phenylbut-3-yn-2-ol 10. Treatment of 4ar with borane dimethylsulfide in tetrahydrofuran at −20 °C afforded 2-((4-chlorophenyl)amino)-1-cyclohexyl-2-phenylethan-1-ol 8, which can be used to synthesize 3-(4-chlorophenyl)-5-cyclohexyl-4-phenyloxazolidin-2-one 9. These results not only validate the potential applications of aminoketone-based skeleton compounds but also offer novel insights into the efficient conversion of CO as versatile C1 feedstock under mild conditions, further underscoring the efficacy and value of this photocatalytic regioselective carbonylation procedure.

**Scheme 4:**
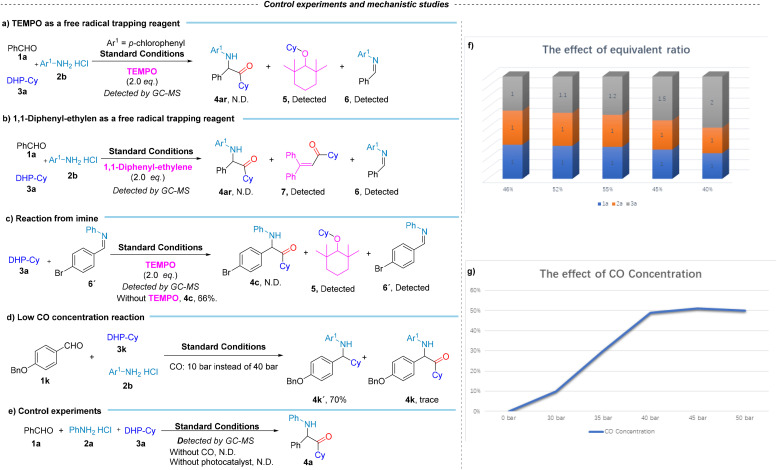
Radical capture experiments, and exploration of influencing factors of catalytic system. 1a, 2a and 3a: (f) CHCl_3_ (0.1 M), CO (40 bar), PC-2 (1.5 mol%), under 400–500 nm irradiation, at 30 °C for 15 h. (g) 1a/2a/3a = 1/1/1.5, CHCl_3_ (0.1 M), PC-2 (1.5 mol%), under 400–500 nm irradiation, at 30 °C for 15 h. Isolated yield.

**Scheme 5:**
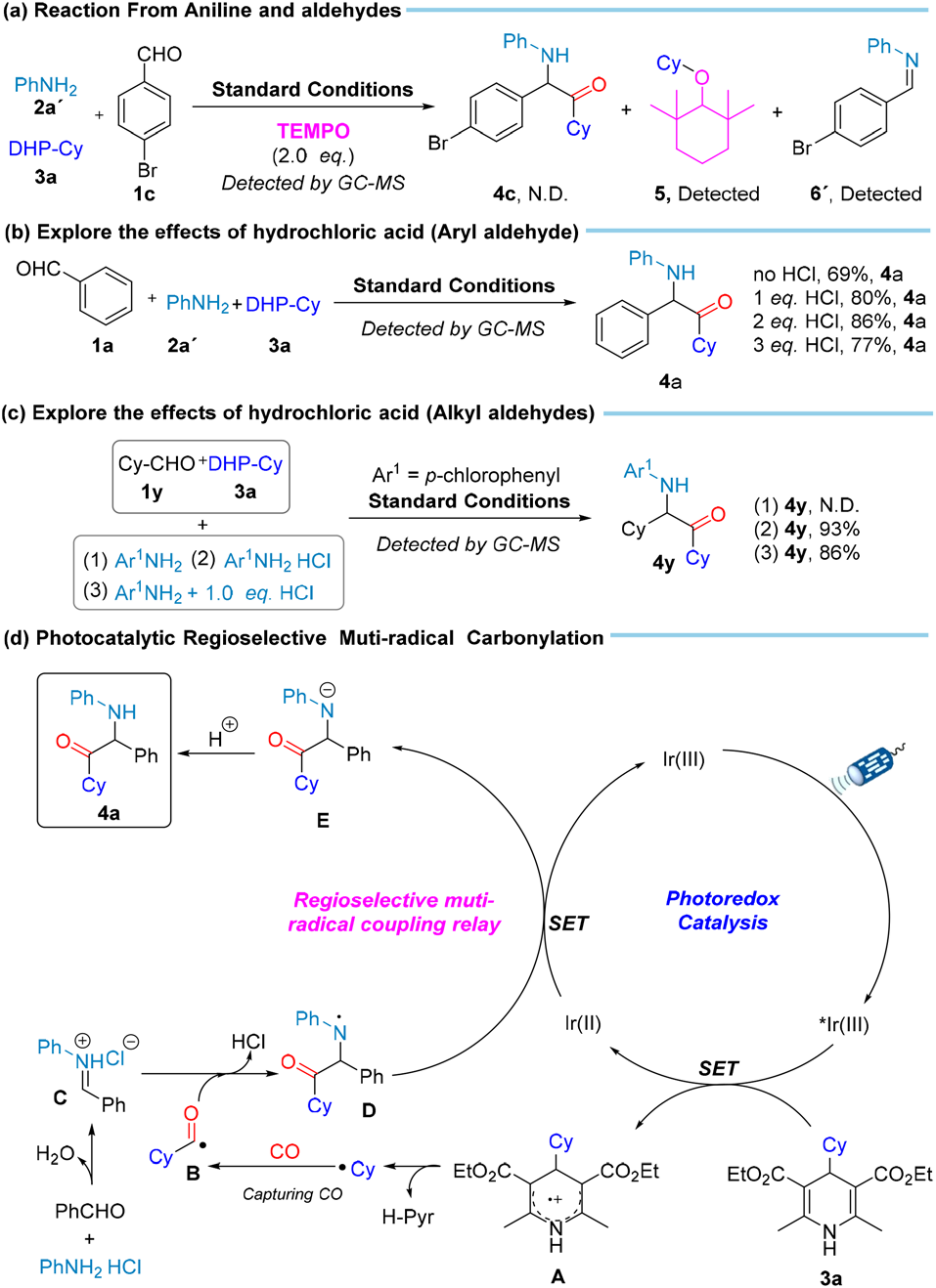
Plausible reaction mechanism for the photocatalytic regioselective carbonylation.

To gain more insight into the mechanism of this reaction, several mechanistic experiments were conducted (Schemes 4 and 5). Firstly, we tested 2,2,6,6-tetramethyl-1-piperidinoxy (TEMPO) as free radical scavenger in our system. Under standard reaction conditions, no product 4a was obtained, and product cyclohexyl-TEMPO 5, as the main product, was detected by GC-MS. Imine 6 can be detected, which validates the presence of the cyclohexyl radical and imine. Then, we tested with 1,1-diphenylethylene and analyzed the reaction products *via* GC-MS. No formation of product 4ar was observed, contrary to expectations. However, the detection of 1-cyclohexyl-3,3-diphenylprop-2-en-1-one 7 and imine 6 was consistent with the presence of cyclohexyl radicals and imines in the reaction system. To better understand the crucial role of imines, we initiated the reaction with imine 6′. Upon adding TEMPO to the reaction system, GC analysis revealed no formation of product 4c, and imine 6′ was detected. In contrast, when TEMPO was omitted, product 4c was successfully obtained. This result proves that imine is an essential intermediate produced during the reaction. Control experiments to verify the necessity of reaction conditions were also performed (Scheme 4, eqn (e)). When the reactions were performed in the absence of light, CO, or photocatalyst, the desired product was not found. These results suggested that light, CO, and photocatalyst are necessary to induce the production of aminoketone compounds. Considering the multi-radical nature of the gas trapping reaction, several side coupling reactions may occur which potentially hindering the efficient formation of the target compounds. To address this, we investigated the effects of the feedstock ratio and CO concentration on the reaction, excluding interference from non-carbonylation by-products. Our findings indicate that with only 10 bar of CO, the main product is the non-carbonylated compound 4k′ (Scheme 4, eqn (d)). In further optimization, we identified that the optimal CO pressure for the reaction is 40 bar (Scheme 4, eqn (f)). Additionally, when exploring the feedstock ratio, the optimal ratio of 1a : 2a : 3a was found to be 1 : 1 : 1.2 (Scheme 4, eqn (g)). These results support our initial hypothesis that the competing non-carbonylation reaction is a primary challenge, which can be mitigated by adjusting the CO concentration and the feedstock ratio. Finally, to clarify the effect of amine hydrochloride on the reaction system, we also carried out a series of experimental investigations (Scheme 5, eqn (a)–(c)). Based on the results, we found that the protonic acid is a crucial factor in promoting high-precision and effective multicomponent radical coupling. This not only provides insight into the reaction mechanism but also validates the feasibility of using the designed hydrochloride salt as a reactant to promote the coupling of acyl radicals for regioselective carbonylation.

Based on all the experimental results and literature precedents,^53–55^ we propose the following possible catalytic cycle (Scheme 5, eqn (d)). Initially, the aldehydes and hydrochloride salt of amines undergo dehydration and condensation to yield imine hydrochloride intermediate C. Simultaneously, under light irradiation, the photosensitizer is excited, leading to the oxidation of Hantzsch esters (3a) by the photoactivated species, generating intermediate A. This process results in the formation of cyclohexyl radical and the release of diethyl 2,6-dimethylpyridine-3,5-dicarboxylate (H-Pyr) which can then neutralize the released HCl. The cyclohexyl radical subsequently captures CO to form the acyl radical B, which undergoes radical addition to intermediate C, generating intermediate D and releasing a hydrogen chloride. Subsequently, with the assistance of photocatalytic single-electron transfer, the intermediate E is formed. Finally, the protonation of intermediate E with H^+^ furnishes the desired carbonylation product aminoketone 4a.

## Conclusions

In summary, we have developed a novel photocatalytic CO-inclusive four-component multi-radical carbonylation coupling reaction with high regioselectivity, enabling the synthesis of a range of substituted α-aminoketones in moderate to good yields. From an application perspective, this protocol can be used for late-stage modifications of complex molecules and other valuable synthetic transformations. Experimental results and mechanistic studies indicate that protonic acid is crucial for achieving efficient regioselective carbonylation, providing a valuable strategy for controlling regioselectivity in multi-radical coupling reactions of imines. The success of this transformation provides valuable insights into the application of highly regioselective and mild photocatalytic strategies, facilitating the orderly coupling of carbonylation radicals with multiple reactive sites in complex reaction systems, and fostering the sustainable development of CO conversion processes.

## Author contributions

X.-F. W. conceived and directed the project. M.-L. Y. performed all the experiments, prepared the manuscript and the SI.

## Conflicts of interest

There are no conflicts to declare.

## Supplementary Material

SC-OLF-D5SC04120A-s001

## Data Availability

The data supporting this article have been included as part of the SI. General comments, general procedure, analytic data, and NMR spectra. See DOI: https://doi.org/10.1039/d5sc04120a.
