# Supplementary material for: Photocatalytic regioselective four-component radical relay carbonylation for α-aminoketones synthesis
Source: Chem Sci. 2025 Aug 5;16(34):15676–83. doi: 10.1039/d5sc04120a (PMC12322701; doi:10.1039/d5sc04120a)

Supplementary Information (SI) for Chemical Science.
This journal is © The Royal Society of Chemistry 2025

Supporting Information

Table of Contents

1. **General information** S2

2. **Preparation of substrates** S3

   2.1 Preparation of Hantzsch Ester 3 .... S3

3. **Complementary reaction optimization Data** S4

4. **Characterization and procedure of α-Aminoketones products 4 and synthetic transformations products** S8

   4.1 General diacylation procedure for the synthesis of α-Aminoketones 4 .... S8

   4.2 General procedure for the synthesis of 1-((4-chlorophenyl)amino)-2-cyclohexyl-1-phenylbut-3-en-2-ol 7 .... S8

   4.3 General procedure for the synthesis of 2-((4-chlorophenyl)amino)-1-cyclohexyl-2-phenylethan-1-ol 8 .... S8

   4.4 General procedure for the synthesis of 3-(4-chlorophenyl)-5-cyclohexyl-4-phenyloxazolidin-2-one 9 .... S9

   4.5 General procedure for the synthesis of 1-((4-chlorophenyl)amino)-2-cyclohexyl-1-phenylbut-3-yn-2-ol 10 .... S9

   4.6 General procedure for the synthesis of 1-((4-chlorophenyl)amino)-2-cyclohexyl-1-phenylpropan-2-ol 11 .... S9

   4.7 General procedure for the synthesis of 1-((4-chlorophenyl)amino)-2-cyclohexyl-1-phenylhexan-2-ol 12 .... S10

5. **Mechanistic investigation** S37

   5.1 Radical trapping experiment by 1,1-diphenylethylene S37

   5.2 Radical trapping experiment by 2,2,6,6-Tetramethylpiperidinyloxyl (TEMPO) S38

6. **References** S42

7. **NMR Spectra** S43

1. General information

Unless otherwise noted, materials were purchased from commercial suppliers and used without further purification. Flash column chromatography was performed using 200-300 mesh silica gel. ^{1}H NMR spectra were recorded on 300 or 400 MHz spectrophotometers. Chemical shifts are reported in ppm relative to tetramethylsilane (TMS, δ = 0 ppm) with the solvent resonance employed as the internal standard ($CDCl_3$: δ = 7.26 ppm). ^{13}C NMR was recorded at 75 MHz or 101 MHz: chemical shifts are reported in ppm from tetramethylsilane (TMS) with the solvent resonance as the internal standard ($CDCl_3$: δ = 77.00 ppm). Electron impact (EI) mass spectra were recorded on AMD 402 mass spectrometer (70 eV). High resolution mass spectra (HR-MS) were recorded on Agilent 6210. The data were given as mass units per charge (m/z). Gas chromatography analysis was performed on an Agilent HP-5890 instrument with an FID detector and HP-5 capillary column (polydimethylsiloxane with 5% phenyl groups, 30 m, 0.32 mm i.d., 0.25 μm film thickness) using argon as carrier gas.

The light source was positioned approximately 23 cm from the reaction vial, placed atop a manufactured autoclave (see **Figure 1**). For every reaction, the powerful light source, Portable Lumatec SUPERLITE S 04[1], was utilized with different set filters: UV-A (λ = 400 - 500 nm) at maximum intensity (100% power). **Figures 2** and **3** illustrate the relevant photophysical properties of the lamps.

Because of the high toxicity of carbon monoxide, all the reactions should be performed in an autoclave. The laboratory should be well-equipped with a CO detector and alarm system.

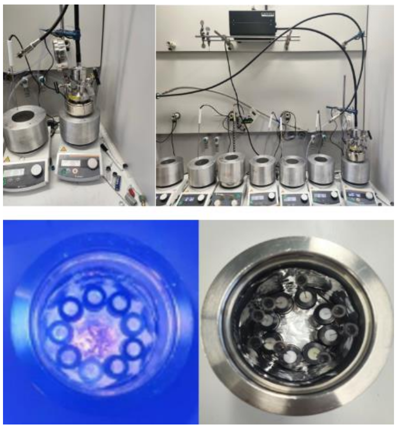

Figure 1. Apparatus for the Photoinduced Regioselective Multi-Radical Carbonylation

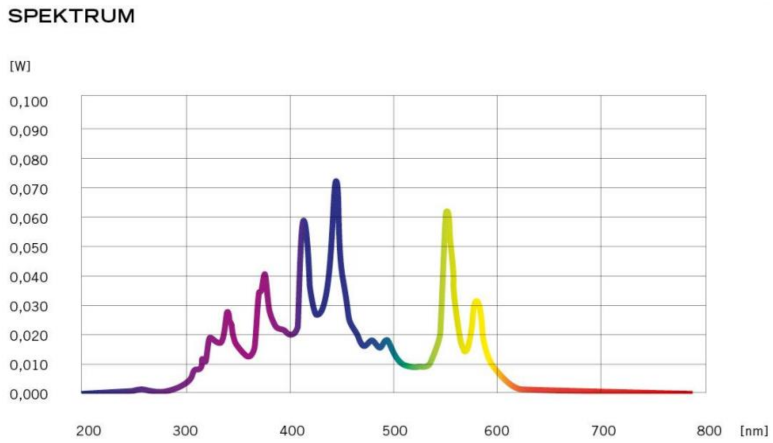

Figure 2. Emission Spectrums of the Portable Lumatec SUPERLITE S 04

OPTISCHE LEISTUNG

Spektrum		Leistung	Intensität
UVA	320–400 nm	2.100 mW	10.500 mW/cm²
UVA + Blau	320–500 nm	6.900 mW	34.500 mW/cm²
Blau	400–500 nm	4.800 mW	24.000 mW/cm²
Weiß	400–700 nm	9.700 mW	48.500 mW/cm²
Violett	415 nm	2.000 mW	10.000 mW/cm²
Blau 440	440 nm	2.300 mW	11.500 mW/cm²
Blau 460	460 nm	2.000 mW	10.000 mW/cm²
Türkis	490 nm	1.200 mW	6.000 mW/cm²
Grün	550 nm	1.400 mW	7.000 mW/cm²
Gelb	570 nm	1.800 mW	9.000 mW/cm²

Figure 3. Technical Specifications of the Portable Lumatec SUPERLITE S 04

2. Preparation of substrates

2.1 Preparation of Hantzsch Ester 3

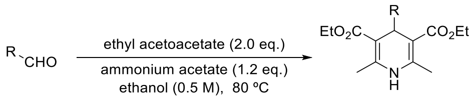

3

A reaction flask was charged with ethyl acetoacetate (1.3 g, 10.0 mmol, 2.0 equiv.), ammonium acetate (0.5 g, 1.2 equiv.), ethanol (10.0 mL). To the above solution, the aldehyde (5.0 mmol) was added slowly. After addition, the system was heated at 80 ºC with stirring. The reaction was monitored by TLC. When the reaction was completed, the solvent was evaporated, vacuum the crude product, half an hour later add petroleum ether ultrasound, to give the corresponding Hantzsch ester. GC Mass and Spectroscopic data in agreement with that reported previously.[2]

3. Complementary reaction optimization Data

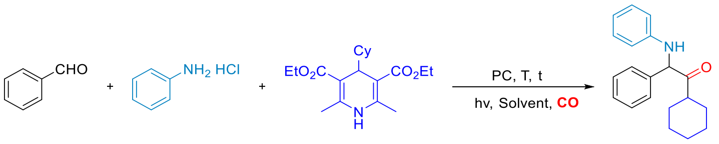

1a 2a 3a 4a

Table 1 Optimization of photosensitizer (PC)

Entry	PC	Yield (%)
1[c]	**PC-1**	35
2[c]	**PC-2**	45
3[c]	**PC-3**	25
4[c]	**PC-4**	-
5[c]	**PC-5**	30
6[c]	**PC-6**	-
7[a]	**PC-2**	30
8[b]	**PC-2**	49
9	-	-

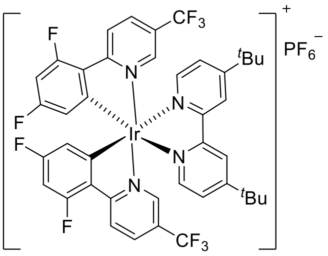

$Ir[dF(CF_3)ppy]_2(dtbbpy)PF_6$

PC-1

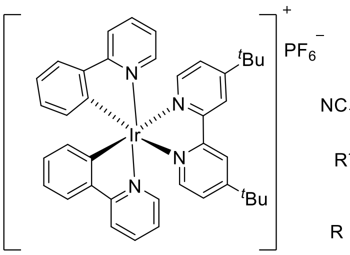

$[Ir(dtbbpy)(ppy)_2]PF_6$

PC-2

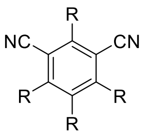

R = carbazole

4CzIPN

PC-5

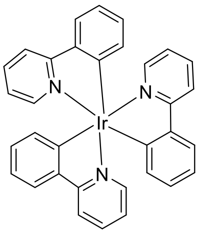

fac-$Ir(ppy)_3$

PC-3

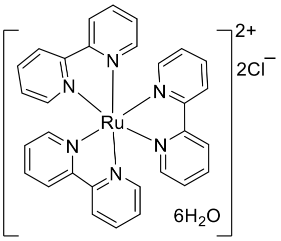

$Ru(bpy)_3Cl_2\cdot 6H_2O$

PC-2

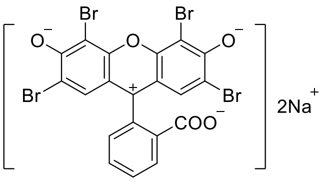

Eosin Y

PC-6

Reaction conditions: **1a** (0.1 mmol), **2a** (0.1 mmol), **3a** (0.15 mmol), CO (40 bar), $CHCl_3$ (1.0 mL), **PC** (1 mol%[a], 1.5 mol%[b], 2 mol%[c]), r.t, 15 h, 400-500 nm. Determined by GC with hexadecane as internal standard.

Table 2. Optimization of pressure (CO)

Entry	pressure(bar)	Yield (%)
1	30	10
2	35	30
3	40	49
4	45	51
5	50	50

Reaction conditions: **1a** (0.1 mmol), **2a** (0.1 mmol), **3a** (0.15 mmol), CO, $CHCl_3$ (1.0 mL), **PC-2** (1.5 mol%), r.t, 15 h, 400-500 nm. Determined by GC with hexadecane as internal standard.

Table 3. Optimization of wavelength

Entry	Wavelength (nm)	Yield (%)
1	320-400	-
2	415	-
3	440	38
4	460	40
5	490	-
6	550	-
7	400-500	49
8	400-700	-

Reaction conditions: **1a** (0.1 mmol), **2a** (0.1 mmol), **3a** (0.15 mmol), CO (40 bar), $CHCl_3$ (1.0 mL), **PC-2** (1.5 mol%), r.t, 15 h, 400-500 nm. Determined by GC with hexadecane as internal standard.

Table 4. Optimization of solvent

Entry	Solvent	Yield (%)
1[a]	$CHCl_3$	49
2[a]	THF	32
3[a]	DMF	-
4[a]	DMAc	-
5[a]	DCE	43

6[a]	DCM	40
7[a]	Tolune	-
8[a]	MeCN	trace
9[b]	$CHCl_3$	40
10[c]	$CHCl_3$	48

Reaction conditions: **1a** (0.1 mmol), **2a** (0.1 mmol), **3a** (0.15 mmol), CO (40 bar), Solvent (0.1M[a], 0.2M[b], 0.05 M[c]), **PC-2** (1.5 mol%), r.t, 15 h, 400-500 nm. Determined by GC with hexadecane as internal standard.

Table 5. Optimization of equivalent ratio

Entry	**1a** : **2a** : **3a**	Yield (%)
1	1.0 : 1.0 : 1.0	46
2	1.0 : 1.0 : 1.1	52
3	1.0 : 1.0 : 1.2	55
4	1.0 : 1.0 : 1.5	45
6	1.0 : 1.0 : 2.0	40

Reaction conditions: **1a**, **2a**, **3a**, CO (40 bar), $CHCl_3$ (1.0 mL), **PC-2** (1.5 mol%), r.t, 15 h, 400-500 nm. Determined by GC with hexadecane as internal standard.

Table 6. Optimization of temperature

Entry	Temperature (^oC)	Yield (%)
1	r.t.	55
2	30	60
3	40	58
4	50	41

Reaction conditions: **1a** (0.1 mmol), **2a** (0.1 mmol), **3a** (0.12 mmol), CO (40 bar), $CHCl_3$ (1.0 mL), **PC-2** (1.5 mol%), 15 h, 400-500 nm. Determined by GC with hexadecane as internal standard.

Table 7. Optimization of time

Entry	Time (h)	Yield (%)
1	12	40
2	15	60
3	20	72
4	24	89 (82)

5	30	90

Reaction conditions: **1a** (0.1 mmol), **2a** (0.1 mmol), **3a** (0.12 mmol), CO (40 bar), $CHCl_3$ (1.0 mL), **PC-2** (1.5 mol%), 30 ^{o}C, 400-500 nm. Determined by GC with hexadecane as internal standard. Isolated yield is shown in parentheses.

4. Characterization and procedure of α-Aminoketones products 4 and synthetic transformations products

4.1 General diacylation procedure for the synthesis of α-Aminoketones 4

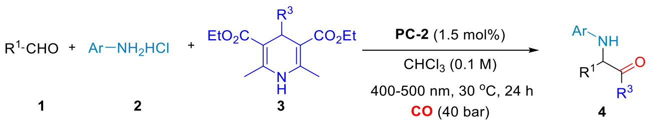

A 4 mL screw-cap vial was charged with Hantzsch ester **3** (0.24 mmol, 1.2 equiv), $ArNH_2$.HCl **2** (0.2 mmol, 1.0 equiv), **PC-2** (2.8 mg, 1.5 mol%), and an oven-dried stirring bar. The vial was closed with a Teflon septum and cap and connected to the atmosphere via a needle. After replacing the nitrogen in the vial three times, Aldehyde (0.2 mmol, 1.0 equiv) using a microinjector. Then, $CHCl_3$ (2 mL) was added was added using a injector. The vial was then moved to a cannula and transferred into a 300 mL photoautoclave (manufactured by Parr Instrument Company®), under a nitrogen atmosphere. At room temperature, the autoclave was washed with CO three times and charged with 40 bar of CO. The autoclave was placed on a heating plate equipped with a magnetic stirrer and an aluminum block. The reaction mixture was allowed to react at 30 °C under UV-A (400-500 nm) for 24 hours. After the reaction was complete, the pressure of the autoclave was carefully released, and the residual CO was washed away with nitrogen. The solvent was removed under vacuum, and the product was purified by column chromatography on silica gel using petroleum ether and ethyl acetate (50:1) to afford the corresponding product **4**.

4.2 General procedure for the synthesis of 1-((4-chlorophenyl)amino)-2-cyclohexyl-1-phenylbut-3-en-2-ol 7

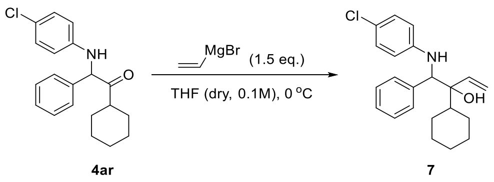

To a stirred solution of **4ar** (33.0 mg, 0.1 mmol) in THF (dry, 1 mL) at °C was added vinyl magnesium bromide (1.5 eq, 0.15 mmol) slowly. The mixture was stirred vigorously for 1.5. The crude product was purified by column chromatography on silica gel using petroleum ether and ethyl acetate (10:1) as to afford **7** (31.9 mg, 90% yield).

4.3 General procedure for the synthesis of 2-((4-chlorophenyl)amino)-1-cyclohexyl-2-phenylethan-1-ol 8

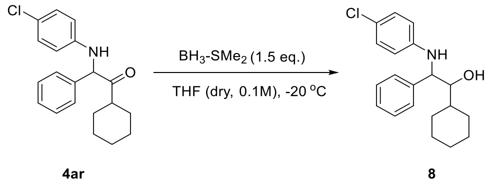

To a stirred solution of **4ar** (33.0 mg, 0.1 mmol) in THF (dry, 1 mL) at –20 °C was added Borane-methyl sulfide complex (1.5 eq, 0.15 mmol) slowly. The mixture was stirred vigorously for 4 h. The crude product was purified by column chromatography on silica gel using petroleum ether and ethyl acetate (10:1) to afford **8** (26.3 mg, 80% yield).

4.4 General procedure for the synthesis of 3-(4-chlorophenyl)-5-cyclohexyl-4-phenyloxazolidin-2-one 9

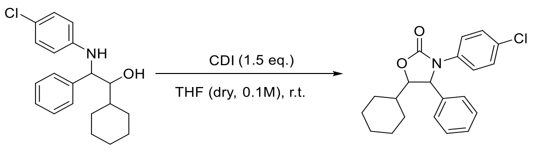

To a stirred solution of **8** (32.9 mg, 0.1 mmol) in THF (dry, 1 mL) at r.t. was added CDI (1.5 eq, 0.15 mmol) slowly. The mixture was stirred vigorously for 6 h. The crude product was purified by column chromatography on silica gel using petroleum ether and ethyl acetate (10:1) to afford **9** (27.2 mg, 85% yield).

4.5 General procedure for the synthesis of 1-((4-chlorophenyl)amino)-2-cyclohexyl-1-phenylbut-3-yn-2-ol 10

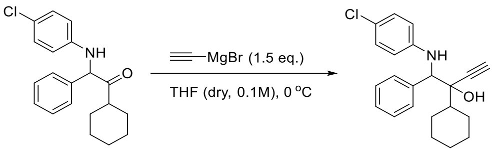

To a stirred solution of **4ar** (33.0 mg, 0.1 mmol) in THF (dry, 1 mL) at 0 °C was added Ethynylmagnesium bromide (1.5 eq, 0.15 mmol) slowly. The mixture was stirred vigorously for 4 h. The crude product was purified by column chromatography on silica gel using petroleum ether and ethyl acetate (10:1) to afford **10** (30.3 mg, 86% yield).

4.6 General procedure for the synthesis of 1-((4-chlorophenyl)amino)-2-cyclohexyl-1-phenylpropan-2-ol 11

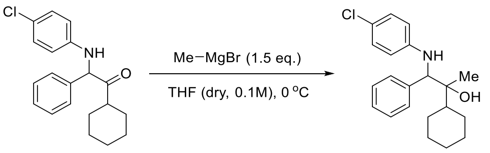

To a stirred solution of **4ar** (33.0 mg, 0.1 mmol) in THF (dry, 1 mL) at 0 °C was added Methylmagnesium bromide (1.5 eq, 0.15 mmol) slowly. The mixture was stirred vigorously for 4 h. The crude product was purified by column chromatography on silica gel using petroleum ether and ethyl acetate (10:1) to afford **11** (31.2 mg, 91% yield).

4.7 General procedure for the synthesis of 1-((4-chlorophenyl)amino)-2-cyclohexyl-1-phenylhexan-2-ol 12

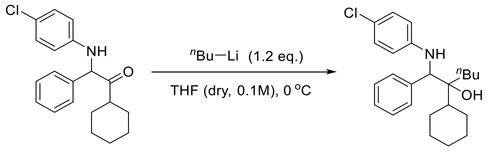

To a stirred solution of **4ar** (33.0 mg, 0.1 mmol) in THF (dry, 1 mL) at 0 oC was added *n*-Butyllithium (1.2 eq, 0.12 mmol) slowly. The mixture was stirred vigorously for 2 h. The crude product was purified by column chromatography on silica gel using petroleum ether and ethyl acetate (10:1) to afford **11** (32.7 mg, 85% yield).

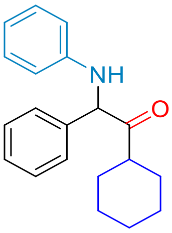

4a

1-cyclohexyl-2-phenyl-2-(phenylamino)ethan-1-one (4a)

Chromatography Pentane/EA = 50:1 (v/v), 48.0 mg (82%), White solid.

^{1}H NMR (300 MHz, $CDCl_3$) δ 7.38 – 6.45 (m, 10H), 5.02 (s, 1H), 2.52 – 2.42 (m, 1H), 1.86 – 0.90 (m, 10H).
^{13}C NMR (75 MHz, $CDCl_3$) δ 208.5, 145.6, 136.4, 134.1, 129.3, 129.2, 117.8, 113.3, 65.4, 47.3, 29.7, 28.3, 25.7, 25.5, 25.0.
HRMS(ESI-TOF) m/z: calcd for $[M^{+}]H^{+}$ $C_{20}H_{23}NO$ 294.1853, found: 294.4851.

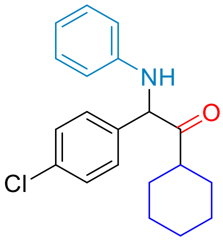

4b

2-(4-chlorophenyl)-1-cyclohexyl-2-(phenylamino)ethan-1-one (4b)

Chromatography Pentane/EA = 50:1 (v/v), 52.4 mg (80%), White solid.

^{1}H NMR (300 MHz, $CDCl_3$) δ 7.33 – 6.43 (m, 9H), 5.01 (s, 1H), 2.49 – 2.39 (m, 1H), 1.85 – 0.90 (m, 10H).
^{13}C NMR (75 MHz, $CDCl_3$) δ 208.5, 145.6, 136.4, 134.1, 129.3, 129.2, 117.8, 113.3, 65.4, 47.3, 29.7, 28.3, 25.7, 25.5, 25.0.
HRMS(ESI-TOF) m/z: calcd for $[M^{+}]H^{+}$ $C_{20}H_{22}ClNO$ 328.1463, found: 328.1469.

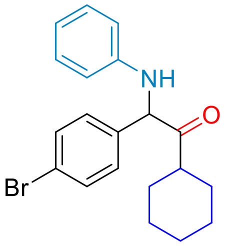

4c

2-(4-bromophenyl)-1-cyclohexyl-2-(phenylamino)ethan-1-one (4c)

Chromatography Pentane/EA = 50:1 (v/v), 60.0 mg (81%), 95% purity, White solid.

^{1}H NMR (300 MHz, CDCl$_3$) δ 7.43 – 6.42 (m, 9H), 5.00 (s, 1H), 2.49 – 2.39 (m, 1H), 1.87 – 0.91 (m, 10H).
^{13}C NMR (75 MHz, CDCl$_3$) δ 208.4, 145.6, 136.9, 132.3, 129.6, 129.2, 122.3, 117.8, 113.3, 65.5, 47.3, 29.7, 28.2, 25.8, 25.5, 25.0.
HRMS(ESI-TOF) m/z: calcd for [M$^+$]H$^+$ $C_{20}H_{22}BrNO_2$ 372.0958, found: 372.0961.

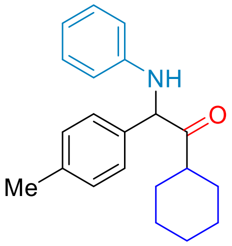

4d

1-cyclohexyl-2-(phenylamino)-2-(p-tolyl)ethan-1-one (4d)

Chromatography Pentane/EA = 50:1 (v/v), 52.2 mg (85%), 95% purity, White solid.

^{1}H NMR (300 MHz, CDCl$_3$) δ 7.25 – 6.46 (m, 9H), 4.99 (s, 1H), 2.52 – 2.42 (m, 1H), 2.24 (s, 3H), 1.85 – 0.92 (m, 10H).
^{13}C NMR (75 MHz, CDCl$_3$) δ 209.3, 146.1, 137.9, 134.6, 129.8, 129.1, 127.9, 117.4, 113.2, 65.7, 47.1, 29.7, 28.2, 25.8, 25.6, 25.1, 21.1.
HRMS(ESI-TOF) m/z: calcd for [M$^+$]H$^+$ $C_{21}H_{25}NO$ 308.2009, found: 308.2018.

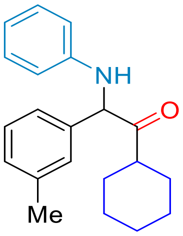

4e

1-cyclohexyl-2-(phenylamino)-2-(m-tolyl)ethan-1-one (4e)

Chromatography Pentane/EA = 50:1 (v/v), 52.8 mg (86%), White solid.

^{1}H NMR (300 MHz, CDCl$_3$) δ 7.25 – 6.45 (m, 9H), 5.00 (s, 1H), 2.52 – 2.42 (m, 1H), 2.25 (s, 3H), 1.86 – 0.83 (m, 10H).
^{13}C NMR (75 MHz, CDCl$_3$) δ 209.3, 146.1, 137.9, 134.6, 129.8, 129.1, 128.9, 128.9, 127.9, 117.4, 113.2, 65.7, 47.2, 29.7, 28.2, 25.8, 25.6, 25.1, 21.1.
HRMS(ESI-TOF) m/z: calcd for [M$^+$]H$^+$ $C_{21}H_{25}NO$ 308.2009, found: 308.2011.

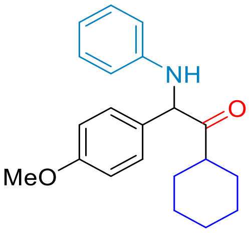

4f

***1-cyclohexyl-2-(4-methoxyphenyl)-2-(phenylamino)ethan-1-one* (4f)**

Chromatography Pentane/EA = 50:1 (v/v), 50.4 mg (78%), White solid.

^{1}H NMR (300 MHz, $CDCl_3$) δ 7.27 – 6.46 (m, 9H), 4.98 (s, 1H), 3.71 (s, 3H), 2.51 – 2.41 (m, 1H), 1.84 – 0.96 (m, 10H).
^{13}C NMR (75 MHz, $CDCl_3$) δ 209.3, 159.5, 145.9, 129.4, 129.2, 129.1, 117.6, 114.5, 113.4, 65.5, 55.2, 47.2, 29.6, 28.3, 25.8, 25.6, 25.1.
HRMS(ESI-TOF) m/z: calcd for $[M^{+}]H^{+}$ $C_{21}H_{25}NO$ 324.1958, found: 324.1955.

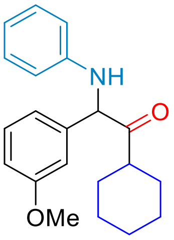

4g

***1-cyclohexyl-2-(3-methoxyphenyl)-2-(phenylamino)ethan-1-one* (4g)**

Chromatography Pentane/EA = 50:1 (v/v), 51.7 mg (80%), White solid.

^{1}H NMR (300 MHz, $CDCl_3$) δ 7.37 – 6.02 (m, 9H), 5.02 (s, 1H), 3.62 (s, 3H), 2.51 – 2.42 (m, 1H), 1.86 – 0.92 (m, 10H).
^{13}C NMR (75 MHz, $CDCl_3$) δ 208.9, 160.6, 147.4, 137.7, 129.9, 129.1, 128.3, 128.0, 106.4, 102.8, 99.2, 66.1, 54.9, 47.2, 29.7, 28.3, 25.8, 25.5, 25.1.
HRMS(ESI-TOF) m/z: calcd for $[M^{+}]H^{+}$ $C_{21}H_{25}NO_2$ 324.1958, found: 324.1957.

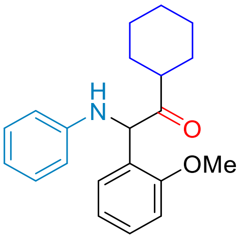

4h

***1-cyclohexyl-2-(2-methoxyphenyl)-2-(phenylamino)ethan-1-one* (4h)**

Chromatography Pentane/EA = 50:1 (v/v), 44.6 mg (69%), Yellow solid.

^{1}H NMR (300 MHz, $CDCl_3$) δ 7.20 – 6.45 (m, 9H), 5.63 (s, 1H), 3.92 (s, 3H), 2.45 – 2.35 (m, 1H), 1.93 – 0.95 (m, 10H).
^{13}C NMR (75 MHz, $CDCl_3$) δ 209.4, 157.2, 146.1, 129.2, 129.1, 128.4, 125.8, 121.3, 117.3, 113.1, 110.8, 58.2, 55.6, 47.1, 29.8, 28.1, 26.0, 25.7, 25.2.
HRMS(ESI-TOF) m/z: calcd for $[M^{+}]H^{+}$ $C_{21}H_{25}NO_2$ 324.1958, found: 324.1952.

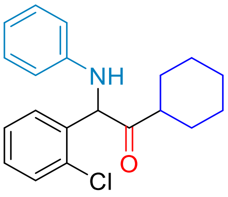

4i

1-(2-chlorophenyl)-3-methyl-1-(phenylamino)butan-2-one **(4i)**

Chromatography Pentane/EA = 50:1 (v/v), 34.5 mg (60%), White solid.

^{1}H NMR (300 MHz, $CDCl_3$) δ 7.39 – 6.46 (m, 9H), 5.67 (s, 1H), 2.79 – 2.67 (m, 9H), 1.12 (d, J = 7.0 Hz, 3H), 0.76 (d, J = 6.6 Hz, 3H).
^{13}C NMR (101 MHz, $CDCl_3$) δ 212.3, 199.2, 140.7, 136.5, 135.8, 132.7, 129.8, 128.9, 128.4, 127.8, 50.7, 48.2, 45.5, 44.9, 30.1, 28.3, 28.3, 25.8, 25.6, 25.6, 22.3, 22.3.
HRMS(ESI-TOF) m/z: calcd for $[M^+]H^+$ $C_{17}H_{18}ClNO$ 288.1150, found: 288.1143.

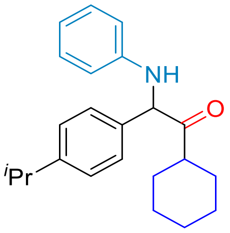

4j

1-cyclohexyl-2-(4-isopropylphenyl)-2-(phenylamino)ethan-1-one (4j)

Chromatography Pentane/EA = 50:1 (v/v), 46.9 mg (70%), White solid.

^{1}H NMR (300 MHz, $CDCl_3$) δ 7.27 – 6.46 (m, 9H), 5.00 (s, 1H), 2.87 – 2.73 (m, 1H), 2.52 – 2.42 (m, 1H), 1.86 – 1.18 (m, 7H), 1.14 (dd, J = 7.0, 3.4 Hz, 6H), 1.09 – 0.93 (m, 3H).
^{13}C NMR (75 MHz, $CDCl_3$) δ 209.4, 148.9, 146.2, 134.9, 129.1, 127.9, 127.1, 117.4, 113.2, 65.7, 47.2, 33.7, 29.7, 28.2, 25.8, 25.6, 25.1, 23.9, 23.8.
HRMS(ESI-TOF) m/z: calcd for $[M^+]H^+$ $C_{23}H_{29}NO$ 336.2322, found: 336.2328.

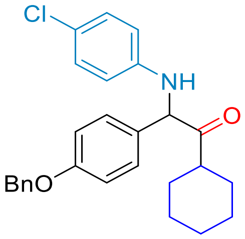

4k

2-(4-(benzyloxy)phenyl)-2-((4-chlorophenyl)amino)-1-cyclohexylethan-1-one **(4k)**

Chromatography Pentane/EA = 50:1 (v/v), 59.0 mg (68%), White solid.

^{1}H NMR (300 MHz, $CDCl_3$) δ 7.35 – 6.35 (m, 13H), 4.94 (s, 2H), 4.92 (s, 1H), 2.49 – 2.39 (m, 1H), 1.83 – 0.77 (m, 10H).
^{13}C NMR (75 MHz, $CDCl_3$) δ 208.9, 158.8, 144.5, 136.6, 129.2, 129.1, 128.9, 128.6, 128.0, 127.5, 121.9, 115.5, 114.4, 70.1, 65.3, 47.2, 29.6, 28.3, 25.7, 25.5, 25.1.
HRMS(ESI-TOF) m/z: calcd for $[M^+]Na^+$ $C_{27}H_{28}ClNO_2$ 456.1700, found: 456.1705.

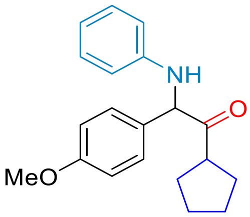

4l

1-cyclopentyl-2-(4-methoxyphenyl)-2-(phenylamino)ethan-1-one (4l)

Chromatography Pentane/EA = 50:1 (v/v), 37.1 mg (60%), White solid.

^{1}H NMR (300 MHz, $CDCl_3$) δ 7.29 – 6.46 (m, 9H), 4.95 (s, 1H), 3.71 (s, 3H), 2.97 – 2.86 (m, 1H), 1.90 – 1.12 (m, 8H).
^{13}C NMR (75 MHz, $CDCl_3$) δ 209.5, 159.5, 146.1, 129.8, 129.2, 129.1, 117.4, 114.5, 113.4, 66.6, 55.2, 47.7, 31.1, 29.6, 26.2, 26.1.
HRMS(ESI-TOF) m/z: calcd for $[M^{+}]H^{+}$ $C_{20}H_{23}NO_2$ 310.1802, found: 310.1805.

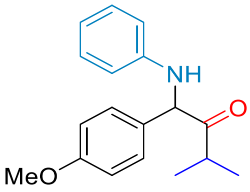

4m

1-(4-methoxyphenyl)-3-methyl-1-(phenylamino)butan-2-one (4m)

Chromatography Pentane/EA = 50:1 (v/v), 38.5 mg (68%), White solid.

^{1}H NMR (300 MHz, $CDCl_3$) δ 7.29 – 6.46 (m, 9H), 5.00 (s, 1H), 3.71 (s, 3H), 2.79 – 2.70 (m, 1H), 1.06 (d, *J* = 7.0 Hz, 3H), 0.73 (d, *J* = 6.7 Hz, 3H).
^{13}C NMR (75 MHz, $CDCl_3$) δ 210.3, 159.5, 146.0, 129.6, 129.2, 129.1, 117.5, 114.5, 113.3, 65.4, 55.2, 36.9, 19.5, 18.4.
HRMS(ESI-TOF) m/z: calcd for $[M^{+}]H^{+}$ $C_{18}H_{21}NO_2$ 284.1645, found: 284.1647.

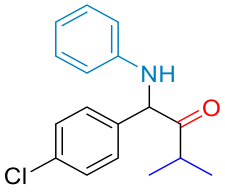

4n

1-(4-chlorophenyl)-3-methyl-1-(phenylamino)butan-2-one (4n)

Chromatography Pentane/EA = 50:1 (v/v), 46.6 mg (81%), White solid.

^{1}H NMR (300 MHz, $CDCl_3$) δ 7.33 – 6.43 (m, 9H), 5.02 (s, 1H), 2.79 – 2.66 (m, 1H), 1.08 (d, *J* = 7.0 Hz, 3H), 0.74 (d, *J* = 6.6 Hz, 3H).
^{13}C NMR (75 MHz, $CDCl_3$) δ 209.5, 145.6, 136.5, 134.1, 129.3, 129.3, 129.2, 117.8, 113.3, 65.4, 37.1, 19.5, 18.3.
HRMS(ESI-TOF) m/z: calcd for $[M^{+}]H^{+}$ $C_{17}H_{18}ClNO$ 288.1150, found: 288.1158.

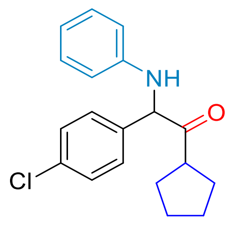

4o

2-(4-chlorophenyl)-1-cyclopentyl-2-(phenylamino)ethan-1-one (4o)

Chromatography Pentane/EA = 50:1 (v/v), 38.3 mg (61%), White solid.

^{1}H NMR (300 MHz, CDCl$_3$) δ 7.33 – 6.42 (m, 9H), 4.97 (s, 1H), 2.92 – 7.87 (m, 1H), 1.88 – 1.15 (m, 8H).
^{13}C NMR (75 MHz, CDCl$_3$) δ 208.6, 145.7, 136.6, 134.0, 129.3, 129.2, 117.7, 113.3, 66.6, 47.8, 31.1, 29.6, 26.2, 26.1.
HRMS(ESI-TOF) m/z: calcd for [M$^+$]H$^+$ $C_{19}H_{20}ClNO$ 314.1306, found: 314.1315.

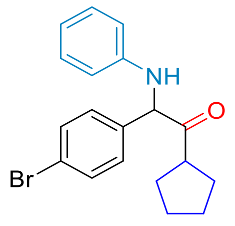

4p

2-(4-bromophenyl)-1-cyclopentyl-2-(phenylamino)ethan-1-one (4p)

Chromatography Pentane/EA = 50:1 (v/v), 35.8 mg (50%), White solid.

^{1}H NMR (300 MHz, CDCl$_3$) δ 7.44 – 6.42 (m, 9H), 4.96 (s, 1H), 2.95 – 2.84 (m, 1H), 1.92 – 1.13 (m, 8H).
^{13}C NMR (75 MHz, CDCl$_3$) δ 208.5, 145.7, 137.2, 132.3, 129.7, 129.2, 122.2, 117.8, 113.3, 66.7, 47.8, 31.1, 29.6, 26.2, 26.1.
HRMS(ESI-TOF) m/z: calcd for [M$^+$]H$^+$ $C_{19}H_{20}BrNO$ 358.0801, found: 358.0805.

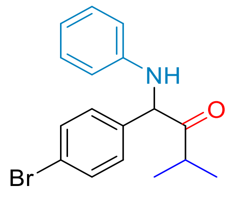

4q

1-(4-bromophenyl)-3-methyl-1-(phenylamino)butan-2-one (4q)

Chromatography Pentane/EA = 50:1 (v/v), 46.5 mg (70%), White solid.

^{1}H NMR (300 MHz, CDCl$_3$) δ 7.44 – 6.43 (m, 9H), 5.02 (s, 1H), 2.78 – 2.66 (m, 1H), 1.08 (d, J = 7.0 Hz, 3H), 0.75 (d, J = 6.7 Hz, 3H).
^{13}C NMR (75 MHz, CDCl$_3$) δ 209.4, 145.6, 137.1, 132.3, 129.6, 129.2, 122.3, 117.8, 113.3, 65.5, 37.1, 19.5, 18.3.
HRMS(ESI-TOF) m/z: calcd for [M$^+$]H$^+$ $C_{17}H_{18}BrNO$ 332.0645, found: 332.0645.

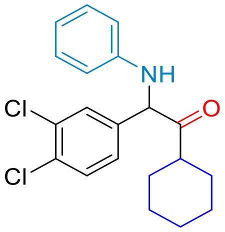

4r

***1-cyclohexyl-2-(3,4-dichlorophenyl)-2-(phenylamino)ethan-1-one* (4r)**

Chromatography Pentane/EA = 50:1 (v/v), 52.8 mg (73%), 95% purity, White solid.

^{1}H NMR (300 MHz, CDCl$_3$) δ 7.47 – 6.42 (m, 8H), 4.98 (s, 1H), 2.49 – 2.39 (m, 1H), 1.86 – 1.01 (m, 10H).
^{13}C NMR (75 MHz, CDCl$_3$) δ 207.8, 145.4, 138.4, 133.4, 132.5, 131.1, 129.7, 129.3, 127.2, 119.6, 118.0, 113.9, 113.3, 64.9, 47.4, 29.7, 28.2, 25.7, 25.5, 24.9.
HRMS(ESI-TOF) m/z: calcd for [M$^+$]H$^+$ $C_{20}H_{21}Cl_2NO$ 362.1073, found: 362.1074.

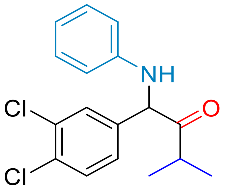

4s

***1-(3,4-dichlorophenyl)-3-methyl-1-(phenylamino)butan-2-one* (4s)**

Chromatography Pentane/EA = 50:1 (v/v), 42.4 mg (66%), White solid.

^{1}H NMR (300 MHz, CDCl$_3$) δ 7.48 (d, J = 2.1 Hz, 1H), 7.38 – 6.43 (m, 7H), 5.00 (s, 1H), 2.80 – 2.66 (m, 10H), 1.09 (d, J = 7.0 Hz, 3H), 0.78 (d, J = 6.7 Hz, 3H).
^{13}C NMR (75 MHz, CDCl$_3$) δ 208.9, 145.4, 138.5, 133.4, 132.5, 131.1, 129.8, 129.3, 127.1, 118.1, 113.3, 64.9, 37.1, 19.5, 18.2.
HRMS(ESI-TOF) m/z: calcd for [M$^+$]H$^+$ $C_{17}H_{17}Cl_2NO$ 322.0760, found: 322.0761.

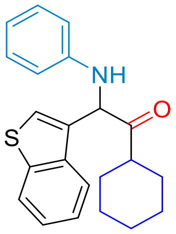

4t

***2-(benzo[b]thiophen-3-yl)-1-cyclohexyl-2-(phenylamino)ethan-1-one* (4t)**

Chromatography Pentane/EA = 50:1 (v/v), 38.4 mg (55%), 95% purity, White solid.

^{1}H NMR (300 MHz, CDCl$_3$) δ 7.88 – 6.51 (m, 10H), 5.47 (s, 1H), 2.49 – 2.39 (m, 1H), 1.89 – 0.79 (m, 10H).
^{13}C NMR (75 MHz, CDCl$_3$) δ 208.9, 146.3, 137.3, 137.2, 132.1, 129.2, 126.2, 124.8, 124.6, 123.2, 121.9, 117.9, 113.4, 61.0, 47.2, 29.9, 28.2, 25.8, 25.5, 25.0.
HRMS(ESI-TOF) m/z: calcd for [M$^+$]H$^+$ $C_{22}H_{23}NOS$ 350.1573, found: 350.1577.

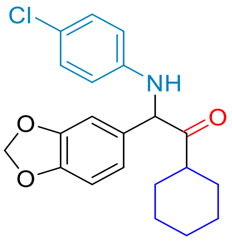

4u

2-(benzo[d][1,3]dioxol-5-yl)-2-((4-chlorophenyl)amino)-1-cyclohexylethan-1-one (4u)

Chromatography Pentane/EA = 50:1 (v/v), 38.6 mg (52%), 95% purity, White solid.

^{1}H NMR (300 MHz, $CDCl_3$) δ 6.96 – 5.86 (m, 7H), 6.96 – 5.86 (m, 2H), 2.52 – 2.43 (m, 1H), 1.85 – 0.96 (m, 10H).
^{13}C NMR (75 MHz, $CDCl_3$) δ 208.6, 190.2, 148.4, 147.7, 144.4, 131.0, 128.9, 122.1, 121.8, 114.4, 108.7, 107.7, 101.3, 65.4, 47.0, 29.6, 28.3, 25.7, 25.5, 25.0.
HRMS(ESI-TOF) m/z: calcd for $[M^{+}]H^{+}$ $C_{21}H_{22}ClNO_3$ 372.1361, found: 372.1366.

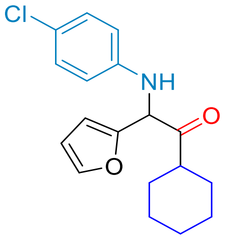

4v

2-((4-chlorophenyl)amino)-1-cyclohexyl-2-(furan-2-yl)ethan-1-one (4v)

Chromatography Pentane/EA = 50:1 (v/v), 31.7 mg (50%), White solid.

^{1}H NMR (300 MHz, $CDCl_3$) δ 7.34 – 6.25 (m, 7H), 5.12 (s, 1H), 1.84 (s, 1H), 2.54 – 2.45 (m, 1H), 1.84 – 1.08 (m, 10H).
^{13}C NMR (75 MHz, $CDCl_3$) δ 206.6, 149.9, 144.4, 142.9, 129.0, 122.7, 114.6, 111.1, 109.0, 59.6, 47.4, 29.4, 28.0, 25.7, 25.6, 25.2.
HRMS(ESI-TOF) m/z: calcd for $[M^{+}]H^{+}$ $C_{18}H_{20}ClNO_2$ 318.1255, found: 318.1258.

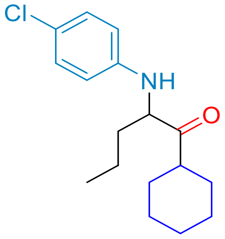

4w

2-((4-chlorophenyl)amino)-1-cyclohexylpentan-1-one (4w)

Chromatography Pentane/EA = 50:1 (v/v), 35.2 mg (60%), White solid.

^{1}H NMR (300 MHz, $CDCl_3$) δ 7.05 – 6.41 (m, 4H), 4.03 – 3.99 (m, 1H), 3.05 – 2.95 (m, 1H), 1.84 – 0.75 (m, 17H).
^{13}C NMR (75 MHz, $CDCl_3$) δ 214.4, 156.1, 129.1, 120.4, 114.4, 62.4, 47.5, 33.7, 30.9, 29.3, 26.2, 26.1, 18.6, 14.0, 13.9.
HRMS(ESI-TOF) m/z: calcd for $[M^{+}]H^{+}$ $C_{17}H_{24}ClNO$ 294.1619, found: 294.1621.

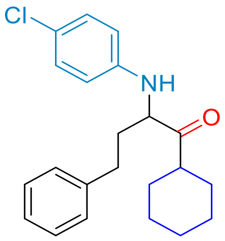

4x

2-((4-chlorophenyl)amino)-1-cyclohexyl-4-phenylbutan-1-one **(4x)**

Chromatography Pentane/EA = 50:1 (v/v), 44.1 mg (62%), White solid.

^{1}H NMR (300 MHz, $CDCl_3$) δ 7.24 – 6.41 (m, 9H), 4.06 – 4.02 (m, 1H), 3.03 – 2.91 (m, 1H), 2.69 – 2.50 (m, 2H), 2.21 – 2.09 (m, 1H), 1.93 – 1.19 (m, 11H).
^{13}C NMR (75 MHz, $CDCl_3$) δ 213.9, 140.8, 139.9, 133.2, 129.2, 128.6, 128.4, 126.2, 114.7, 62.1, 47.7, 46.5, 33.1, 31.5, 30.8, 29.2, 26.1, 26.1.
HRMS(ESI-TOF) m/z: calcd for $[M^{+}]H^{+}$ $C_{22}H_{26}ClNO$ 356.1776, found: 356.1778.

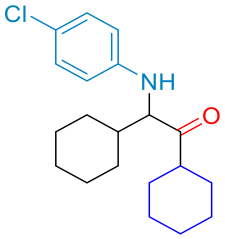

4y

2-((4-chlorophenyl)amino)-1,2-dicyclohexylethan-1-one **(4y)**

Chromatography Pentane/EA = 50:1 (v/v), 62.1 mg (93%), 95% purity, White solid.

^{1}H NMR (300 MHz, $CDCl_3$) δ 7.00 (d, *J* = 9.0 Hz, 2H), 6.45 (d, *J* = 8.9 Hz, 2H), 3.86 (d, *J* = 5.0 Hz, 1H), 2.52 – 2.42 (m, 1H), 1.72 – 0.93 (m, 21H).
^{13}C NMR (75 MHz, $CDCl_3$) δ 214.0, 146.7, 129.0, 122.2, 114.7, 66.9, 48.3, 40.6, 30.7, 29.2, 28.1, 27.7, 26.2, 26.1, 26.0, 25.9, 25.7, 25.3.
HRMS(ESI-TOF) m/z: calcd for $[M^{+}]H^{+}$ $C_{20}H_{28}ClNO$ 334.1932, found: 334.1927.

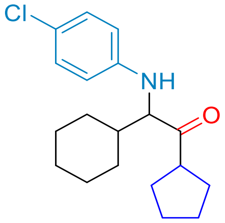

4z

2-((4-chlorophenyl)amino)-2-cyclohexyl-1-cyclopentylethan-1-one **(4z)**

Chromatography Pentane/EA = 50:1 (v/v), 51.1 mg (80%), 95% purity, White solid.

^{1}H NMR (300 MHz, $CDCl_3$) δ 7.01 (d, *J* = 9.0 Hz, 2H), 6.45 (d, *J* = 9.0 Hz, 2H), 3.80 (d, *J* = 5.3 Hz, 1H), 3.02 – 2.91 (m, 1H), 1.79 – 0.78 (m, 19H).
^{13}C NMR (75 MHz, $CDCl_3$) δ 214.5, 146.6, 129.0, 122.2, 114.6, 68.1, 48.5, 40.6, 30.6, 30.4, 29.3, 28.3, 26.3, 26.2, 26.2, 26.1, 26.0.
HRMS(ESI-TOF) m/z: calcd for $[M^{+}]H^{+}$ $C_{19}H_{26}ClNO$ 320.1776, found: 320.1779.

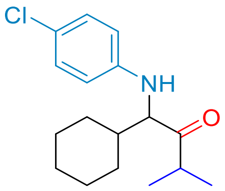

4aa

1-((4-chlorophenyl)amino)-1-cyclohexyl-3-methylbutan-2-one (4aa)

Chromatography Pentane/EA = 50:1 (v/v), 56.4 mg (96%), 95% purity, White solid.

^{1}H NMR (300 MHz, $CDCl_3$) δ 7.01 (d, *J* = 8.9 Hz, 2H), 6.47 (d, *J* = 8.9 Hz, 2H), 3.88 (d, *J* = 5.2 Hz, 1H), 2.78 – 2.69 (m, 1H), 1.72 – 1.03 (m, 11H), 0.97 (d, *J* = 6.8 Hz, 6H).
^{13}C NMR (75 MHz, $CDCl_3$) δ 214.8, 146.5, 134.2, 129.5, 129.1, 122.4, 114.8, 66.9, 40.7, 38.1, 30.7, 28.2, 26.2, 26.1, 26.0, 18.9, 17.7.
HRMS(ESI-TOF) m/z: calcd for $[M^{+}]H^{+}$ $C_{17}H_{24}ClNO$ 294.1619, found: 335:2008.

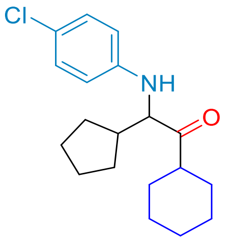

4ab

2-((4-chlorophenyl)amino)-1-cyclohexyl-2-cyclopentylethan-1-one (4ab)

Chromatography Pentane/EA = 50:1 (v/v), 56.2 mg (88%), White solid.

^{1}H NMR (300 MHz, $CDCl_3$) δ 7.02 (d, *J* = 9.0 Hz, 2H), 6.43 (d, *J* = 9.0 Hz, 2H), 3.90 (d, *J* = 6.7 Hz, 1H), 2.75 – 2.62 (m, 1H), 2.55 – 2.45 (m, 1H), 1.93 – 1.08 (m, 18H).
^{13}C NMR (75 MHz, $CDCl_3$) δ 213.4, 146.6, 129.0, 122.3, 114.4, 65.8, 47.2, 37.6, 29.4, 27.8, 25.9, 25.6, 25.3, 25.2, 24.3, 18.3.
HRMS(ESI-TOF) m/z: calcd for $[M^{+}]H^{+}$ $C_{19}H_{26}ClNO$ 320.8805, found: 320.8809.

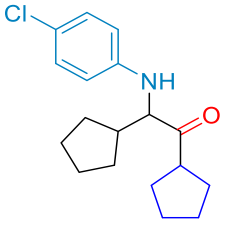

4ac

2-((4-chlorophenyl)amino)-1,2-dicyclopentylethan-1-one (4ac)

Chromatography Pentane/EA = 50:1 (v/v), 47.7 mg (78%), White solid.

^{1}H NMR (400 MHz, $CDCl_3$) δ 7.02 (d, *J* = 8.8 Hz, 2H), 6.43 (d, *J* = 8.8 Hz, 2H), 3.87 (d, *J* = 7.0 Hz, 1H), 3.00 – 2.92 (m, 1H), 2.73 – 2.63 (m, 1H), 1.94 – 1.18 (m, 7H).
^{13}C NMR (101 MHz, $CDCl_3$) δ 214.0, 146.4, 129.1, 122.3, 114.3, 66.9, 47.4, 37.5, 30.9, 29.4, 26.2, 26.1, 25.2, 24.5, 18.3.
HRMS(ESI-TOF) m/z: calcd for $[M^{+}]H^{+}$ $C_{18}H_{24}ClNO$ 306.1619, found: 306.1621.

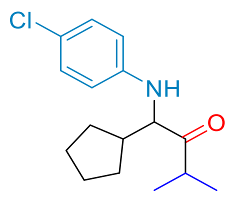

4ad

1-((4-chlorophenyl)amino)-1-cyclopentyl-3-methylbutan-2-one (4ad)

Chromatography Pentane/EA = 50:1 (v/v), 49.2 mg (88%), White solid.

^{1}H NMR (300 MHz, $CDCl_3$) δ 7.02 (d, *J* = 9.0 Hz, 2H), 6.44 (d, *J* = 8.9 Hz, 2H), 3.92 (d, *J* = 6.8 Hz, 1H), 2.83 – 2.61 (m, 2H), 1.94 – 1.18 (m, 8H), 0.97 (d, *J* = 7.0 Hz, 3H), 0.92 (d, *J* = 6.7 Hz, 3H).
^{13}C NMR (75 MHz, $CDCl_3$) δ 214.3, 146.5, 129.1, 122.4, 114.4, 65.8, 37.7, 36.9, 25.2, 24.4, 19.2, 18.3, 17.9.
HRMS(ESI-TOF) m/z: calcd for $[M^+]H^+$ $C_{16}H_{22}ClNO$ 280.1463, found: 280.1466.

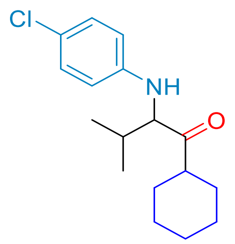

4ae

2-((4-chlorophenyl)amino)-1-cyclohexyl-3-methylbutan-1-one (4ae)

Chromatography Pentane/EA = 50:1 (v/v), 47.0 mg (80%), 95% purity, White solid.

^{1}H NMR (300 MHz, $CDCl_3$) δ 7.01 (d, *J* = 8.8 Hz, 2H), 6.47 (d, *J* = 8.9 Hz, 2H), 3.87 (d, *J* = 4.7 Hz, 1H), 2.52 – 2.42 (m, 1H), 2.14 – 2.06 (m, 1H), 1.72 – 1.12 (m, 10H), 0.97 (d, *J* = 6.8 Hz, 3H), 0.81 (d, *J* = 6.9 Hz, 3H).
^{13}C NMR (75 MHz, $CDCl_3$) δ 213.9, 146.7, 129.5, 129.1, 122.4, 114.8, 67.2, 48.2, 30.5, 29.3, 27.7, 25.9, 25.7, 25.3, 20.3, 17.5.
HRMS(ESI-TOF) m/z: calcd for $[M^+]H^+$ $C_{17}H_{24}ClNO$ 294.1619, found: 294.1619.

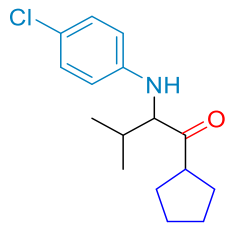

4af

2-((4-chlorophenyl)amino)-1-cyclopentyl-3-methylbutan-1-one (4af)

Chromatography Pentane/EA = 50:1 (v/v), 39.1 mg (70%), 95% purity, White solid.

^{1}H NMR (300 MHz, $CDCl_3$) δ 7.02 (d, *J* = 8.9 Hz, 2H), 6.46 (d, *J* = 8.9 Hz, 2H), 3.80 (d, *J* = 5.1 Hz, 1H), 3.02 – 2.91 (m, 1H), 2.16 – 2.06 (m, 1H), 1.79 – 1.21 (m, 8H), 0.96 (d, *J* = 6.8 Hz, 3H), 0.85 (d, *J* = 6.9 Hz, 3H).
^{13}C NMR (75 MHz, $CDCl_3$) δ 214.6, 146.6, 134.3, 129.5, 129.1, 122.4, 114.7, 68.4, 48.5, 38.9, 30.7, 30.5, 29.3, 26.1, 20.2, 17.8.
HRMS(ESI-TOF) m/z: calcd for $[M^+]H^+$ $C_{16}H_{22}ClNO$ 280.1463, found: 280.1462.

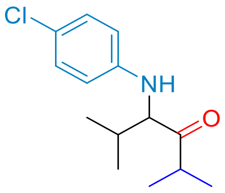

4ag

4-((4-chlorophenyl)amino)-2,5-dimethylhexan-3-one (4ag)

Chromatography Pentane/EA = 50:1 (v/v), 48.2 mg (95%), White solid.

^{1}H NMR (400 MHz, $CDCl_3$) δ 7.02 (d, *J* = 8.9 Hz, 2H), 6.48 (d, *J* = 8.9 Hz, 2H), 3.88 (d, *J* = 4.9 Hz, 1H), 2.78 – 2.71 (m, 1H), 2.14 – 2.06 (m, 1H), 0.98 – 0.96 (m, 9H), 0.83 (d, *J* = 6.8 Hz, 3H).
^{13}C NMR (101 MHz, $CDCl_3$) δ 214.9, 146.6, 129.1, 122.4, 114.8, 67.1, 38.0, 30.5, 20.2, 19.0, 17.7, 17.6.
HRMS(ESI-TOF) m/z: calcd for $[M^+]H^+$ $C_{14}H_{20}ClNO$ 254.1306, found: 254.1310.

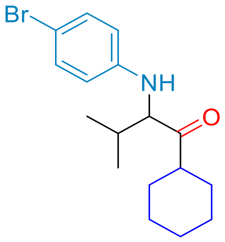

4ah

2-((4-bromophenyl)amino)-1-cyclohexyl-3-methylbutan-1-one (4ah)

Chromatography Pentane/EA = 50:1 (v/v), 64.2 mg (95%), White solid.

^{1}H NMR (300 MHz, $CDCl_3$) δ 7.01 (d, *J* = 9.0 Hz, 2H), 6.47 (d, *J* = 8.9 Hz, 2H), 3.87 (d, *J* = 4.8 Hz, 1H), 2.52 – 2.43 (m, 1H), 2.16 – 2.06 (m, 1H), 1.74 – 1.09 (m, 10H), 0.97 (d, *J* = 6.8 Hz, 3H), 0.81 (d, *J* = 6.8 Hz, 3H).
^{13}C NMR (75 MHz, $CDCl_3$) δ 214.0, 146.7, 129.1, 122.4, 117.3, 114.8, 113.9, 67.1, 48.3, 30.5, 29.3, 27.7, 25.9, 25.7, 25.3, 20.3, 17.5.
HRMS(ESI-TOF) m/z: calcd for $[M^+]H^+$ $C_{25}H_{24}ClNO$ 390.1619, found: 390.1622.

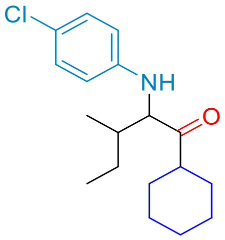

4ai

2-((4-chlorophenyl)amino)-1-cyclohexyl-3-methylpentan-1-one (4ai)

Chromatography Pentane/EA = 50:1 (v/v), 49.2 mg (80%), pale yellow solid.

^{1}H NMR (400 MHz, $CDCl_3$) δ 7.01 (d, *J* = 8.8 Hz, 2H), 6.57 – 6.33 (m, 2H), 4.23 – 4.16 (m, 0.91H), 3.81 (d, *J* = 5.7 Hz, 1H), 3.67 – 3.62 (m, 0.1H), 3.00 – 2.92 (m, 0.9H), 2.82 – 2.77 (m, 0.11H), 1.66 – 0.78 (m, 19H).
^{13}C NMR (101 MHz, $CDCl_3$) δ 214.9, 214.6(minor), 165.9, 146.6(minor), 146.4, 134.2, 129.5, 129.1(minor), 122.4(minor), 122.3, 114.8(minor), 114.5, 67.8, 66.4(minor), 48.5, 48.3(minor), 38.9, 37.2, 36.8(minor), 30.8(minor), 30.7, 29.5(minor), 29.2(minor), 27.1(minor), 26.1, 24.9, 23.9(minor), 22.9, 16.3, 14.2(minor), 14.0, 11.9(minor), 11.6, 11.1(minor).
HRMS(ESI-TOF) m/z: calcd for $[M^+]H^+$ $C_{18}H_{26}ClNO$ 308.1776, found: 308.1779.

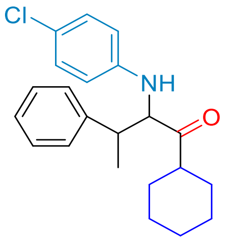

4aj

2-((4-chlorophenyl)amino)-1-cyclohexyl-3-phenylbutan-1-one (4aj)

Chromatography Pentane/EA = 50:1 (v/v), 28.5 mg (40%), 95% purity, White solid.

^{1}H NMR (300 MHz, $CDCl_3$) δ 7.28 – 6.21 (m, 9H), 4.11 (d, J = 6.0 Hz, 0.8H), 4.00 (d, J = 6.0 Hz, 0.2H), 3.24 – 3.11 (m, 1H), 2.89 – 2.85 (m, 0.2H), 2.73 – 2.67 (m, 0.8H), 1.71 – 0.79 (m, 13H).
^{13}C NMR (75 MHz, $CDCl_3$) δ 216.1(minor), 213.6, 145.8(minor), 142.8, 141.5(minor), 129.5(minor), 129.1, 128.9(minor), 128.8, 128.7(minor), 127.8, 127.7(minor), 127.3(minor), 127.0, 122.6, 114.9, 114.5(minor), 68.9, 68.6(minor), 49.5, 47.7(minor), 42.3, 41.3(minor), 30.9, 30.7(minor), 30.3, 29.7(minor), 28.6, 26.2, 26.1(minor), 26.0, 22.9, 18.1(minor), 16.1, 14.0(minor).
HRMS(ESI-TOF) m/z: calcd for $[M^{+}]H^{+}$ $C_{22}H_{26}ClNO$ 356.1776, found: 356.1773.

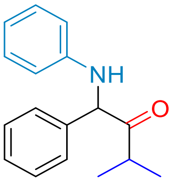

4ak

3-methyl-1-phenyl-1-(phenylamino)butan-2-one (4ak)

Chromatography Pentane/EA = 50:1 (v/v), 40.5 mg (80%), pale yellow solid.

^{1}H NMR (300 MHz, $CDCl_3$) δ 7.38 – 6.48 (m, 10H), 5.05 (s, 1H), 2.83 – 2.69 (m, 1H), 1.08 (d, J = 7.0 Hz, 3H), 0.72 (d, J = 6.7 Hz, 3H).
^{13}C NMR (75 MHz, $CDCl_3$) δ 210.1, 145.8, 137.8, 129.1, 129.1, 128.3, 128.1, 117.7, 113.4, 66.3, 37.0, 19.5, 18.3.
HRMS(ESI-TOF) m/z: calcd for $[M^{+}]H^{+}$ $C_{17}H_{19}NO$ 254.1539, found: 254.2536.

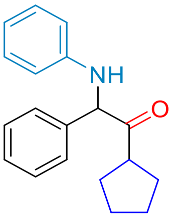

4al

1-cyclopentyl-2-phenyl-2-(phenylamino)ethan-1-one (4al)

Chromatography Pentane/EA = 50:1 (v/v), 41.9 mg (75%), pale yellow solid.

^{1}H NMR (300 MHz, $CDCl_3$) δ 7.40 – 6.46 (m, 10H), 5.00 (s, 1H), 2.95 – 2.89 (m, 1H), 1.93 – 0.76 (m, 8H).
^{13}C NMR (75 MHz, $CDCl_3$) δ 209.2, 146.1, 138.0, 129.1, 129.1, 128.2, 128.1, 117.5, 113.3, 67.3, 47.8, 31.1, 29.5, 26.2, 26.1.
HRMS(ESI-TOF) m/z: calcd for $[M^{+}]H^{+}$ $C_{19}H_{21}NO$ 280.1696, found: 280.1702.

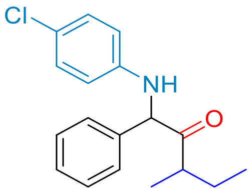

4am

1-((4-chlorophenyl)amino)-3-methyl-1-phenylpentan-2-one (4am)

Chromatography Pentane/EA = 50:1 (v/v), 45.2 mg (75%), 95% purity, dr = 4:1, slightly sticky White solid.

^{1}H NMR (300 MHz, $CDCl_3$) δ 7.36 – 7.21 (m, 5H), 6.94 (d, *J* = 8.8 Hz, 2H), 6.39 (d, *J* = 8.8 Hz, 2H), 4.96 (d, *J* = 17.2 Hz, 1H), 2.68 – 2.54 (m, 1H), 1.70 – 1.56 (m, 1H), 1.44 – 0.27 (m, 7H).
^{13}C NMR (75 MHz, $CDCl_3$) δ 208.9, 208.8(minor), 144.4, 137.1, 136.7(minor), 129.5(minor), 129.2, 129.1(minor), 128.9, 128.5, 128.5(minor), 128.2, 128.1(minor), 114.4, 67.4, 66.3(minor), 44.0, 44.0(minor), 27.2, 25.5(minor), 17.8, 16.2(minor), 11.6, 11.1(minor).
HRMS(ESI-TOF) m/z: calcd for $[M^+]H^+$ $C_{18}H_{20}ClNO$ 302.1306, found: 302.1308.

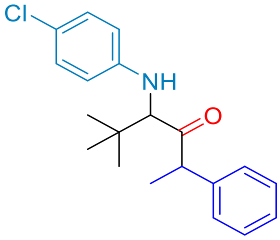

4an

4-((4-chlorophenyl)amino)-5,5-dimethyl-2-phenylhexan-3-one (4an)

Chromatography Pentane/EA = 50:1 (v/v), 34.9 mg (50%), White solid.

^{1}H NMR (400 MHz, $CDCl_3$) δ 7.26 – 6.92 (m, 7H), 6.48 (d, *J* = 8.8 Hz, 2H), 2.87 – 2.86 (m, 1H), 2.79 (s, 1H), 1.21 (d, *J* = 6.8 Hz, 3H), 0.91 (s, 9H).
^{13}C NMR (101 MHz, $CDCl_3$) δ 212.5, 146.5, 145.8, 129.5, 128.9, 128.3, 127.8, 127.8, 127.6, 126.0, 125.7, 113.9, 113.9, 56.3, 56.2, 47.2, 46.5, 31.9, 27.6, 21.0, 17.9.
HRMS(ESI-TOF) m/z: calcd for $[M^+]H^+$ $C_{20}H_{25}ClNO$ 330.1619, found: 330.1624.

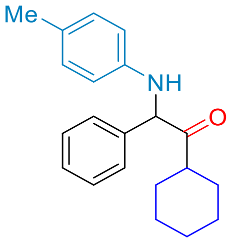

4ao

1-cyclohexyl-2-phenyl-2-(p-tolylamino)ethan-1-one (4ao)

Chromatography Pentane/EA = 50:1 (v/v), 30.7 mg (50%), White solid.

^{1}H NMR (300 MHz, $CDCl_3$) δ 7.37 – 7.19 (m, 5H), 6.82 (d, *J* = 7.9 Hz, 2H), 6.42 (d, *J* = 8.5 Hz, 2H), 5.02 (s, 1H), 2.52 – 2.42 (m, 1H), 2.10 (s, 3H), 1.86 – 0.76 (m, 10H).
^{13}C NMR (101 MHz, $CDCl_3$) δ 209.28, 137.85, 129.62, 129.03, 128.20, 128.07, 126.72, 119.60, 113.44, 66.50, 47.28, 29.65, 28.28, 25.78, 25.56, 25.08, 20.32.
HRMS(ESI-TOF) m/z: calcd for $[M^+]H^+$ $C_{21}H_{25}NO$ 308.2009, found: 308.2002.

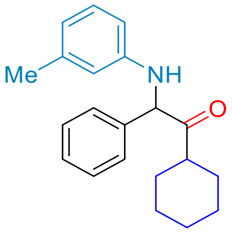

4ap

1-cyclohexyl-2-phenyl-2-(m-tolylamino)ethan-1-one (4ap)

Chromatography Pentane/EA = 50:1 (v/v), 38.1 mg (62%), White solid.

^{1}H NMR (300 MHz, $CDCl_3$) δ 7.37 – 6.27 (m, 9H), 5.02 (s, 1H), 2.52 – 2.42 (m, 1H), 2.13 (s, 3H), 1.86 – 0.95 (m, 10H).
^{13}C NMR (75 MHz, $CDCl_3$) δ 209.1, 138.9, 129.0, 129.0, 128.2, 128.1, 118.6, 114.4, 110.4, 66.2, 47.3, 29.7, 28.3, 25.8, 25.6, 25.1, 21.5.
HRMS(ESI-TOF) m/z: calcd for $[M^{+}]H^{+}$ $C_{21}H_{25}NO$ 308.2009, found: 308.2017.

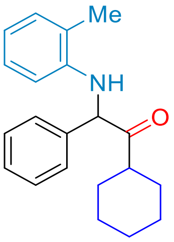

4aq

1-cyclohexyl-2-phenyl-2-(o-tolylamino)ethan-1-one (4aq)

Chromatography Pentane/EA = 50:1 (v/v), 24.5 mg (40%), White solid.

^{1}H NMR (300 MHz, $CDCl_3$) δ 7.36 – 6.41 (m, 9H), 5.03 (s, 1H), 2.51 – 2.42 (m, 1H), 2.09 (s, 3H), 1.86 – 1.00 (m, 10H).
^{13}C NMR (75 MHz, $CDCl_3$) δ 209.1, 137.5, 129.6, 129.5, 129.0, 128.3, 128.2, 127.2, 124.7, 123.9, 113.9, 66.7, 47.3, 29.6, 28.3, 25.8, 25.6, 25.1, 20.3.
HRMS(ESI-TOF) m/z: calcd for $[M^{+}]H^{+}$ $C_{21}H_{25}NO$ 308.2009, found: 308.2011.

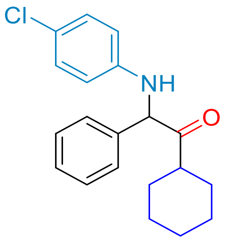

4ar

2-((4-chlorophenyl)amino)-1-cyclohexyl-2-phenylethan-1-one (4ar)

Chromatography Pentane/EA = 50:1 (v/v), 53.1 mg (81%), 95% purity, White solid.

^{1}H NMR (300 MHz, $CDCl_3$) δ 7.35 – 7.18 (m, 5H), 6.93 (d, *J* = 8.8 Hz, 2H), 6.38 (d, *J* = 8.8 Hz, 2H), 4.98 (s, 1H), 2.49 – 2.42 (m, 1H), 1.87 – 0.95 (m, 10H).
^{13}C NMR (75 MHz, $CDCl_3$) δ 208.7, 144.4, 137.2, 129.1, 128.9, 128.4, 127.9, 122.1, 114.3, 66.0, 47.2, 29.6, 28.3, 25.7, 25.5, 25.0.
HRMS(ESI-TOF) m/z: calcd for $[M^{+}]H^{+}$ $C_{20}H_{22}ClNO$ 328.1463, found: 328.1469.

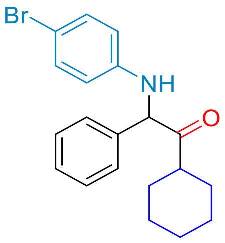

4as

2-((4-bromophenyl)amino)-1-cyclohexyl-2-phenylethan-1-one **(4as)**

Chromatography Pentane/EA = 50:1 (v/v), 29.4 mg (79%), White solid.

^{1}H NMR (300 MHz, $CDCl_3$) δ 7.35 – 7.22 (m, 5H), 6.94 (d, *J* = 8.8 Hz, 2H), 6.39 (d, *J* = 8.9 Hz, 2H), 4.98 (s, 1H), 2.51 – 2.41 (m, 1H), 1.86 – 0.90 (m, 10H).
^{13}C NMR (75 MHz, $CDCl_3$) δ 208.7, 144.4, 137.2, 129.2, 128.9, 128.4, 128.0, 122.1, 114.4, 66.0, 47.2, 29.6, 28.3, 25.7, 25.5, 25.0.
HRMS(ESI-TOF) m/z: calcd for [M$^+$]H$^+$ $C_{20}H_{22}BrNO$ 372.0958, found: 372.0955.

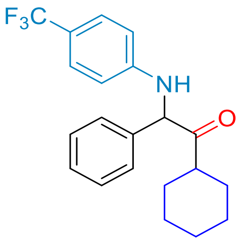

4at

1-cyclohexyl-2-phenyl-2-((4-(trifluoromethyl)phenyl)amino)ethan-1-one **(4at)**

Chromatography Pentane/EA = 50:1 (v/v), 36.1 mg (50%), White solid.

^{1}H NMR (300 MHz, $CDCl_3$) δ 7.35 – 7.19 (m, 7H), 6.49 (d, *J* = 8.3 Hz, 2H), 5.05 (s, 1H), 2.53 – 2.43 (m, 1H), 1.88 – 0.88 (m, 10H).
^{13}C NMR (75 MHz, $CDCl_3$) δ 208.3, 148.2, 136.8, 129.3, 128.6, 127.9, 126.5, 126.5, 112.6, 65.5, 47.2, 29.7, 28.3, 25.7, 25.5, 25.0.
^{19}F NMR (282 MHz, $CDCl_3$) δ -61.14 (s, 3F).
HRMS(ESI-TOF) m/z: calcd for [M$^+$]H$^+$ $C_{21}H_{22}F_3NO$ 362.1726, found: 362.1736.

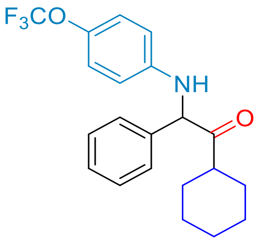

4au

1-cyclohexyl-2-phenyl-2-((4-(trifluoromethoxy)phenyl)amino)ethan-1-one **(4au)**

Chromatography Pentane/EA = 50:1 (v/v), 56.6 mg (75%), White solid.

^{1}H NMR (300 MHz, $CDCl_3$) δ 7.37 – 6.39 (m, 9H), 4.97 (s, 1H), 2.51 – 2.41 (m, 1H), 1.86 – 0.90 (m, 10H).
^{13}C NMR (75 MHz, $CDCl_3$) δ 208.7, 144.7, 140.5, 137.3, 129.2, 128.5, 128.0, 122.3, 113.5, 66.2, 47.2, 29.7, 28.3, 25.7, 25.5, 25.0.
^{19}F NMR (376 MHz, $CDCl_3$) δ -58.47 (m, 3F).
HRMS(ESI-TOF) m/z: calcd for [M$^+$]H$^+$ $C_{21}H_{22}F_3NO_2$ 378.1675, found: 378.1677.

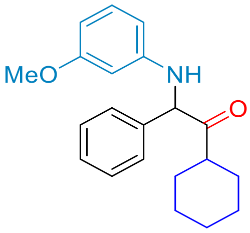

4av

1-cyclohexyl-2-((3-methoxyphenyl)amino)-2-phenylethan-1-one (4av)

Chromatography Pentane/EA = 50:1 (v/v), 49.8 mg (77%), pale yellow solid.

^{1}H NMR (300 MHz, $CDCl_3$) δ 7.37 – 7.18 (m, 5H), 6.91 (t, J = 8.1 Hz, 1H), 6.15 – 6.02 (m, 3H), 5.02 (s, 1H), 3.62 (s, 3H), 2.51 – 2.42 (m, 1H), 1.86 – 0.92 (m, 10H).
^{13}C NMR (75 MHz, $CDCl_3$) δ 208.9, 160.6, 147.4, 137.7, 129.9, 129.1, 128.3, 128.0, 106.4, 102.8, 99.2, 66.1, 54.9, 47.2, 29.7, 28.3, 25.8, 25.5, 25.1.
HRMS(ESI-TOF) m/z: calcd for $[M^{+}]H^{+}$ $C_{21}H_{25}NO_2$ 324.1958, found: 324.1963.

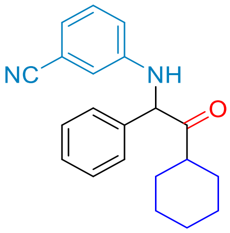

4aw

3-((2-cyclohexyl-2-oxo-1-phenylethyl)amino)benzonitrile (4aw)

Chromatography Pentane/EA = 50:1 (v/v), 50.9 mg (80%), White solid.

^{1}H NMR (300 MHz, $CDCl_3$) δ 7.36 – 6.45 (m, 9H), 5.01 (s, 1H), 2.51 – 2.41 (m, 1H), 1.88 – 0.91 (m, 10H).
^{13}C NMR (75 MHz, $CDCl_3$) δ 208.7, 145.7, 137.3, 129.1, 129.1, 128.4, 127.9, 122.6, 121.5, 116.5, 114.2, 84.1, 65.8, 47.2, 29.6, 28.3, 25.7, 25.5, 25.0.
HRMS(ESI-TOF) m/z: calcd for $[M^{+}]H^{+}$ $C_{21}H_{22}N_2O$ 319.1805, found: 319.1808.

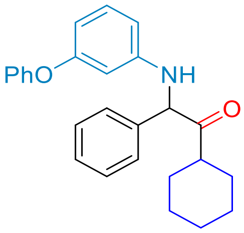

4ax

1-cyclohexyl-2-((3-phenoxyphenyl)amino)-2-phenylethan-1-one (4ax)

Chromatography Pentane/EA = 50:1 (v/v), 51.6 mg (67%), pale yellow solid.

^{1}H NMR (400 MHz, $CDCl_3$) δ 7.26 – 6.08 (m, 14H), 4.96 (s, 1H), 2.45 – 2.38 (m, 1H), 1.83 – 0.87 (m, 10H).
^{13}C NMR (101 MHz, $CDCl_3$) δ 208.8, 158.4, 156.8, 147.4, 137.3, 130.1, 129.6, 129.1, 128.3, 128.0, 123.1, 119.2, 108.5, 107.6, 103.3, 65.9, 47.2, 29.6, 28.2, 25.7, 25.5, 25.0.
HRMS(ESI-TOF) m/z: calcd for $[M^{+}]H^{+}$ $C_{26}H_{27}NO_2$ 386.2115, found: 386.2122.

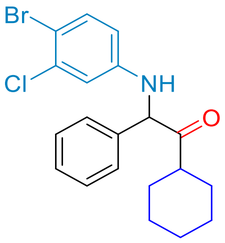

4ay

2-((4-bromo-3-chlorophenyl)amino)-1-cyclohexyl-2-phenylethan-1-one (4ay)

Chromatography Pentane/EA = 50:1 (v/v), 56.9 mg (70%), pale yellow solid.

^{1}H NMR (300 MHz, $CDCl_3$) δ 7.33 – 7.20 (m, 5H), 7.15 (d, *J* = 8.7 Hz, 1H), 6.55 (d, *J* = 2.7 Hz, 1H), 6.26 – 6.22 (m, 1H), 4.96 (s, 1H), 2.49 – 2.39 (m, 1H), 1.87 – 0.88 (m, 10H).
^{13}C NMR (75 MHz, $CDCl_3$) δ 208.3, 145.9, 136.6, 134.5, 133.6, 129.3, 128.6, 127.9, 114.5, 113.4, 108.6, 65.6, 47.2, 29.6, 28.3, 25.7, 25.5, 25.0.
HRMS(ESI-TOF) m/z: calcd for $[M^{+}]H^{+}$ $C_{20}H_{21}BrNO$ 406.0568, found: 406.0567.

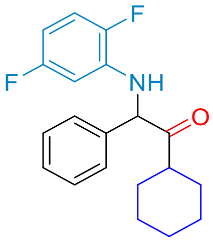

4az

1-cyclohexyl-2-((2,5-difluorophenyl)amino)-2-phenylethan-1-one (4az)

Chromatography Pentane/EA = 50:1 (v/v), 34.9 mg (53%), 95% purity, White solid.

^{1}H NMR (300 MHz, $CDCl_3$) δ 7.35 – 6.24 (m, 8H), 5.00 (s, 1H), 2.51 – 2.41 (m, 1H), 1.86 – 0.95 (m, 10H).
^{13}C NMR (75 MHz, $CDCl_3$) δ 208.4, 137.1, 129.2, 128.5, 127.9, 112.9, 112.8, 112.8, 112.7, 110.5, 110.4, 110.2, 110.2, 103.9, 103.6, 103.5, 103.2, 66.1, 47.3, 29.6, 28.3, 25.7, 25.5, 25.0.
^{19}F NMR (282 MHz, $CDCl_3$) δ -125.62 (s, 1F), -130.94 (s, 1F).
HRMS(ESI-TOF) m/z: calcd for $[M^{+}]H^{+}$ $C_{20}H_{21}F_2NO$ 330.1664, found: 330.1671.

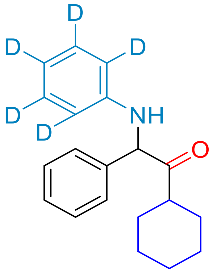

4ba

1-cyclohexyl-2-phenyl-2-((phenyl-d5)amino)ethan-1-one (4ba)

Chromatography Pentane/EA = 50:1 (v/v), 47.7 mg (80%), White solid.

^{1}H NMR (300 MHz, $CDCl_3$) δ 7.38 – 7.17 (m, 5H), 5.02 (s, 1H), 2.52 – 2.43 (m, 1H), 1.87 – 0.78 (m, 10H).
^{13}C NMR (75 MHz, $CDCl_3$) δ 209.1, 145.8, 137.7, 129.5, 129.1, 128.3, 128.0, 66.2, 47.2, 29.7, 28.3, 25.8, 25.5, 25.1.
HRMS(ESI-TOF) m/z: calcd for $[M^{+}]H^{+}$ $C_{20}H_{18}D_5NO$ 299.2166, found: 299.2160.

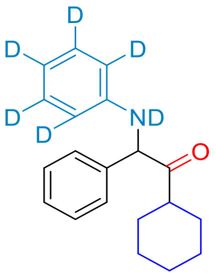

4bb

2 1-cyclohexyl-2-phenyl-2-((phenyl-d5)amino-d)ethan-1-one (4bb)

Chromatography Pentane/EA = 50:1 (v/v), 42.5 mg (60%), White solid.

^{1}H NMR (400 MHz, $CDCl_3$) δ 7.37 – 7.17 (m, 5H), 5.02 (s, 1H), 2.51 – 2.44 (m, 1H), 1.86 – 0.92 (m, 10H).
^{13}C NMR (101 MHz, $CDCl_3$) δ 209.1, 145.9, 137.8, 129.1, 128.2, 128.2, 128.0, 66.2, 47.2, 29.7, 28.3, 25.8, 25.5, 25.1.
HRMS(ESI-TOF) m/z: calcd for $[M^+]H^+$ $C_{20}H_{17}D_5NO$ 299,2166, found: 299.2174.

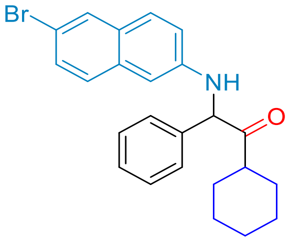

4bc

2-((6-bromonaphthalen-2-yl)amino)-1-cyclohexyl-2-phenylethan-1-one (4bc)

Chromatography Pentane/EA = 50:1 (v/v), 46.4 mg (55%), 95% purity, White solid.

^{1}H NMR (300 MHz, $CDCl_3$) δ 7.68 – 6.87 (m, 10H), 6.53 (d, *J* = 2.4 Hz, 1H), 5.13 (s, 1H), 2.56 – 2.46 (m, 1H), 1.90 – 0.76 (m, 10H).
^{13}C NMR (75 MHz, $CDCl_3$) δ 208.7, 143.7, 137.1, 133.3, 129.5, 129.3, 129.2, 128.5, 128.1, 128.0, 127.6, 119.1, 115.2, 105.4, 66.0, 47.3, 29.7, 28.3, 25.8, 25.5, 25.1.
HRMS(ESI-TOF) m/z: calcd for $[M^+]H^+$ $C_{24}H_{24}BrNO$ 422.1114, found: 422.1119.

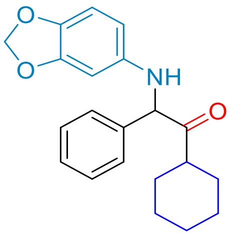

4bd

2-(benzo[d][1,3]dioxol-5-ylamino)-1-cyclohexyl-2-phenylethan-1-one (4bd)

Chromatography Pentane/EA = 50:1 (v/v), 33.7 mg (50%), White solid.

^{1}H NMR (400 MHz, $CDCl_3$) δ 7.35 – 7.19 (m, 5H), 6.67 (d, *J* = 8.6 Hz, 1H), 6.21 (d, *J* = 2.3 Hz, 1H), 6.12 – 7.09 (m, 1H), 4.94 (s, 1H), 2.49 – 2.42 (m, 1H), 2.10 (s, 2H), 1.86 – 0.89 (m, 10H).
^{13}C NMR (101 MHz, $CDCl_3$) δ 208.6, 144.5, 142.9, 137.1, 129.3, 128.9, 128.6, 127.9, 127.6, 109.7, 107.1, 95.7, 66.5, 47.2, 30.9, 29.6, 28.3, 25.7, 25.5, 25.0.
HRMS(ESI-TOF) m/z: calcd for $[M^+]H^+$ $C_{21}H_{23}NO_3$ 338.1751, found: 338.1755.

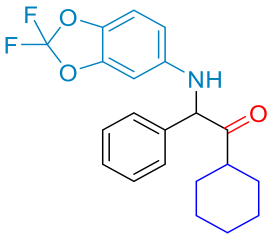

4be

1-cyclohexyl-2-((2,2-difluorobenzo[d][1,3]dioxol-5-yl)amino)-2-phenylethan-1-one (4be)

Chromatography Pentane/EA = 50:1 (v/v), 46.3 mg (62%), Yellow solid.

^{1}H NMR (300 MHz, $CDCl_3$) δ 7.36 – 7.18 (m, 5H), 6.67 (d, *J* = 8.6 Hz, 1H), 6.21 (d, *J* = 2.4 Hz, 1H), 6.13 – 6.09 (m, 1H), 4.91 (s, 1H), 2.96 – 2.85 (m, 1H), 1.92 – 1.07 (m, 10H).
^{13}C NMR (75 MHz, $CDCl_3$) δ 208.8, 144.5, 143.0, 137.3, 135.8, 134.9, 131.6, 129.3, 128.5, 127.9, 109.7, 107.1, 95.7, 67.7, 47.7, 31.1, 29.6, 26.2, 26.1.
^{19}F NMR (282 MHz, $CDCl_3$) δ -50.47 (s, 1H), -50.49 (s, 1H).
HRMS(ESI-TOF) m/z: calcd for $[M^{+}]H^{+}$ $C_{21}H_{21}F_2NO_3$ 374.1562, found: 374.1566.

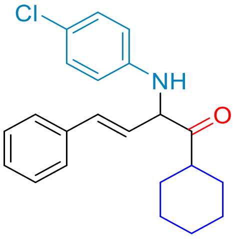

4bf

(E)-2-((4-chlorophenyl)amino)-1-cyclohexyl-4-phenylbut-3-en-1-one (4bf)

Chromatography Pentane/EA = 50:1 (v/v), 37.5 mg (53%), 95% purity, Brown solid.

^{1}H NMR (400 MHz, $CDCl_3$) δ 7.31 – 6.99 (m, 5H), 7.07 – 6.98 (m, 2H), 6.75 (d, *J* = 15.8, 1H), 6.51 – 6.47 (m, 2H), 6.04 – 5.98 (m, 1H), 4.67 (d, *J* = 7.4 Hz, 1H), 2.70 – 2.64 (m, 1H), 1.87 – 1.13 (m, 10H).
^{13}C NMR (101 MHz, $CDCl_3$) δ 208.8, 145.3, 144.9, 134.7, 129.1, 128.7, 128.3, 126.6, 124.9, 114.6, 64.1, 47.6, 29.6, 28.1, 25.8, 25.6, 25.2.
HRMS(ESI-TOF) m/z: calcd for $[M^{+}]H^{+}$ $C_{22}H_{24}ClNO$ 354.1619, found: 354.1621.

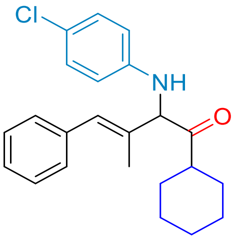

4bg

(E)-2-((4-chlorophenyl)amino)-1-cyclohexyl-3-methyl-4-phenylbut-3-en-1-one (4bg)

Chromatography Pentane/EA = 50:1 (v/v), 44.1 mg (60%), Brown viscous solid.

^{1}H NMR (400 MHz, $CDCl_3$) δ 7.33 – 6.99 (m, 7H), 6.84 (s, 1H), 6.56 – 6.52 (m, 2H), 4.66 (s, 1H), 2.82 – 2.75 (m, 1H) , 1.86 – 1.58 (m, 4H), 1.56 (s, 1H), 1.27 – 1.12 (m, 6H).
^{13}C NMR (101 MHz, $CDCl_3$) δ 209.5, 144.8, 136.8, 134.9, 132.5, 129.0, 128.8, 128.4, 127.2, 122.0, 114.5, 70.2, 46.7, 29.7, 28.3, 25.8, 25.6, 25.2, 13.2.
HRMS(ESI-TOF) m/z: calcd for $[M^{+}]Na^{+}$ $C_{23}H_{26}ClNO$ 368.1776, found: 368.1779.

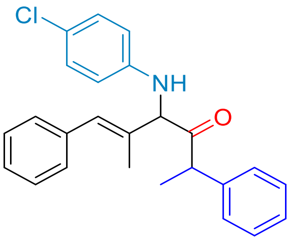

4bh

(E)-4-((4-chlorophenyl)amino)-5-methyl-2,6-diphenylhex-5-en-3-one (4bh)

Chromatography Pentane/EA = 50:1 (v/v), 49.1 mg (63%), 95% purity, Brown viscous solid.

^{1}H NMR (300 MHz, $CDCl_3$) δ 7.29 – 6.86 (m, 15H), 3.86 (d, *J* = 10.9 Hz, 0.53H), 3.79 (d, *J* = 10.9 Hz, 0.52H), 3.50 – 3.39 (m, 1H), 2.08 (s, 1.5H), 1.77 (s, 1.5H), 1.27 (d, *J* = 6.8 Hz, 2H), 0.91 (d, *J* = 7.1 Hz, 1H).
^{13}C NMR (75 MHz, $CDCl_3$) δ 208.3, 207.8(minor), 145.5, 144.2(minor), 144.1(minor), 143.7, 138.2(minor), 137.6(minor), 137.2, 137.1, 129.5(minor), 129.2(minor), 128.8, 128.8, 128.7(minor), 128.5, 128.4(minor), 128.3, 128.3(minor), 127.9, 127.9(minor), 127.6(minor), 127.6, 127.5(minor), 127.4, 126.9, 126.6(minor), 126.4, 126.2(minor), 125.9, 66.7, 66.6(minor), 42.3, 41.9(minor), 30.5(minor), 30.5, 21.1, 20.2(minor).
HRMS(ESI-TOF) m/z: calcd for $[M^{+}]Na^{+}$ $C_{25}H_{24}ClNO$ 412.1439, found: 412.1442.

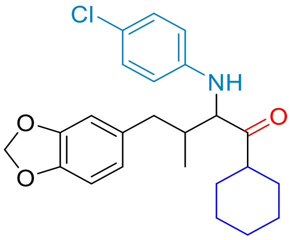

4bi

4-(benzo[d][1,3]dioxol-5-yl)-2-((4-chlorophenyl)amino)-1-cyclohexyl-3-methylbutan-1-one (4bi)

Chromatography Pentane/EA = 50:1 (v/v), 64.5 mg (78%), Brown viscous solid.

^{1}H NMR (300 MHz, $CDCl_3$) δ 7.05 – 6.38 (m, 7H), 5.88 – 5.85 (m, 2H), 4.36 – 4.33 (m, 1H), 4.17 (d, *J* = 5.2 Hz, 0.3H), 3.92 (d, *J* = 5.2 Hz, 0.7H), 2.69 – 2.44 (m, 2H), 2.27 – 1.05 (m, 10H), 0.87 – 0.74 (m, 3H).
^{13}C NMR (75 MHz, $CDCl_3$) δ 213.9, 147.7(minor), 147.6, 146.6(minor), 146.3, 146.0(minor), 145.9, 133.8(minor), 133.5, 129.1, 129.0(minor), 122.8(minor), 122.6, 122.1(minor), 122.0, 115.3(minor), 114.9, 109.4, 109.3(minor), 108.2(minor), 108.1, 100.9(minor), 100.8, 66.3, 63.9(minor), 48.6, 47.9(minor), 40.3(minor), 38.2, 37.9, 36.9(minor), 29.2, 29.2(minor), 27.7, 27.6(minor), 25.9, 25.8(minor), 25.6, 25.6(minor), 25.3, 25.2(minor), 16.8, 14.2(minor).
HRMS(ESI-TOF) m/z: calcd for $[M^{+}]H^{+}$ $C_{24}H_{28}ClNO_3$ 414.1831, found: 414.1823.

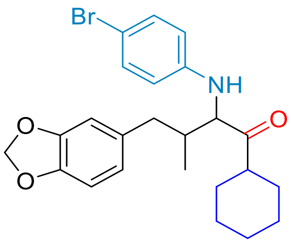

4bj

4-(benzo[d][1,3]dioxol-5-yl)-2-((4-bromophenyl)amino)-1-cyclohexyl-3-methylbutan-1-one (4bj)

Chromatography Pentane/EA = 50:1 (v/v), 64.1 mg (70%), Brown viscous solid.

^1H NMR (300 MHz, $CDCl_3$) δ 7.07 – 6.38 (m, 8H), 5.88 – 5.85 (m, 2H), 3.95 (d, *J* = 5.6 Hz, 0.2H), 3.86 (d, *J* = 5.6 Hz, 0.8H), 3.04 – 2.84 (m, 1H), 2.69 – 2.61 (m, 1H), 2.49 – 2.42 (m, 0.2H), 2.31 – 2.23 (m, 0.8H), 1.76 – 1.18 (m, 11H), 0.88 – 0.76 (m, 3H).
^{13}C NMR (75 MHz, $CDCl_3$) δ 214.4, 214.3(minor), 147.7(minor), 147.6, 146.5(minor), 146.2, 146.0(minor), 145.9, 133.7(minor), 133.4, 129.1, 129.1(minor), 122.8(minor), 122.6, 122.1(minor), 122.1, 115.2(minor), 114.7, 109.4, 109.3(minor), 108.2(minor), 108.2, 100.9(minor), 100.9, 67.5, 65.0(minor), 48.7, 48.3(minor), 40.2(minor), 38.4, 37.9, 36.8(minor), 30.7, 30.6(minor), 30.2, 30.1(minor), 29.7(minor), 29.4, 28.9(minor), 28.9, 26.2, 26.1(minor), 16.8, 14.3(minor).
HRMS(ESI-TOF) m/z: calcd for $[M^+]H^+$ $C_{24}H_{29}NO_3$ 380.2220, found: 380.2230.

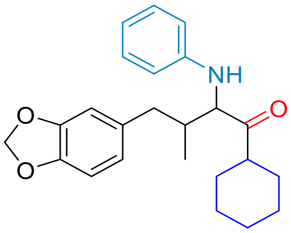

4bk

4-(benzo[d][1,3]dioxol-5-yl)-1-cyclohexyl-3-methyl-2-(phenylamino)butan-1-one **(4bk)**

Chromatography Pentane/EA = 50:1 (v/v), 47.8mg (63%), Brown viscous solid.

^1H NMR (300 MHz, $CDCl_3$) δ 7.12 – 6.48 (m, 8H), 5.86 (d, *J* = 6.2 Hz, 2H), 4.07 (d, *J* = 3.4 Hz, 0.3H), 3.99 (d, *J* = 5.3 Hz, 0.7H), 2.74 – 2.63 (m, 1H), 2.55 – 2.38 (m, 1H), 2.28 – 2.07 (m, 2H), 1.72 – 1.13 (m, 10H), 0.88 – 0.74 (m, 3H).
^{13}C NMR (75 MHz, $CDCl_3$) δ 214.2, 214.1(minor), 147.9(minor), 147.7, 147.6, 147.5(minor), 145.9(minor), 145.9, 134.0(minor), 133.7, 129.3, 129.2(minor), 122.2(minor), 122.0, 118.2(minor), 118.1, 114.1(minor), 113.8, 109.4, 109.4(minor), 108.2(minor), 108.1, 100.8(minor), 100.8, 66.2, 63.9(minor), 48.6, 47.9(minor), 40.4(minor), 38.3, 38.0, 37.0(minor), 29.2, 29.1, 27.8, 27.7(minor), 25.9, 25.8(minor), 25.7, 25.6(minor), 25.4, 25.3(minor), 16.8, 14.2(minor).
HRMS(ESI-TOF) m/z: calcd for $[M^+]Na^+$ $C_{24}H_{29}NO_3$ 402.2040, found: 402.2045.

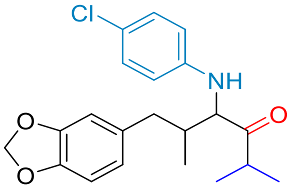

4bl

6-(benzo[d][1,3]dioxol-5-yl)-4-((4-chlorophenyl)amino)-2,5-dimethylhexan-3-one **(4bl)**

Chromatography Pentane/EA = 50:1 (v/v), 56.1 mg (75%), Brown viscous solid.

^1H NMR (300 MHz, $CDCl_3$) δ 7.06 – 6.42 (m, 7H), 5.87 (d, *J* = 6.7 Hz, 2H), 4.02 (d, *J* = 3.2 Hz, 0.2H), 3.94 (d, *J* = 5.4 Hz, 0.8H), 2.81 – 2.59 (m, 2H), 2.29 – 2.07 (m, 2H), 1.30 – 1.18 (m, 2H), 1.00 – 0.85 (m, 7H).
^{13}C NMR (75 MHz, $CDCl_3$) δ 214.6, 214.0(minor), 147.7, 147.6(minor), 146.0, 145.9(minor), 133.4, 133.3(minor), 129.2, 129.1(minor), 124.8, 124.5(minor), 122.9, 122.1(minor), 115.5, 115.1(minor), 109.4, 109.3(minor), 108.2, 108.2(minor), 100.9, 100.9(minor), 66.4, 63.9(minor), 40.1, 38.4, 38.3(minor), 38.0, 37.7, 36.9(minor), 29.7, 27.0(minor), 18.9, 17.7(minor), 16.8, 14.21(minor).
HRMS(ESI-TOF) m/z: calcd for $[M^+]H^+$ $C_{21}H_{24}ClNO_3$ 374.1518, found: 374.1521.

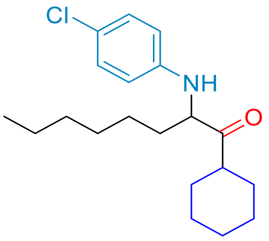

4bm

2-((4-chlorophenyl)amino)-1-cyclohexyloctan-1-one **(4bm)**

Chromatography Pentane/EA = 50:1 (v/v), 48.8 mg (67%), pale yellow viscous solid.

^{1}H NMR (400 MHz, $CDCl_3$) δ 7.02 (d, *J* = 8.8 Hz, 2H), 6.43 (d, *J* = 8.8 Hz, 2H), 4.09 – 4.03 (m, 1H), 2.56 – 2.49 (m, 1H), 1.75 – 0.77 (m, 23H).
^{13}C NMR (101 MHz, $CDCl_3$) δ 213.9, 145.7, 129.1, 122.3, 114.4, 61.5, 47.3, 31.6, 31.4, 30.2, 29.6, 29.2, 27.9, 25.9, 25.7, 25.3, 25.2, 22.5, 14.0.
HRMS(ESI-TOF) m/z: calcd for $[M^{+}]H^{+}$ $C_{20}H_{30}ClNO$ 334.1932, found: 334.1938.

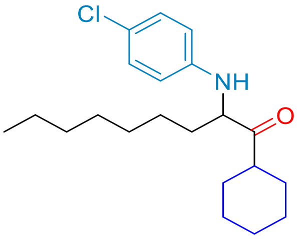

4bn

2-((4-chlorophenyl)amino)-1-cyclohexylnonan-1-one **(4bn)**

Chromatography Pentane/EA = 50:1 (v/v), 36.2 mg (54%), White viscous solid. dr = 9:1.

^{1}H NMR (400 MHz, $CDCl_3$) δ 7.02 (d, *J* = 8.8 Hz, 2H), 6.43 (d, *J* = 8.9 Hz, 2H), 4.09 – 4.03 (m, 1H), 2.55 – 2.48 (m, 1H), 2.33 – 2.19 (m, 1H), 1.75 – 0.77 (m, 24H).
^{13}C NMR (101 MHz, $CDCl_3$) δ 213.9, 145.7, 129.5, 129.1, 129.1, 114.8, 114.4, 61.5, 47.3, 31.7, 31.5, 29.6, 29.5, 29.0, 27.9, 25.9, 25.7, 25.3, 25.2, 22.6, 14.0.
HRMS(ESI-TOF) m/z: calcd for $[M^{+}]Na^{+}$ $C_{21}H_{32}ClNO$ 372.2065, found: 372.2068.

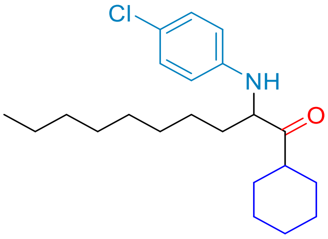

4bo

2-((4-chlorophenyl)amino)-1-cyclohexyldecan-1-one (4bo)

Chromatography Pentane/EA = 50:1 (v/v), 42.6 mg (50%), White viscous solid.

^{1}H NMR (300 MHz, $CDCl_3$) δ 7.02 (d, *J* = 9.0 Hz, 2H), 6.43 (d, *J* = 9.0 Hz, 2H), 4.06 – 4.03 (m, 1H), 2.79 – 2.71 (m, 1H), 2.56 – 2.47 (m, 1H), 1.76 – 0.77 (m, 26H).
^{13}C NMR (75 MHz, $CDCl_3$) δ 213.9, 145.7, 129.1, 122.2, 114.4, 61.5, 47.3, 31. 8, 31.5, 29.6, 29.5, 29.3, 29.1, 27.9, 25.9, 25.7, 25.3, 25.2, 22.6, 14.1.
HRMS(ESI-TOF) m/z: calcd for $[M^{+}]H^{+}$ $C_{22}H_{34}ClNO$ 364.2402, found: 364.2407.

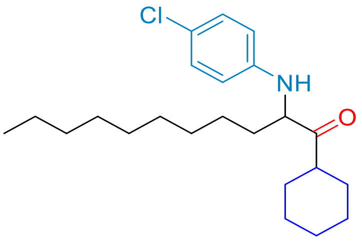

4bp

2-((4-chlorophenyl)amino)-1-cyclohexylundecan-1-one (4bp)

Chromatography Pentane/EA = 50:1 (v/v), 46.2 mg (69%), White viscous solid.

^{1}H NMR (400 MHz, $CDCl_3$) δ 7.02 (d, *J* = 8.8 Hz, 2H), 6.43 (d, *J* = 8.8 Hz, 2H), 4.33 – 4.04 (m, 2H), 2.56 – 2.49 (m, 1H), 1.76 – 0.80 (m, 28H).
^{13}C NMR (101 MHz, $CDCl_3$) δ 213.9, 165.9, 145.7, 129.5, 129.1, 122.2, 114.4, 67.8, 61.4, 47.3, 38.9, 31.8, 31.5, 30.6, 29.6, 29.2, 27.9, 25.9, 25.7, 25.3, 23.9, 22.6, 14.1, 11.1.
HRMS(ESI-TOF) m/z: calcd for $[M^{+}]H^{+}$ $C_{23}H_{36}ClNO$ 378.2558, found: 378.2562.

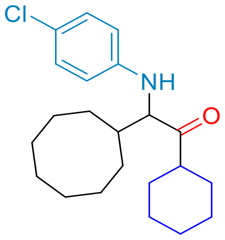

4bq

2-((4-chlorophenyl)amino)-1-cyclohexyl-2-cyclooctylethan-1-one **(4bq)**

Chromatography Pentane/EA = 50:1 (v/v), 40.0 mg (50%), 95% purity, White solid.

^{1}H NMR (300 MHz, $CDCl_3$) δ 7.08 – 6.95 (m, 2H), 6.46 – 6.29 (m, 2H), 4.20 – 3.83 (m, 1H), 3.25 – 2.84 (m, 1H), 2.51 – 1.08 (m, 25H).
^{13}C NMR (75 MHz, $CDCl_3$) δ 214.14, 146.52, 129.06, 123.95, 122.29, 114.69, 114.02, 67.71, 61.22, 48.36, 41.94, 40.51, 39.56, 31.78, 29.35, 27.85, 27.45, 26.83, 26.63, 26.07, 25.66, 25.07.
HRMS(ESI-TOF) m/z: calcd for $[M^{+}]H^{+}$ $C_{22}H_{32}ClNO$ 362.2245, found: 362.2252.

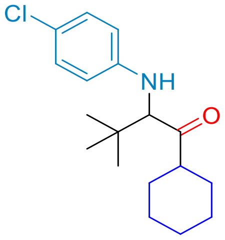

4br

2-((4-chlorophenyl)amino)-1-cyclohexyl-3,3-dimethylbutan-1-one **(4br)**

Chromatography Pentane/EA = 50:1 (v/v), 52.9 mg (86%), 95% purity, White solid.

^{1}H NMR (400 MHz, $CDCl_3$) δ 7.01 (d, *J* = 8.3 Hz, 2H), 6.48 (d, *J* = 8.8 Hz, 2H), 3.75 (s, 1H), 3.01 – 2.94 (m, 1H), 1.70 – 1.23 (m, 10H), 0.96 (s, 9H).
^{13}C NMR (101 MHz, $CDCl_3$) δ 216.2, 165.9, 146.6, 134.2, 129.5, 129.1, 122.5, 115.0, 70.8, 67.7, 51.3, 38.9, 35.3, 30.9, 30.5, 30.5, 28.9, 28.3, 27.6, 26.9, 26.1, 26.0, 23.9, 22.9, 14.0, 11.1.
HRMS(ESI-TOF) m/z: calcd for $[M^{+}]H^{+}$ $C_{18}H_{26}ClNO$ 308.1776, found: 308.1779.

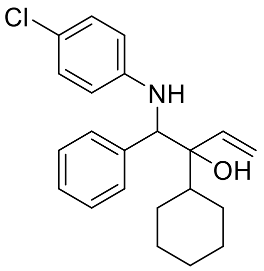

7

1-((4-chlorophenyl)amino)-2-cyclohexyl-1-phenylbut-3-en-2-ol **(7)**

Chromatography Pentane/EA = 10:1 (v/v), 31.9 mg (90%), Brown viscous solid.

^{1}H NMR (300 MHz, $CDCl_3$) δ 7.84 – 4.98 (m, 13H), 4.42 (s, 0.8H), 4.24 (s, 0.2H), 1.76 – 0.94 (m, 11H).
^{13}C NMR (75 MHz, $CDCl_3$) δ 145.8, 145.7(minor), 143.3, 138.3, 138.0(minor), 133.8, 129.1(minor), 128.9, 128.9(minor), 128.8, 128.5(minor), 128.5, 127.9(minor), 127.7, 127.0(minor), 116.7, 116.0(minor), 114.4, 114.1(minor), 79.8, 61.9, 61.4(minor), 44.2, 42.6(minor), 30.2, 28.9(minor), 26.5, 26.3, 26.2(minor), 26.0, 25.8, 25.7(minor), 25.3(minor), 24.74.
HRMS(ESI-TOF) m/z: calcd for $[M^+]H^+$ $C_{22}H_{26}ClNO$ 356.1776, found: 356.17780.

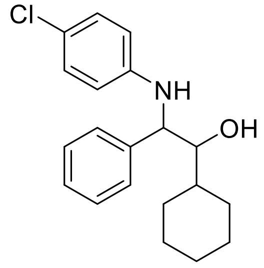

8

2-((4-chlorophenyl)amino)-1-cyclohexyl-2-phenylethan-1-ol **(8)**

Chromatography Pentane/EA = 10:1 (v/v), 26.3 mg (80%), White viscous solid.

^{1}H NMR (300 MHz, $CDCl_3$) δ 7.29 – 6.35 (m, 9H), 4.34 (d, J = 3.0 Hz, 0.6H), 4.30 (d, J = 3.0 Hz, 0.4H), 3.66 – 3.62 (m, 0.4H), 3.58 - 3.55 (m, 0.6H), 2.06 – 0.81 (m, 10H).
^{13}C NMR (75 MHz, $CDCl_3$) δ 145.5, 145.1(minor), 140.9, 138.0(minor), 129.4, 128.9(minor), 128.9, 128.8(minor), 128.5, 128.1(minor), 127.7, 127.5(minor), 126.8, 121.9, 114.6, 114.6(minor), 80.4, 79.1(minor), 60.8, 60.6(minor), 43.1, 42.4(minor), 29.7, 29.6(minor), 29.4, 29.1(minor), 25.6, 25.6(minor), 25.5, 25.4(minor).
HRMS(ESI-TOF) m/z: calcd for $[M^+]H^+$ $C_{20}H_{24}ClNO$ 330.1619, found: 330.1622.

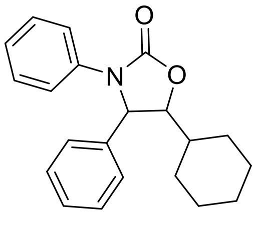

9

3-(4-chlorophenyl)-5-cyclohexyl-4-phenyloxazolidin-2-one **(9)**

Chromatography Pentane/EA = 10:1 (v/v), 27.2 mg (85%), 95% purity, White solid.

^{1}H NMR (400 MHz, $CDCl_3$) δ 7.35 – 7.09 (m, 9H), 4.89 (d, J = 5.4 Hz, 1H), 4.18 (m, 1H), 2.30 – 1.14 (m, 11H).
^{13}C NMR (101 MHz, $CDCl_3$) δ 161.9, 155.4, 138.4, 135.8, 129.7, 129.4, 128.9, 126.4, 121.9, 85.2, 65.5, 43.8, 28.1, 28.1, 25.5, 25.4.
HRMS(ESI-TOF) m/z: calcd for $[M^+]Na^+$ $C_{21}H_{22}ClNO_2$ 378.1231, found: 378.1237.

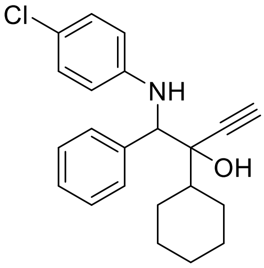

10

1-((4-chlorophenyl)amino)-2-cyclohexyl-1-phenylbut-3-yn-2-ol **(10)**

Chromatography Pentane/EA = 10:1 (v/v), 30.3 mg (86%), 95% purity, Brown viscous solid.

^{1}H NMR (300 MHz, $CDCl_3$) δ 7.37 – 5.59 (m, 9H), 4.49 (s, 0.8H), 4.33 (s, 0.2H), 2.48 (s, 0.8H), 2.45 (s, 0.2H), 2.01 – 0.76 (m, 12H).
^{13}C NMR (75 MHz, $CDCl_3$) δ 145.4(minor), 145.2, 138.1(minor), 137.9, 128.9, 128.9(minor), 128.8, 128.7(minor), 128.4, 128.4(minor), 128.1, 128.1(minor), 122.1, 117.3, 115.4, 115.1, 114.6, 114.6, 83.8(minor), 83.8, 75.5, 74.8(minor), 63.9, 61.5(minor), 46.9, 44.4, 42.9(minor), 29.7, 28.4(minor), 28.3, 27.8(minor), 27.0, 26.2, 26.1(minor), 25.9, 25.7(minor).
HRMS(ESI-TOF) m/z: calcd for $[M^{+}]H^{+}$ $C_{22}H_{24}ClNO$ 354.1619, found: 354.1615.

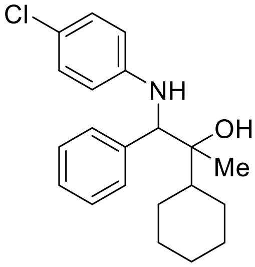

11

1-((4-chlorophenyl)amino)-2-cyclohexyl-1-phenylpropan-2-ol **(11)**

Chromatography Pentane/EA = 10:1 (v/v), 31.2 mg (91%), Brown viscous solid.

^{1}H NMR (300 MHz, $CDCl_3$) δ 7.32 – 7.09 (m, 9H), 4.17 (s, 0.7H), 4.13 (s, 0.7H), 1.75 – 1.32 (m, 11H), 1.18 (s, 2H), 0.90 (s, 1H).
^{13}C NMR (75 MHz, $CDCl_3$) δ 145.9(minor), 145.8, 139.9(minor), 139.6, 128.9, 128.8(minor), 128.3, 128.2(minor), 127.5, 127.4(minor), 126.92, 124.25, 122.2(minor), 121.50, 114.8(minor), 114.30, 64.8(minor), 64.7, 46.6, 45.9(minor), 28.8(minor), 28.7, 27.9, 27.9(minor), 26.7(minor), 26.7, 26.1, 25.9(minor), 25.8, 25.7(minor), 25.1(minor), 21.2(minor), 20.4.
HRMS(ESI-TOF) m/z: calcd for $[M^{+}]H^{+}$ $C_{21}H_{26}ClNO$ 344.1776, found: 344.1770.

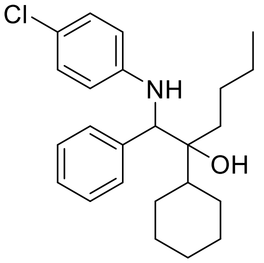

12

1-((4-chlorophenyl)amino)-2-cyclohexyl-1-phenylhexan-2-ol **(12)**

Chromatography Pentane/EA = 10:1 (v/v), 32.7 mg (85%), 95% purity, Brown viscous solid.

^{1}H NMR (300 MHz, $CDCl_3$) δ 7.32 – 7.09 (m, 9H), 4.17 (s, 0.7H), 4.13 (s, 0.7H), 1.75 – 1.32 (m, 11H), 1.18 (s, 2H), 0.90 (s, 1H).

^{13}C NMR (75 MHz, $CDCl_3$) δ 145.9(minor), 145.8, 139.9(minor), 139.6, 128.9, 128.8(minor), 128.3, 128.2(minor), 127.5, 127.4(minor), 126.92, 124.25, 122.2(minor), 121.50, 114.8(minor), 114.30, 64.8(minor), 64.7, 46.6, 45.9(minor), 28.8(minor), 28.7, 27.9, 27.9(minor), 26.7(minor), 26.7, 26.1, 25.9(minor), 25.8, 25.7(minor), 25.1(minor), 21.2(minor), 20.4.
HRMS(ESI-TOF) m/z: calcd for $[M^+]H^+$ $C_{24}H_{32}ClNO$ 386.2245, found: 386.2240.

5. Mechanistic investigation

5.1 Radical trapping experiment by 1,1-diphenylethylene

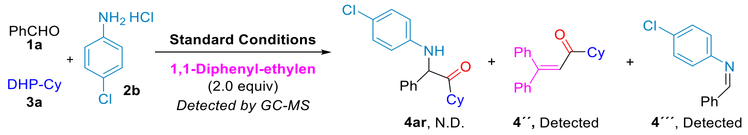

Scheme 1 Radical capture experiments by 1,1-diphenylethylene

A 4 mL screw-cap vial was charged with Hantzsch ester **3a** (40.0 mg, 0.12 mmol, 1.2 equiv), $ArNH_2$.HCl **2b** (0.1 mmol, 17.0 mg, 1.0 equiv), **PC-2** (1.4 mg, 1.5 mol%), and an oven-dried stirring bar. The vial was closed with a Teflon septum and cap and connected to the atmosphere via a needle. After replacing the nitrogen in the vial three times, Aldehyde **1a** (0.1 mmol, 11.0 mg, 1.0 equiv) using a microinjector. Then, $CHCl_3$ (1 mL) was added was added using injector. The vial was then moved to a cannula and transferred into a 300 mL photoautoclave (manufactured by Parr Instrument Company®), under a nitrogen atmosphere. At room temperature, the autoclave was washed with CO three times and charged with 40 bar of CO. The autoclave was placed on a heating plate equipped with a magnetic stirrer and an aluminum block. The reaction mixture was allowed to react at 30 ℃ under UV-A (400-500 nm) for 24 hours. After the reaction was complete, the pressure of the autoclave was carefully released, and the residual CO was washed away with nitrogen. Then, a proper amount of solvent was taken for GC-MS analysis. The result is shown in **Figure 3**. When 1,1-diphenylethylene were added to the reaction, no target product **4ar** was detected, and acyl radicals were trapped by 1,1-diphenylethylene. Data in agreement with that reported previously.[3]

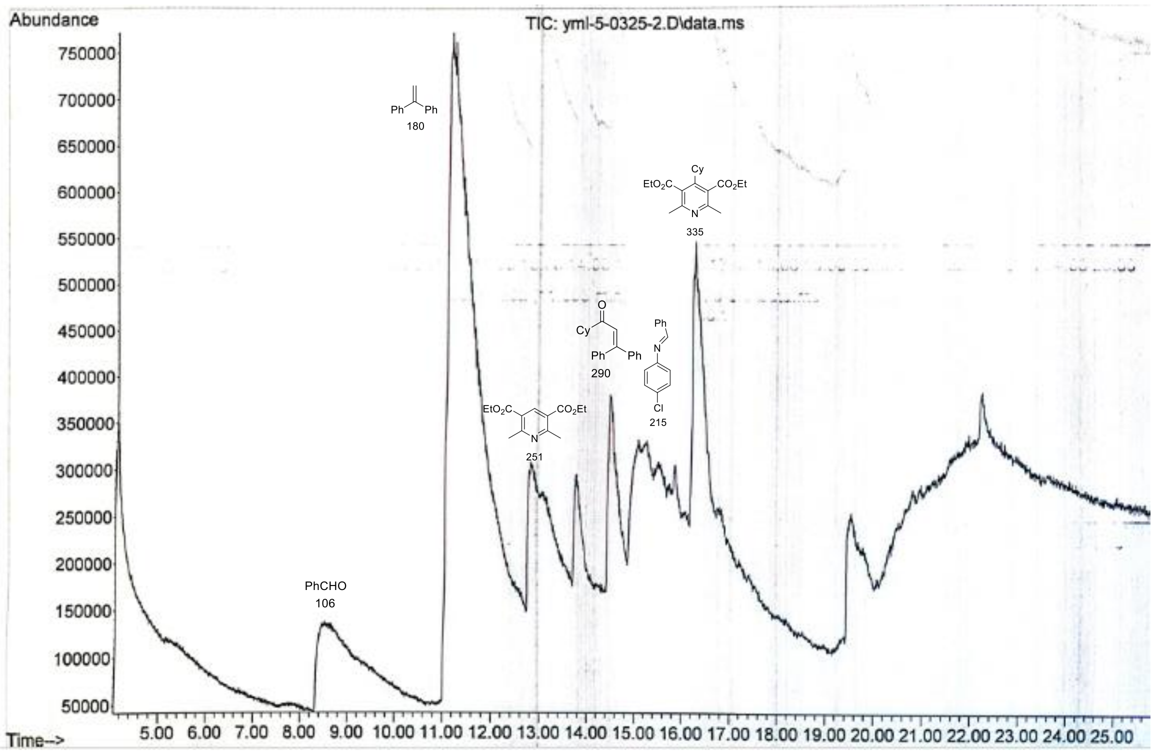

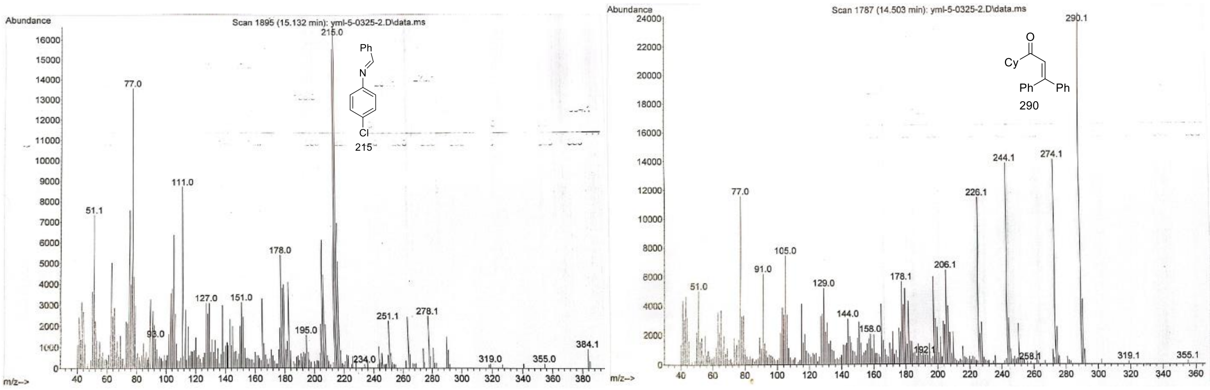

Figure 4 GC-MS of the reaction solvent

5.2 Radical trapping experiment by 2,2,6,6-Tetramethylpiperidinyloxyl (TEMPO)

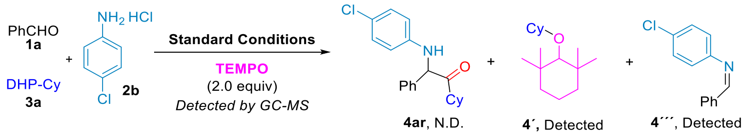

Scheme 2 Radical capture experiments by TEMPO

A 4 mL screw-cap vial was charged with Hantzsch ester **3a** (40.0 mg, 0.12 mmol, 1.2 equiv), $ArNH_2$.HCl **2b** (0.1 mmol, 17.0 mg, 1.0 equiv), **PC-2** (1.4 mg, 1.5 mol%), and an oven-dried stirring bar. The vial was closed with a Teflon septum and cap and connected to the atmosphere via a needle. After replacing the nitrogen in the vial three times, Aldehyde **1a** (0.1 mmol, 11.0 mg, 1.0 equiv) using a microinjector. Then, $CHCl_3$ (1 mL) was added was added using injector. The vial was then moved to a cannula and transferred into a 300 mL photoautoclave (manufactured by Parr Instrument Company®), under a nitrogen atmosphere. At room temperature, the autoclave was washed with CO three times and charged with 40 bar of CO. The autoclave was placed on a heating plate equipped with a magnetic stirrer and an aluminum block. The reaction mixture was allowed to react at 30 ℃ under UV-A (400-500 nm) for 24 hours. After the reaction was complete, the pressure of the autoclave was carefully released, and the residual CO was washed away with nitrogen. Then, a proper amount of solvent was taken for GC-MS analysis. The result is shown in **Figure 4**. When TEMPO were added to the reaction, no target product **4ar** was detected, and alkyl radical was trapped by TEMPO. Data in agreement with that reported previously.[4]

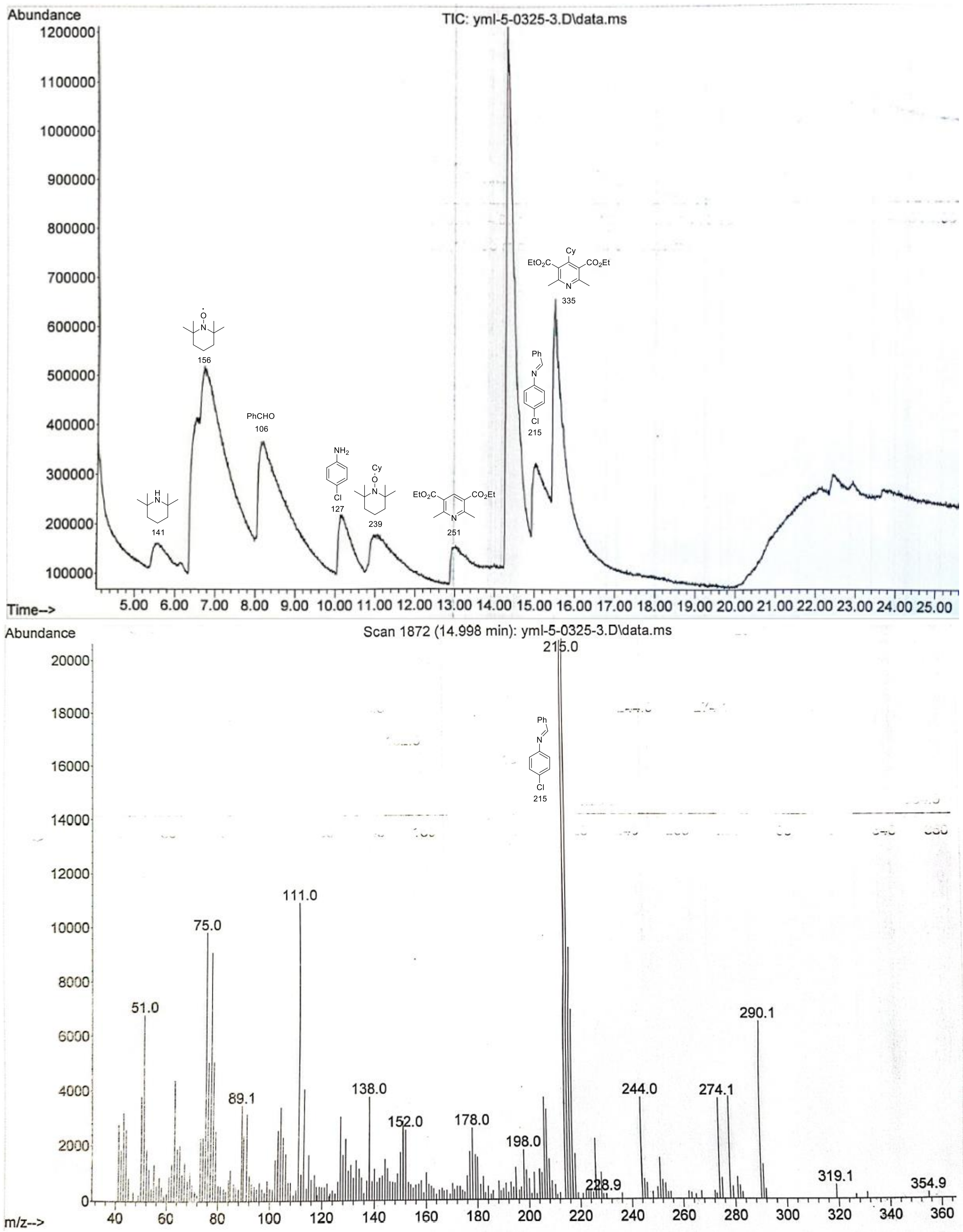

Figure 5 GC-MS of the reaction solvent

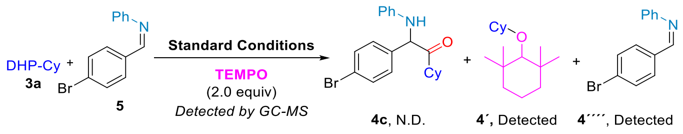

Scheme 3 Radical capture experiments by TEMPO

A 4 mL screw-cap vial was charged with Hantzsch ester **3a** (40.0 mg, 0.12 mmol, 1.2 equiv), imine **5** (0.1 mmol, 26.0 mg, 1.0 equiv), **PC-2** (1.4 mg, 1.5 mol%), and an oven-dried stirring bar. The vial was closed with a Teflon septum and cap and connected to the atmosphere via a needle. After replacing the nitrogen in the vial three times, Aldehyde **1a** (0.1 mmol, 11.0 mg, 1.0 equiv) using a microinjector. Then, $CHCl_3$ (1 mL) was added was added using injector. The vial was then moved to a cannula and transferred into a 300 mL photoautoclave (manufactured by Parr Instrument Company®), under a nitrogen atmosphere. At room temperature, the autoclave was washed with CO three times and charged with 40 bar of CO. The autoclave was placed on a heating plate equipped with a magnetic stirrer and an aluminum block. The reaction mixture was allowed to react at 30 ℃ under UV-A (400-500 nm) for 24 hours. After the reaction was complete, the pressure of the autoclave was carefully released, and the residual CO was washed away with nitrogen. Then, a proper amount of solvent was taken for GC-MS analysis. The result is shown in **Figure 4**. When TEMPO were added to the reaction, no target product **4c** was detected, and alkyl radical was trapped by TEMPO. Data in agreement with that reported previously.[4]

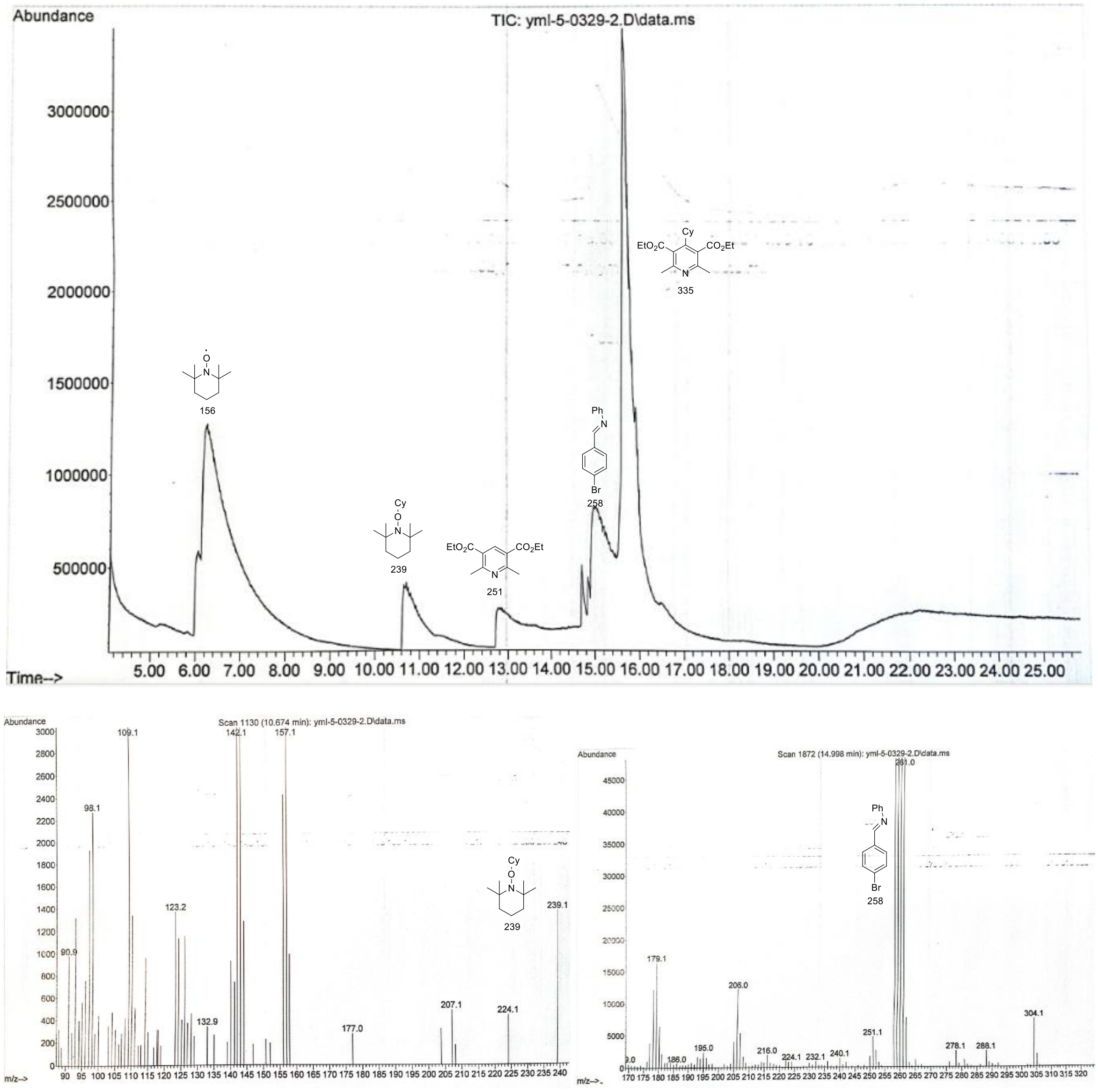

Figure 6 GC-MS of the reaction solvent

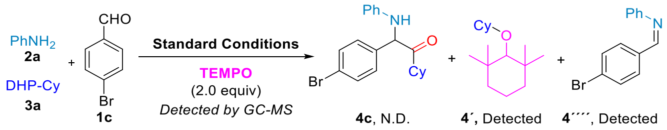

Scheme 4 Radical capture experiments by TEMPO

A 4 mL screw-cap vial was charged with Hantzsch ester **3a** (40.0 mg, 0.12 mmol, 1.2 equiv), $ArNH_2$.HCl **2b** (0.1 mmol, 17.0 mg, 1.0 equiv), **PC-2** (1.4 mg, 1.5 mol%), and an oven-dried stirring bar. The vial was closed with a Teflon septum and cap and connected to the atmosphere via a needle. After replacing the nitrogen in the vial three times, Aldehyde **1c** (0.1 mmol, 19.0 mg, 1.0 equiv) using a microinjector. Then, $CHCl_3$ (1 mL) was added was added using injector. The vial was then moved to a cannula and transferred into a 300 mL photoautoclave (manufactured by Parr Instrument Company®), under a nitrogen atmosphere. At room temperature, the autoclave was washed with CO three times and charged with 40 bar of CO. The autoclave was placed on a heating plate equipped with a magnetic stirrer and an aluminum block. The reaction mixture was allowed to react at 30 ℃ under UV-A (400-500 nm) for 24 hours. After the reaction was complete, the pressure of the autoclave was carefully released, and the residual CO was washed away with nitrogen. Then, a proper amount of solvent was taken for GC-MS analysis. The result is shown in **Figure 4**. When TEMPO were added to the reaction, no target product **4c** was detected, and alkyl radical was trapped by TEMPO. Data in agreement with that reported previously.[4]

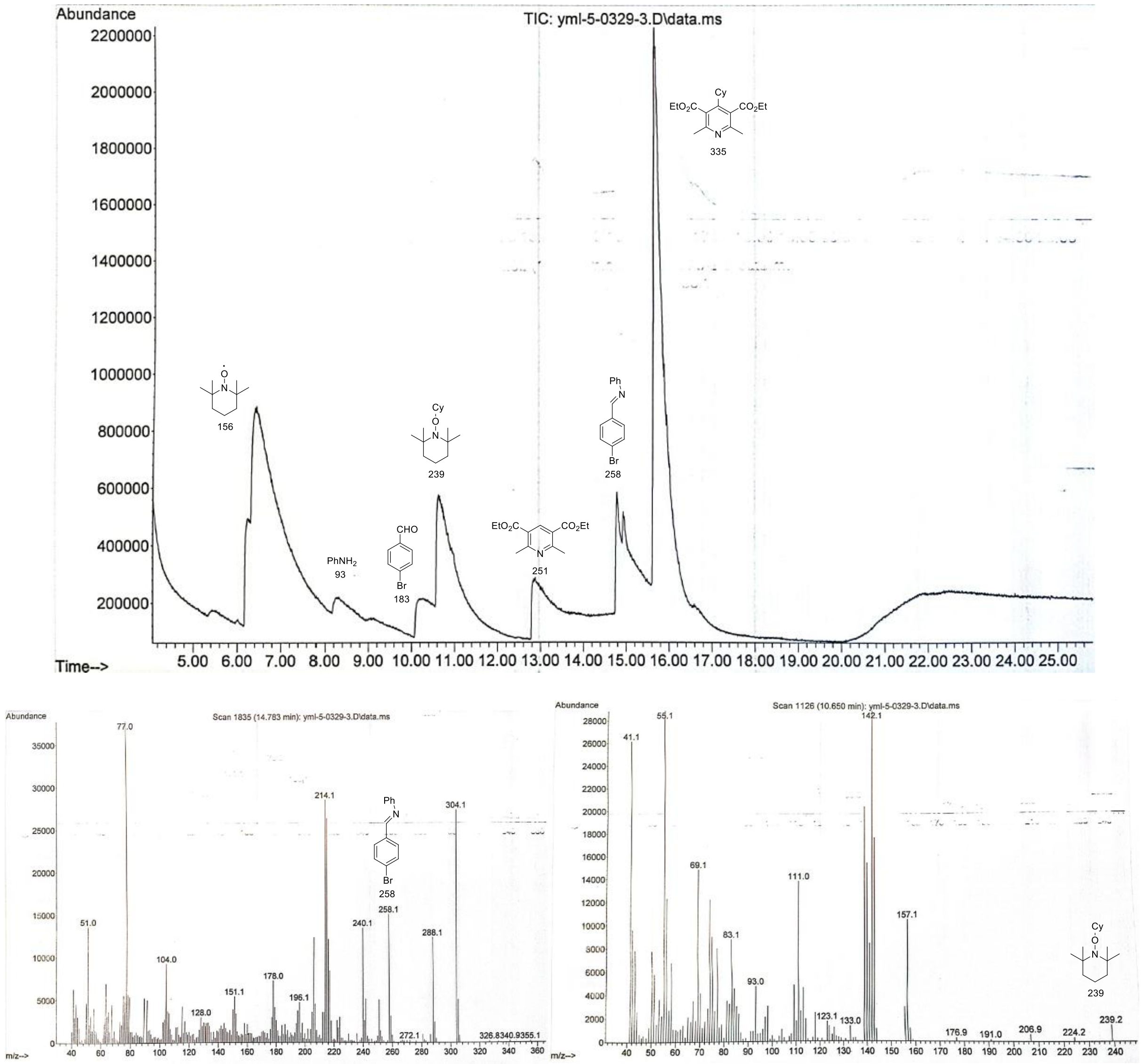

Figure 7 GC-MS of the reaction solvent

6. References

[1]. http://www.lumatec.de/de/produkte/uv-lichtquelle-superlite-s04/.

[2]. G. Li, R. Chen, L. Wu, Q. Fu, X. Zhang, Z. Tang, *Angew. Chem. Int. Ed*. **2013**, *52*, 8432 – 8436.

[3]. F. Zhao, C.-L. Lia, X.-F. Wu, *Chem. Commun.*, **2020**, *56*, 9182-9185.

[4]. a) D. Liu, Y. Li, X. Qi, C. Liu, Y. Lan, A. Lei, *Org. Lett*. **2015**, *17*, 998 – 1001; b) F. Zhao, X.-W. Gu, R. Franke, X.-F. Wu, *Angew. Chem. Int. Ed*. **2022**, *61*, e202214812.

7. NMR Spectra

4a ^{1}H NMR (300 MHz, $CDCl_3$)

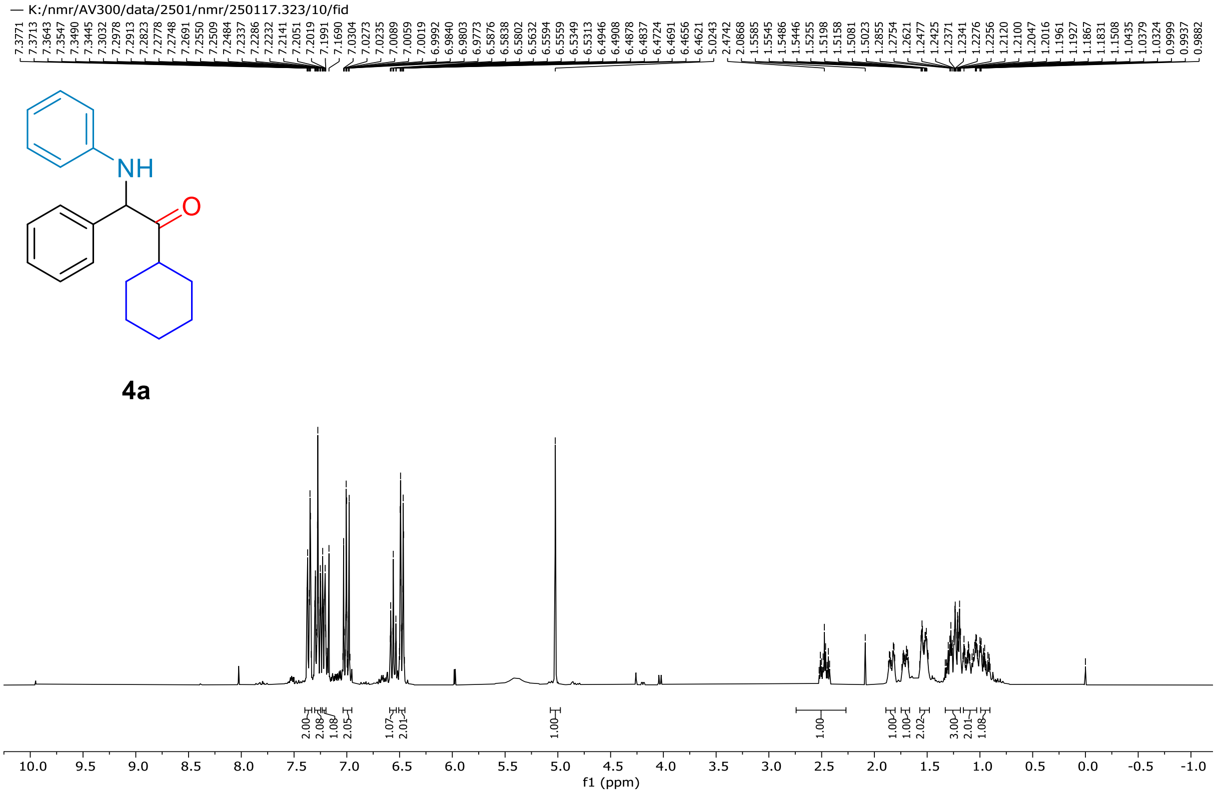

4a ^{13}C NMR (75 MHz, $CDCl_3$)

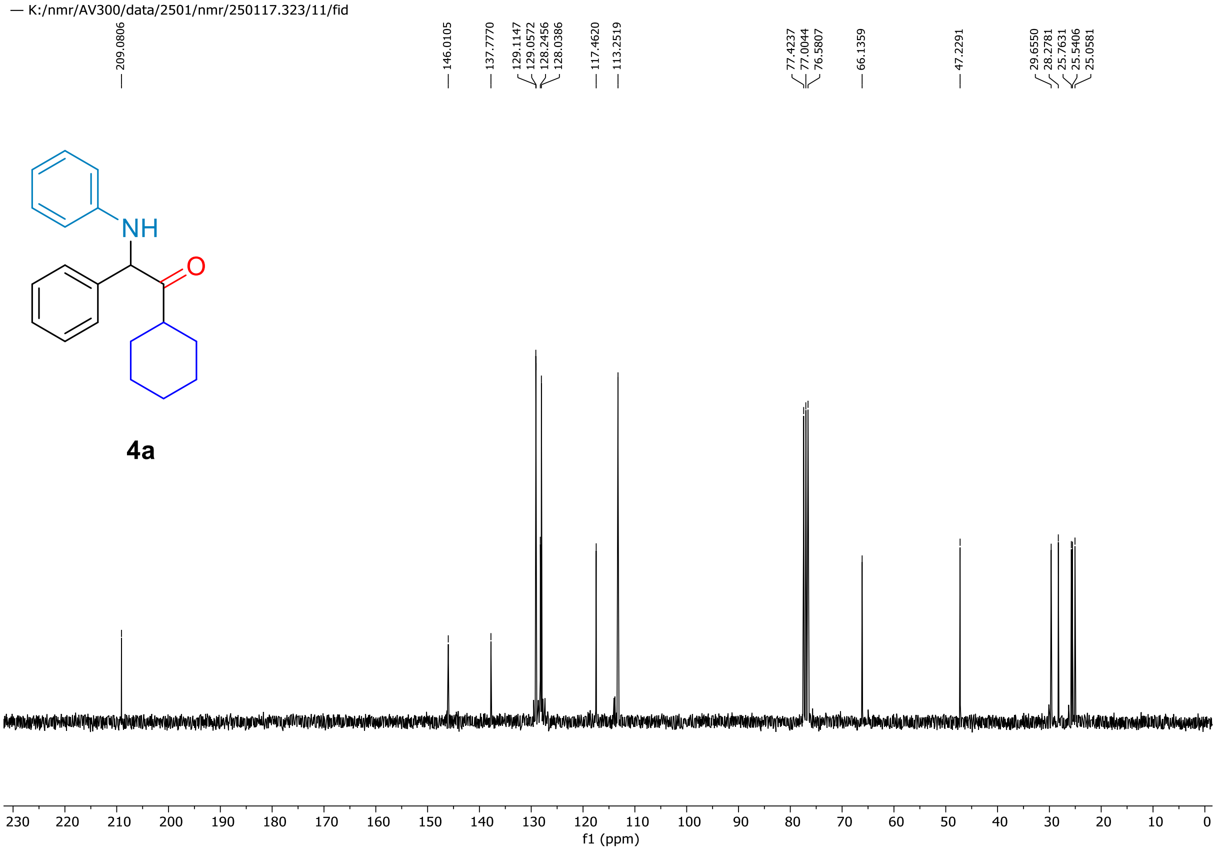

4b ^{1}H NMR (300 MHz, $CDCl_3$)

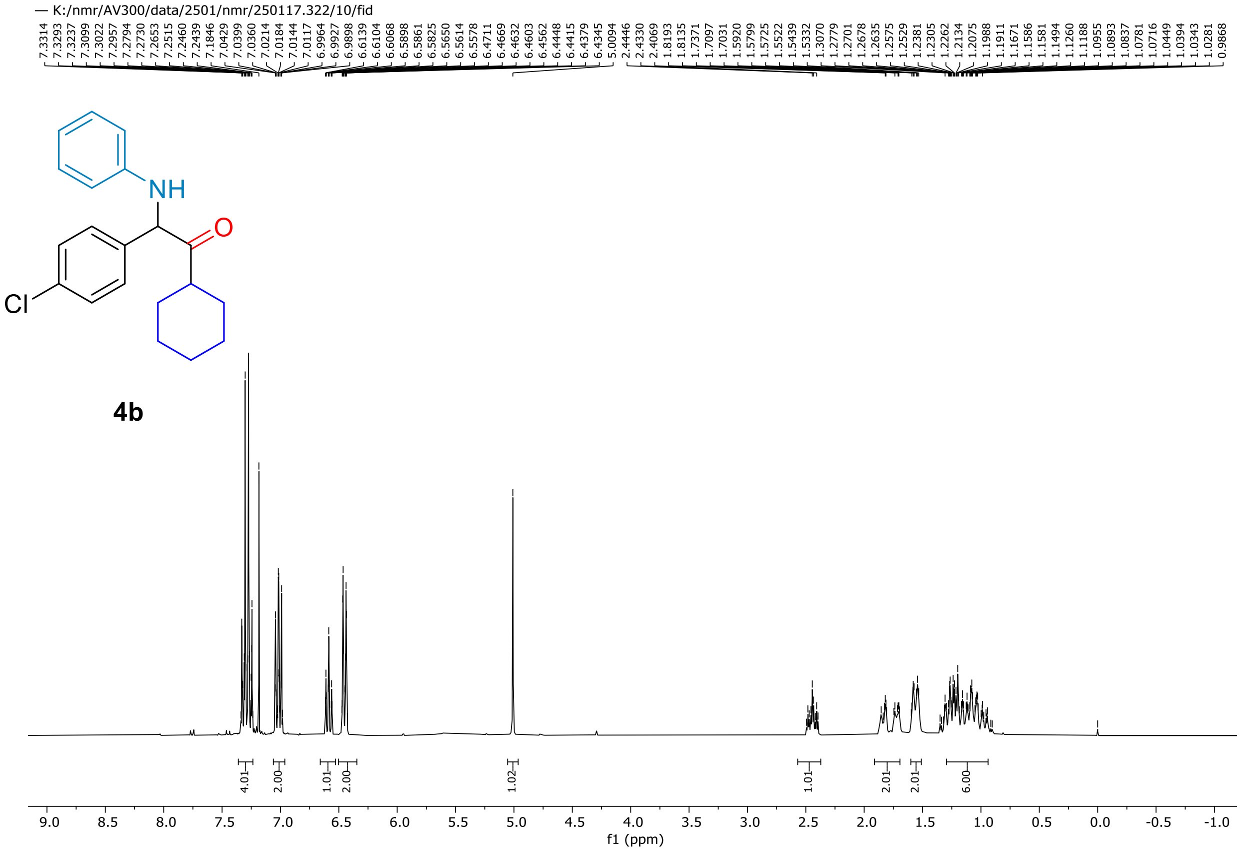

4b ^{13}C NMR (75 MHz, $CDCl_3$)

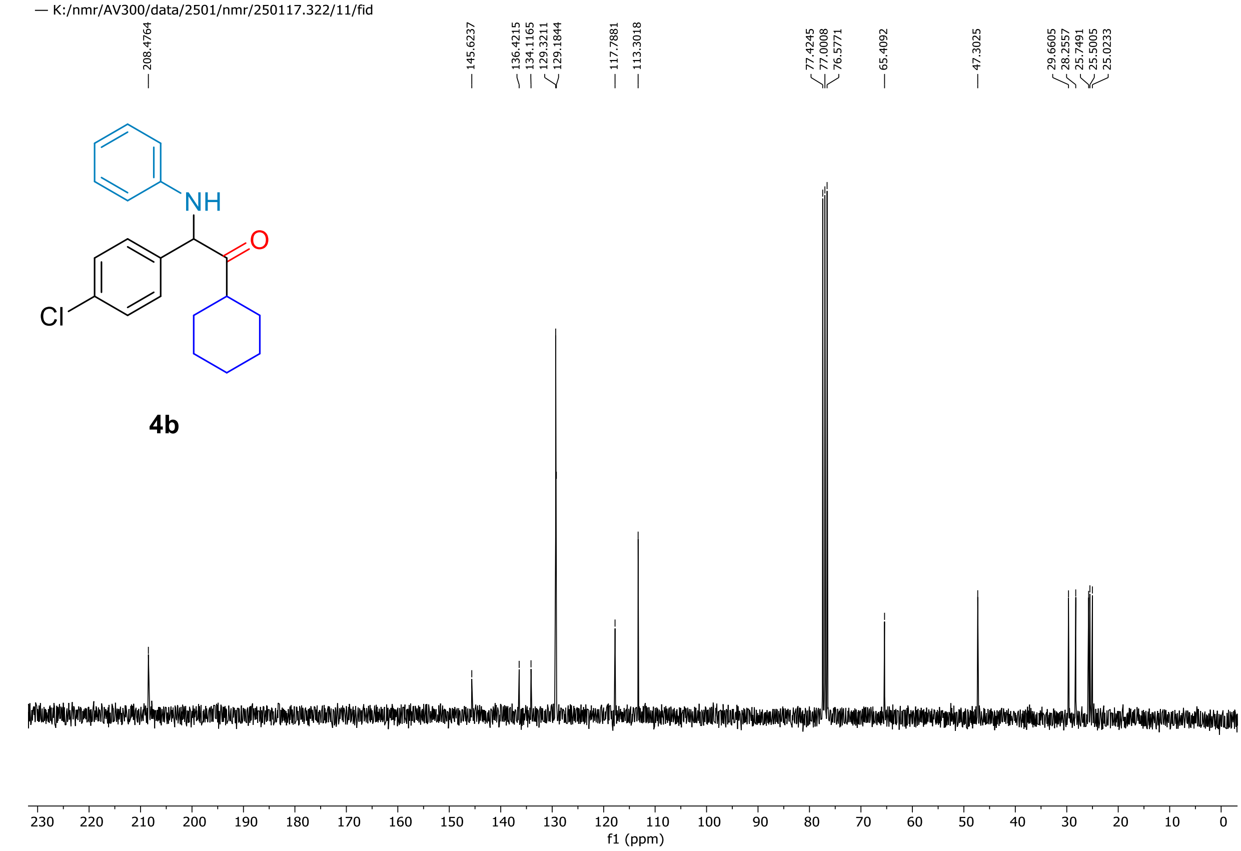

4c ^{1}H NMR (300 MHz, $CDCl_3$)

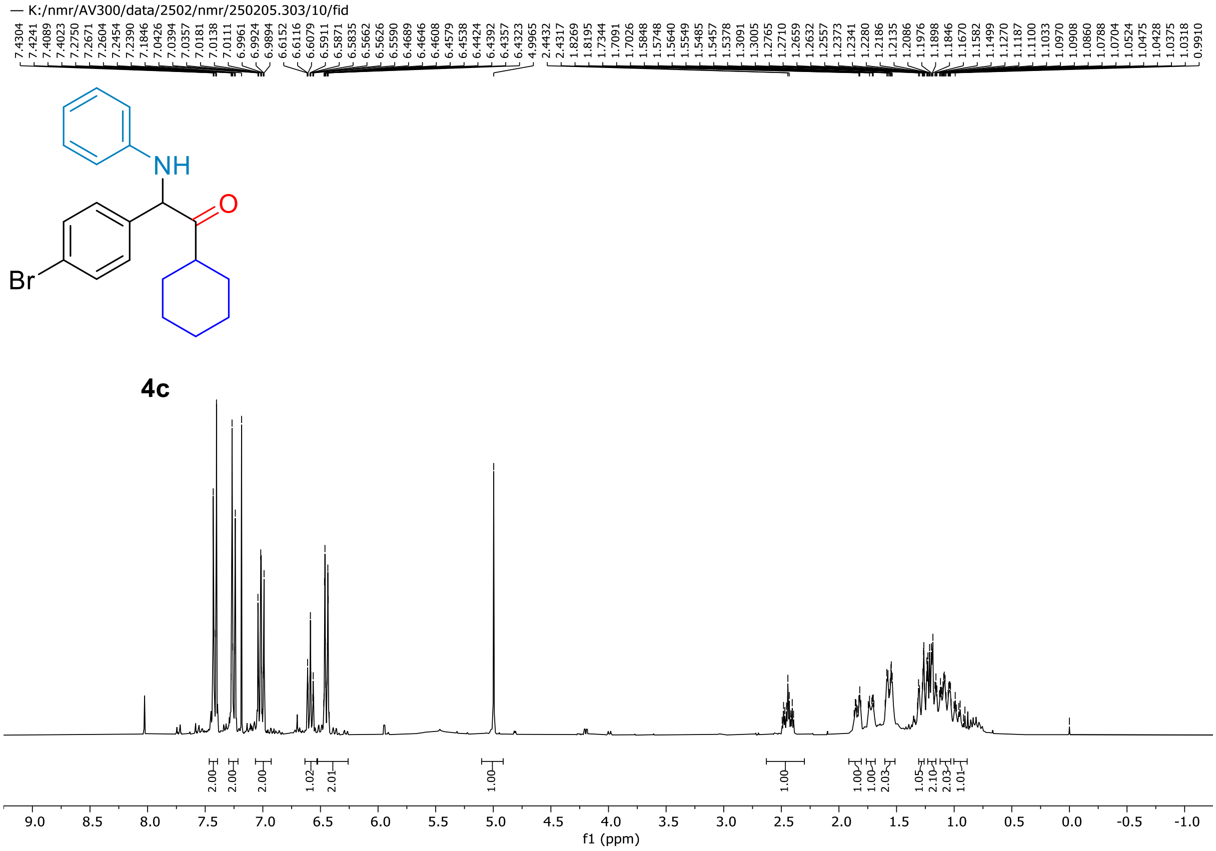

4c ^{13}C NMR (75 MHz, $CDCl_3$)

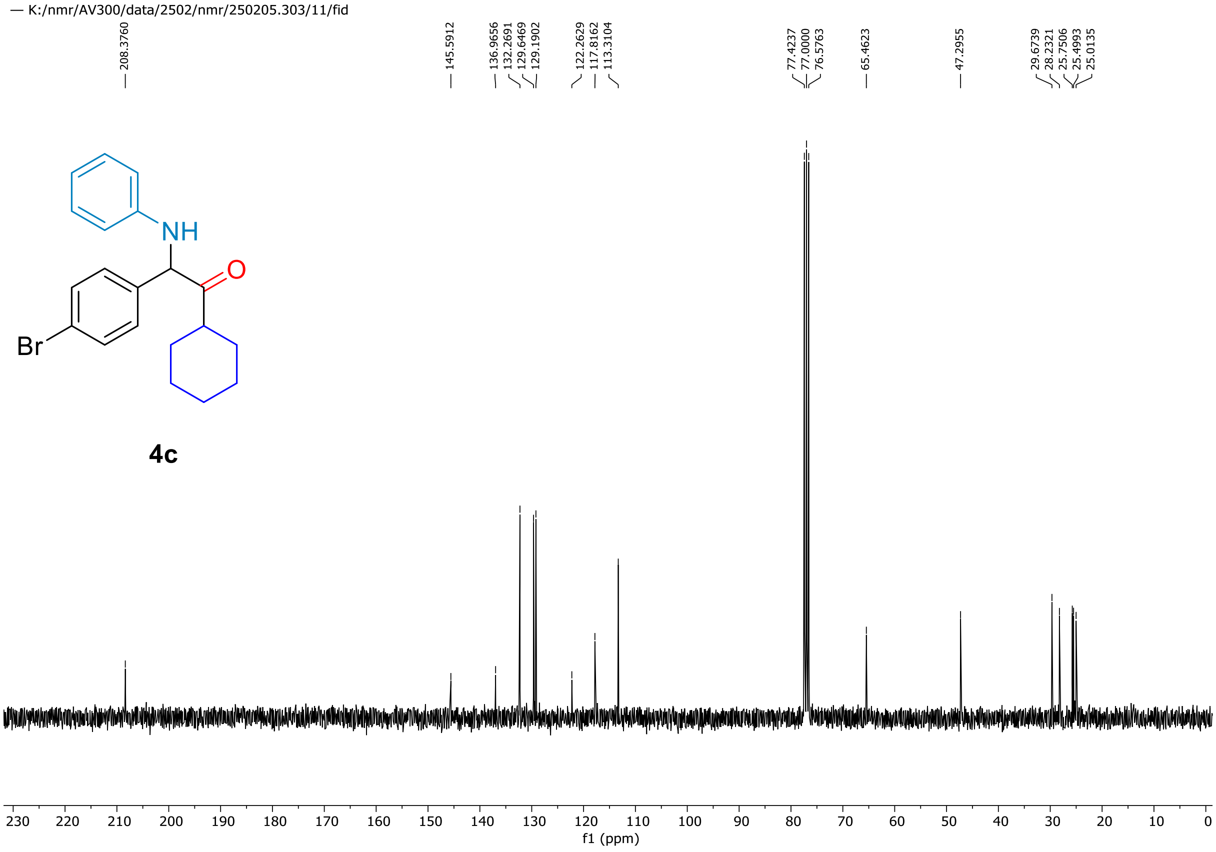

4d ^{1}H NMR (300 MHz, $CDCl_3$)

— K:/nmr/AV300/data/2501/nmr/250123.324/10/fid

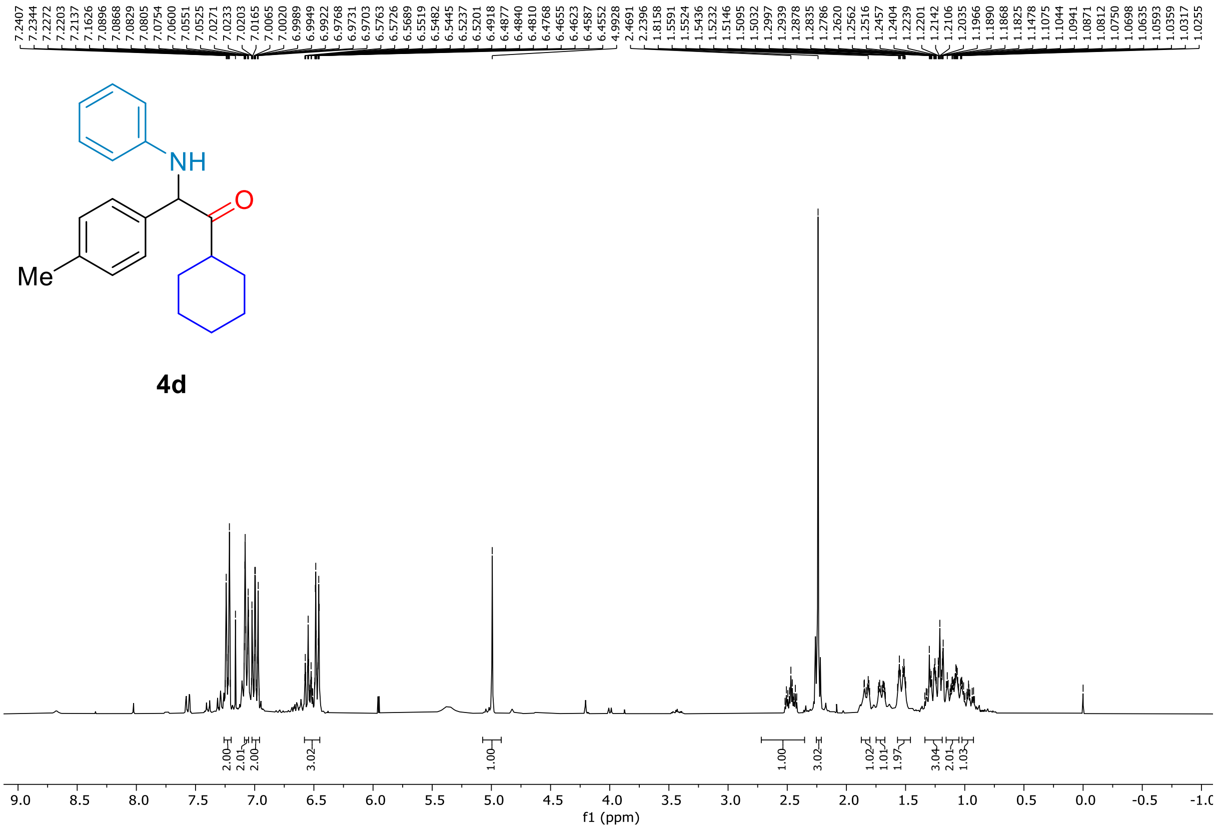

4d

4d ^{13}C NMR (75 MHz, $CDCl_3$)

— K:/nmr/AV300/data/2501/nmr/250123.324/11/fid

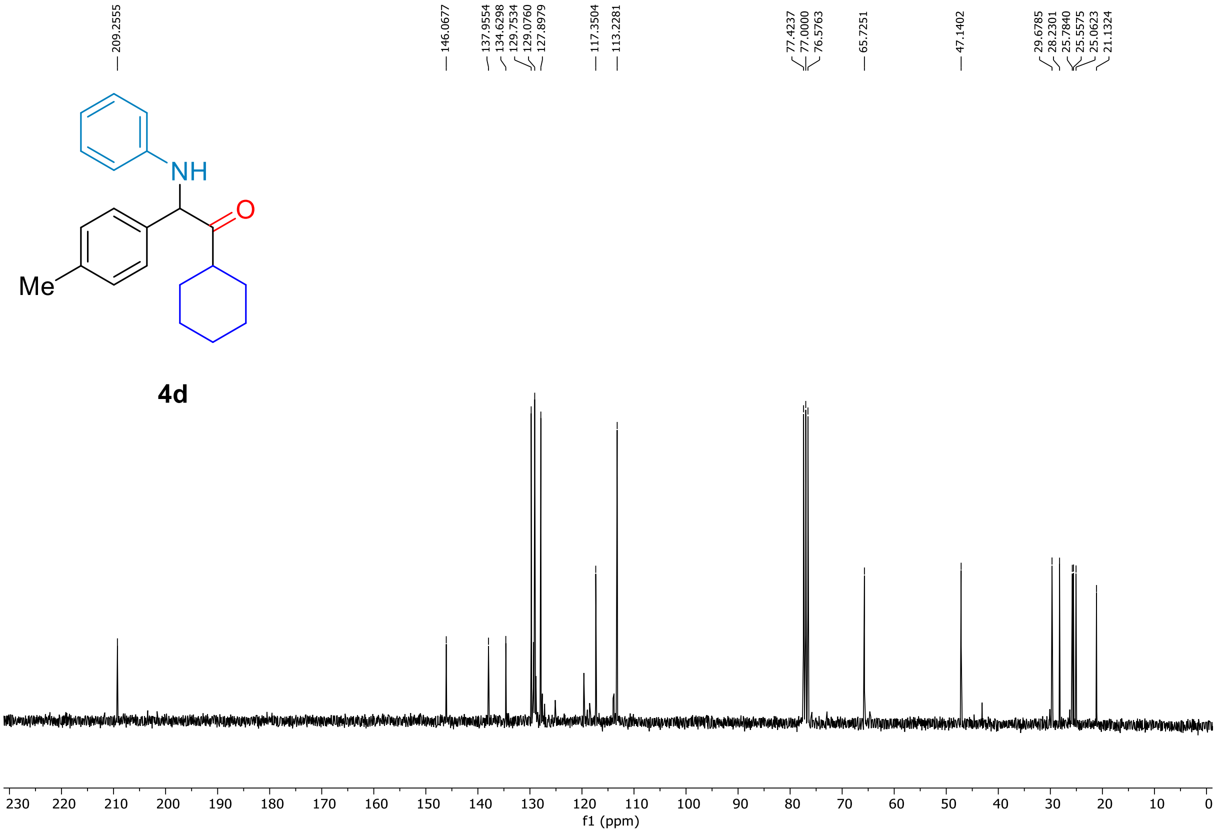

4d

4e ^{1}H NMR (300 MHz, $CDCl_3$)

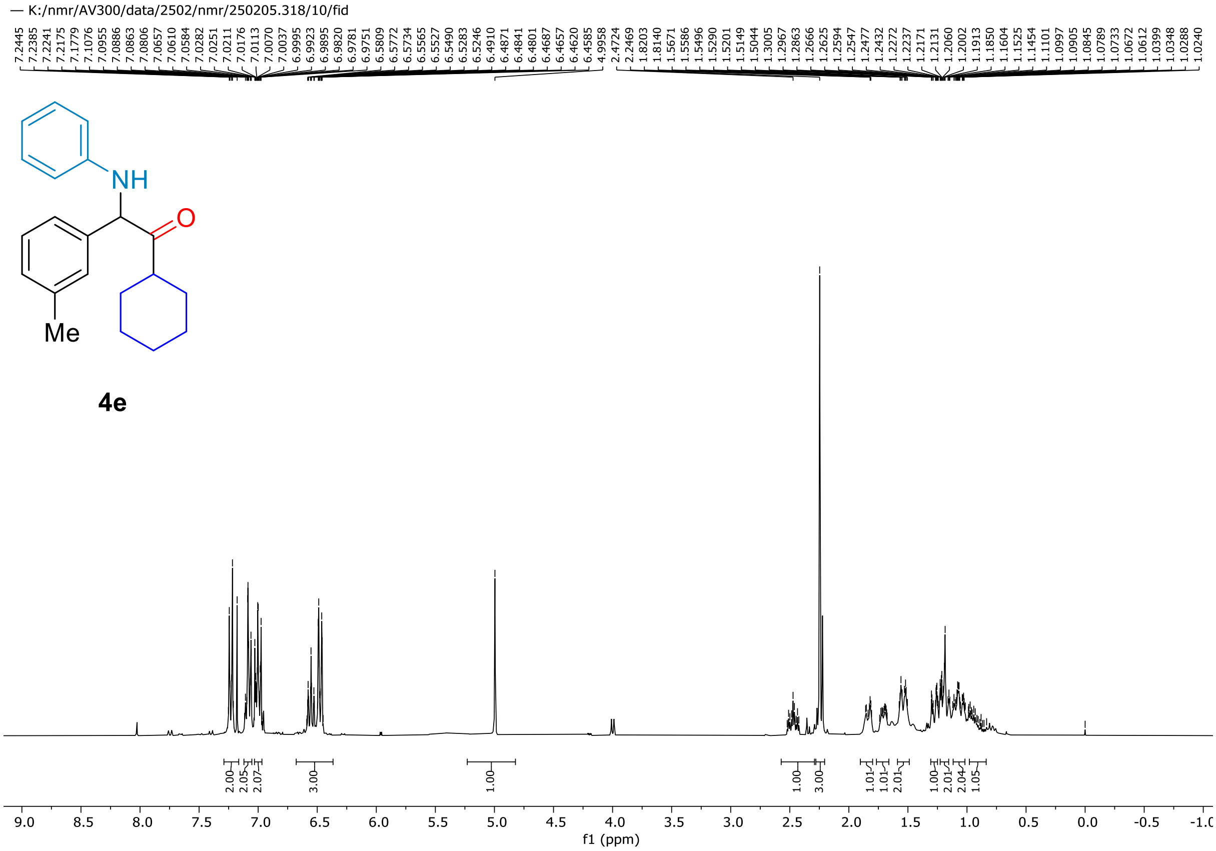

4e ^{13}C NMR (75 MHz, $CDCl_3$)

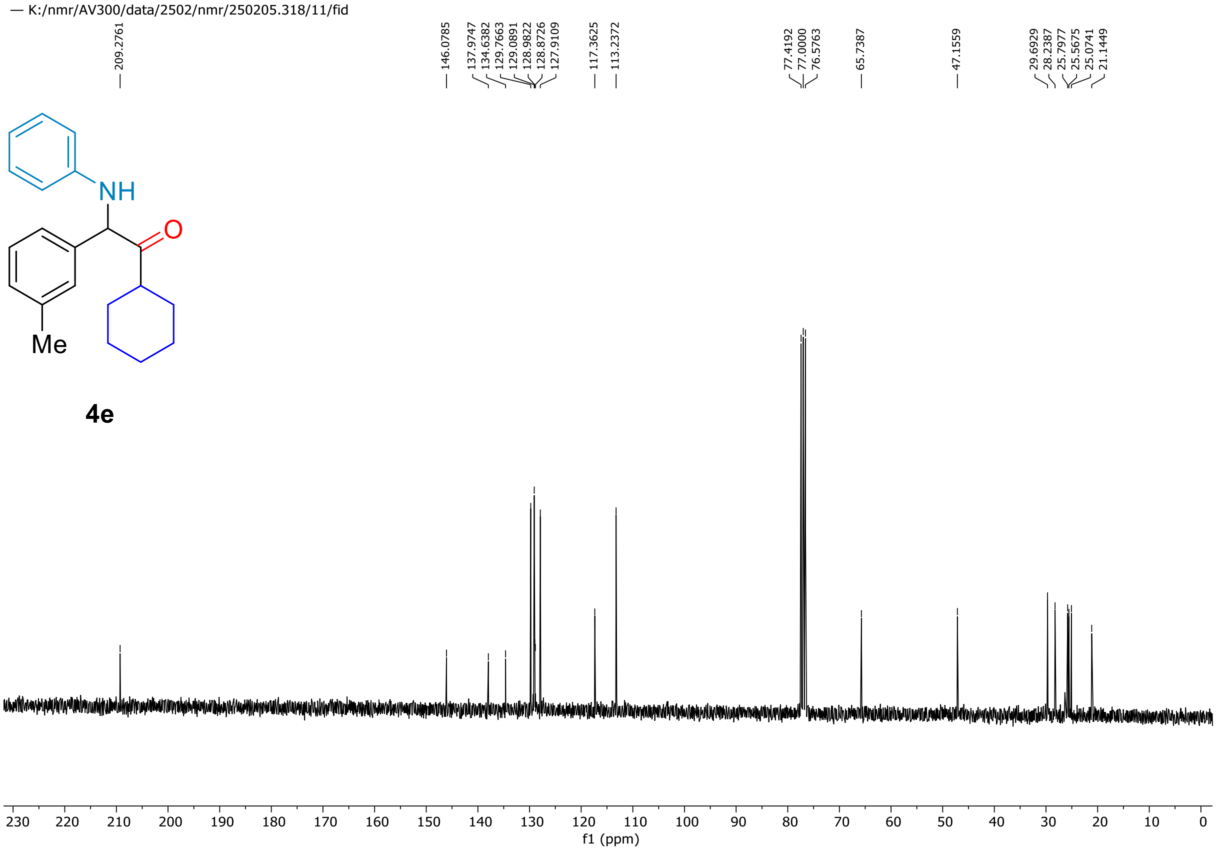

4f ^{1}H NMR (300 MHz, $CDCl_3$)

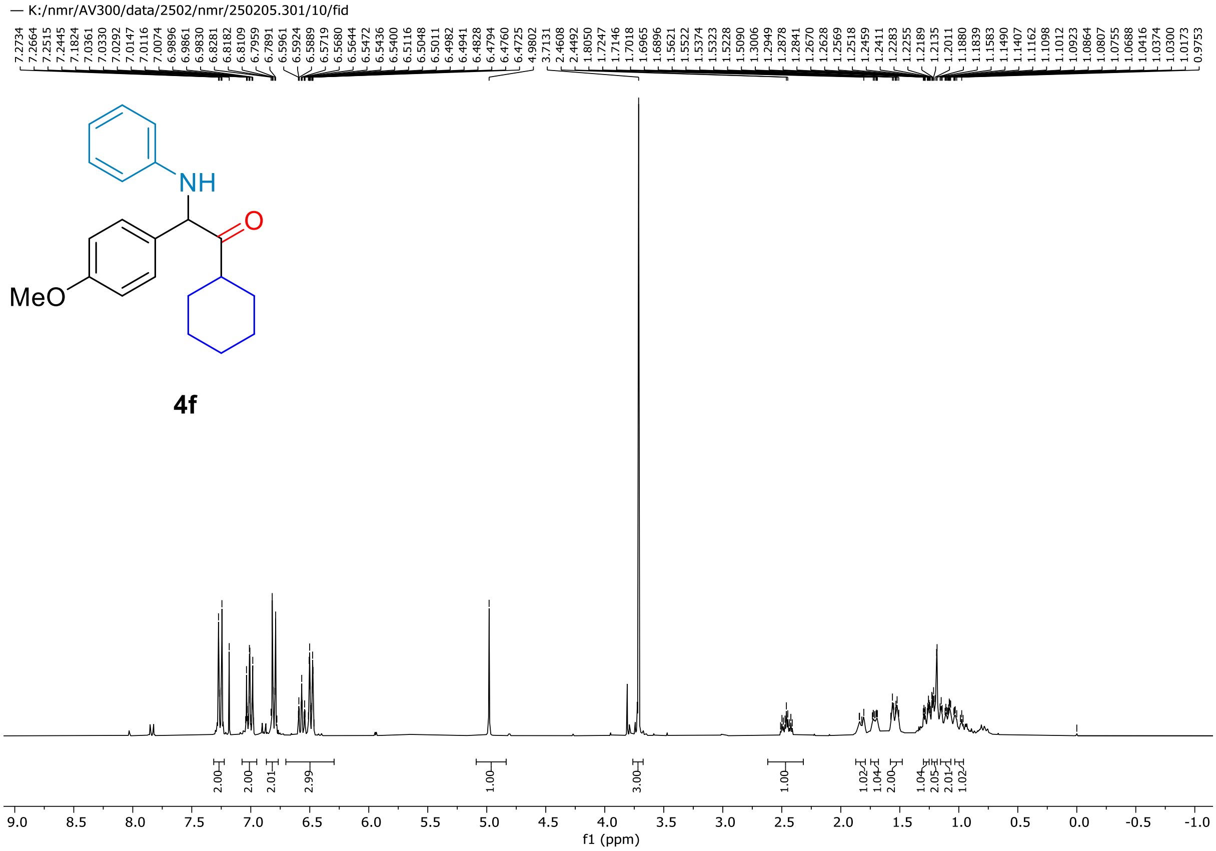

4f ^{13}C NMR (75 MHz, $CDCl_3$)

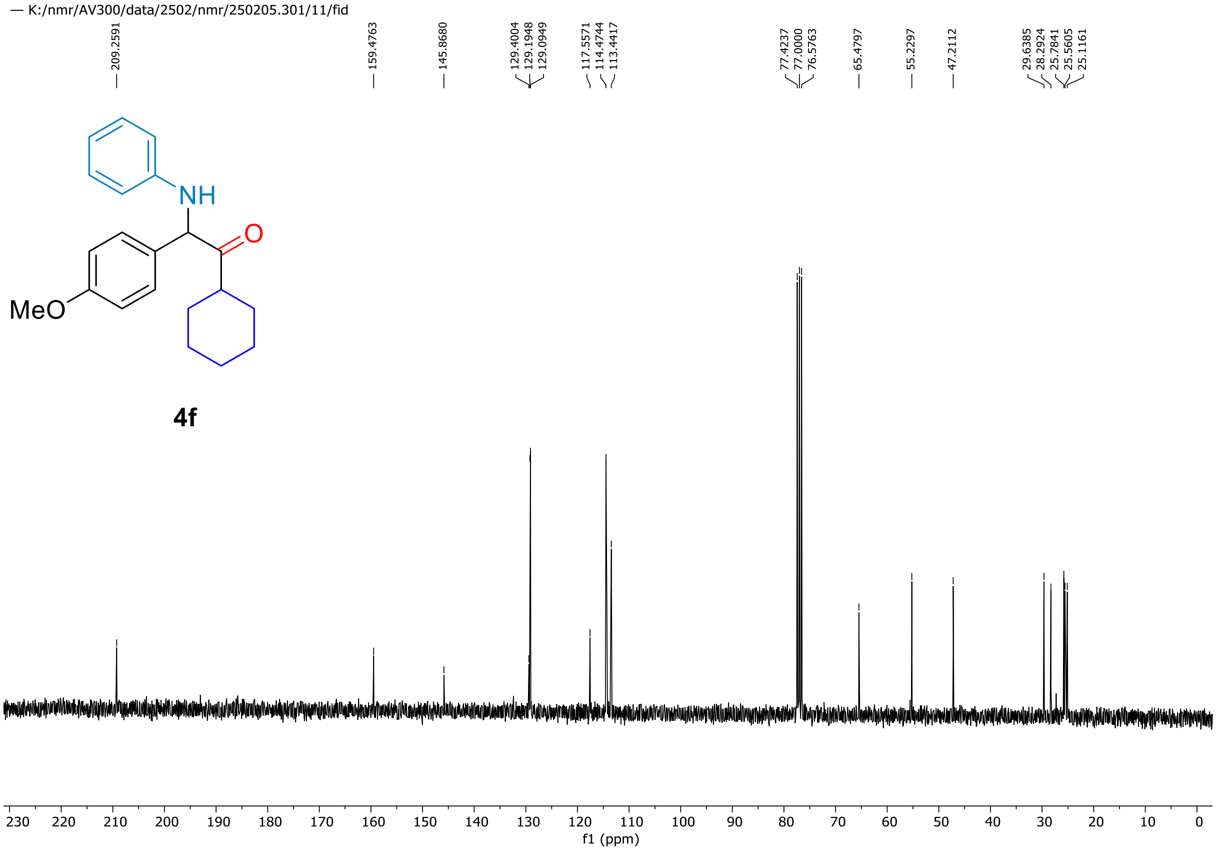

4g ^{1}H NMR (400 MHz, $CDCl_3$)

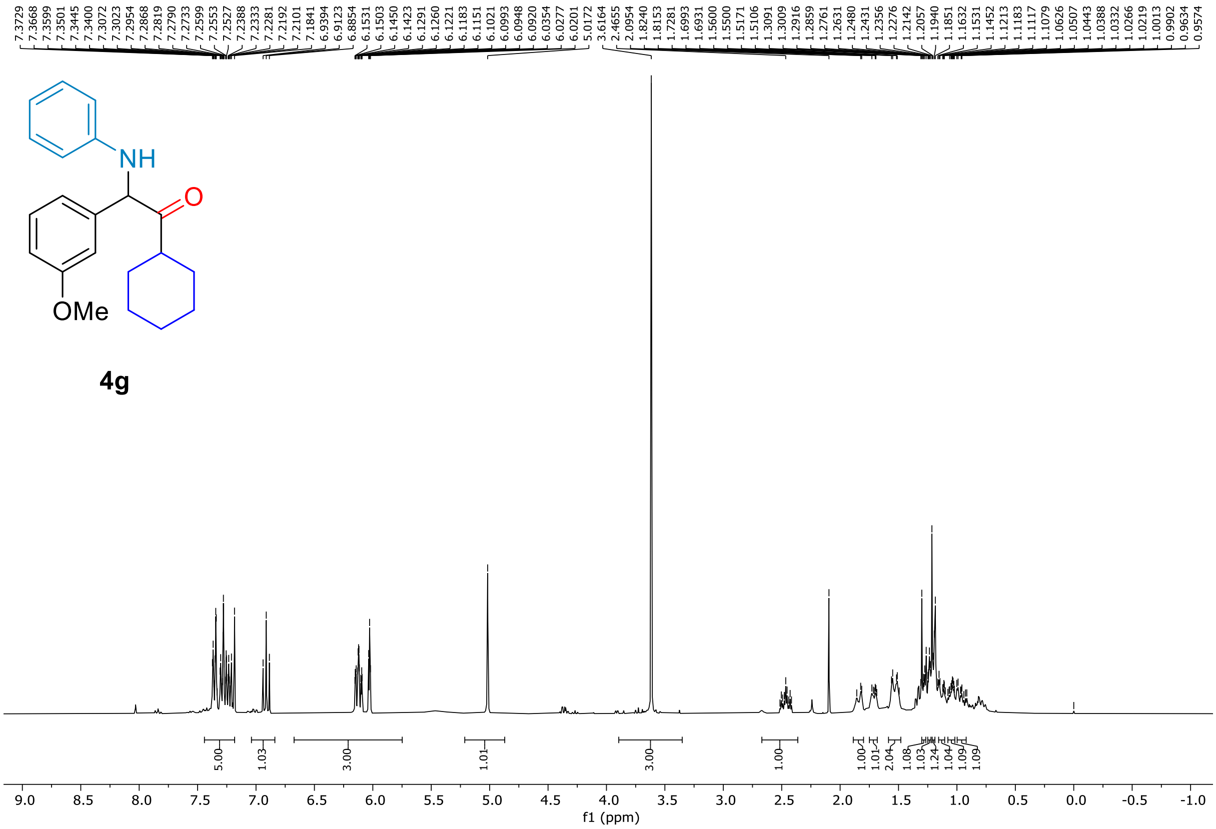

4g

4g ^{13}C NMR (101 MHz, $CDCl_3$)

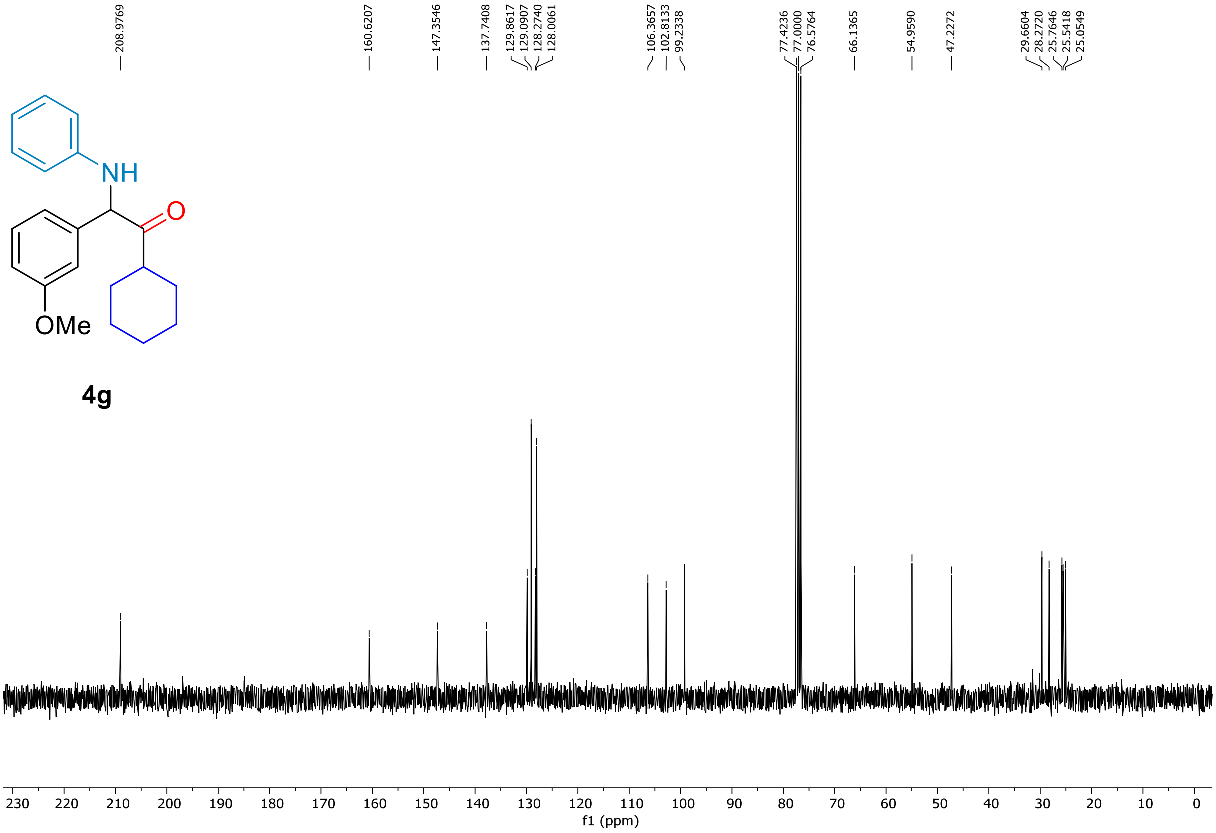

4g

4h ^{1}H NMR (300 MHz, $CDCl_3$)

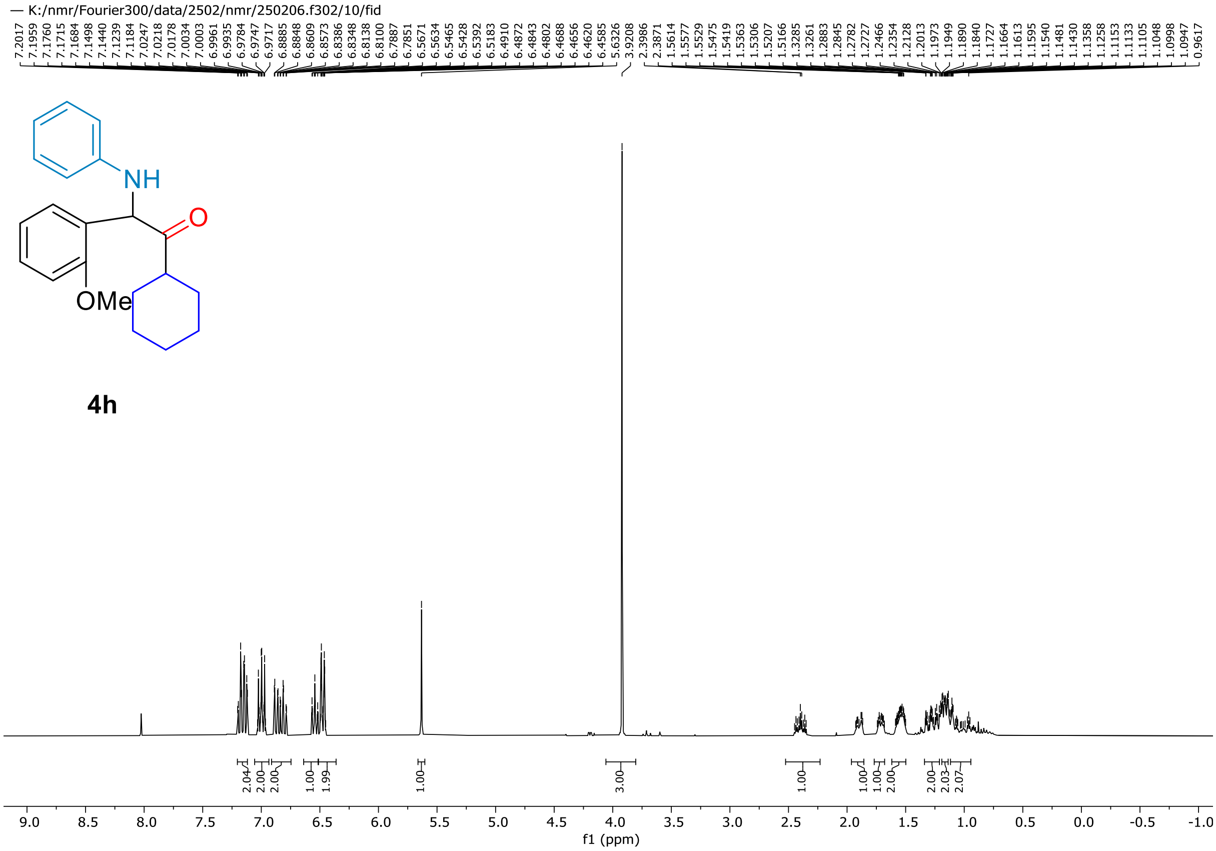

4h ^{13}C NMR (75 MHz, $CDCl_3$)

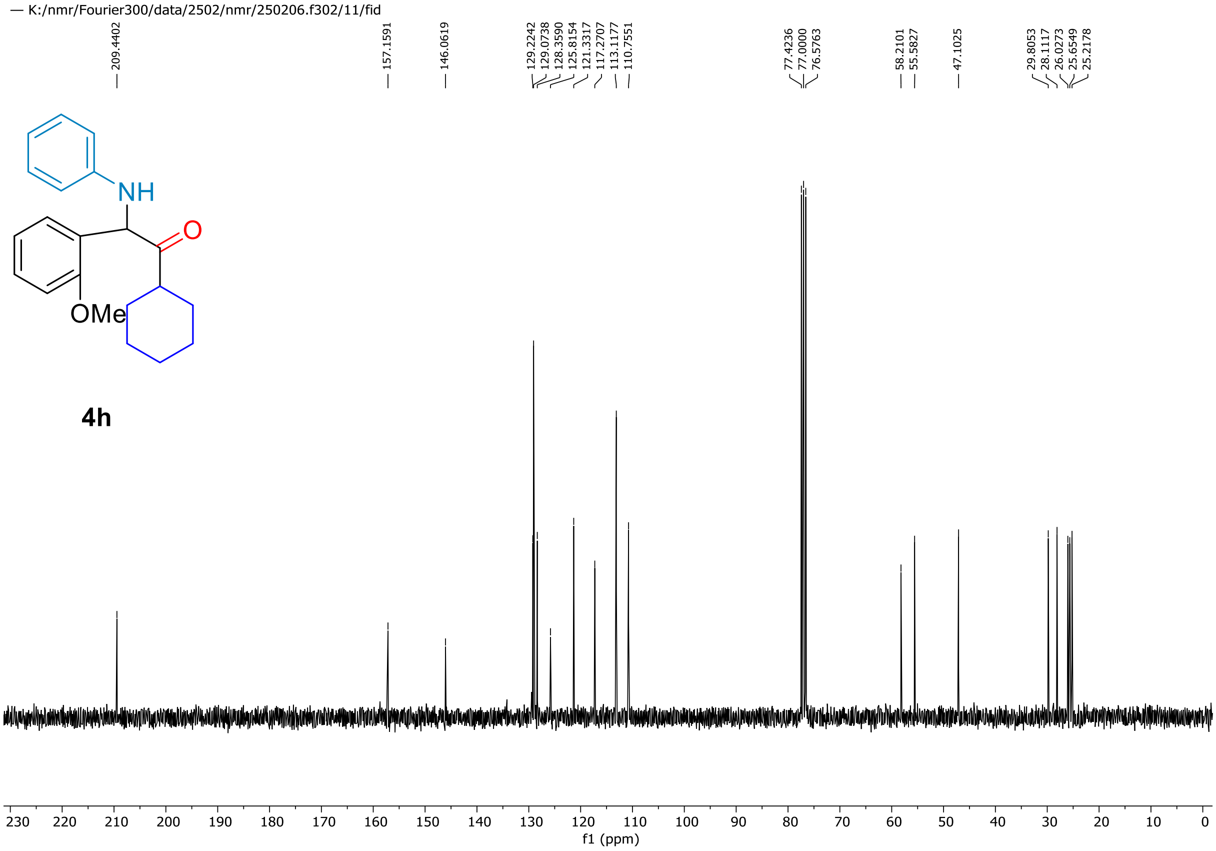

4i ^{1}H NMR (400 MHz, CDCl$_3$)

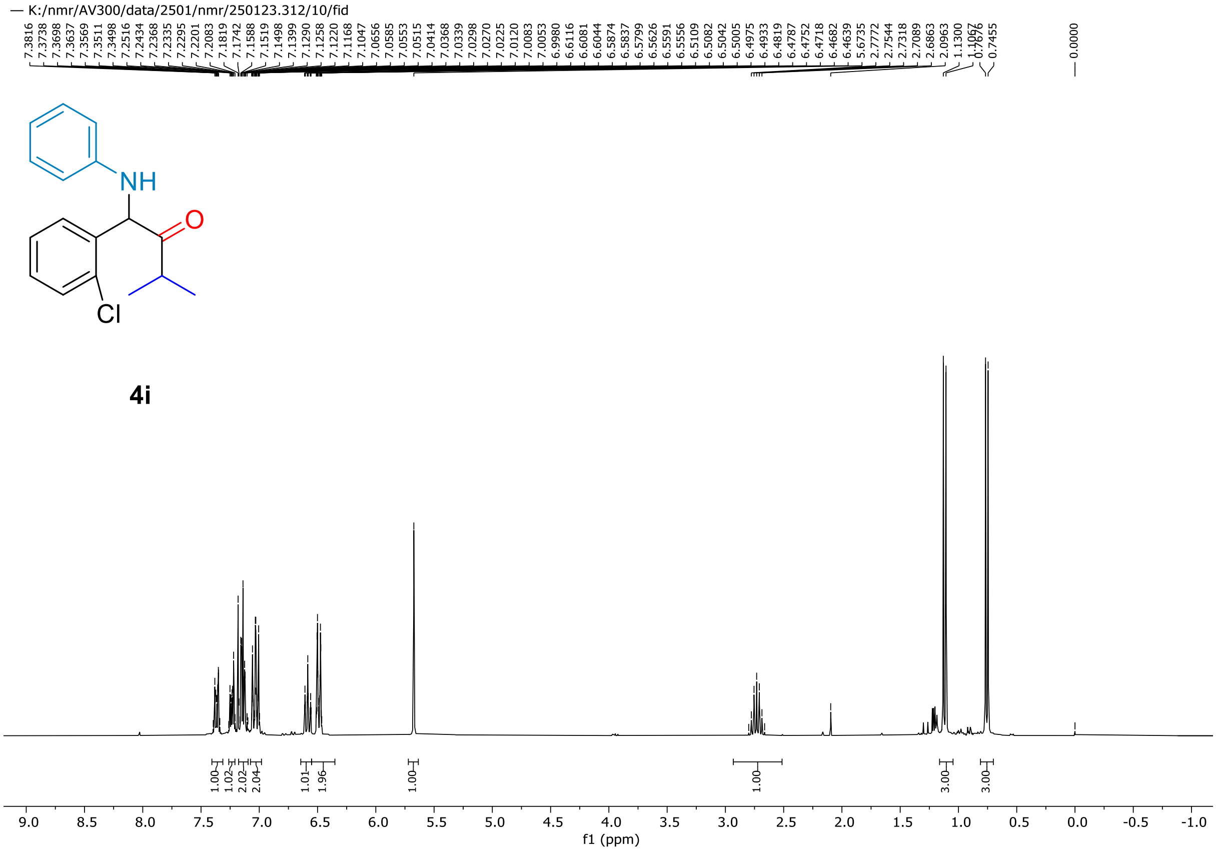

4i ^{13}C NMR (101 MHz, CDCl$_3$)

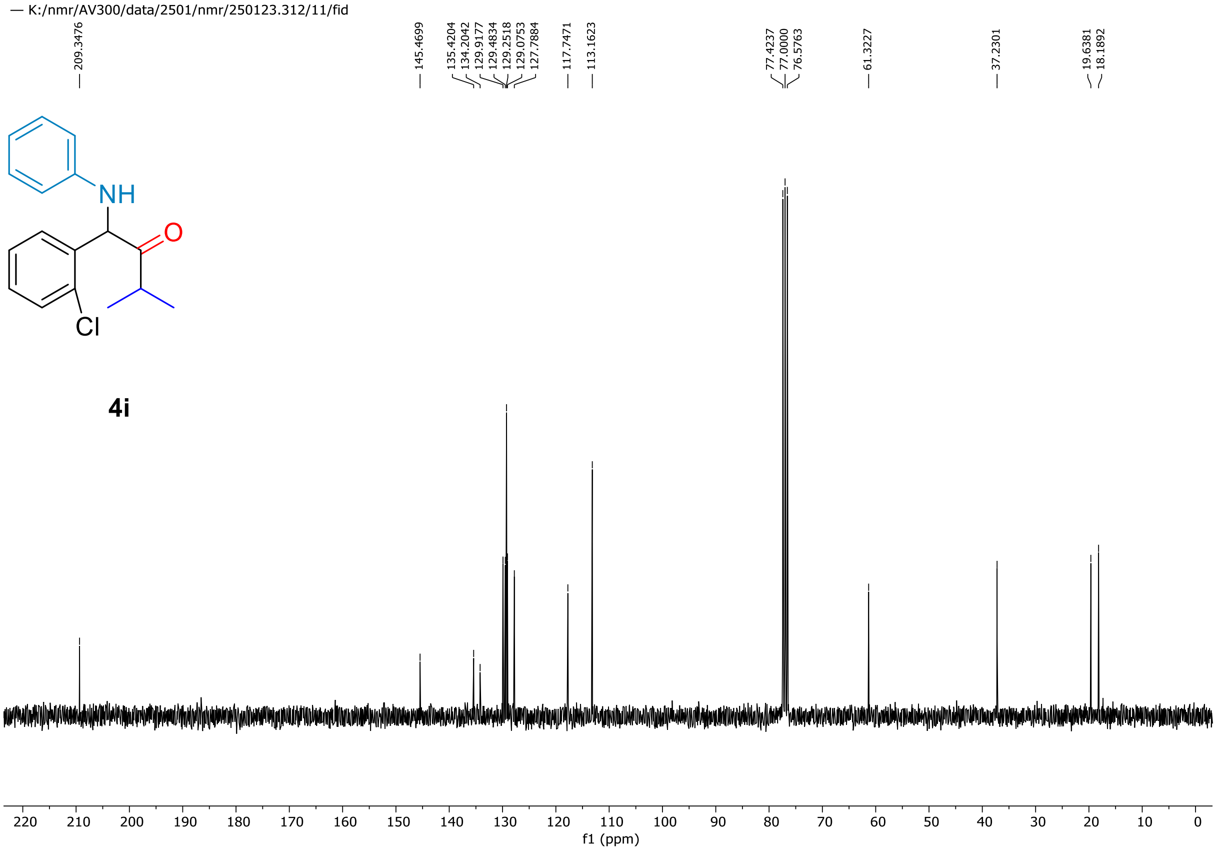

4j ^{1}H NMR (300 MHz, $CDCl_3$)

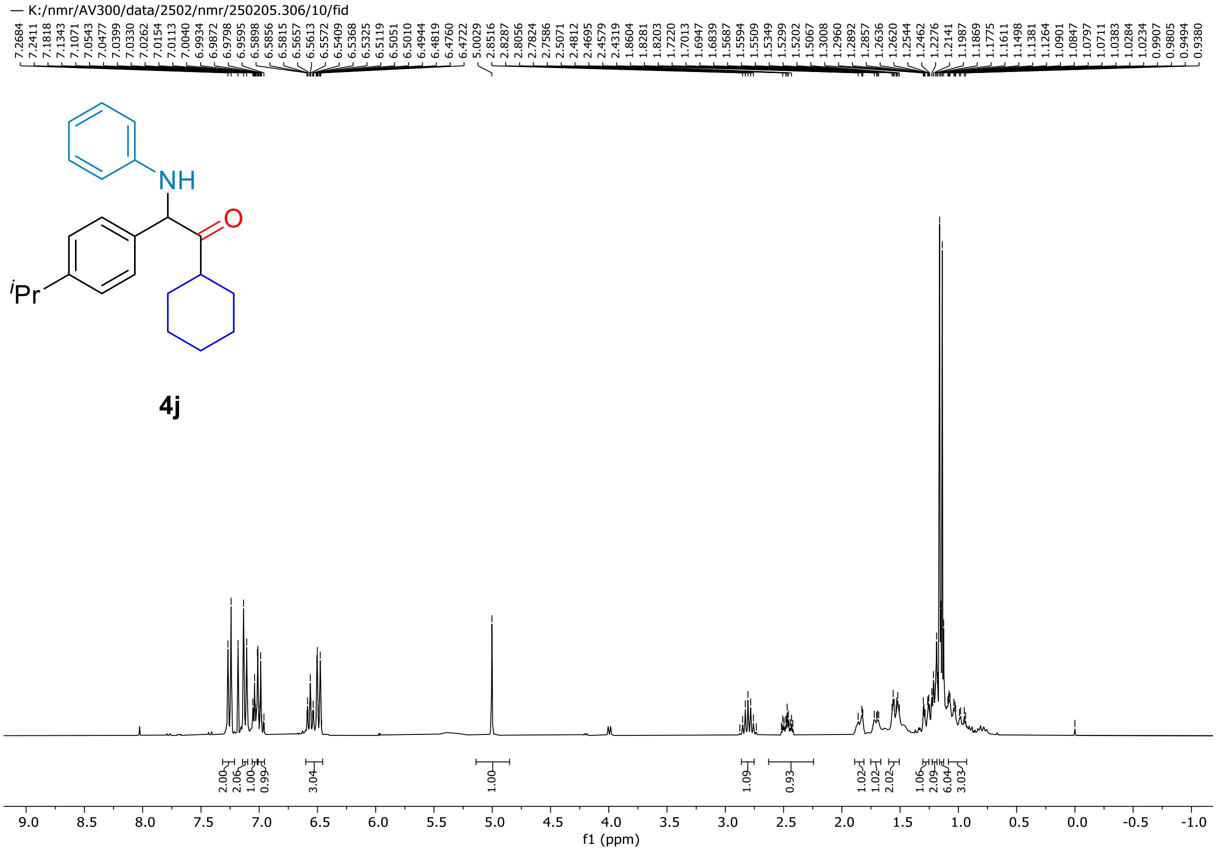

4j ^{13}C NMR (75 MHz, $CDCl_3$)

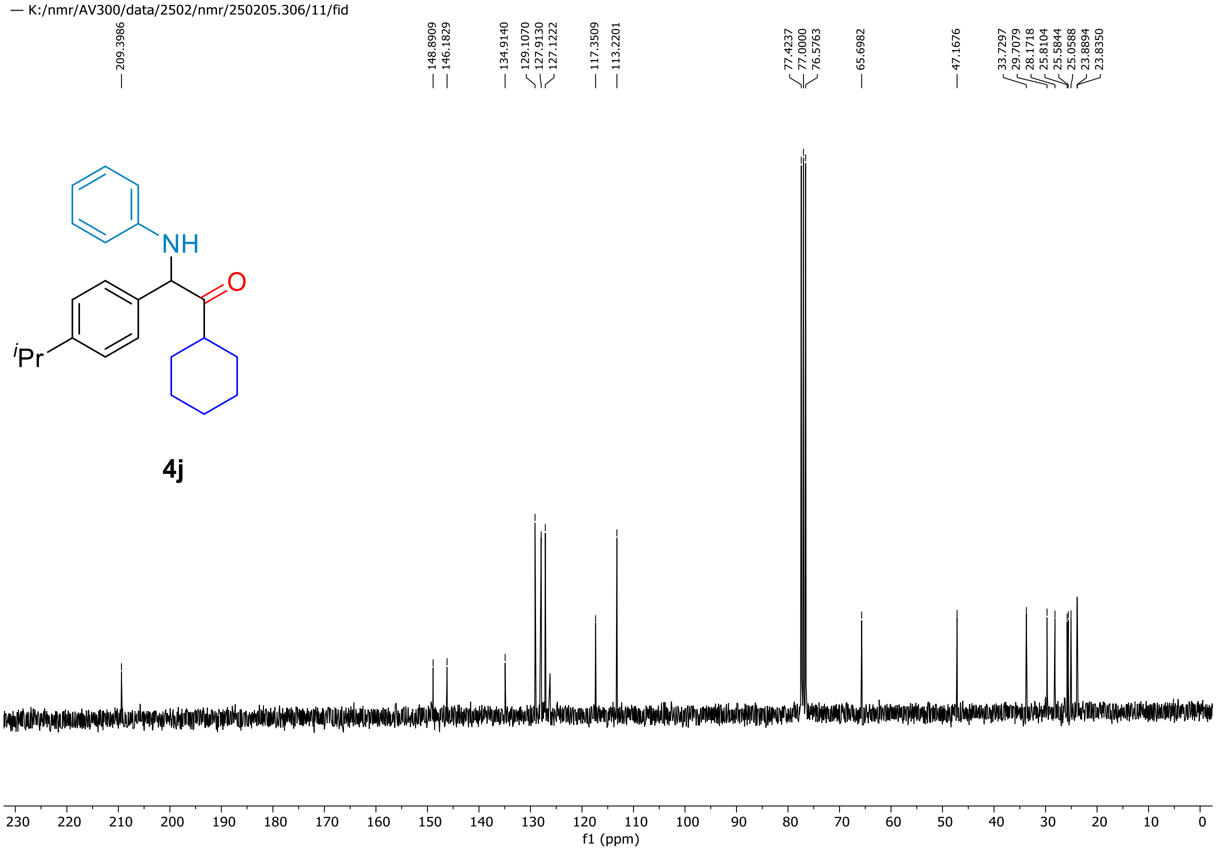

4k ^{1}H NMR (300 MHz, CDCl$_3$)

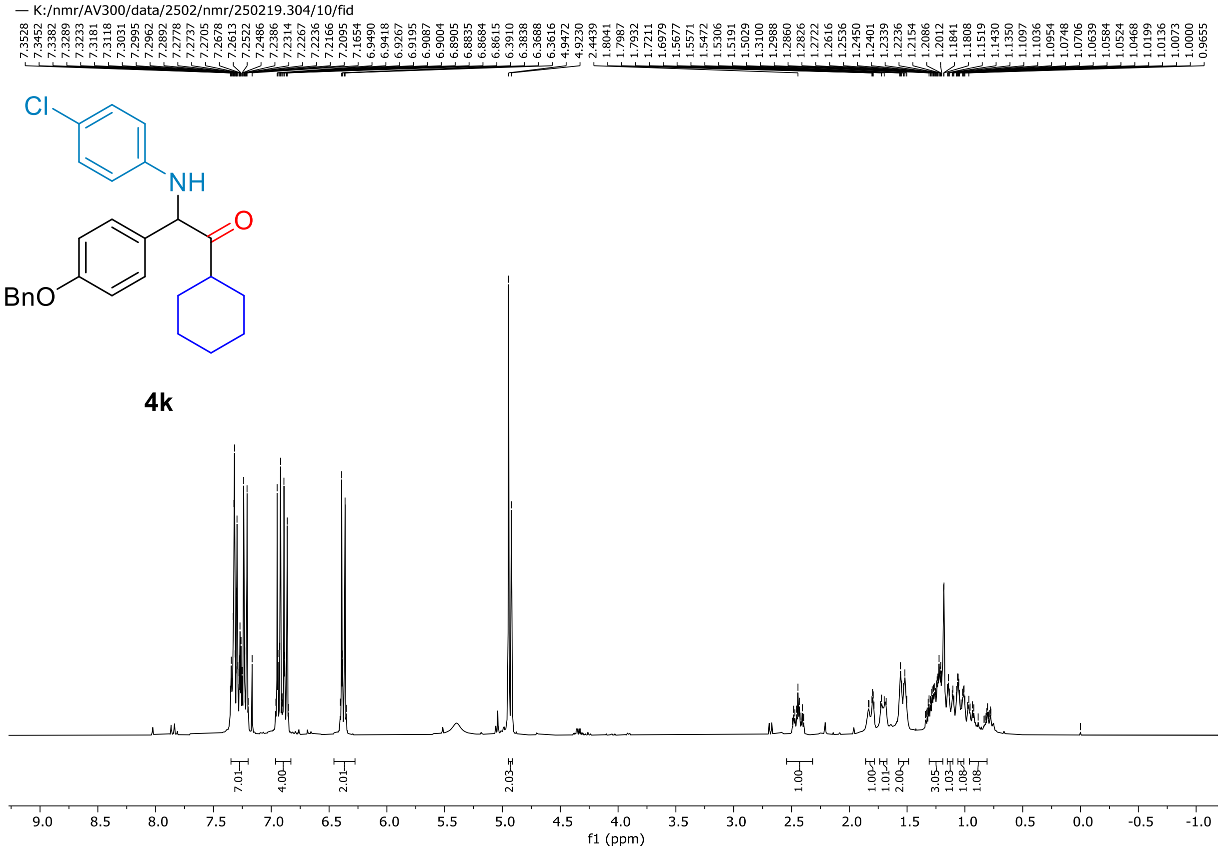

4k ^{13}C NMR (75 MHz, CDCl$_3$)

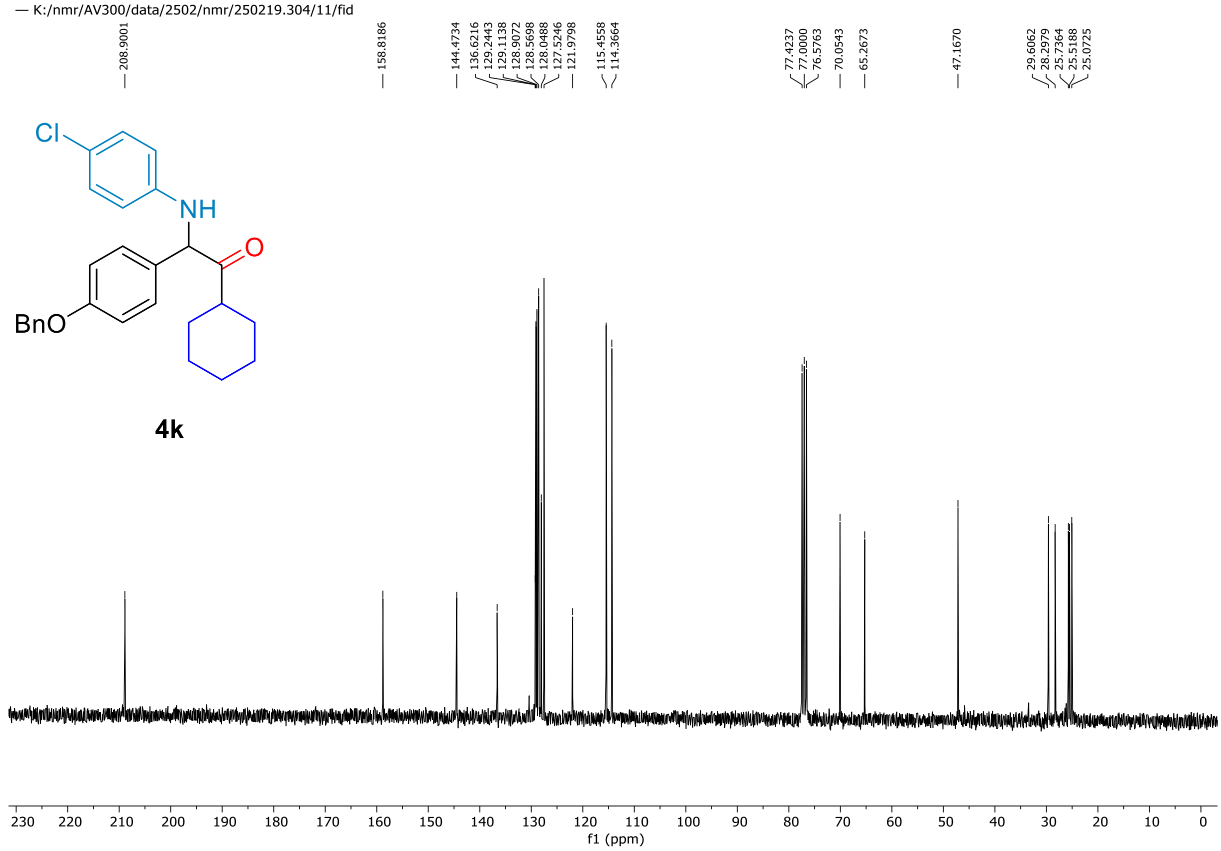

4l ^{1}H NMR (300 MHz, $CDCl_3$)

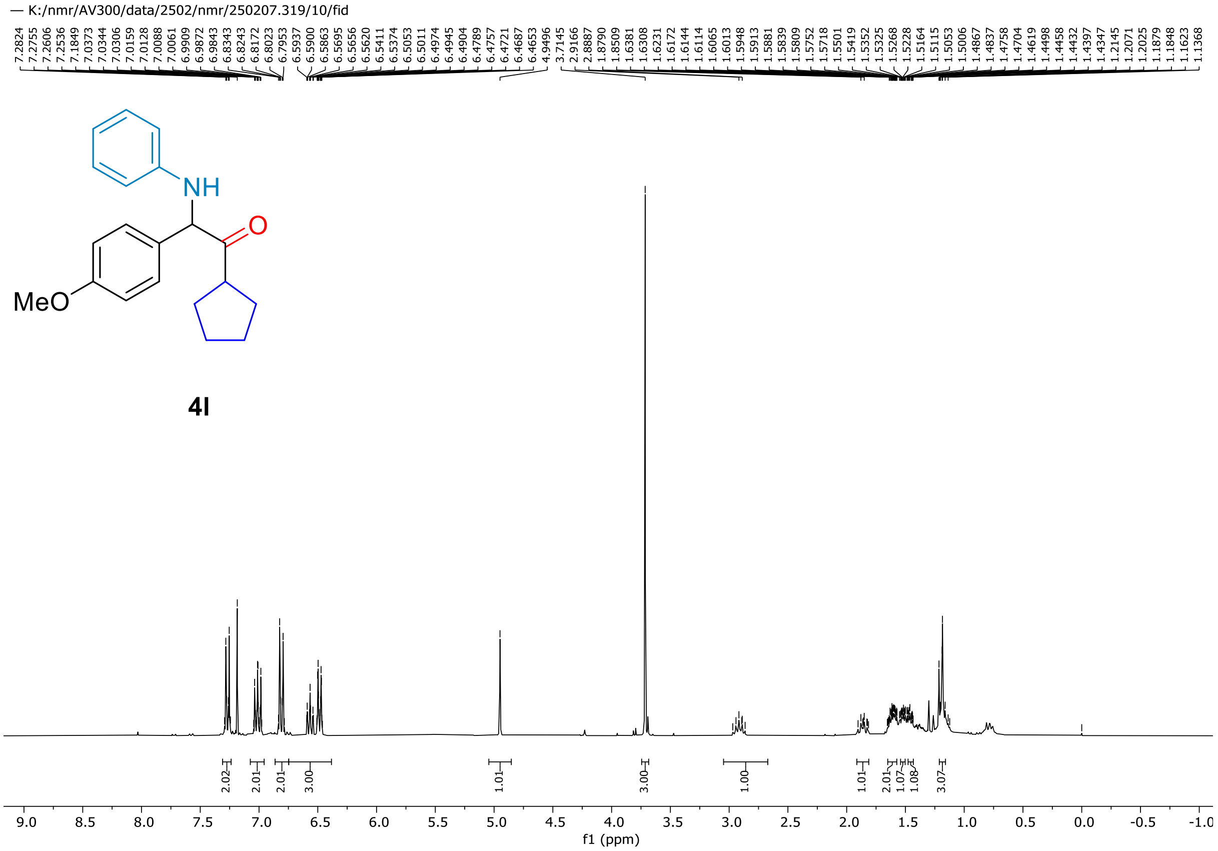

4l ^{13}C NMR (75 MHz, $CDCl_3$)

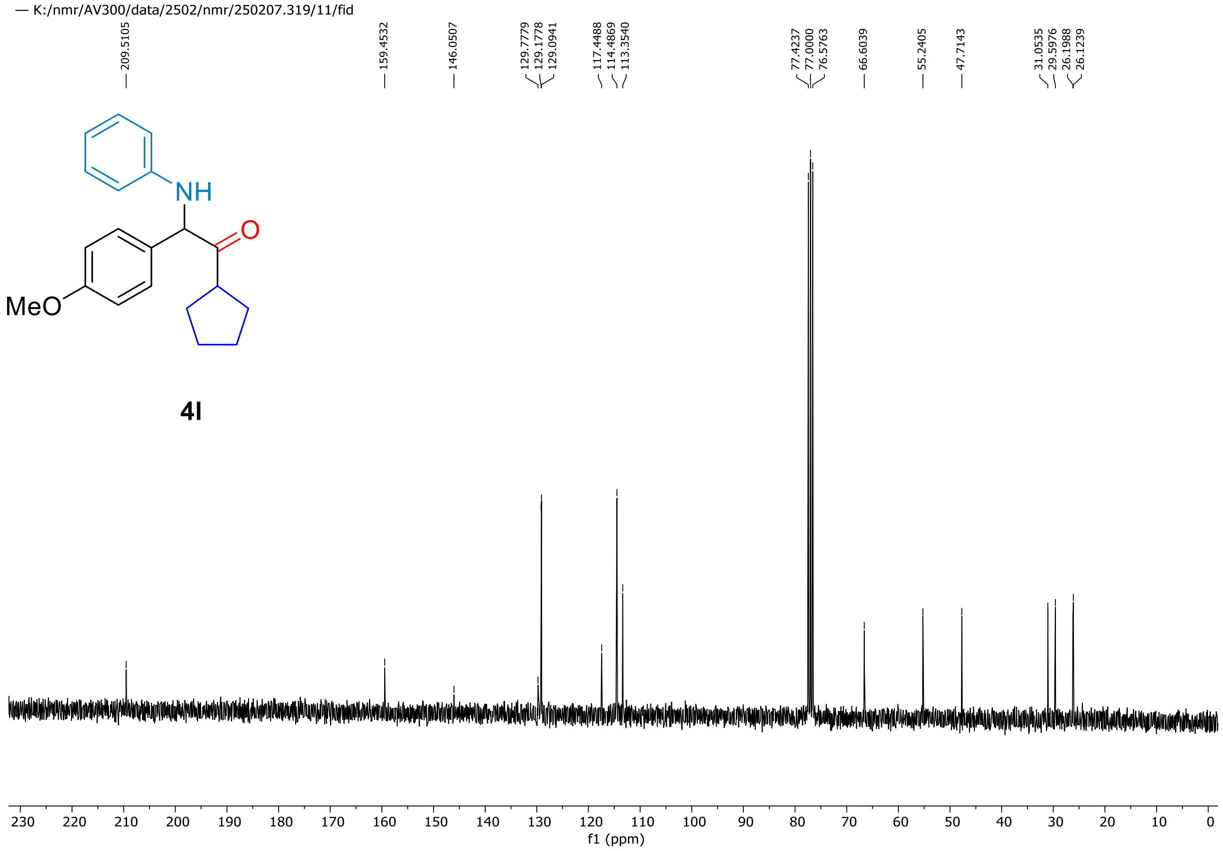

4m ^{1}H NMR (400 MHz, $CDCl_3$)

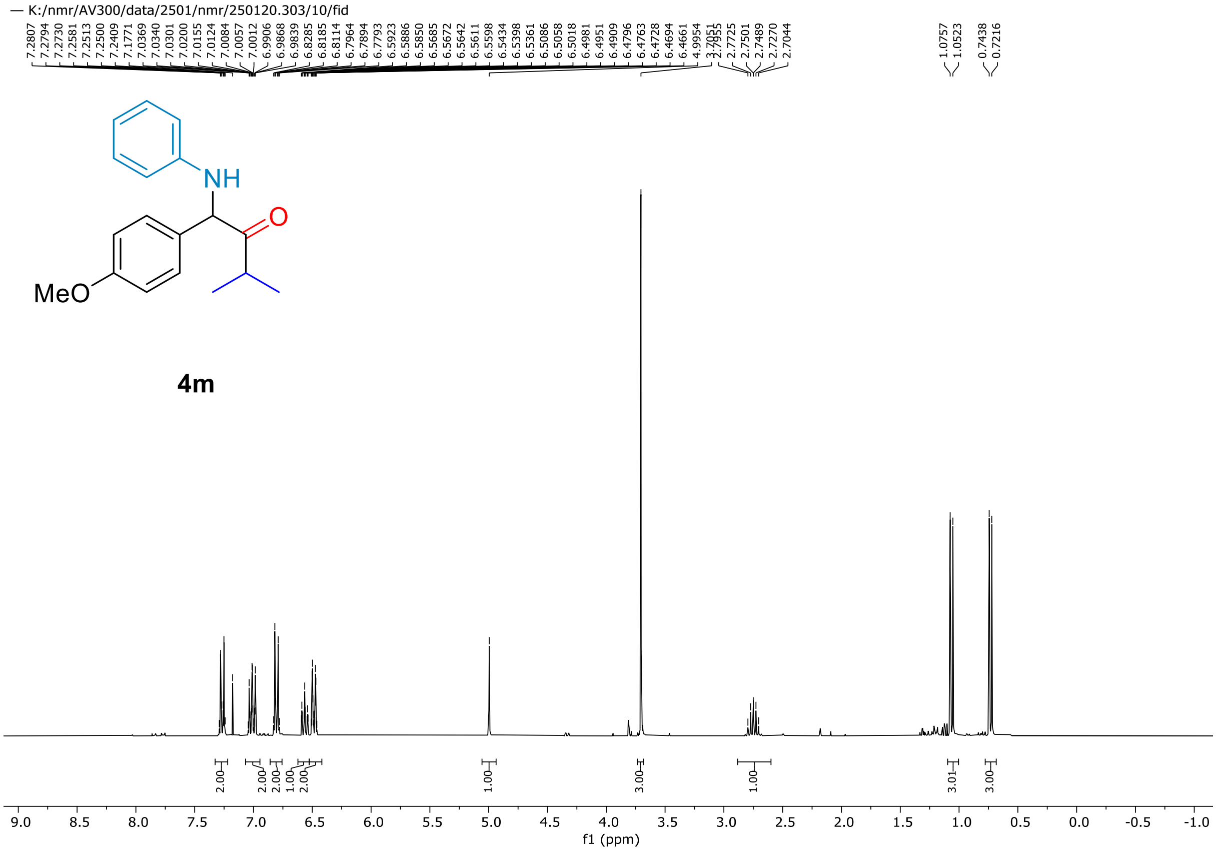

4m ^{13}C NMR (101 MHz, $CDCl_3$)

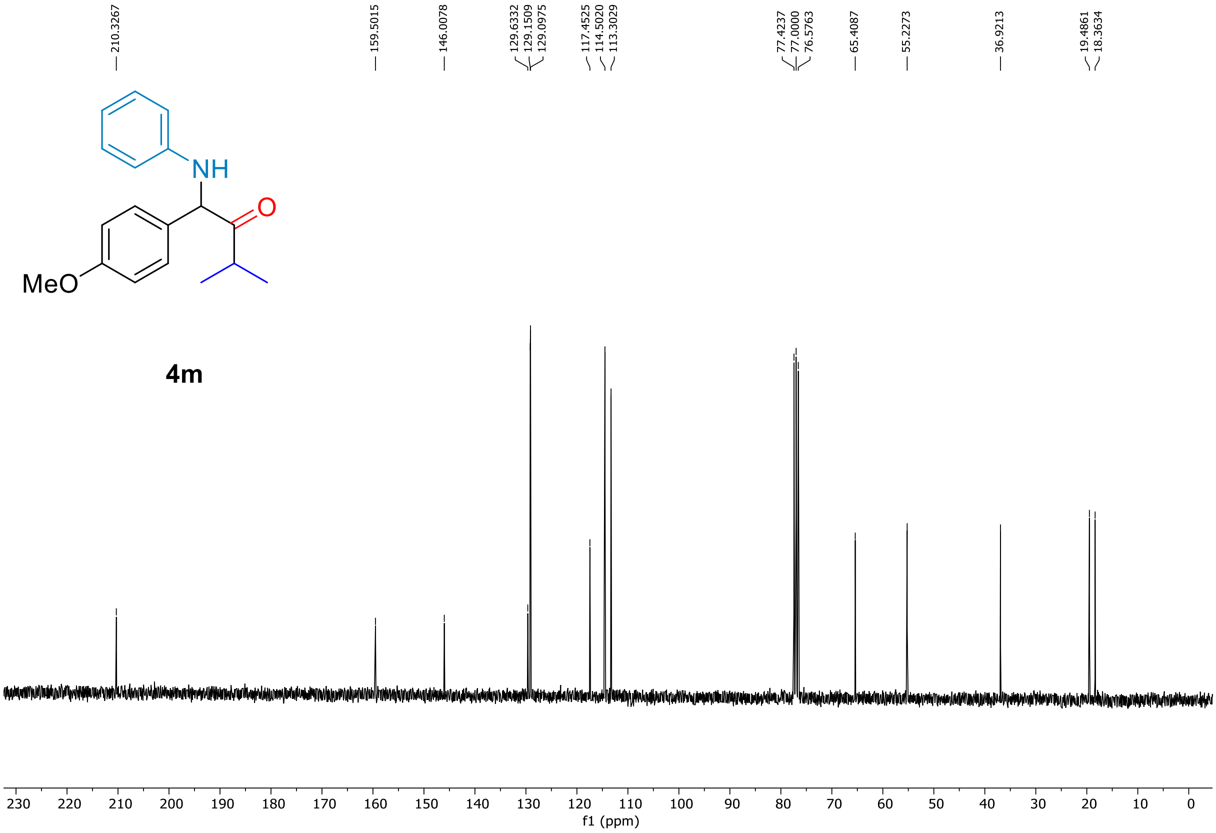

4n ^{1}H NMR (300 MHz, $CDCl_3$)

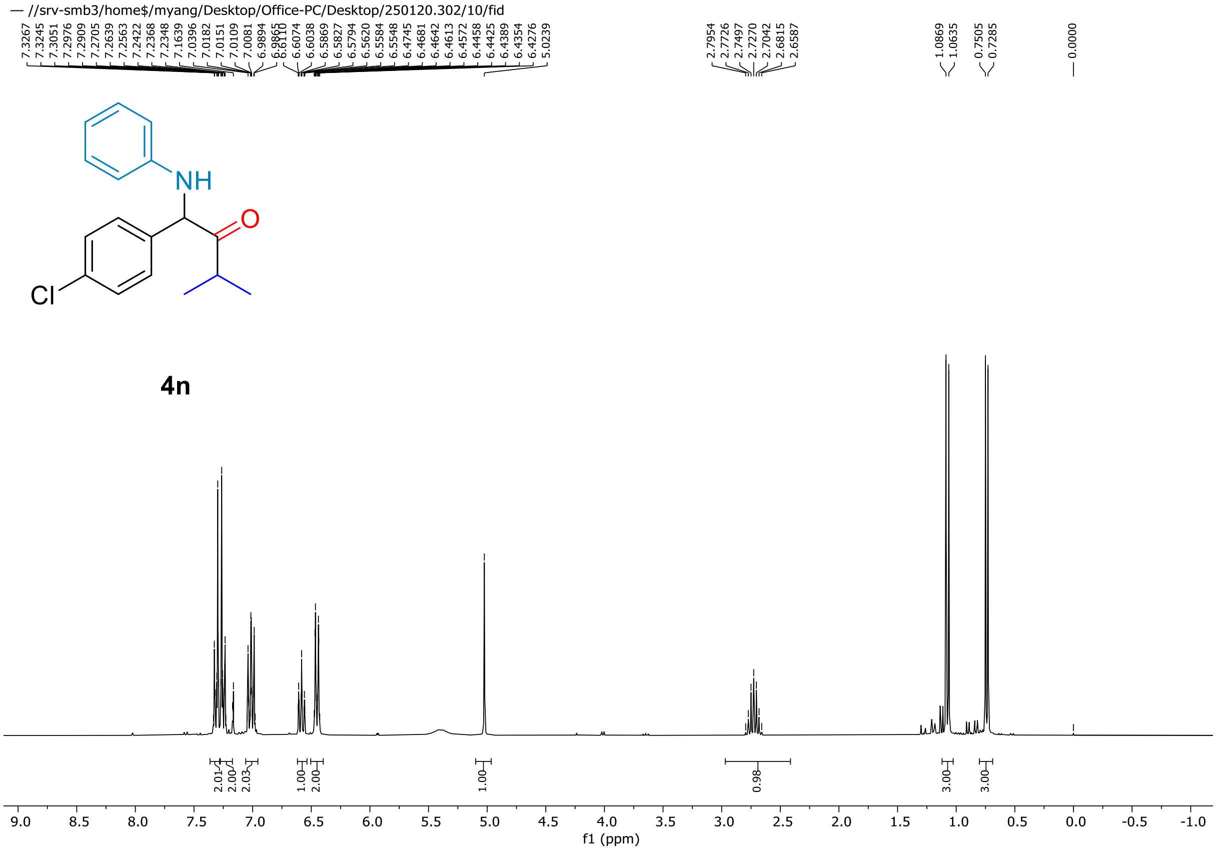

4n ^{13}C NMR (75 MHz, $CDCl_3$)

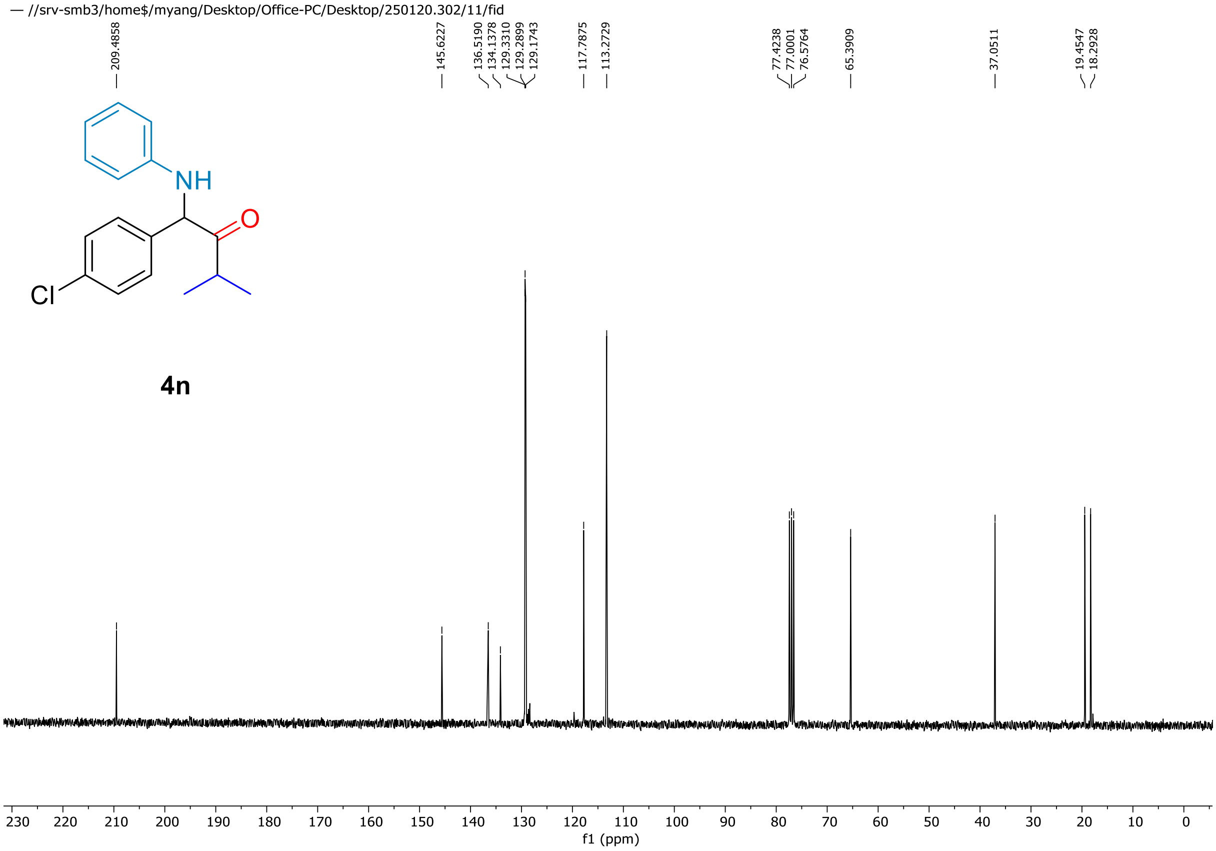

4o ^{1}H NMR (400 MHz, CDCl$_3$)

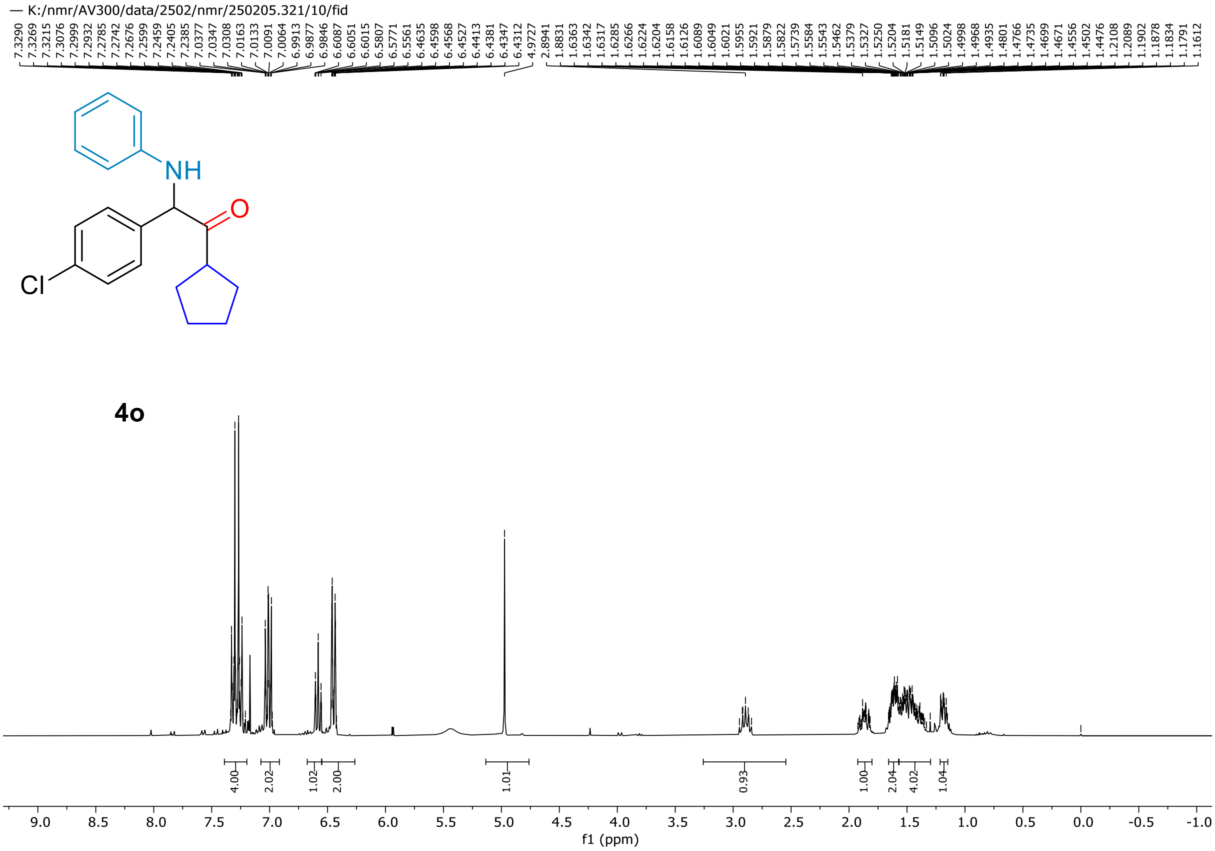

4o ^{13}C NMR (101 MHz, CDCl$_3$)

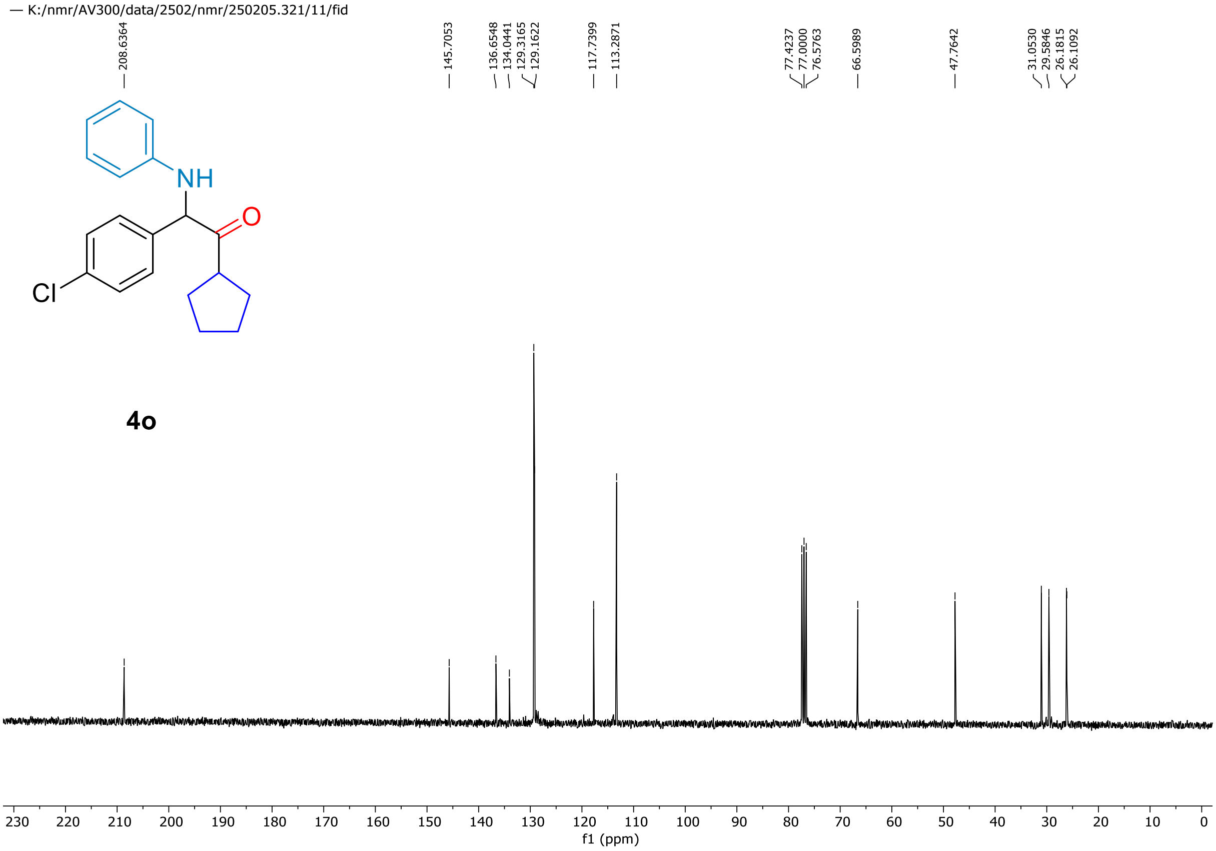

4p ^{1}H NMR (300 MHz, $CDCl_3$)

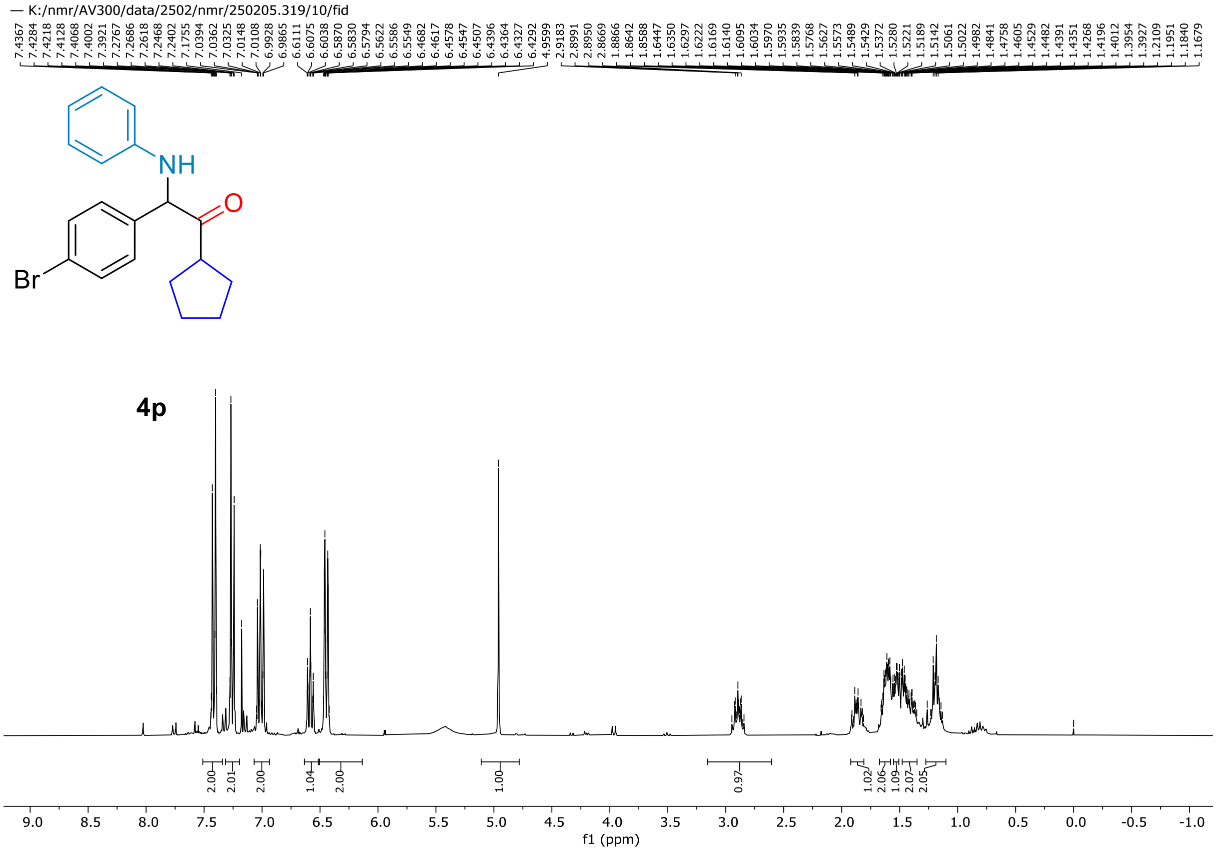

4p ^{13}C NMR (75 MHz, $CDCl_3$)

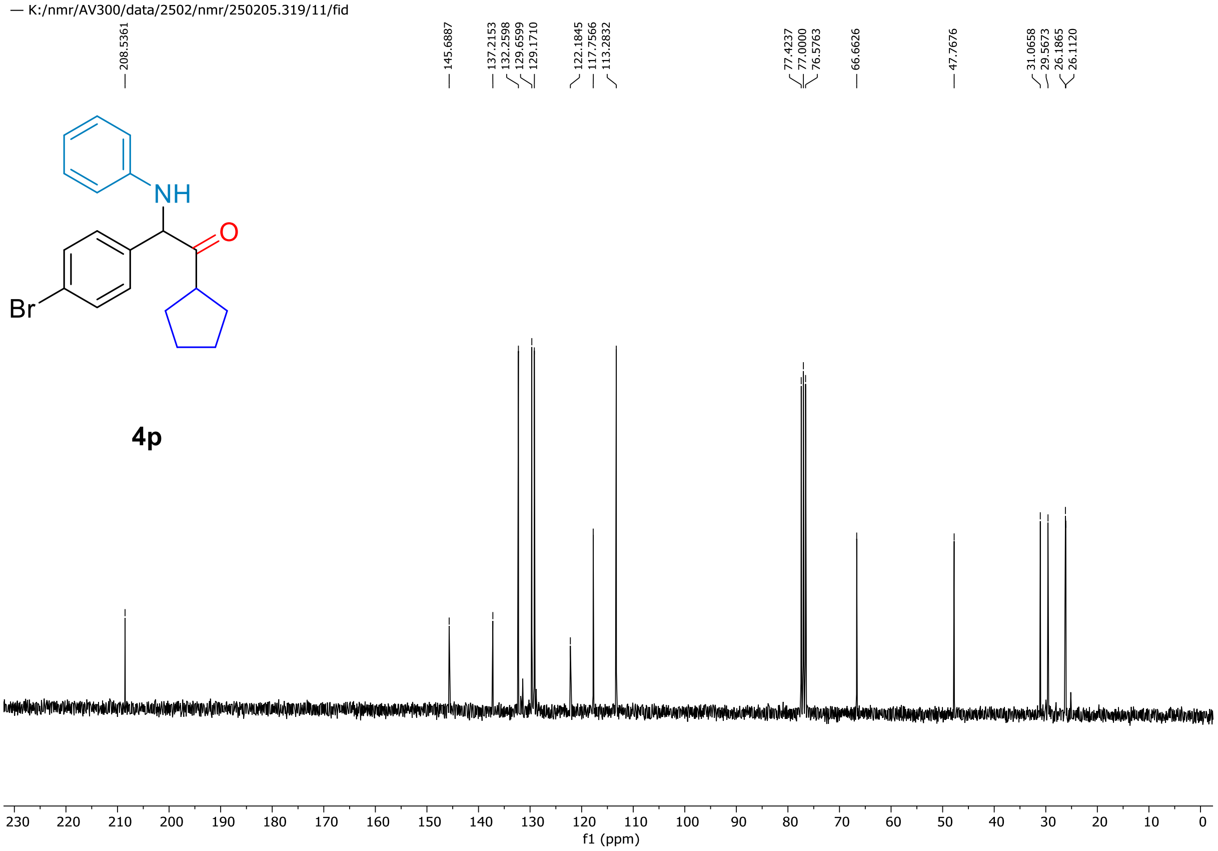

4q ^{1}H NMR (300 MHz, $CDCl_3$)

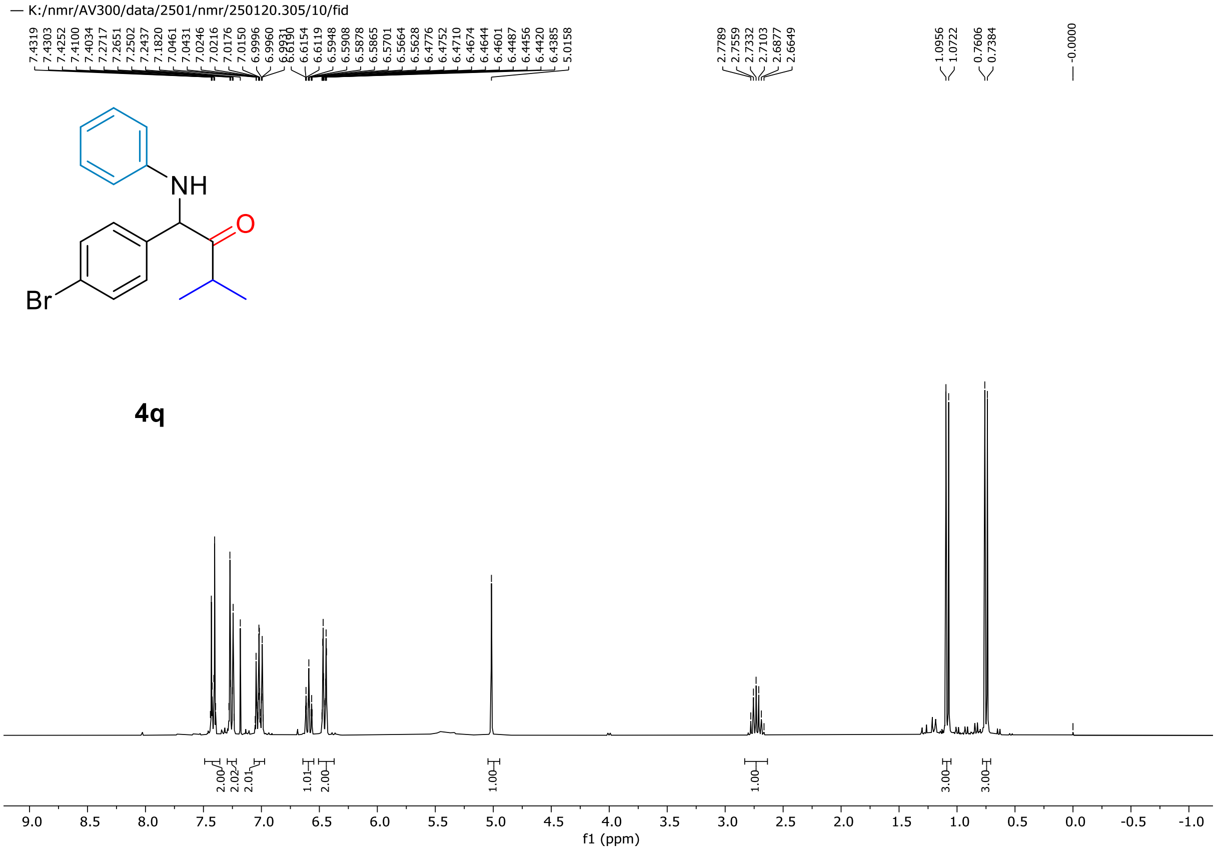

4q ^{13}C NMR (75 MHz, $CDCl_3$)

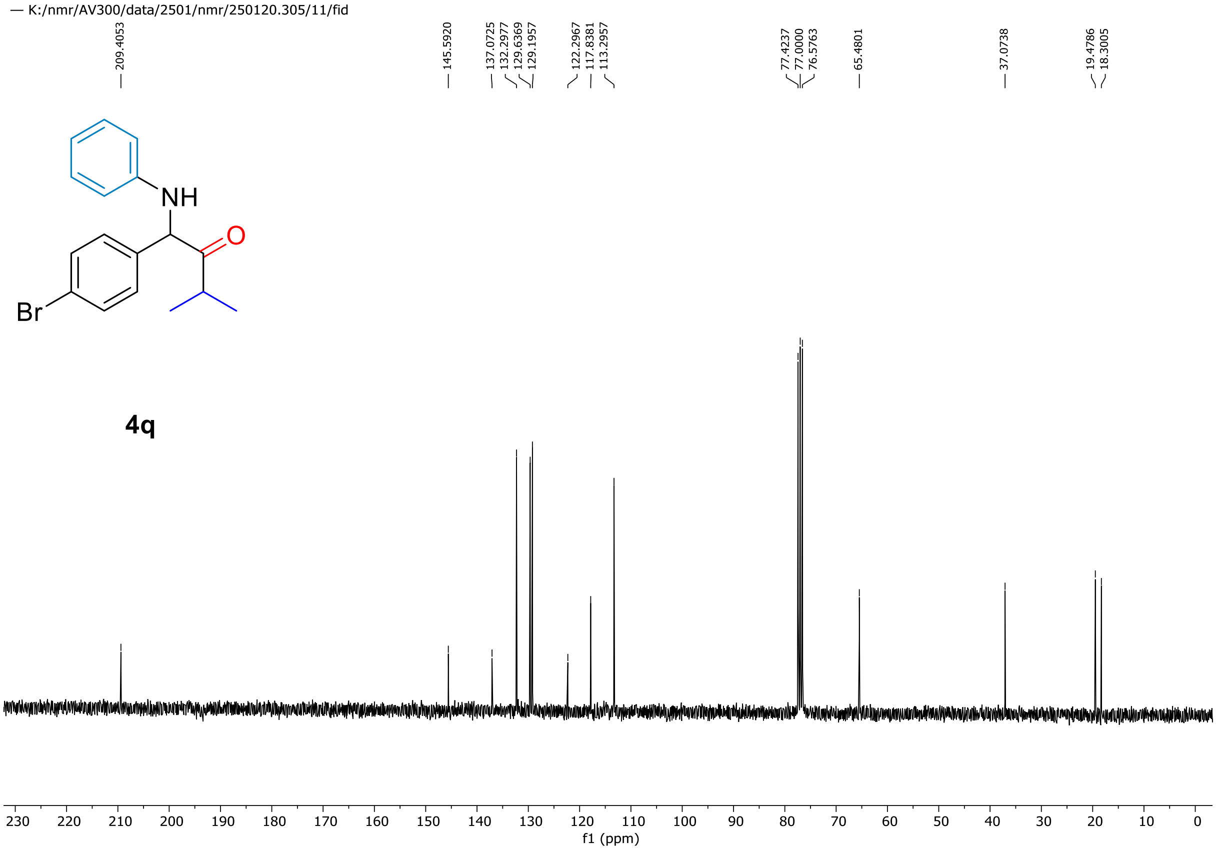

4r ^{1}H NMR (300 MHz, CDCl$_3$)

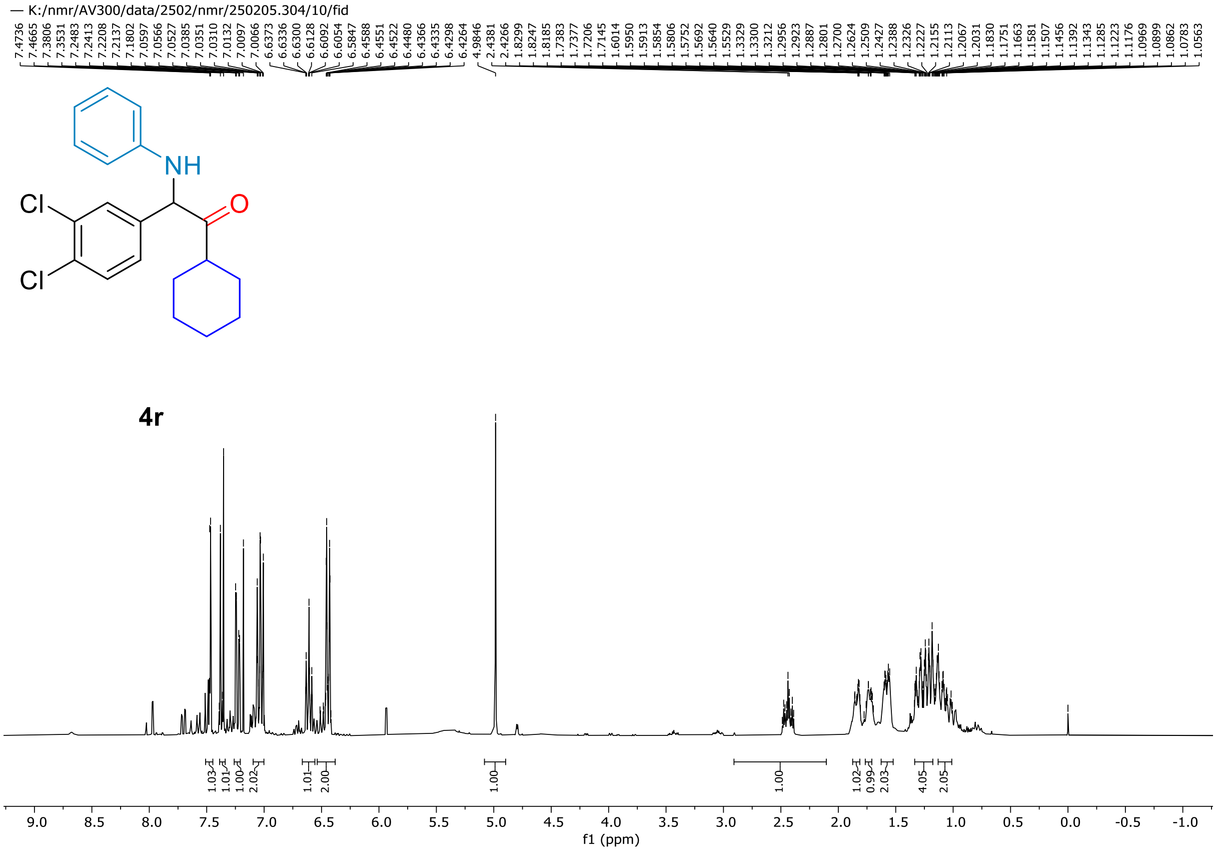

4r ^{13}C NMR (75 MHz, CDCl$_3$)

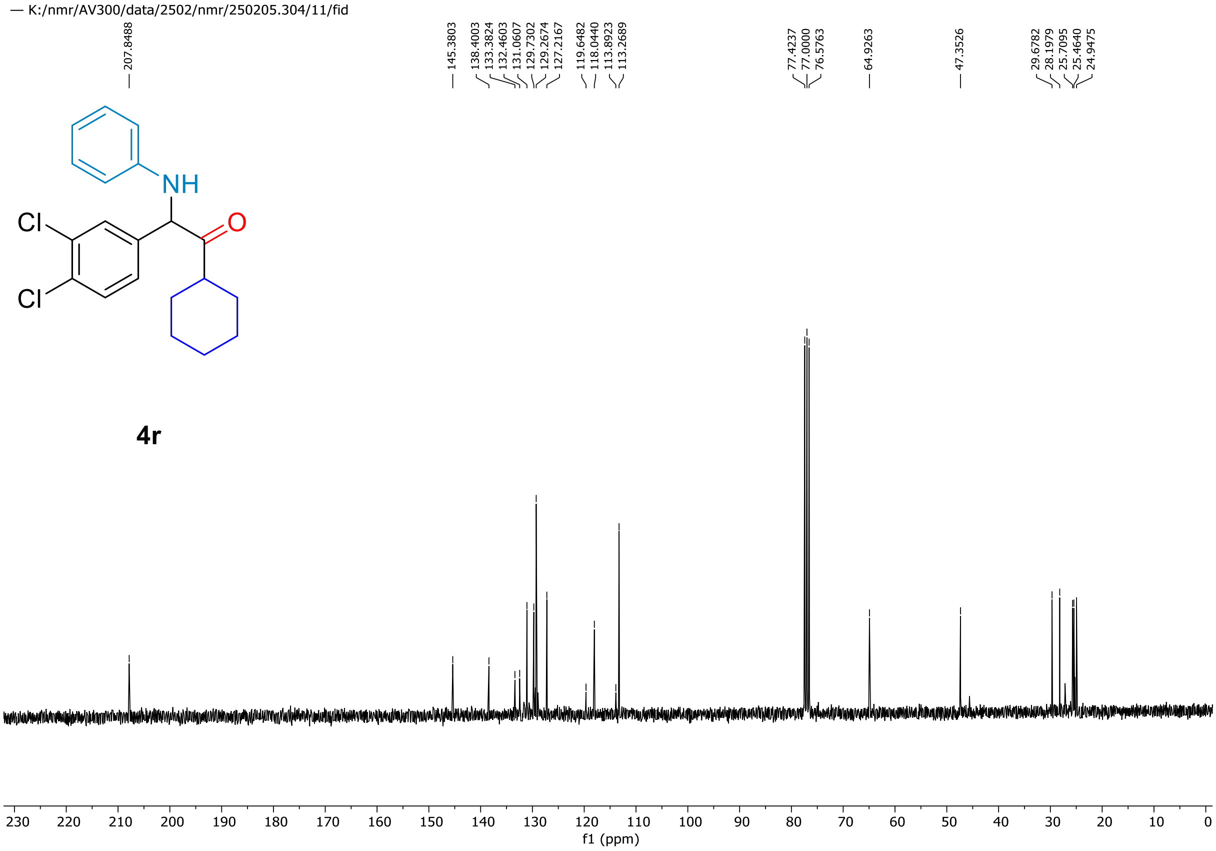

4s ^{1}H NMR (300 MHz, $CDCl_3$)

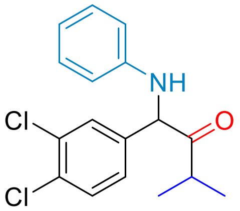

4s

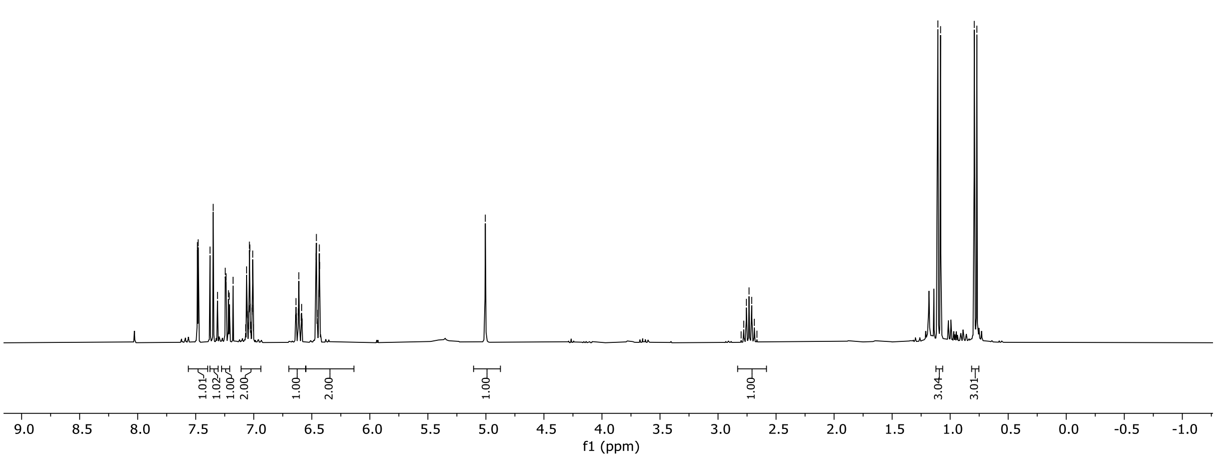

4s ^{13}C NMR (75 MHz, $CDCl_3$)

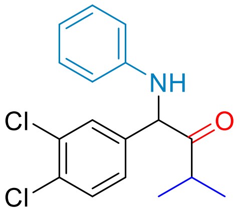

4s

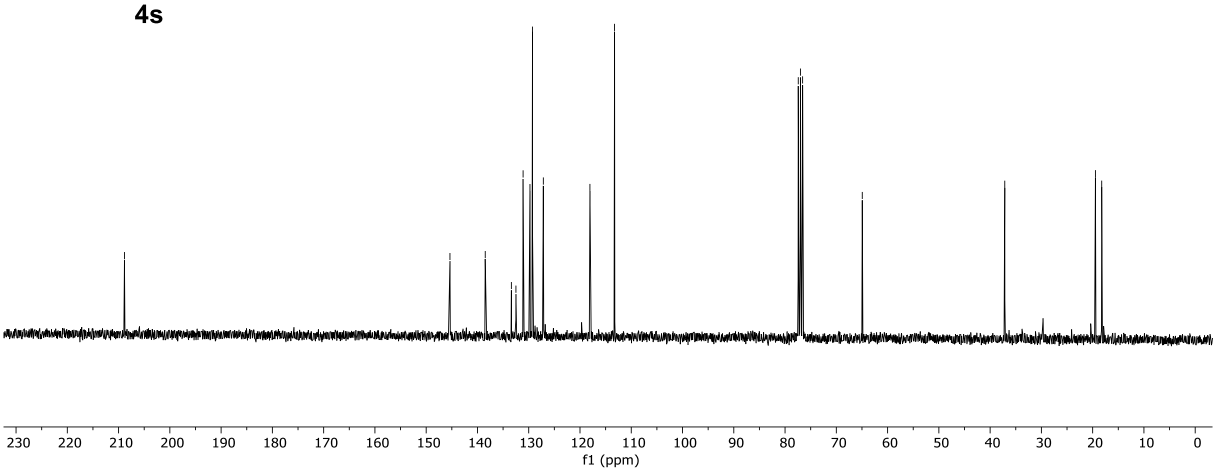

4t ^{1}H NMR (300 MHz, CDCl$_3$)

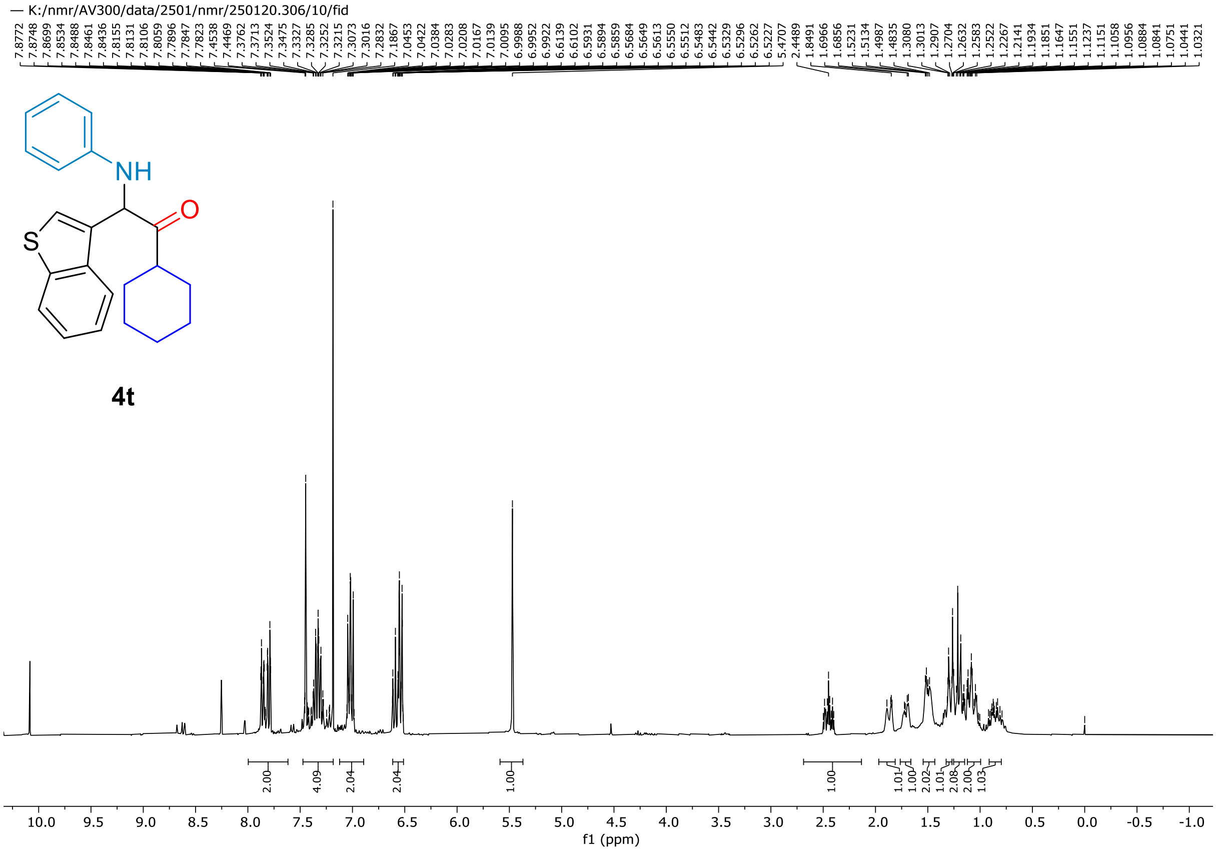

4t ^{13}C NMR (75 MHz, CDCl$_3$)

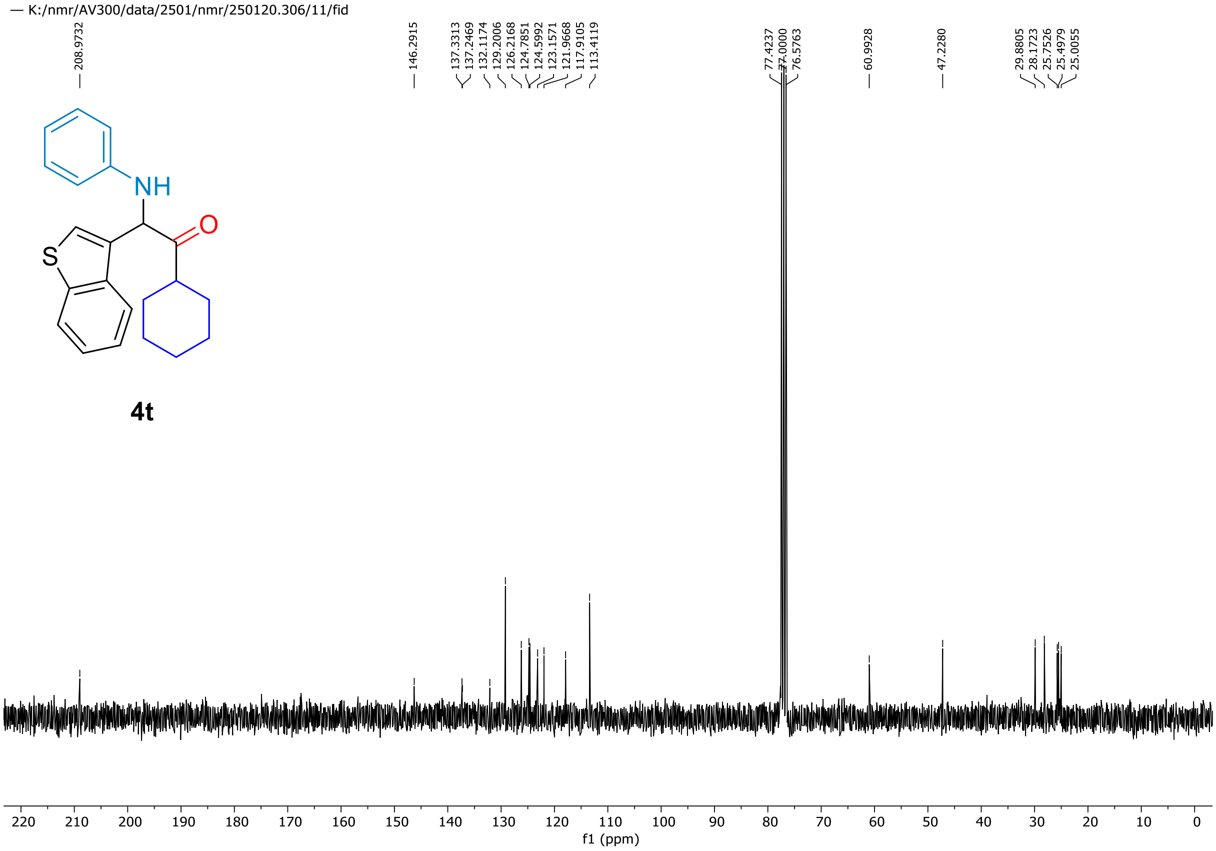

4u ^{1}H NMR (300 MHz, $CDCl_3$)

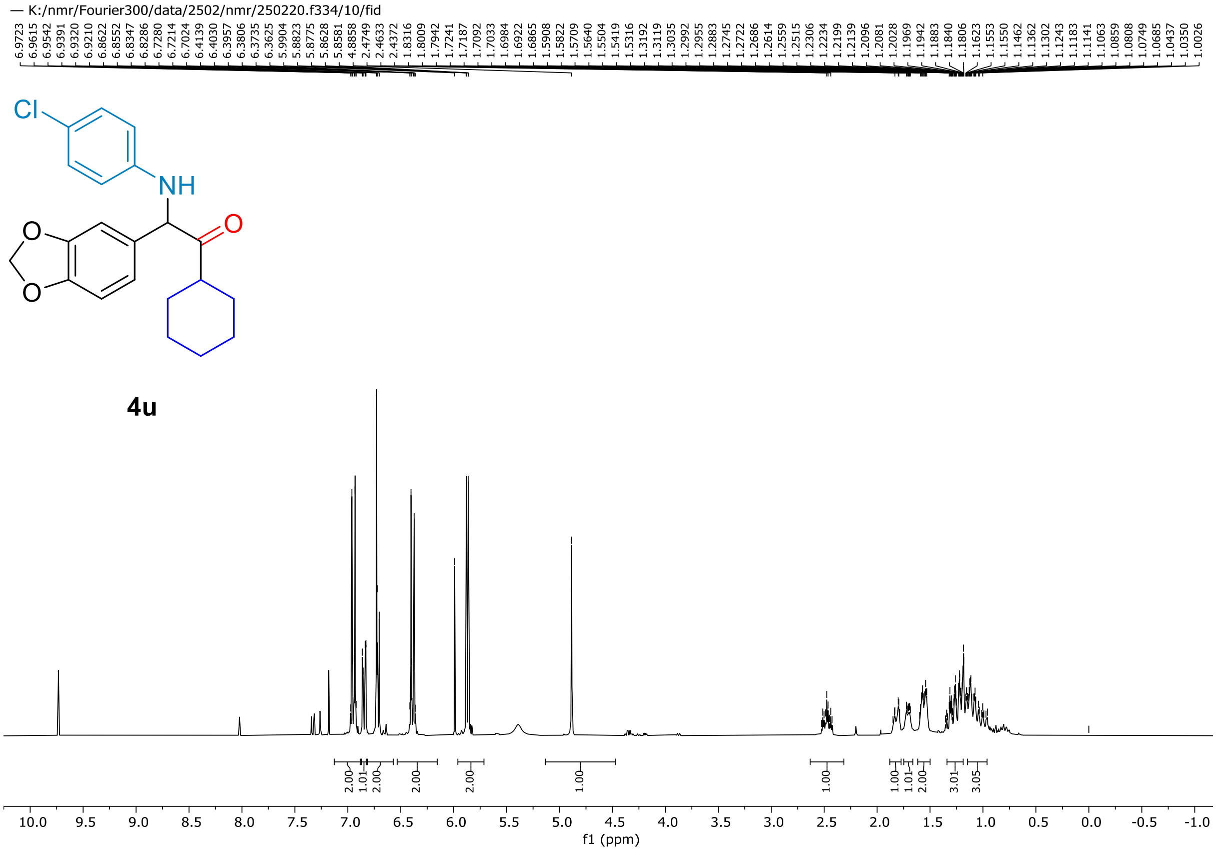

4u ^{13}C NMR (75 MHz, $CDCl_3$)

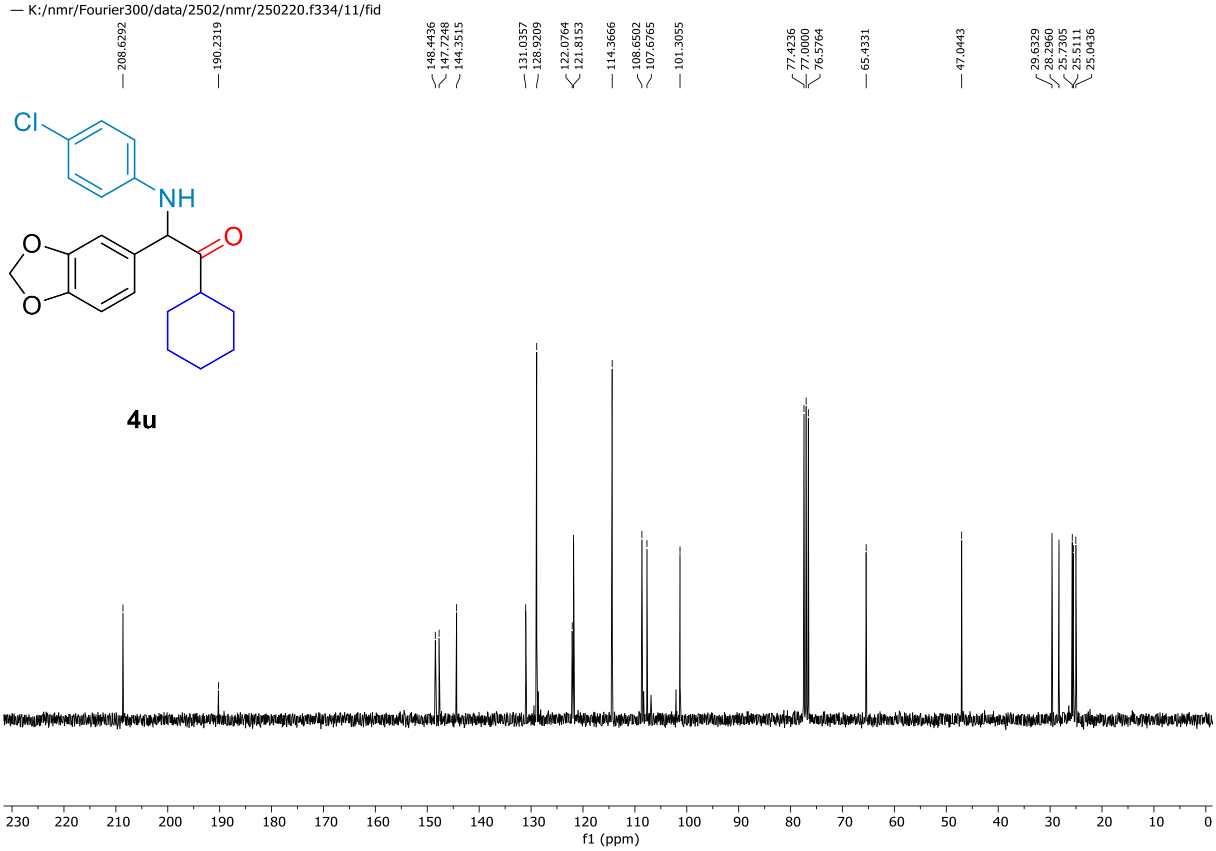

4v ^{1}H NMR (400 MHz, $CDCl_3$)

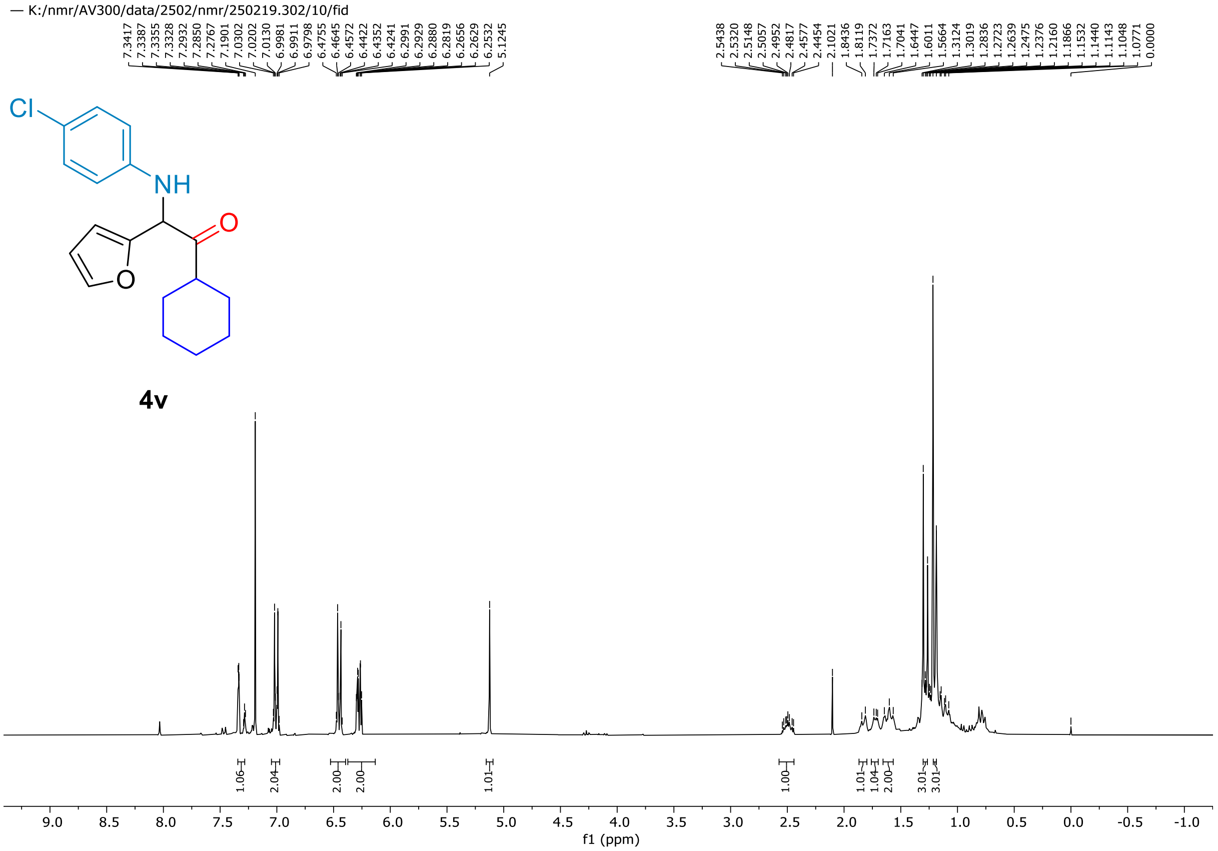

4v ^{13}C NMR (101 MHz, $CDCl_3$)

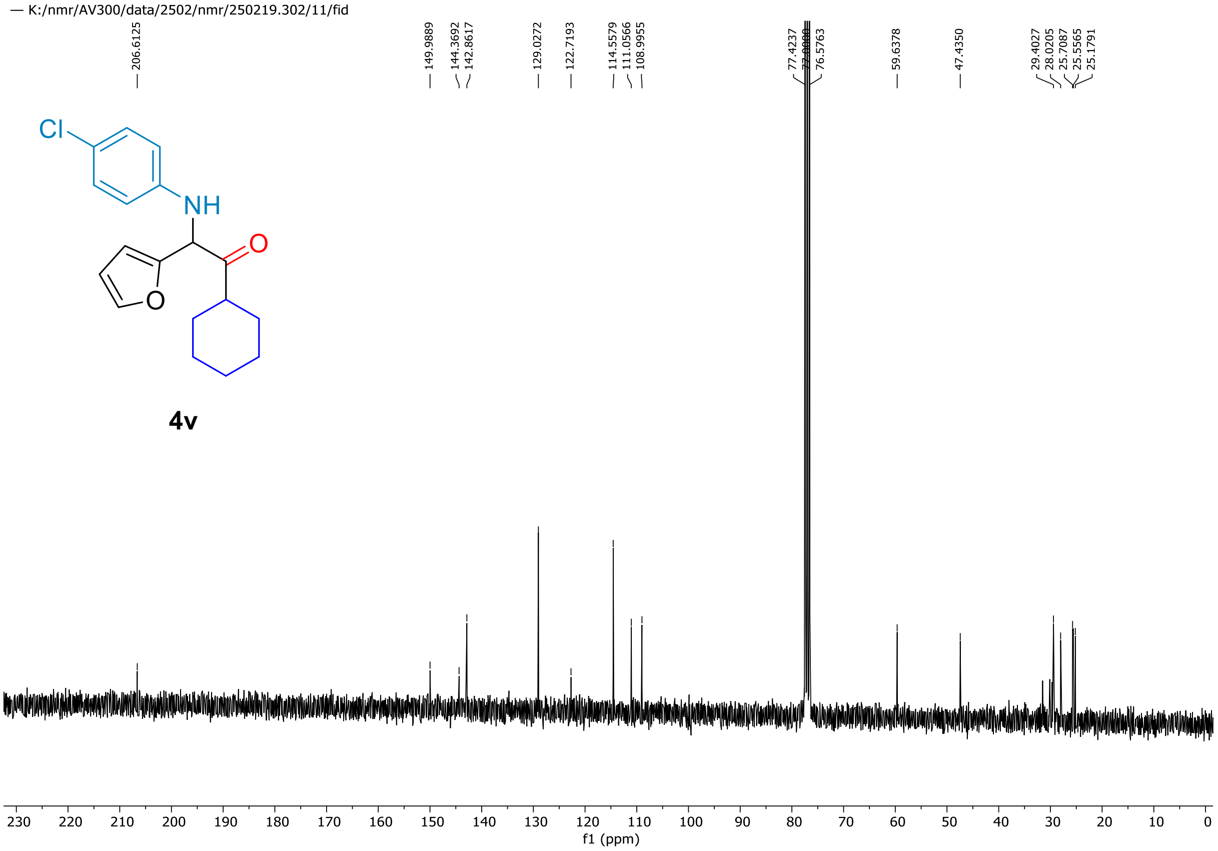

4w ^{1}H NMR (300 MHz, $CDCl_3$)

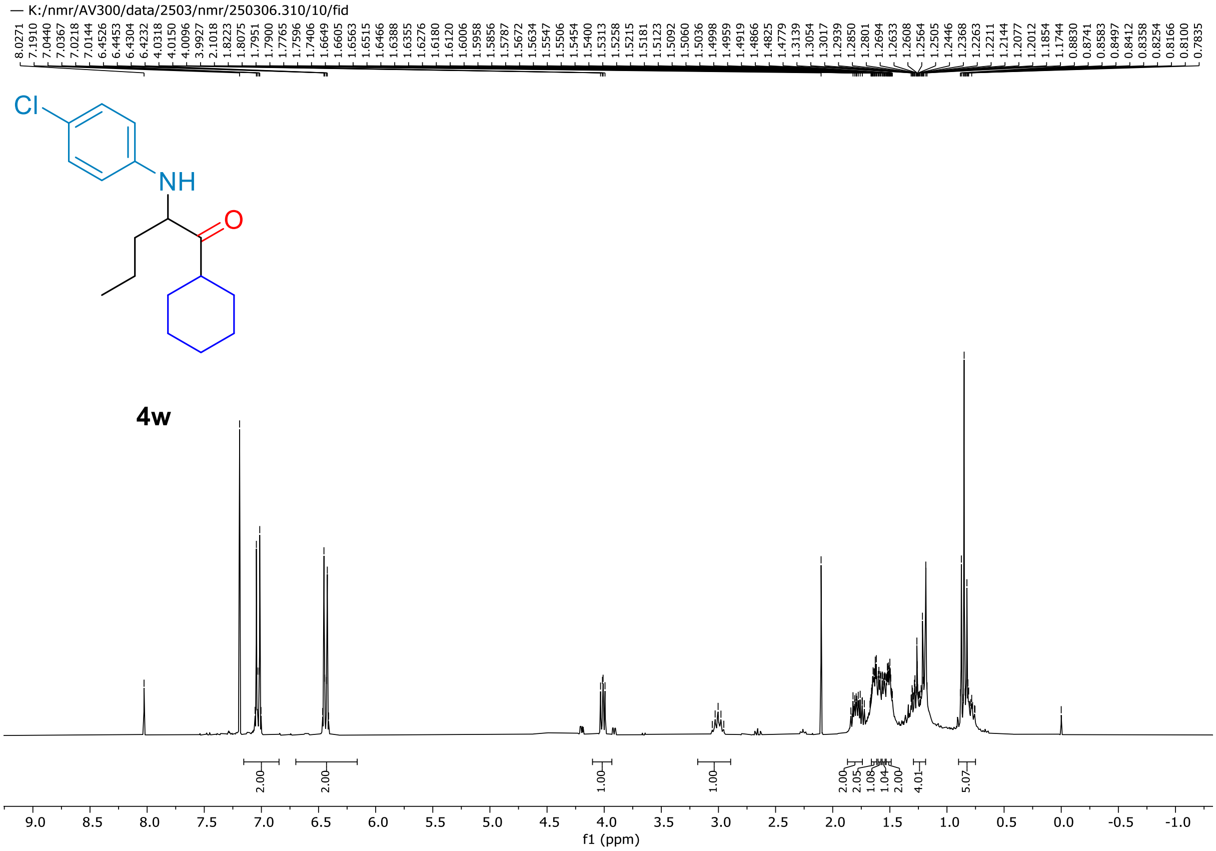

4w ^{13}C NMR (75 MHz, $CDCl_3$)

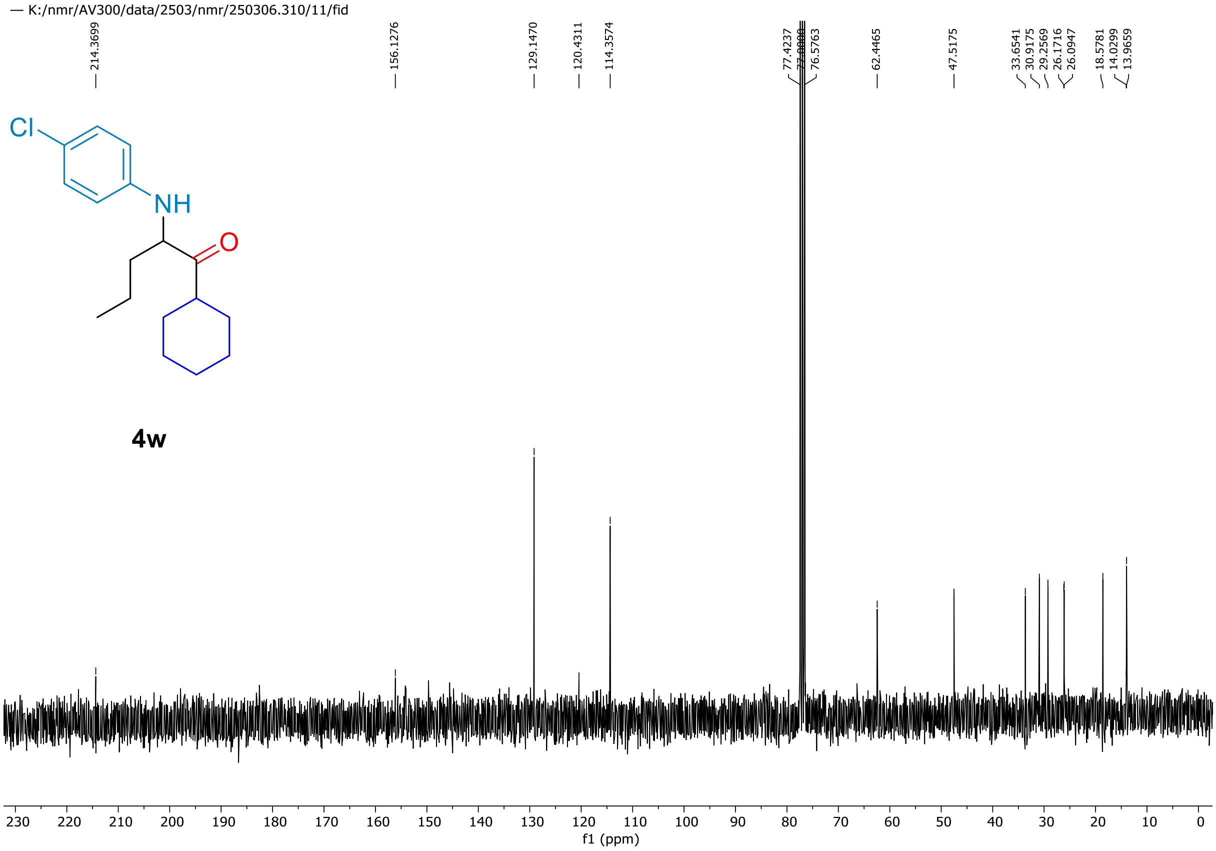

4x ^{1}H NMR (400 MHz, $CDCl_3$)

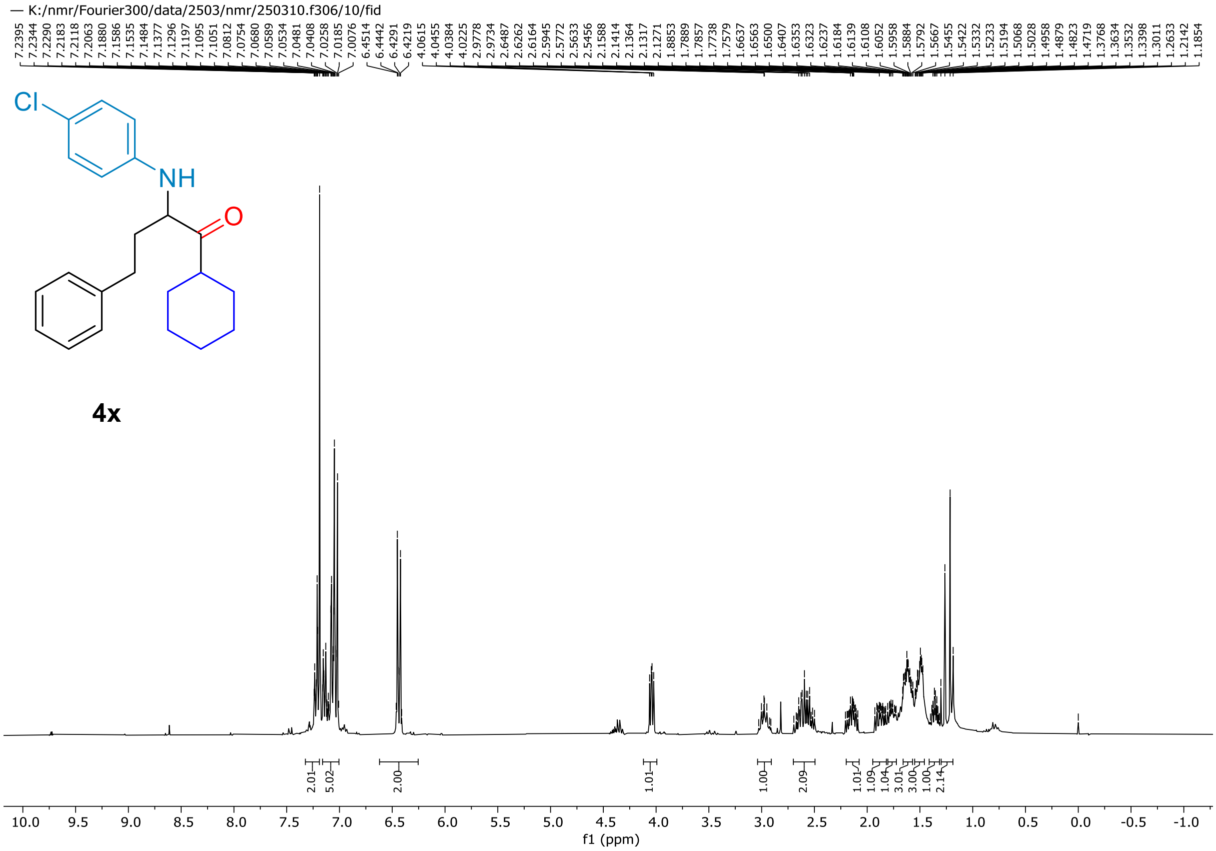

4x ^{13}C NMR (101 MHz, $CDCl_3$)

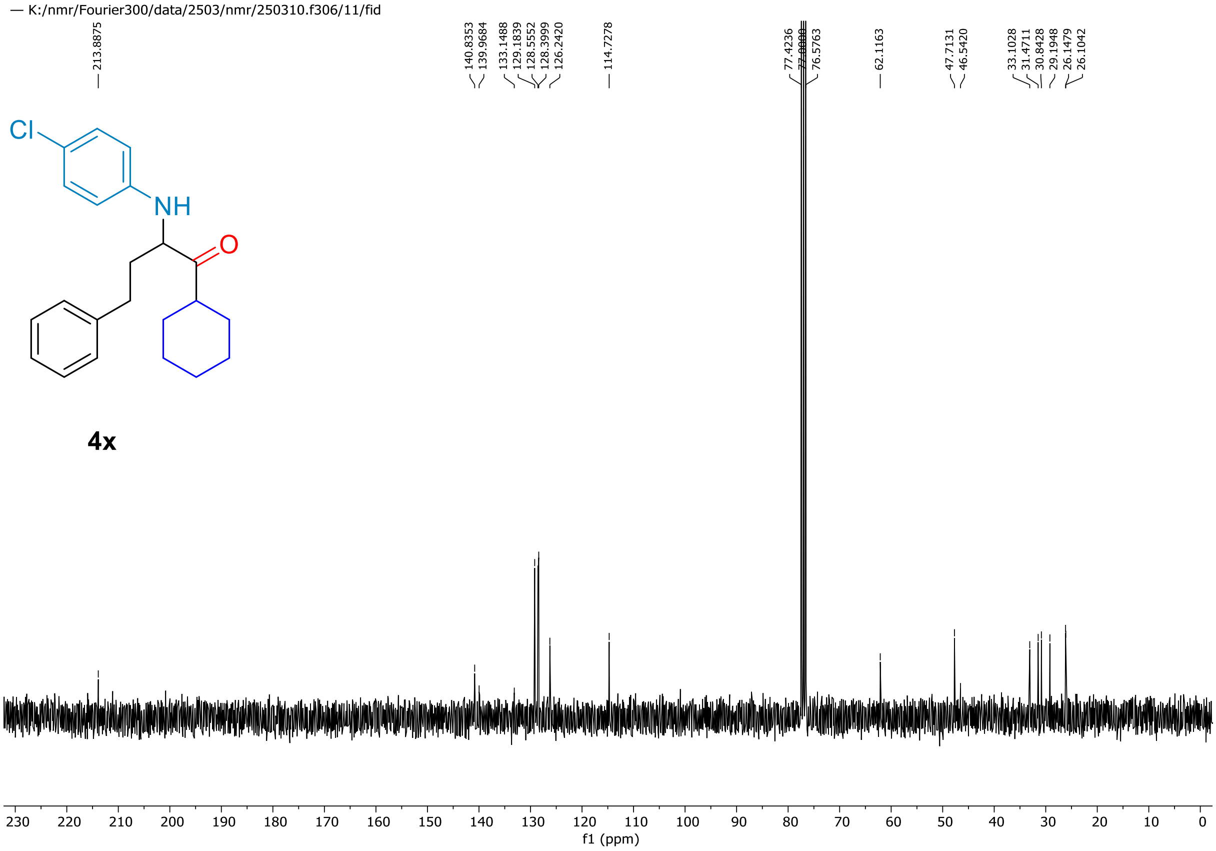

4y ^{1}H NMR (400 MHz, $CDCl_3$)

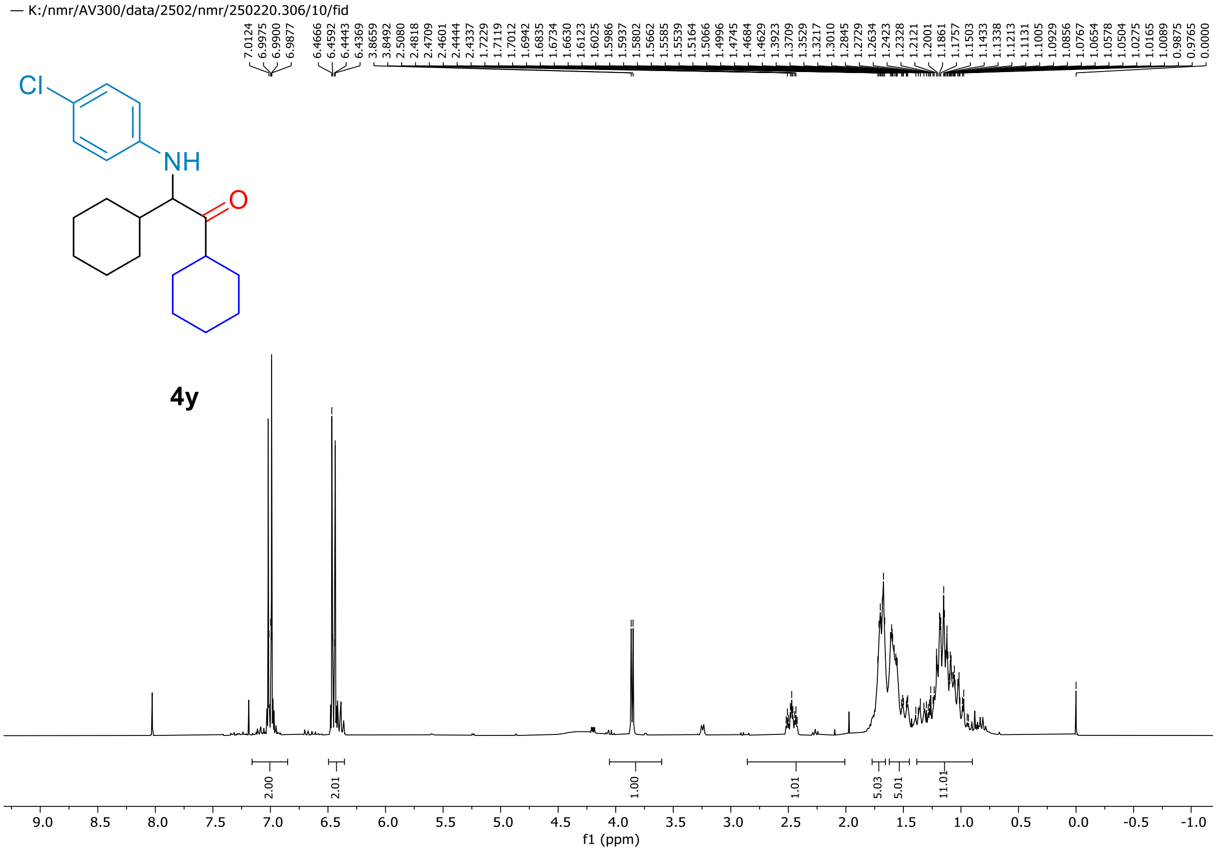

4y ^{13}C NMR (101 MHz, $CDCl_3$)

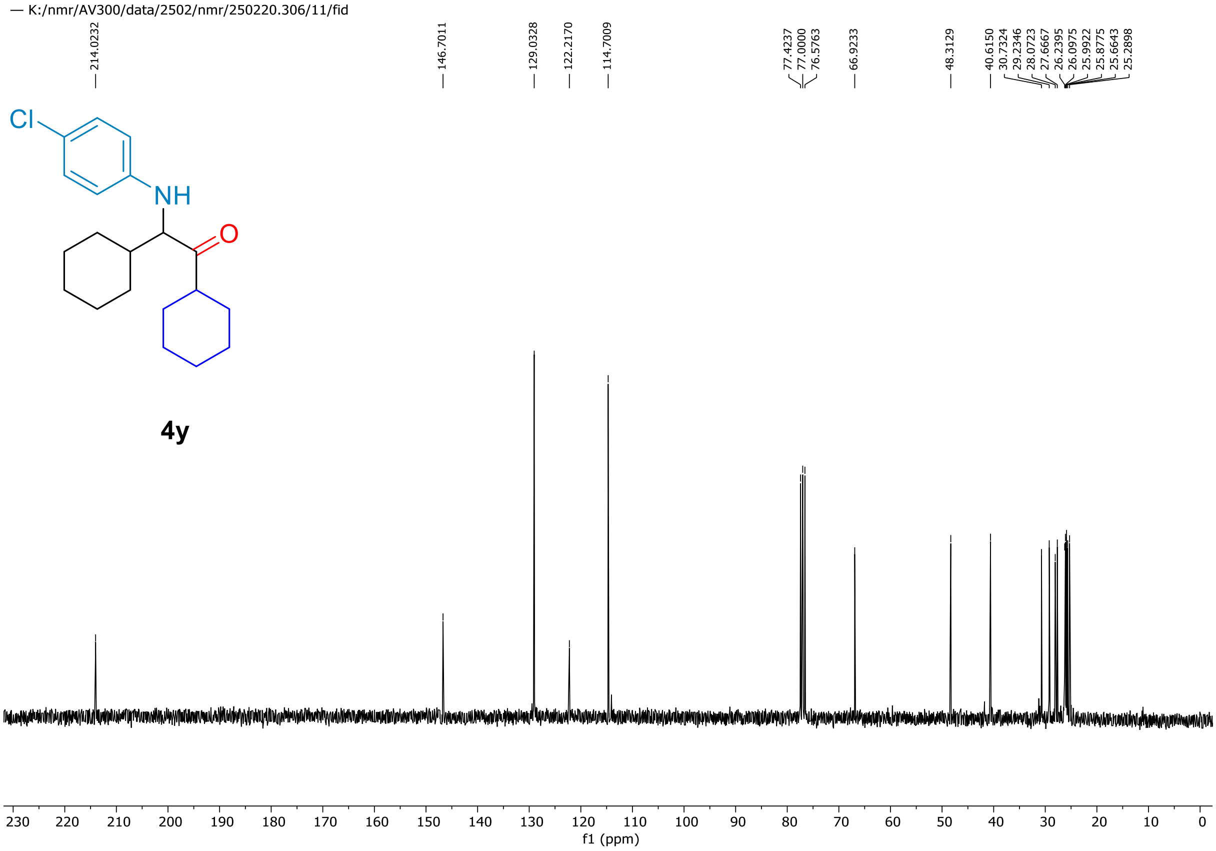

4z ^{1}H NMR (400 MHz, $CDCl_3$)

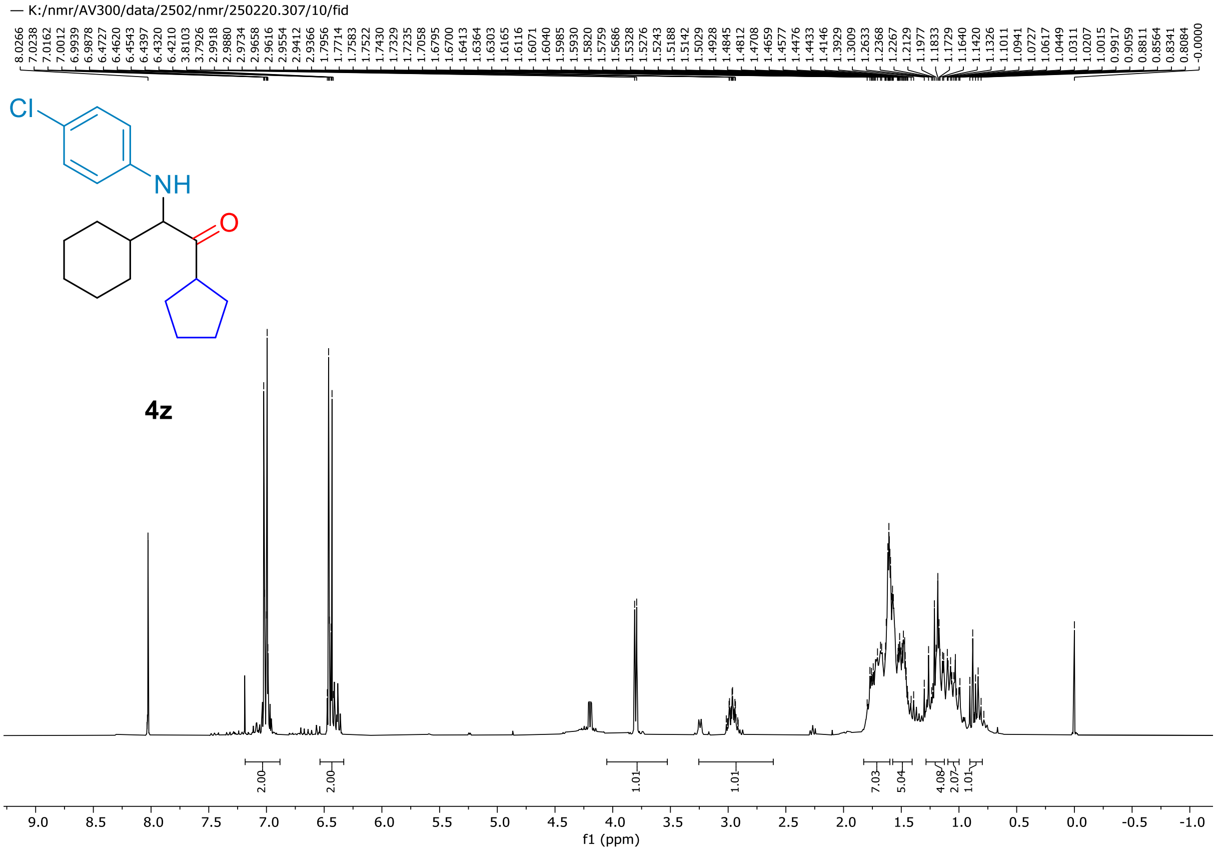

4z ^{13}C NMR (101 MHz, $CDCl_3$)

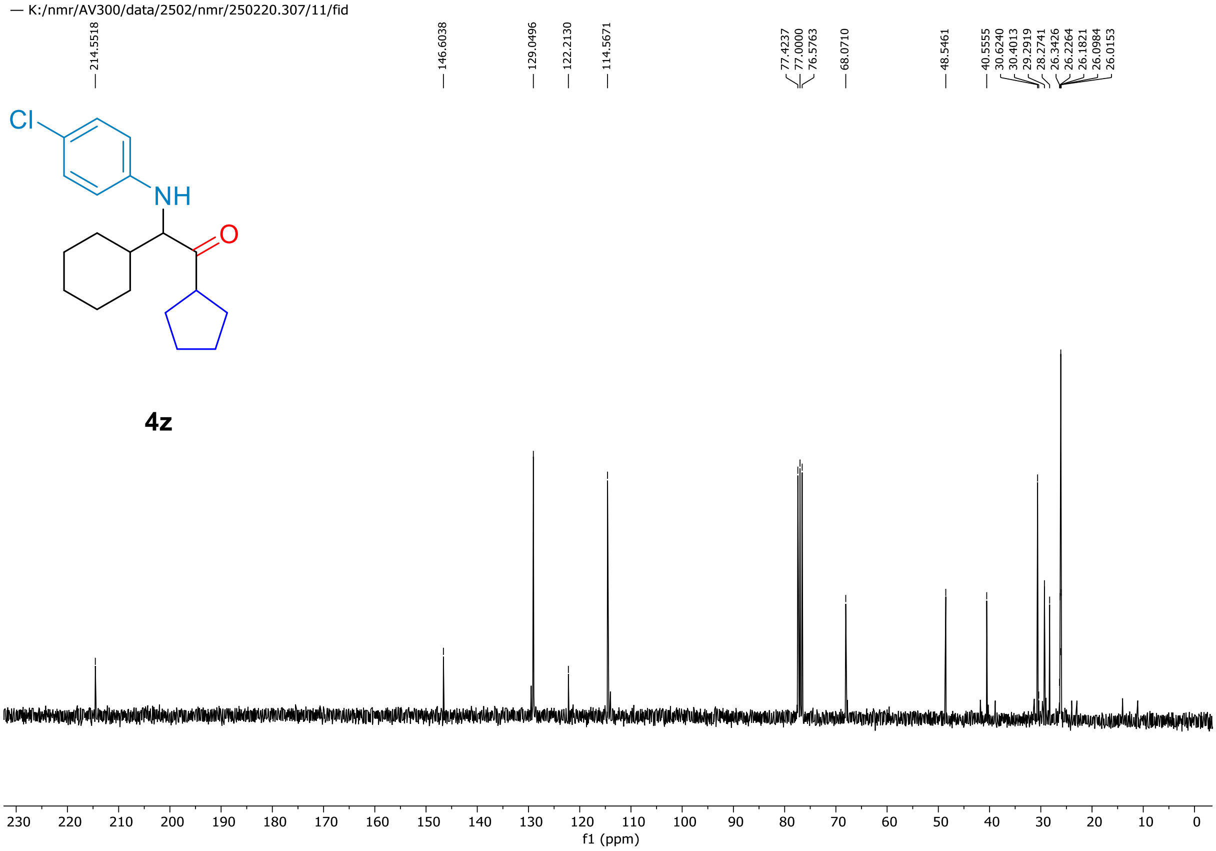

4aa ^{1}H NMR (400 MHz, $CDCl_3$)

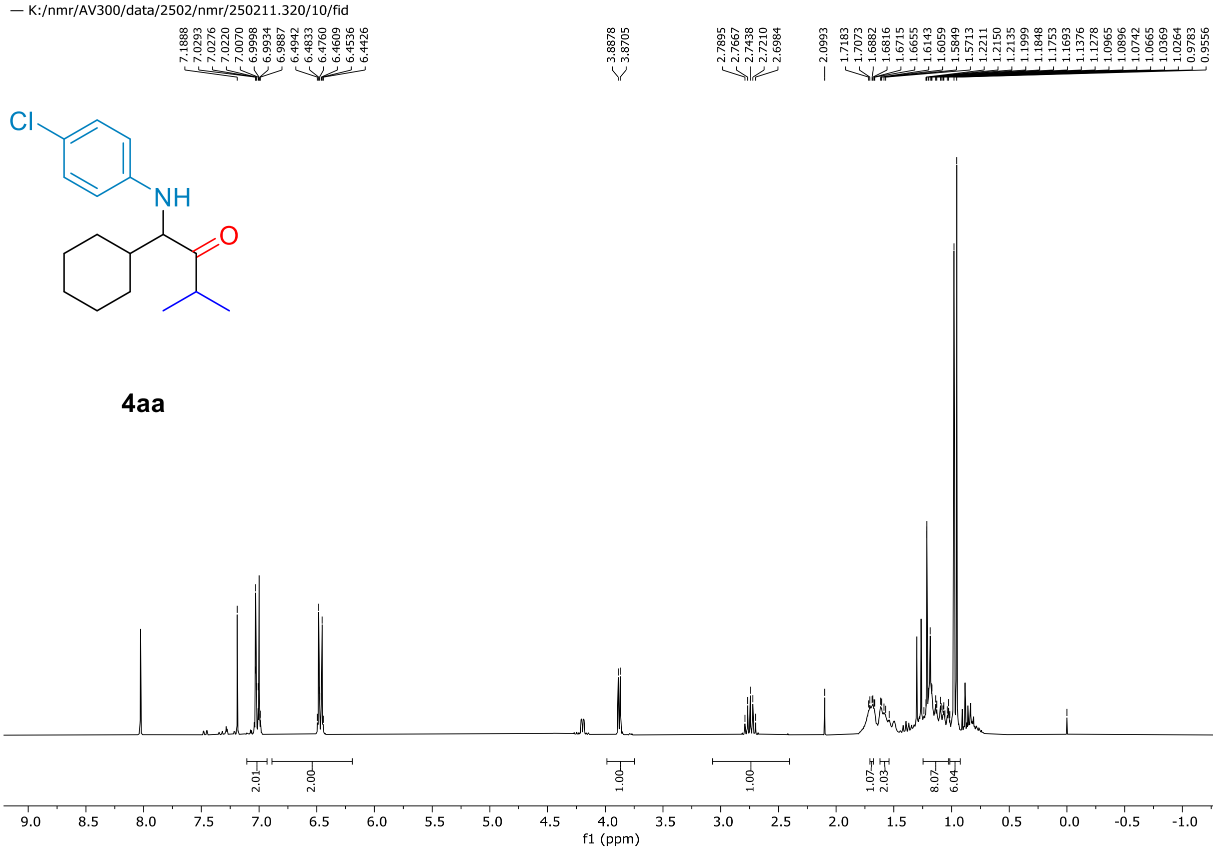

4aa ^{13}C NMR (101 MHz, $CDCl_3$)

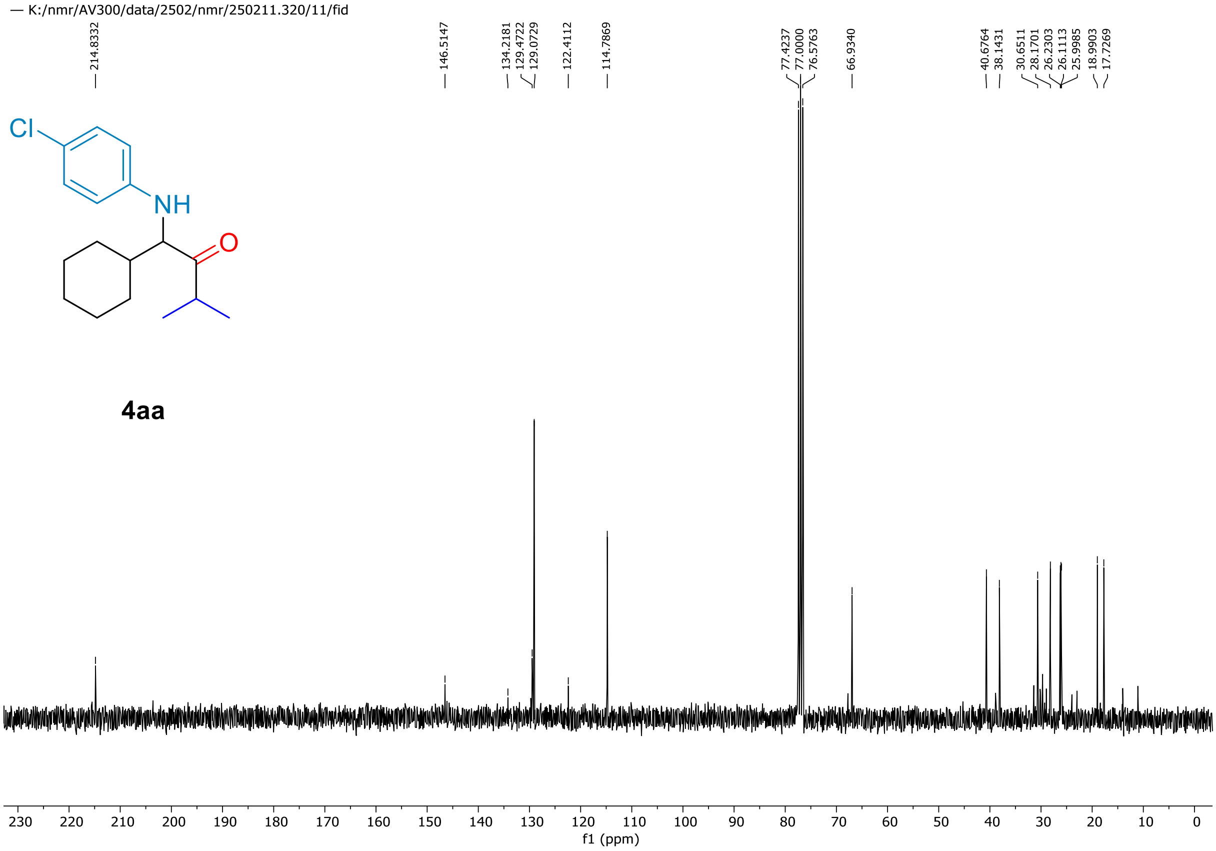

4ab ^{1}H NMR (400 MHz, $CDCl_3$)

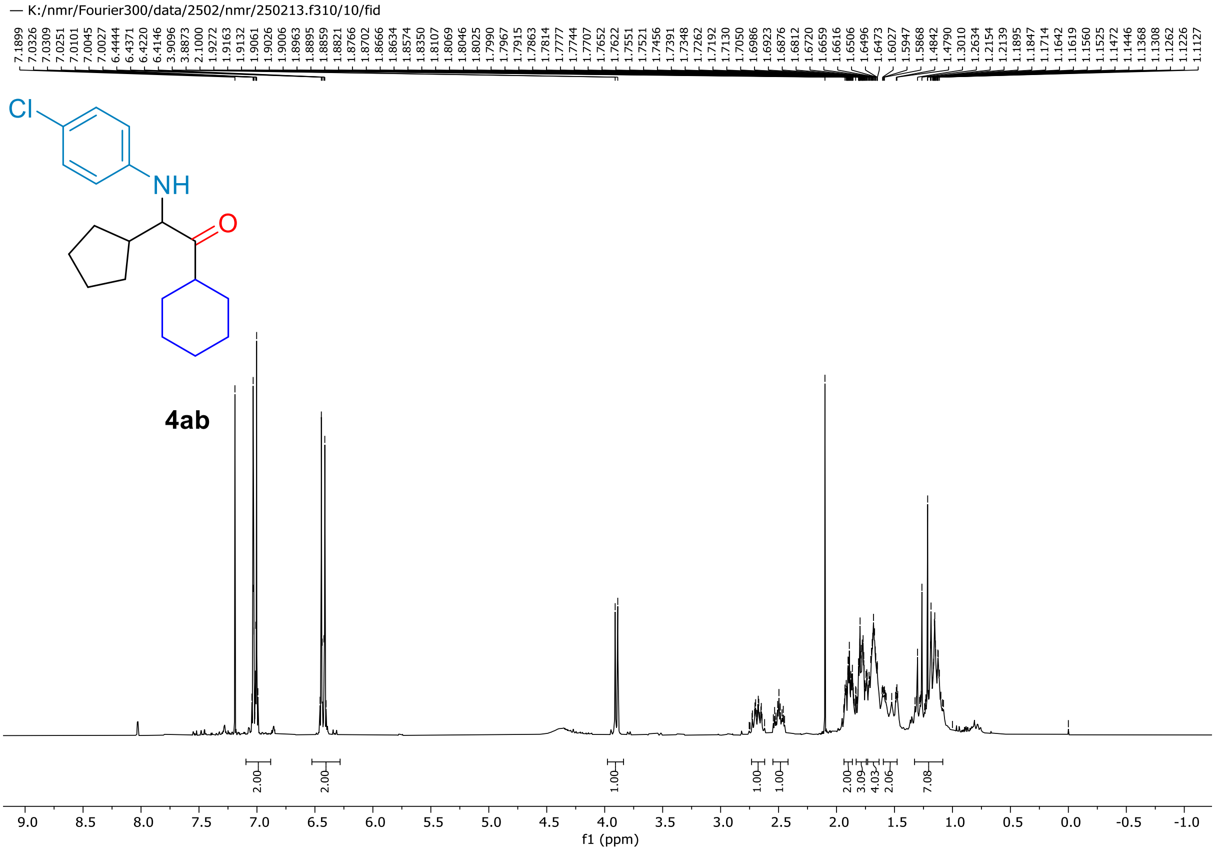

4ab ^{13}C NMR (101 MHz, $CDCl_3$)

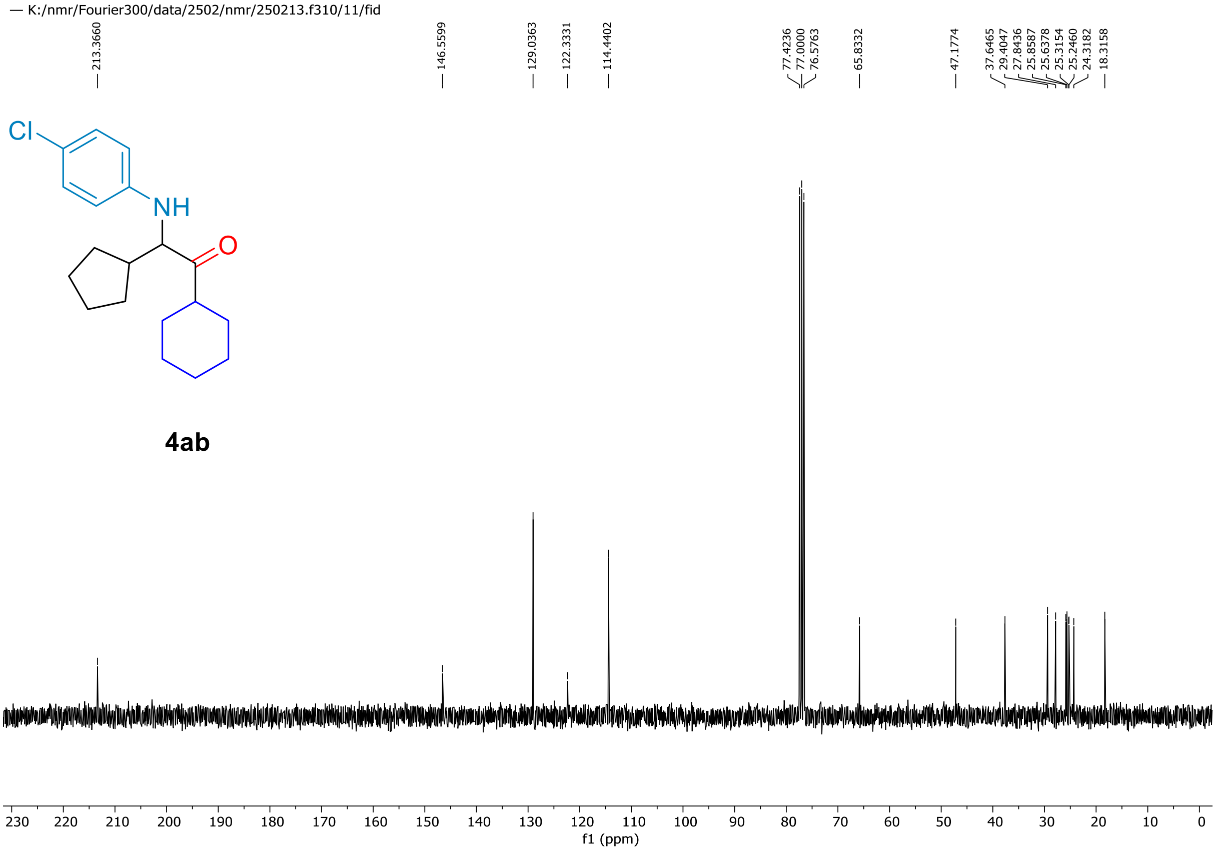

4ac ^{1}H NMR (300 MHz, $CDCl_3$)

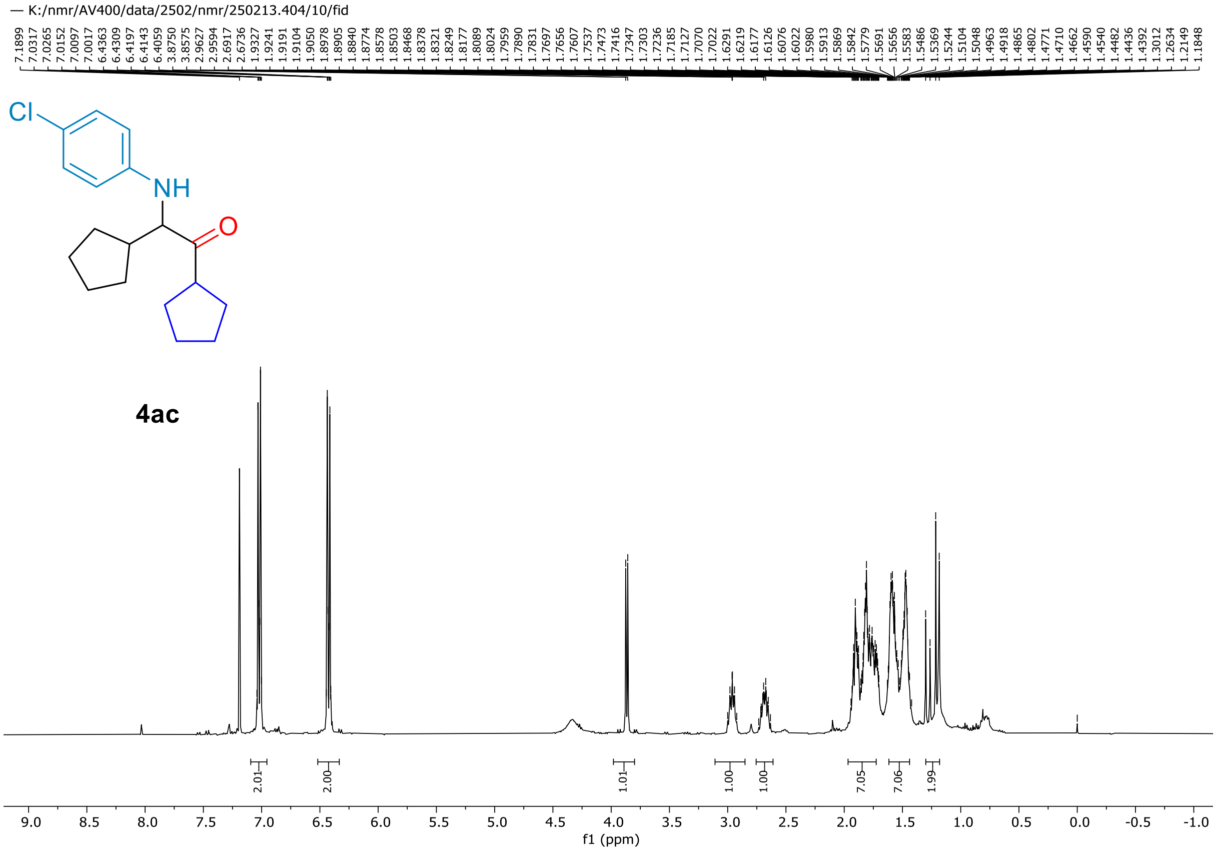

4ac ^{13}C NMR (75 MHz, $CDCl_3$)

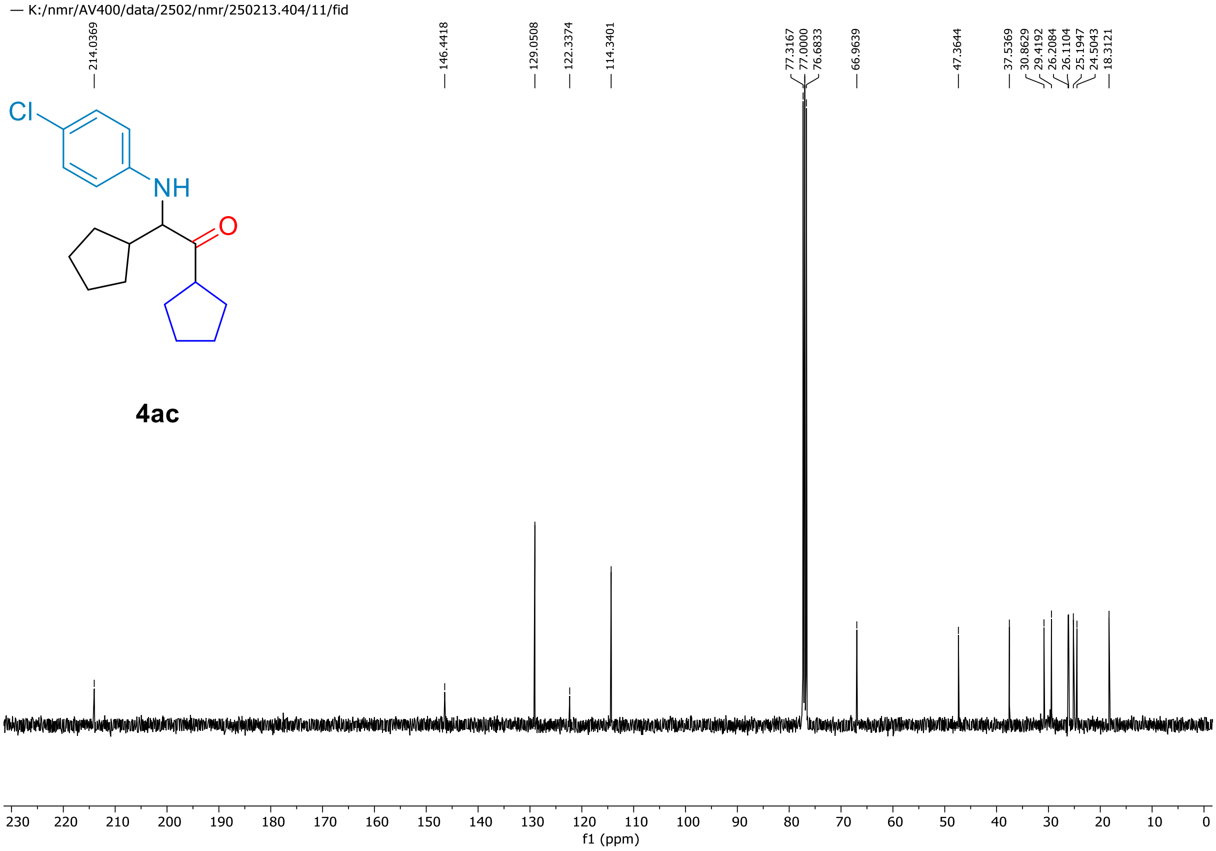

4ad ^{1}H NMR (300 MHz, $CDCl_3$)

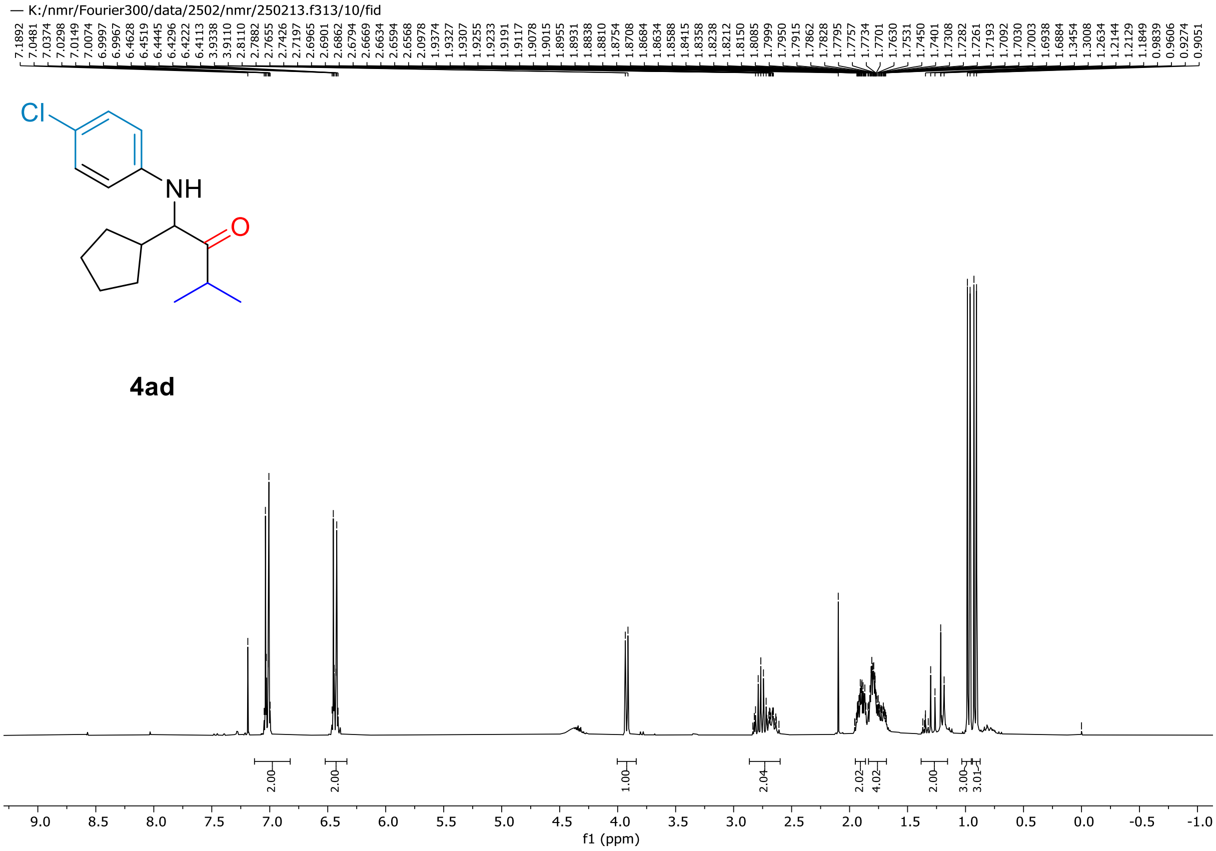

4ad ^{13}C NMR (75 MHz, $CDCl_3$)

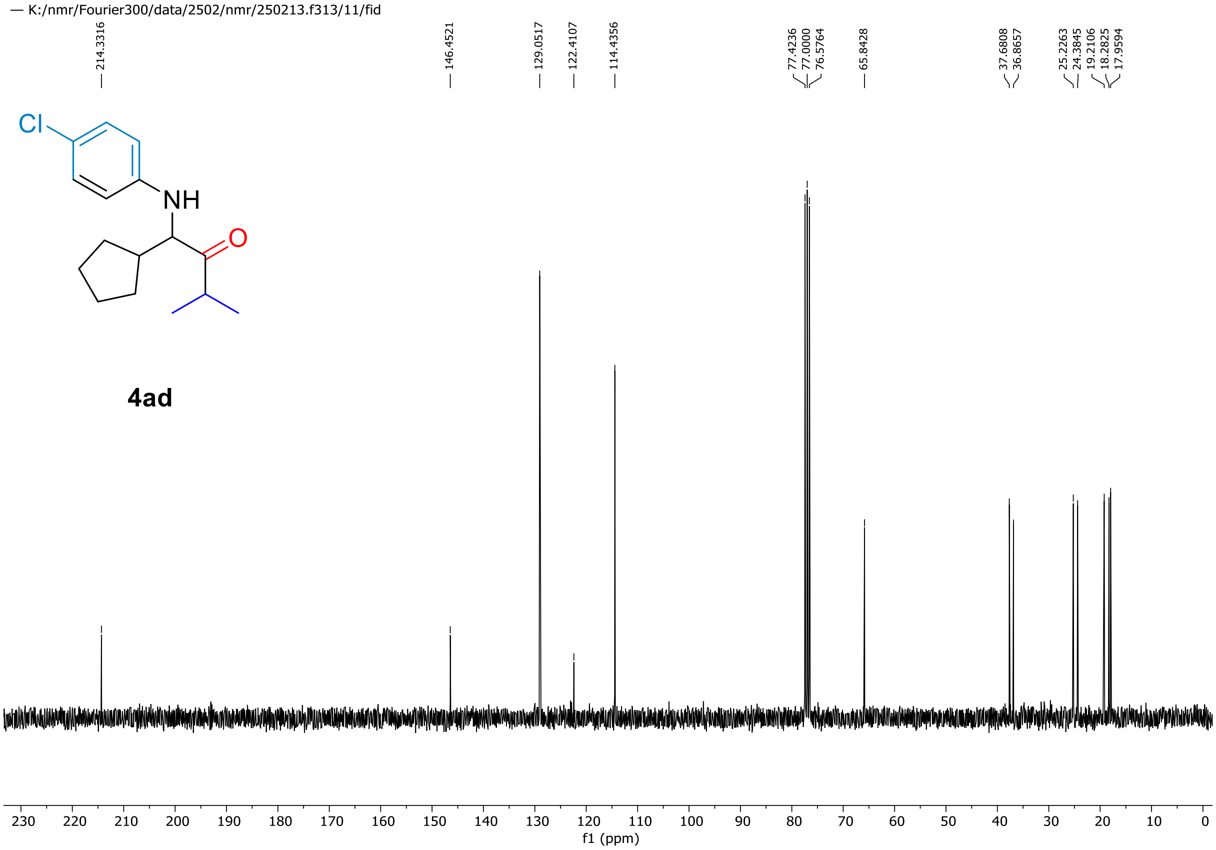

4ae ^{1}H NMR (300 MHz, $CDCl_3$)

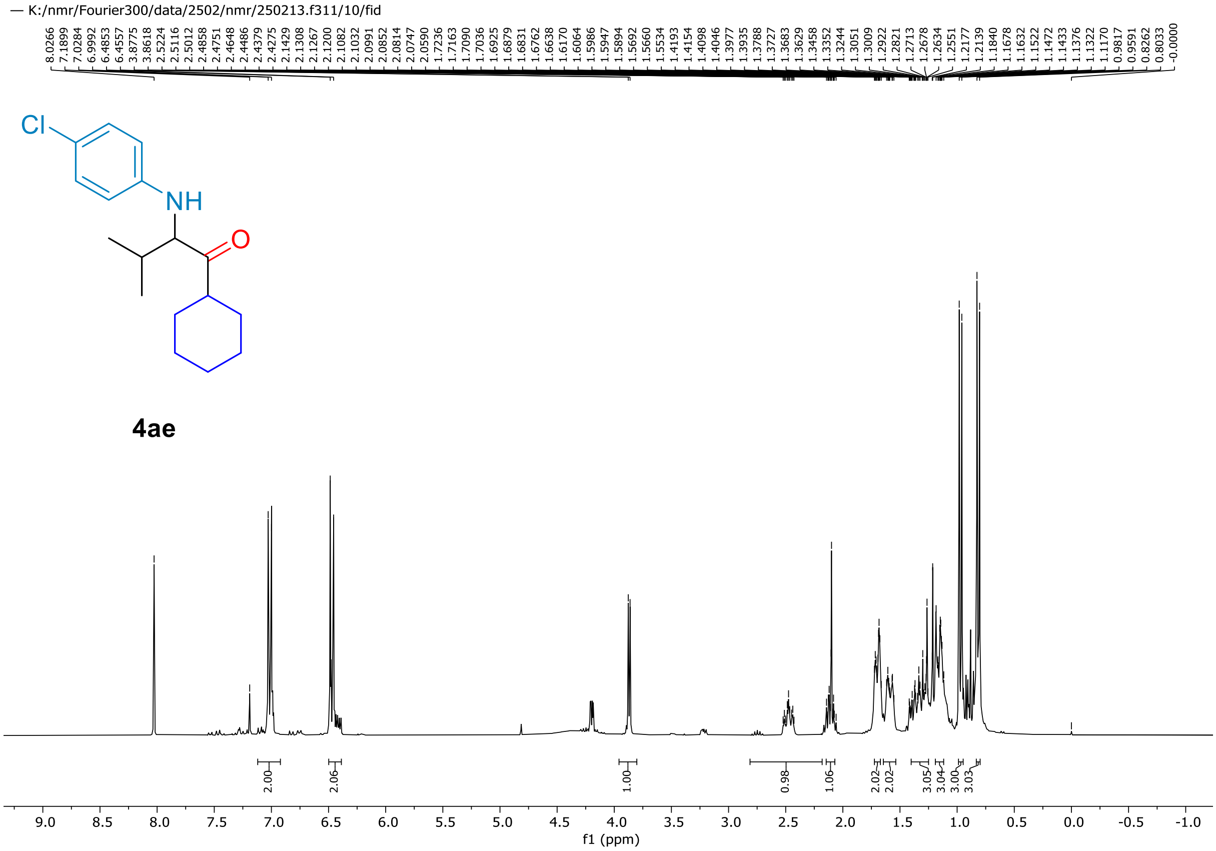

4ae ^{13}C NMR (75 MHz, $CDCl_3$)

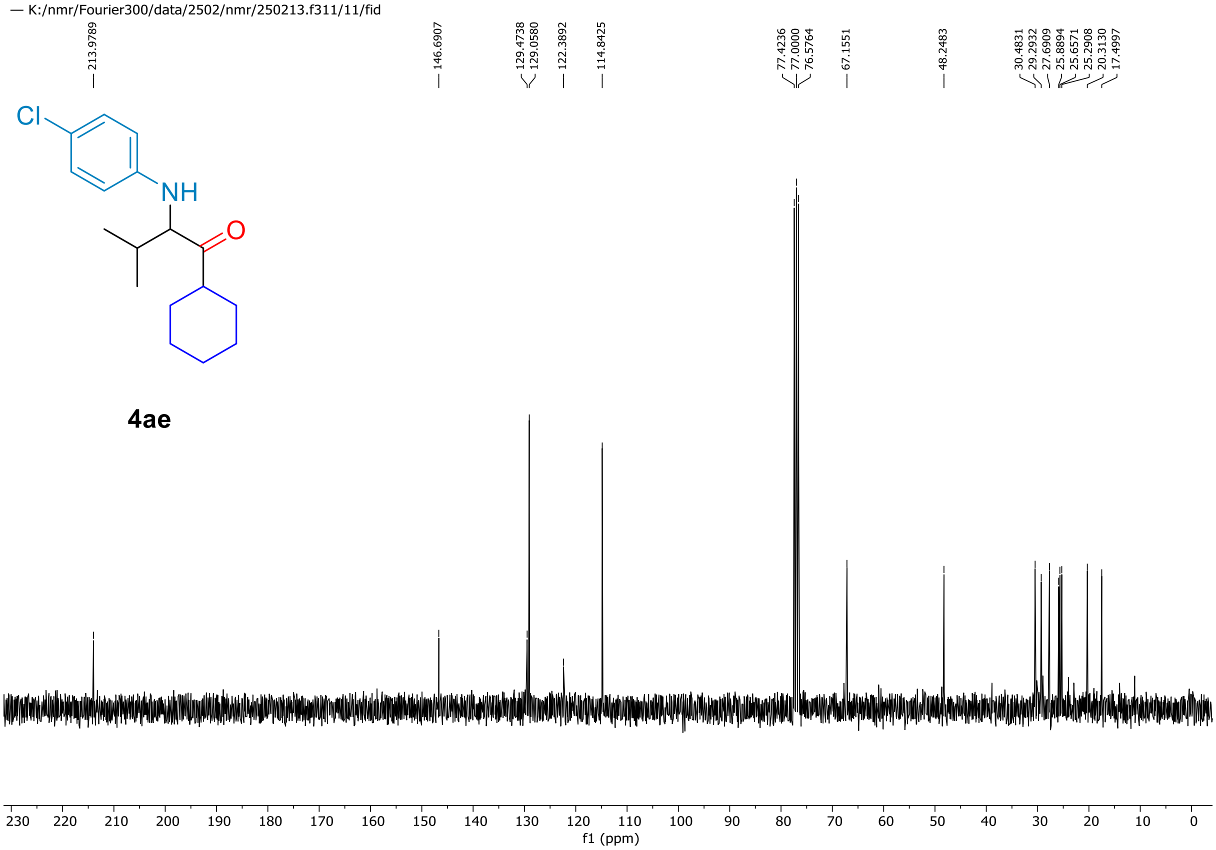

4af ^{1}H NMR (300 MHz, $CDCl_3$)

— K:/nmr/Fourier300/data/2502/nmr/250220.f337/10/fid

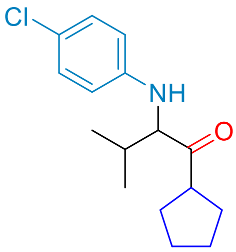

4af

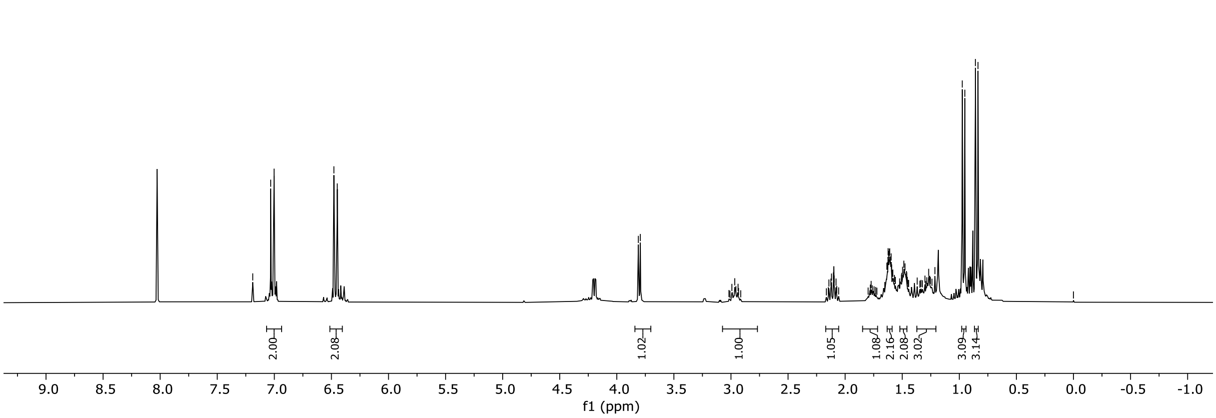

4af ^{13}C NMR (101 MHz, $CDCl_3$)

— K:/nmr/Fourier300/data/2502/nmr/250220.f337/11/fid

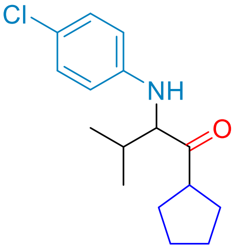

4af

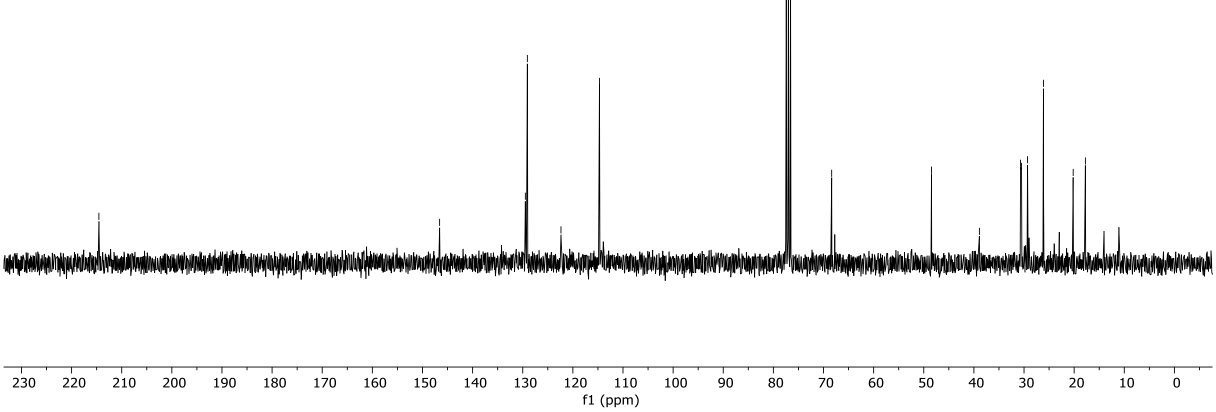

4ag ^{1}H NMR (400 MHz, $CDCl_3$)

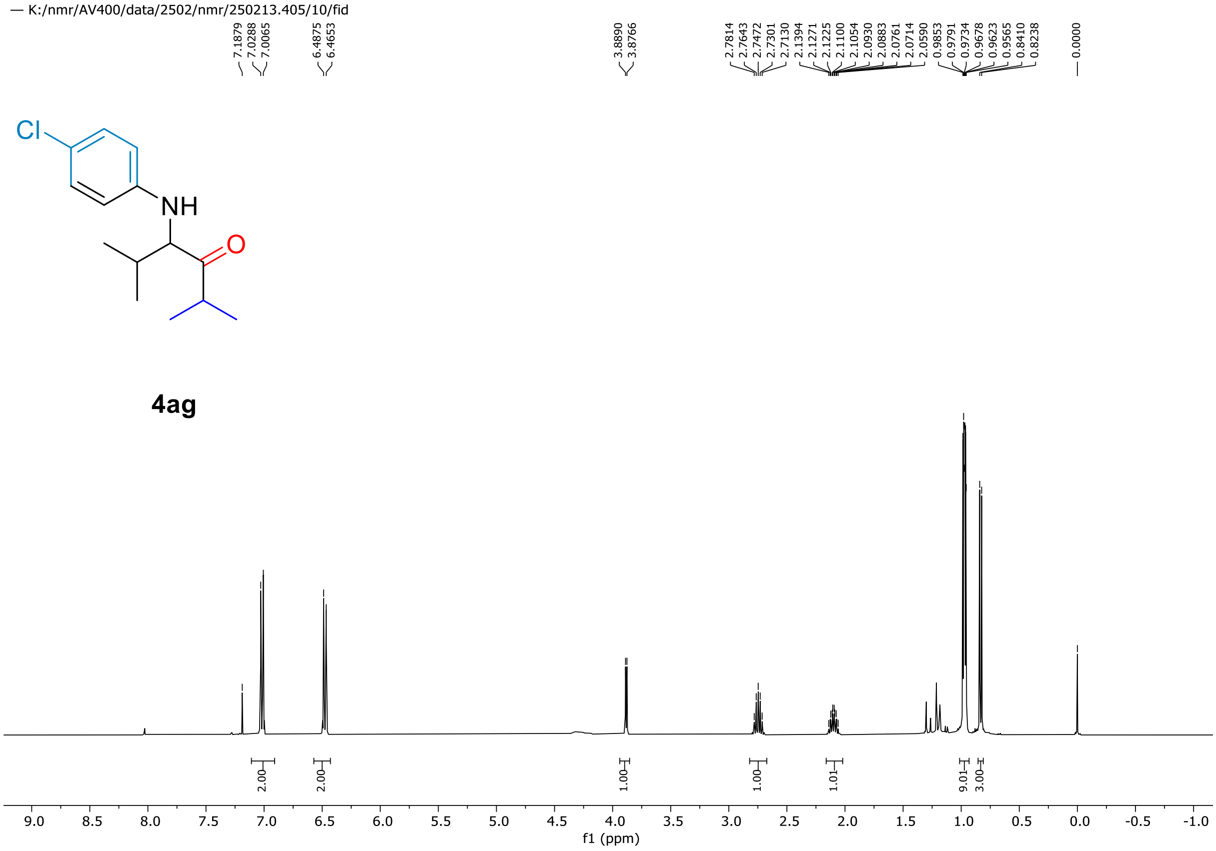

4ag ^{13}C NMR (101 MHz, $CDCl_3$)

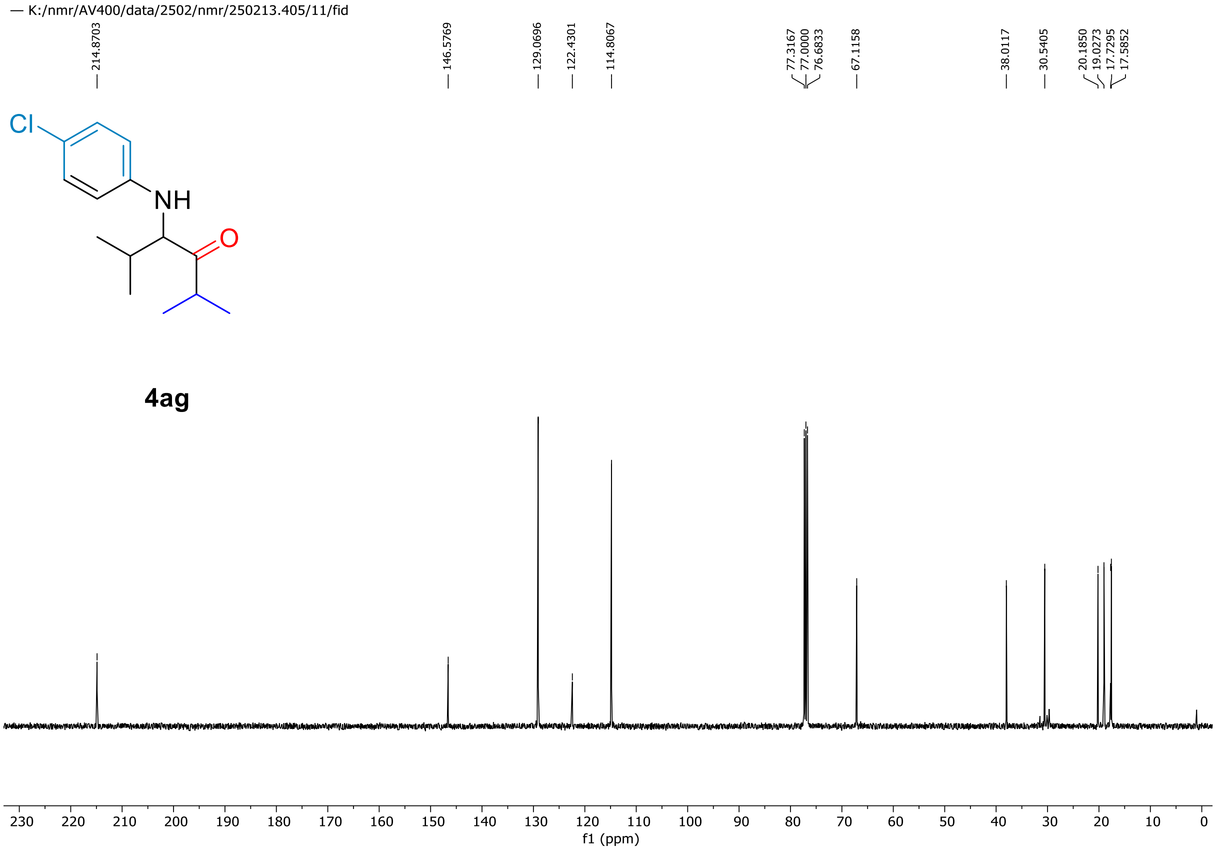

4ah ^{1}H NMR (300 MHz, $CDCl_3$)

— K:/nmr/Fourier300/data/2502/nmr/250220.f336/10/fid

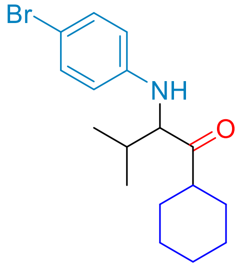

4ah

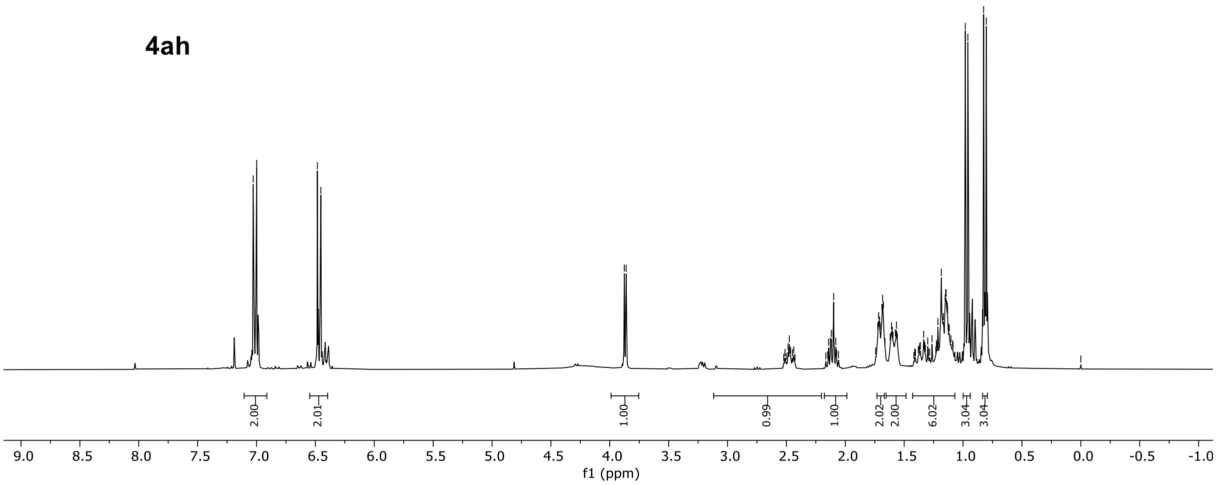

4ah ^{13}C NMR (75 MHz, $CDCl_3$)

— K:/nmr/Fourier300/data/2502/nmr/250220.f336/11/fid

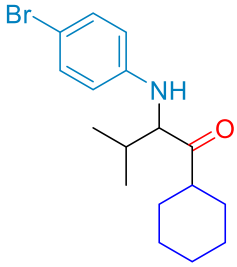

4ah

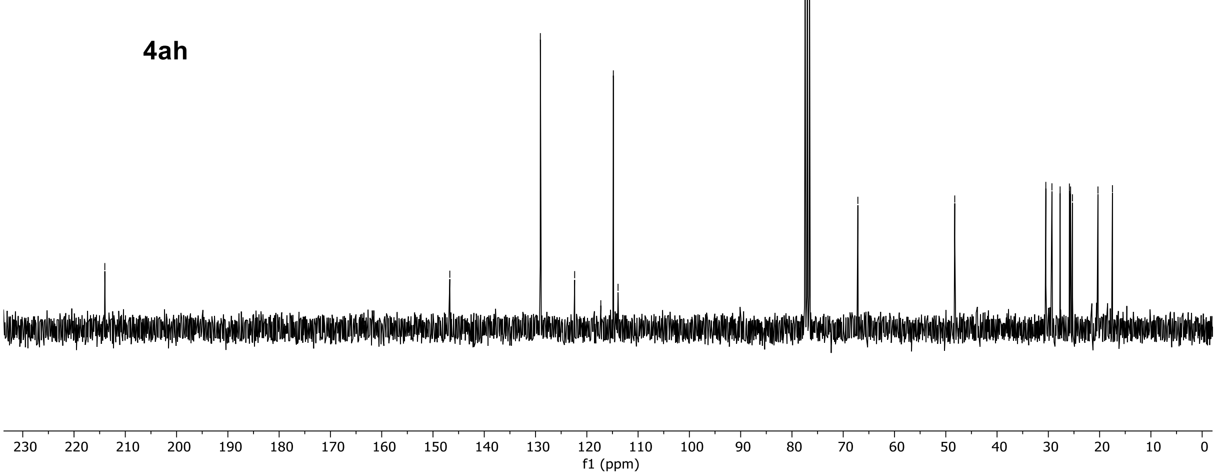

4ai ^{1}H NMR (400 MHz, $CDCl_3$)

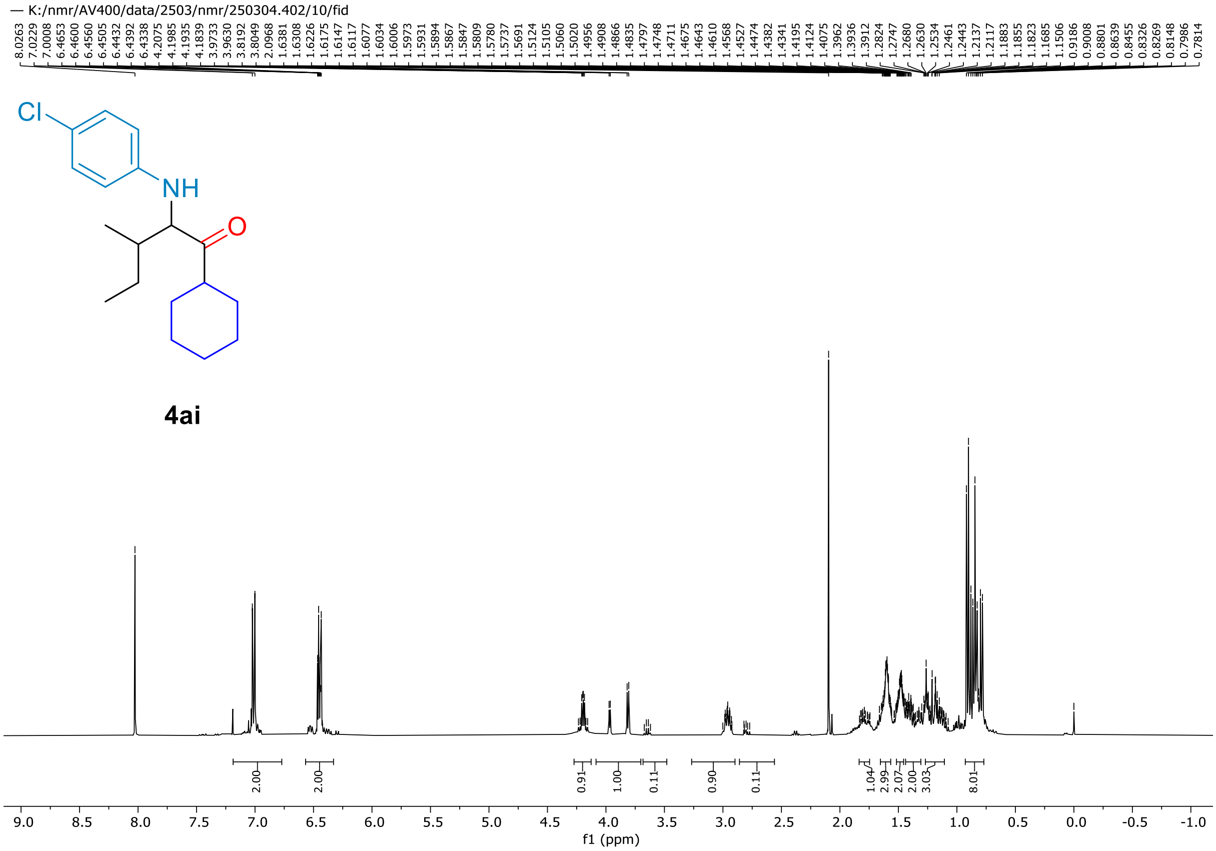

4ai ^{13}C NMR (110 MHz, $CDCl_3$)

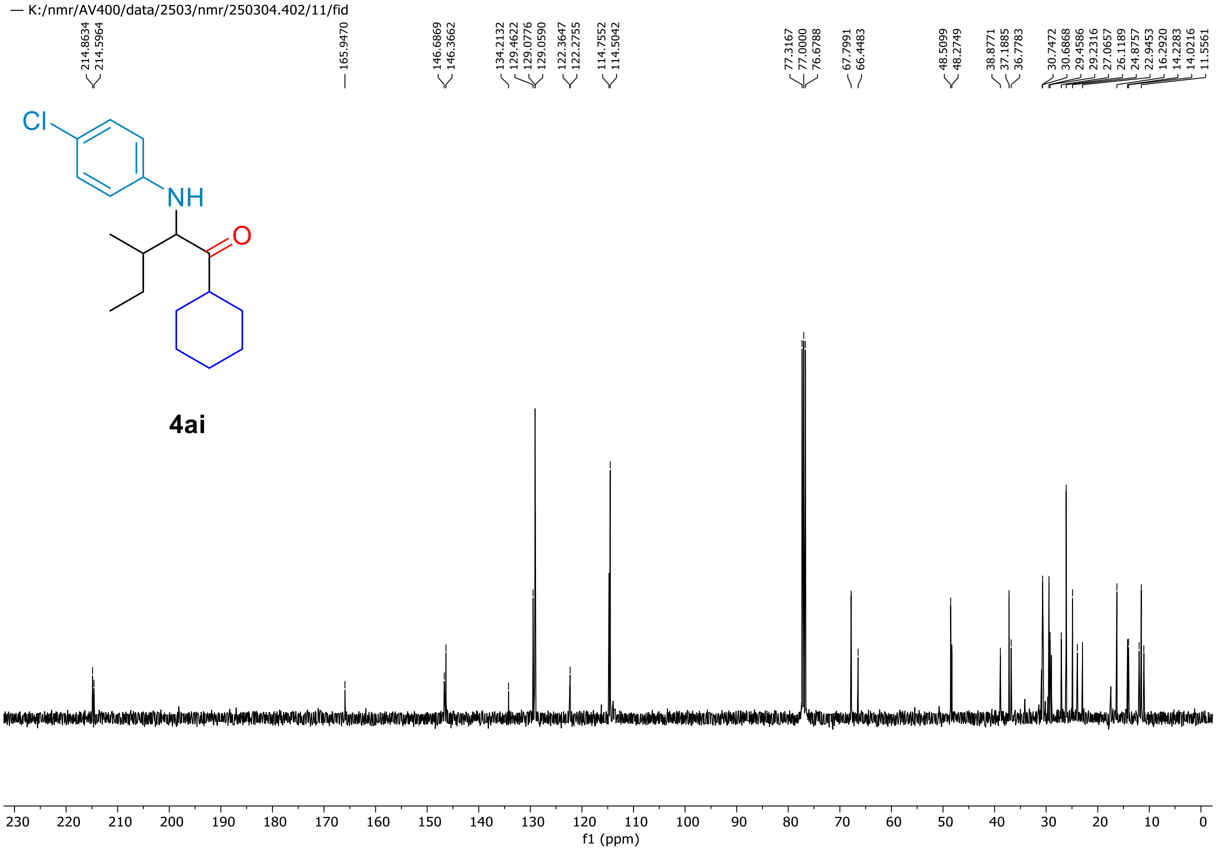

4aj ^{1}H NMR (300 MHz, $CDCl_3$)

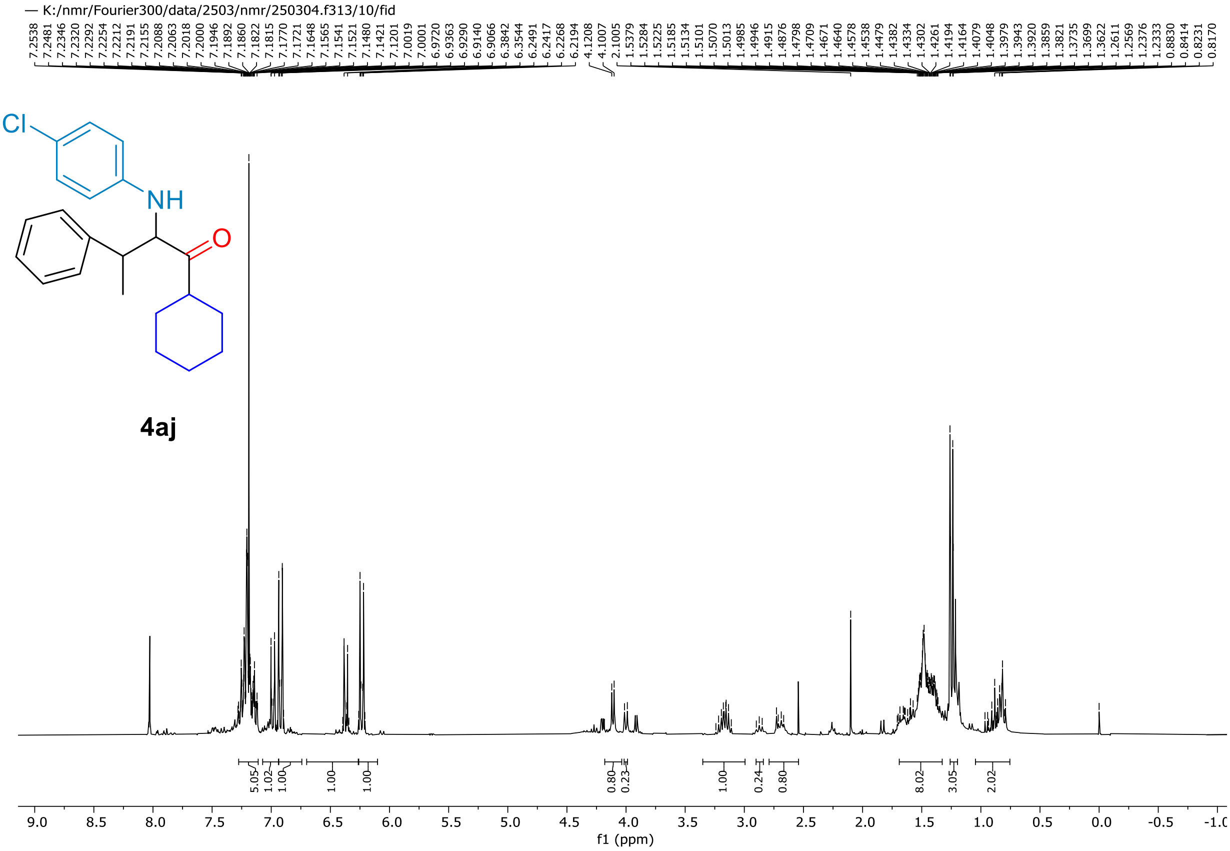

4aj

4aj ^{13}C NMR (75 MHz, $CDCl_3$)

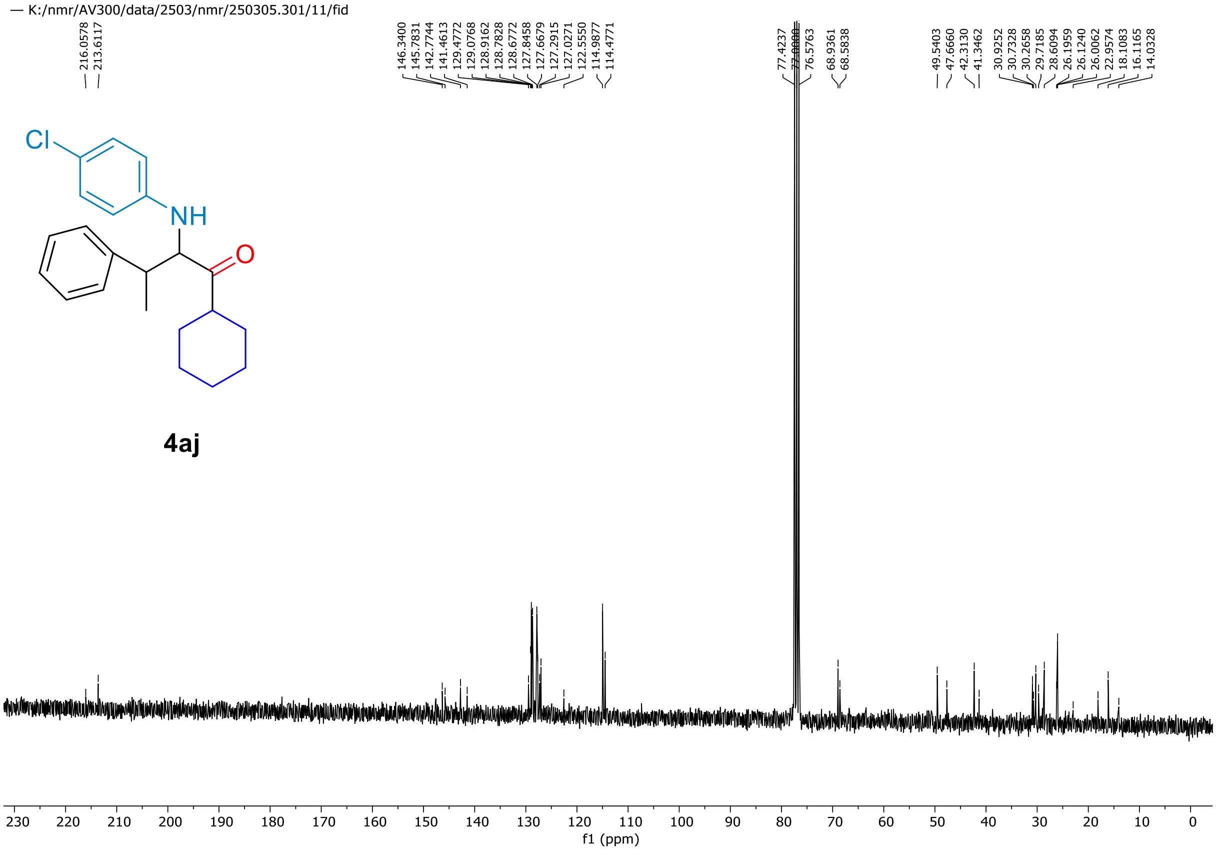

4aj

4ak ^{1}H NMR (300 MHz, $CDCl_3$)

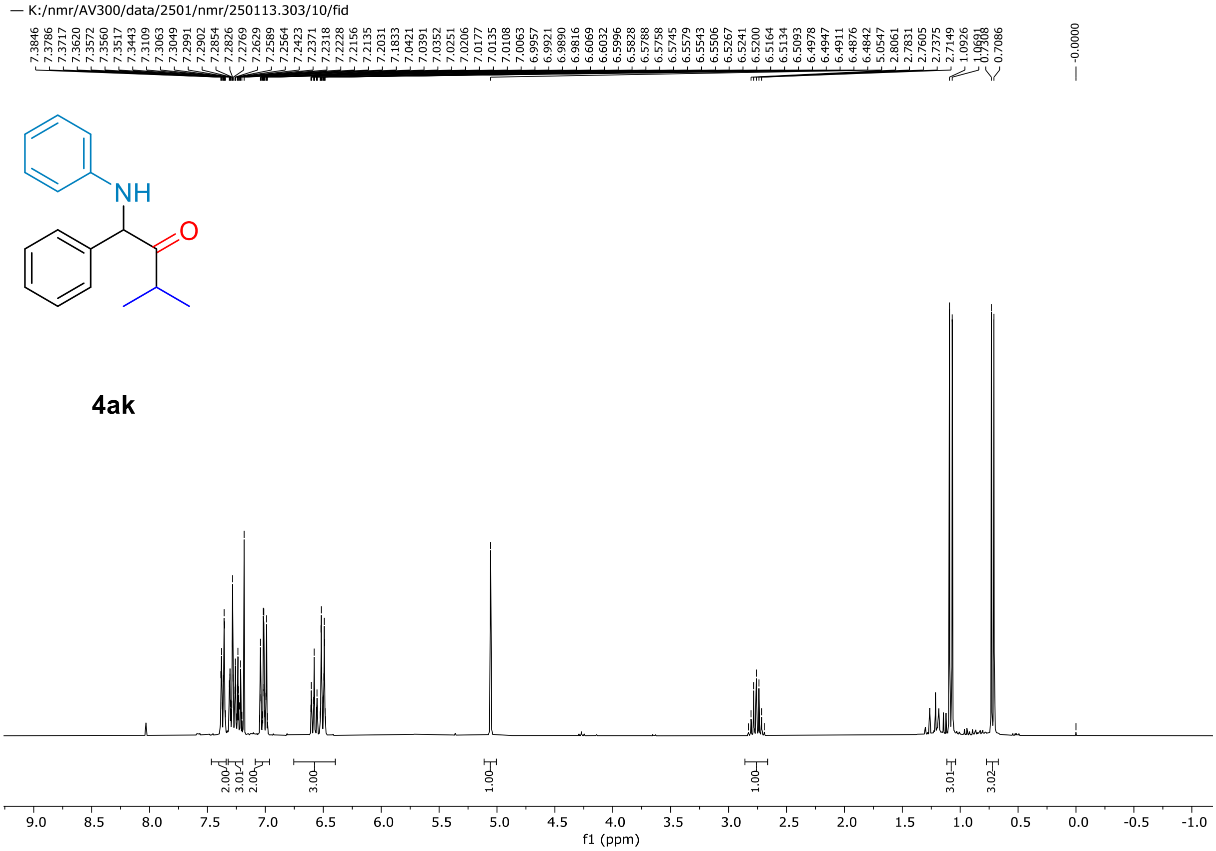

4ak ^{13}C NMR (75 MHz, $CDCl_3$)

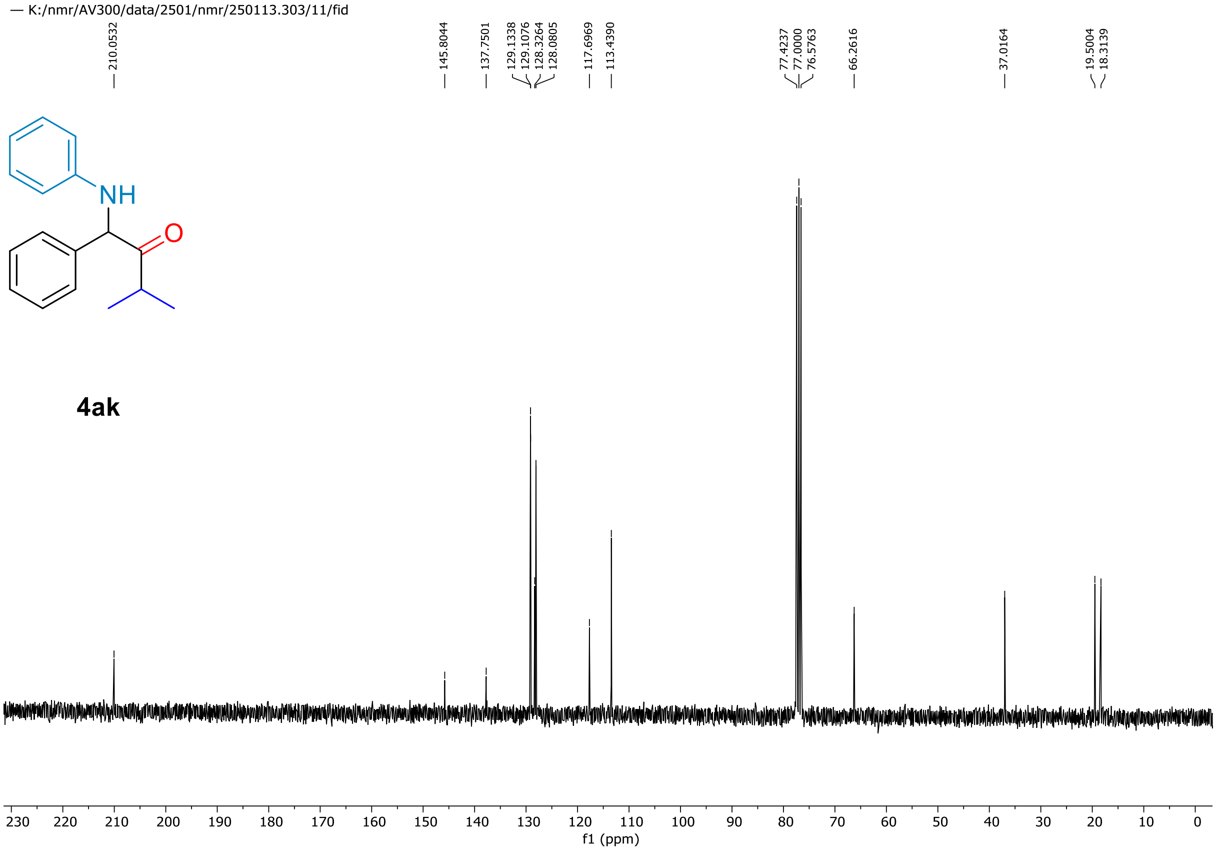

4al ^{1}H NMR (300 MHz, CDCl$_3$)

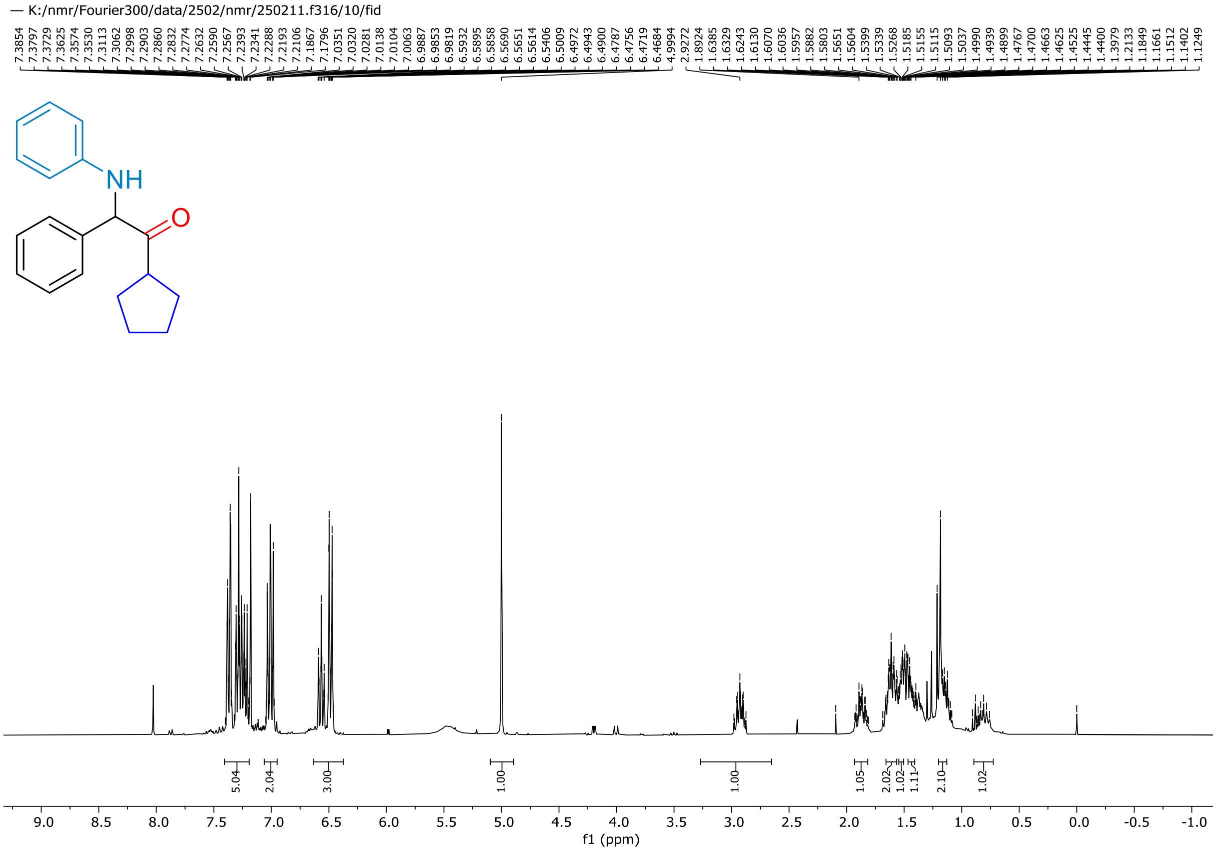

4al ^{13}C NMR (75 MHz, CDCl$_3$)

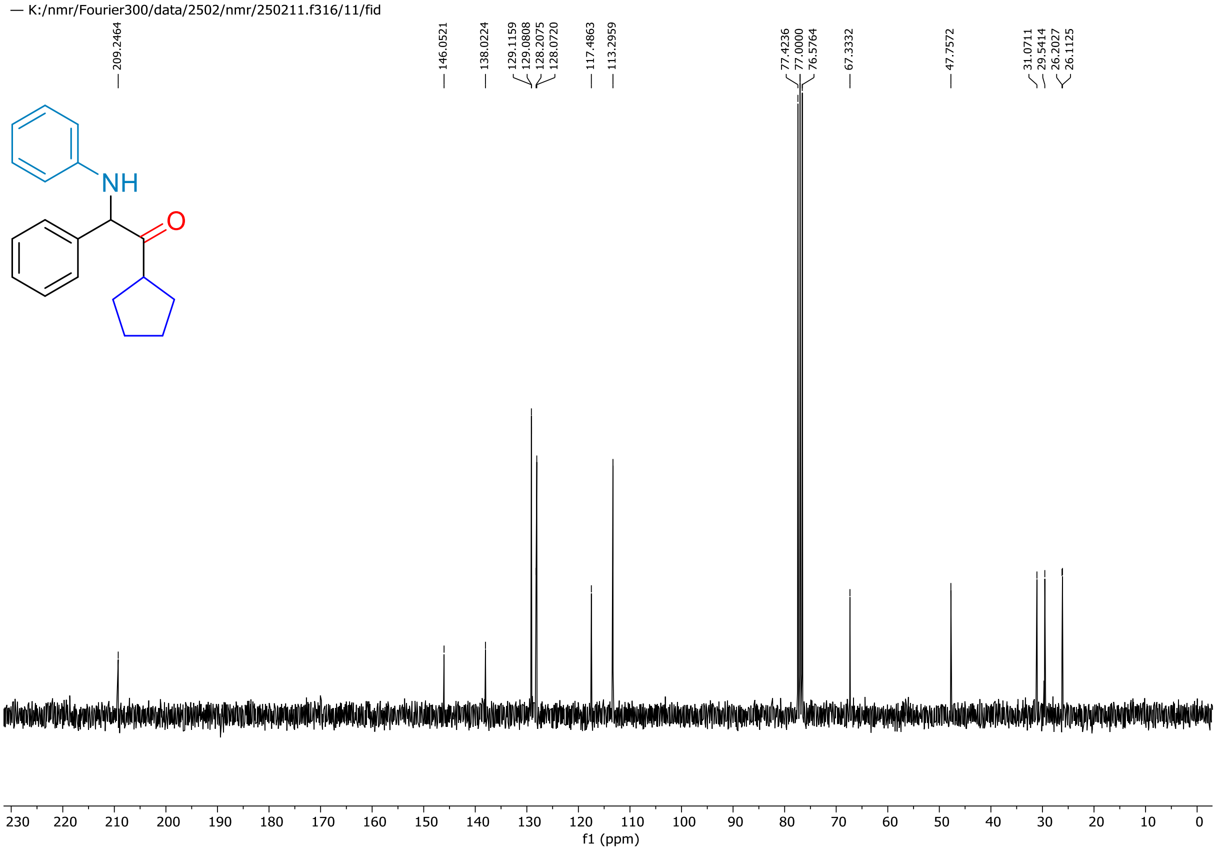

4am ^{1}H NMR (300 MHz, $CDCl_3$)

— K:/nmr/Fourier300/data/2503/nmr/250304.f314/10/fid

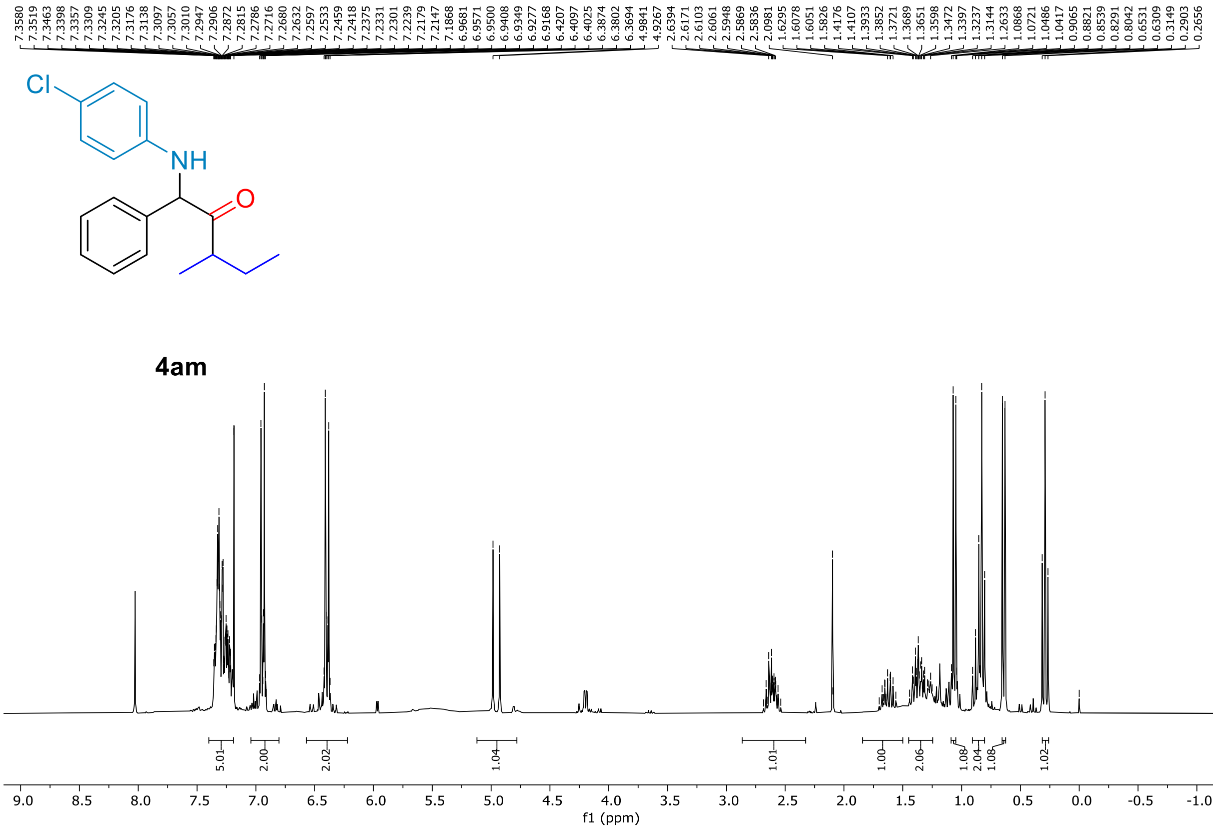

4am ^{13}C NMR (75 MHz, $CDCl_3$)

— K:/nmr/Fourier300/data/2503/nmr/250304.f314/11/fid

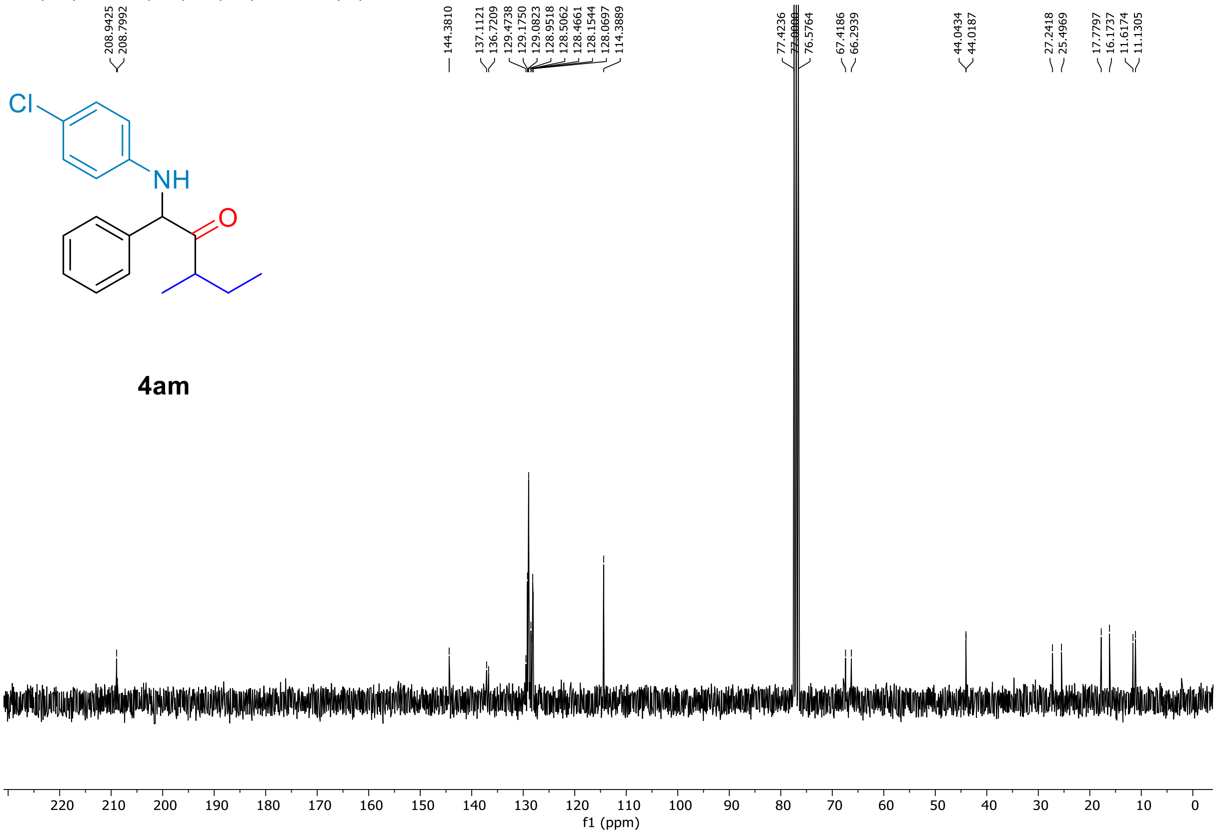

4an ^{1}H NMR (400 MHz, $CDCl_3$)

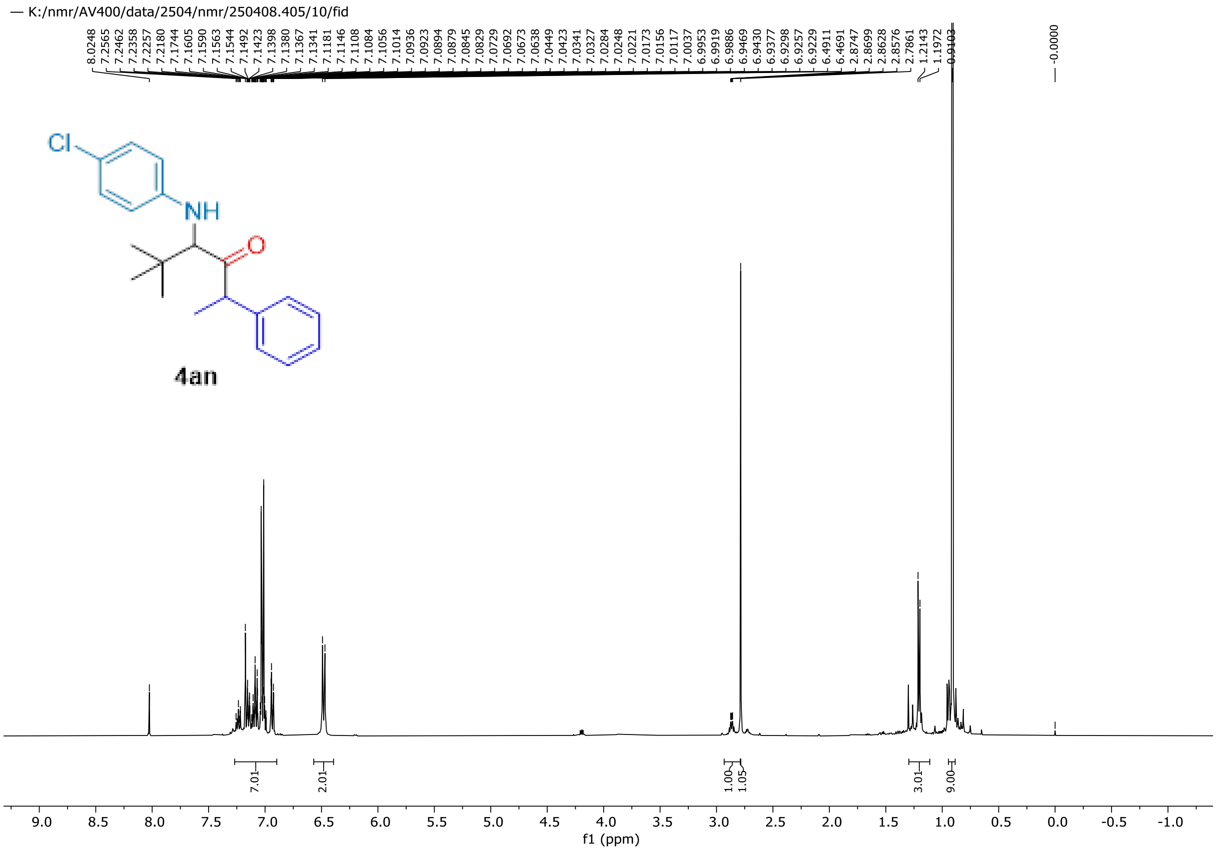

4an ^{13}C NMR (101 MHz, $CDCl_3$)

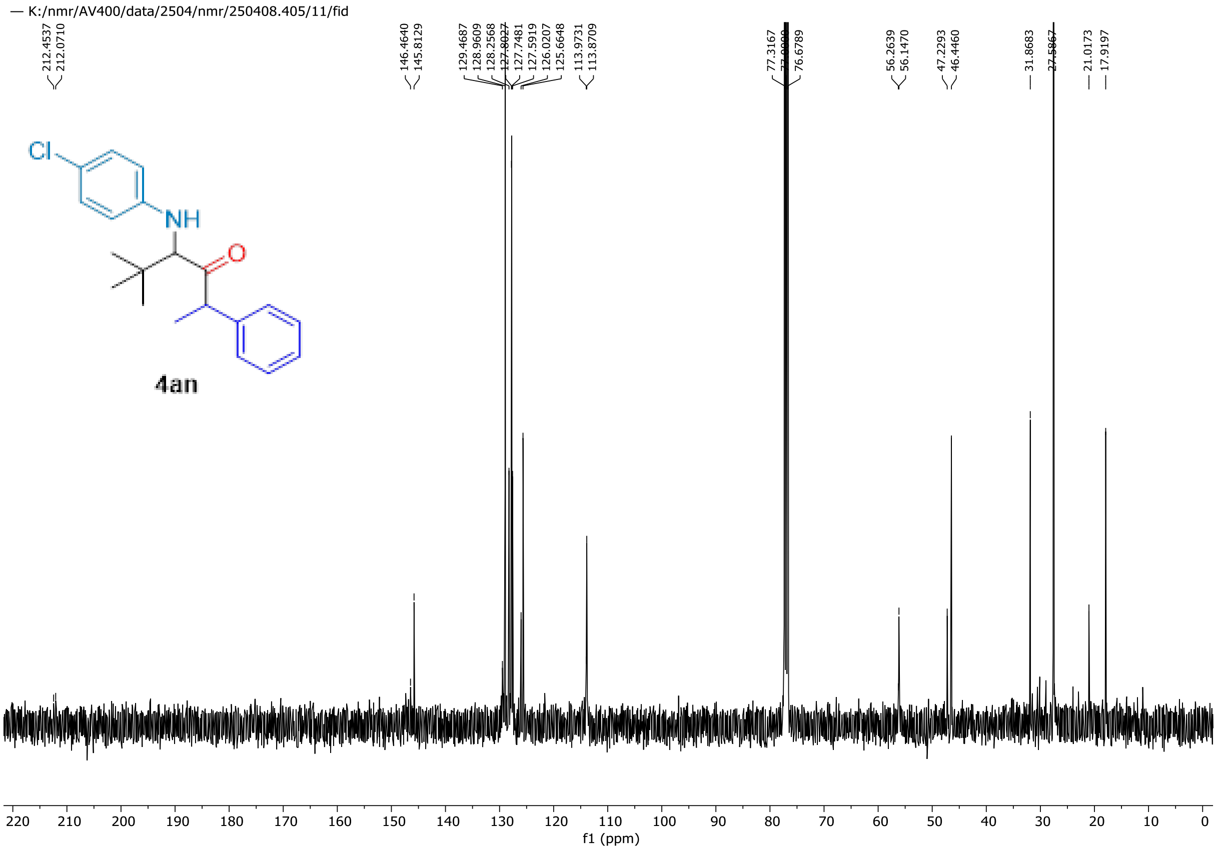

4ao ^{1}H NMR (300 MHz, $CDCl_3$)

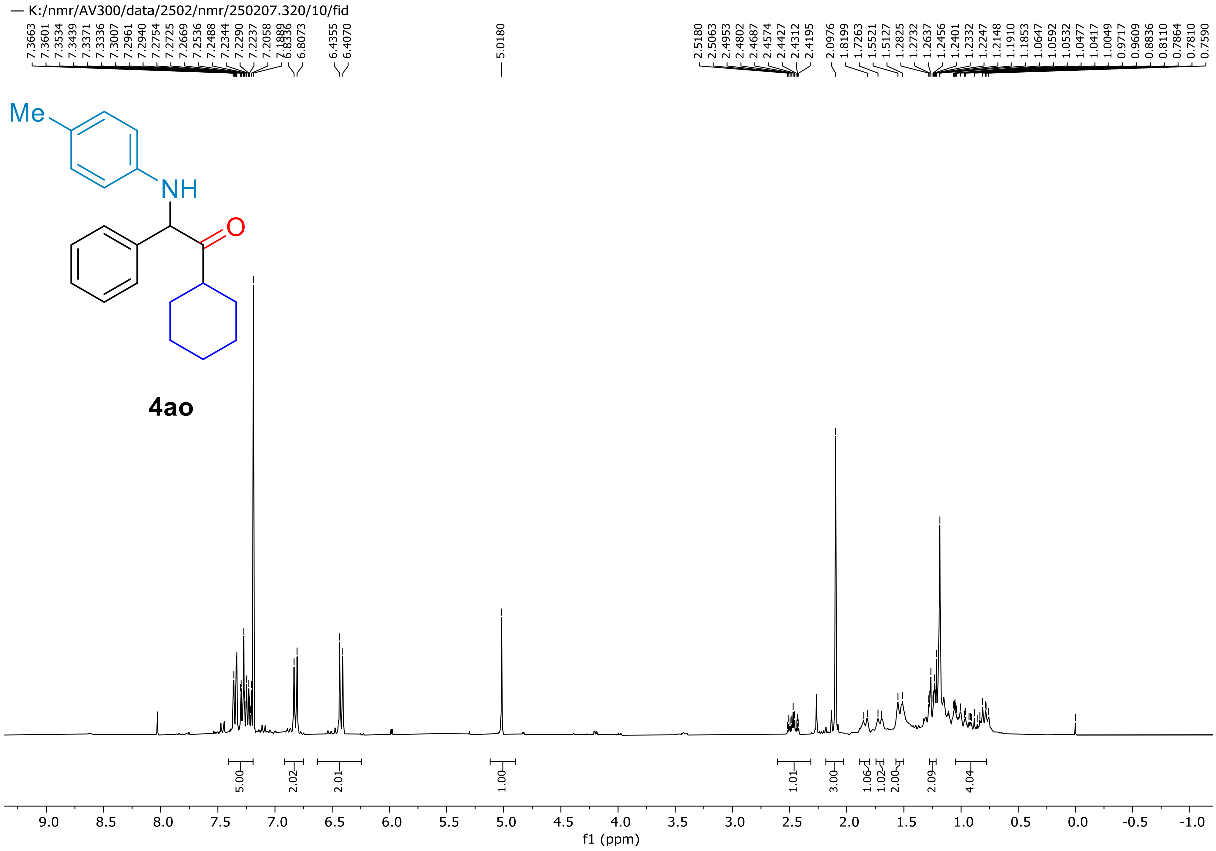

4ao ^{13}C NMR (75 MHz, $CDCl_3$)

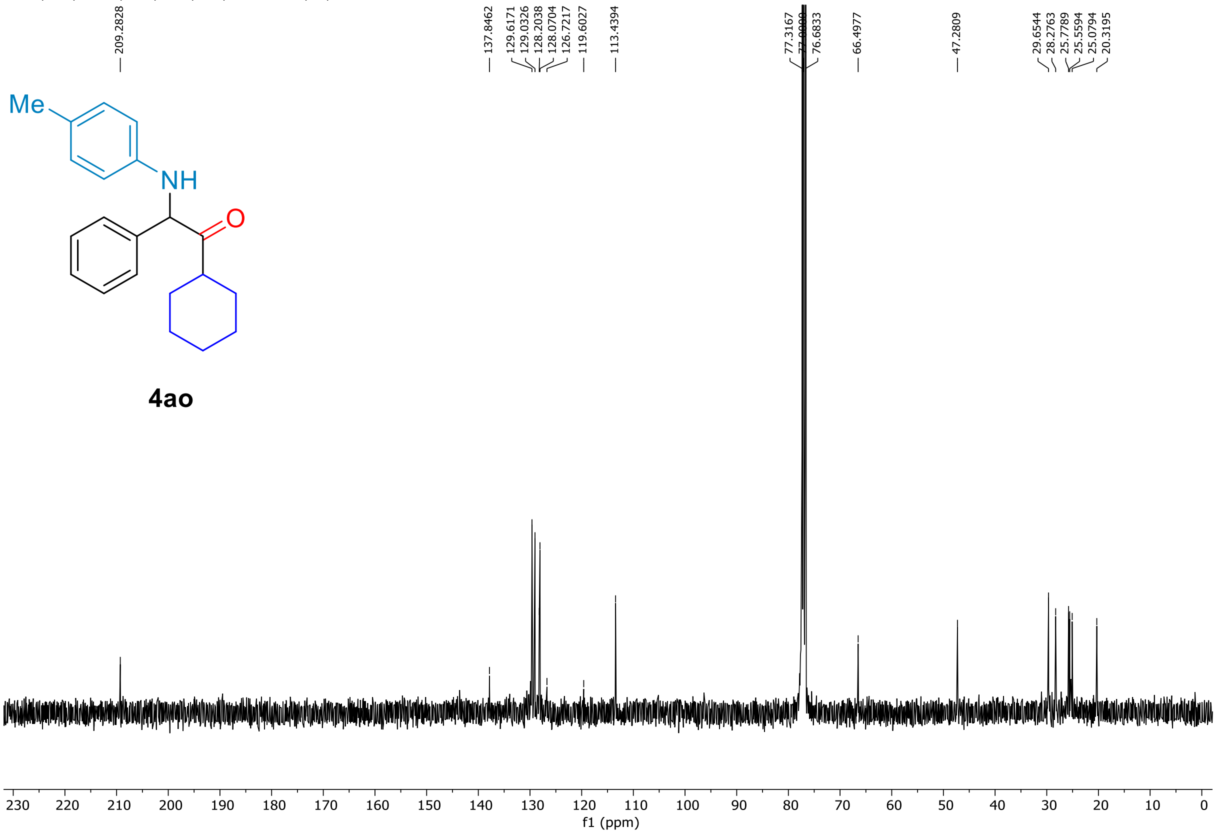

4ap ^{1}H NMR (300 MHz, $CDCl_3$)

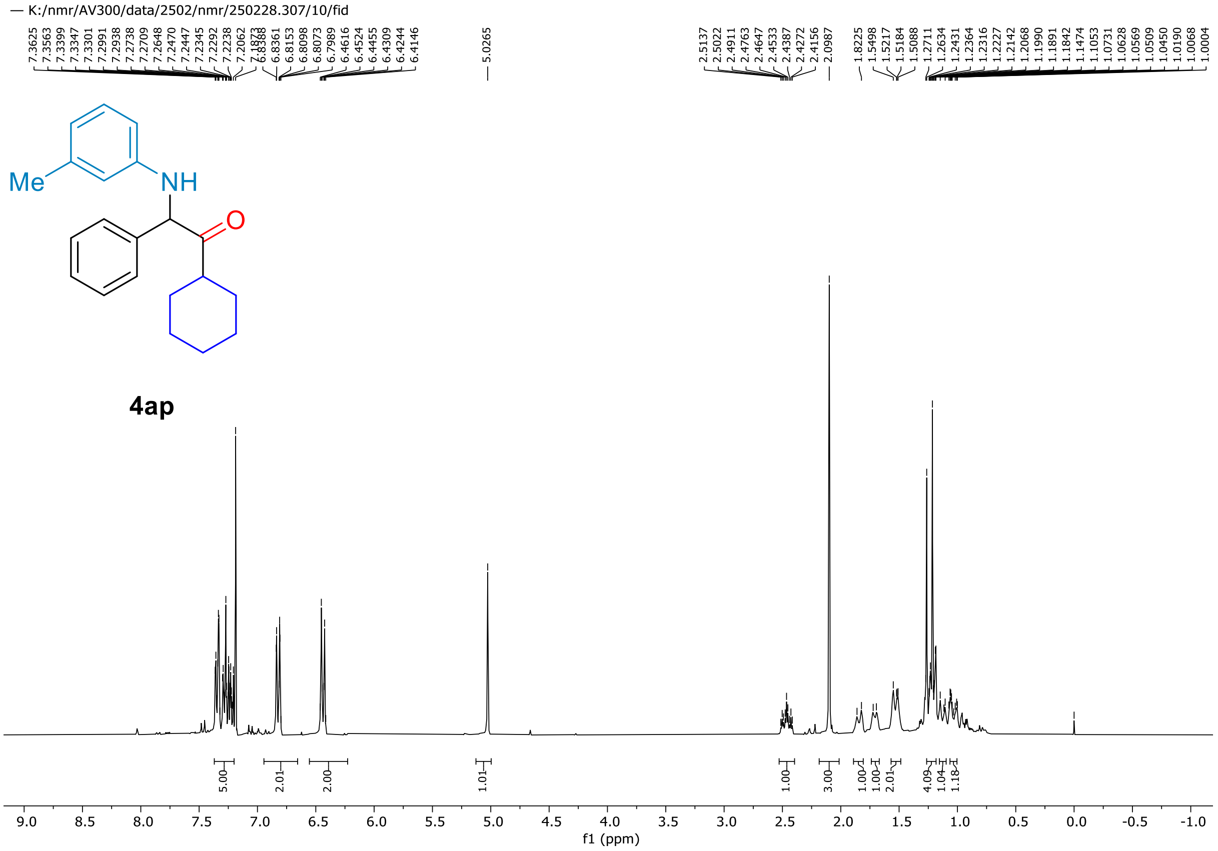

4ap ^{13}C NMR (75 MHz, $CDCl_3$)

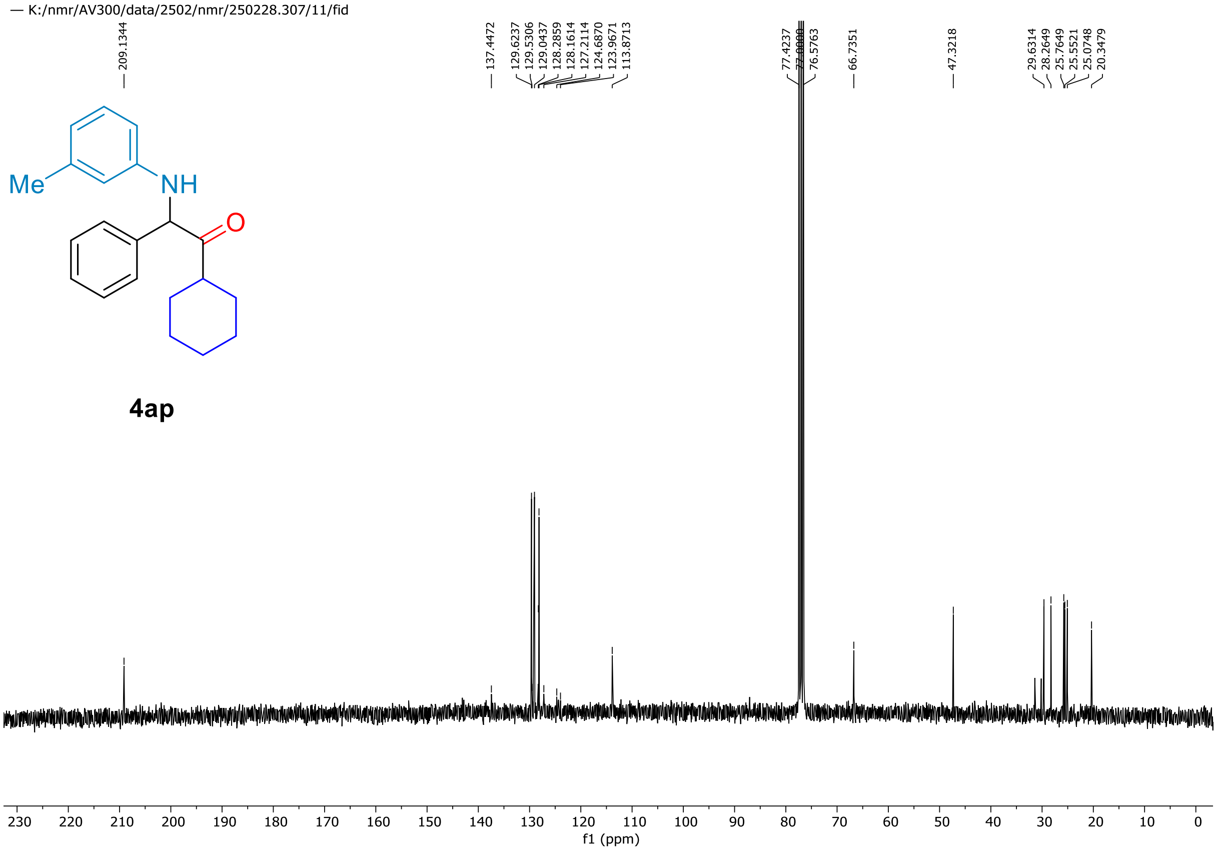

4aq ^{1}H NMR (400 MHz, $CDCl_3$)

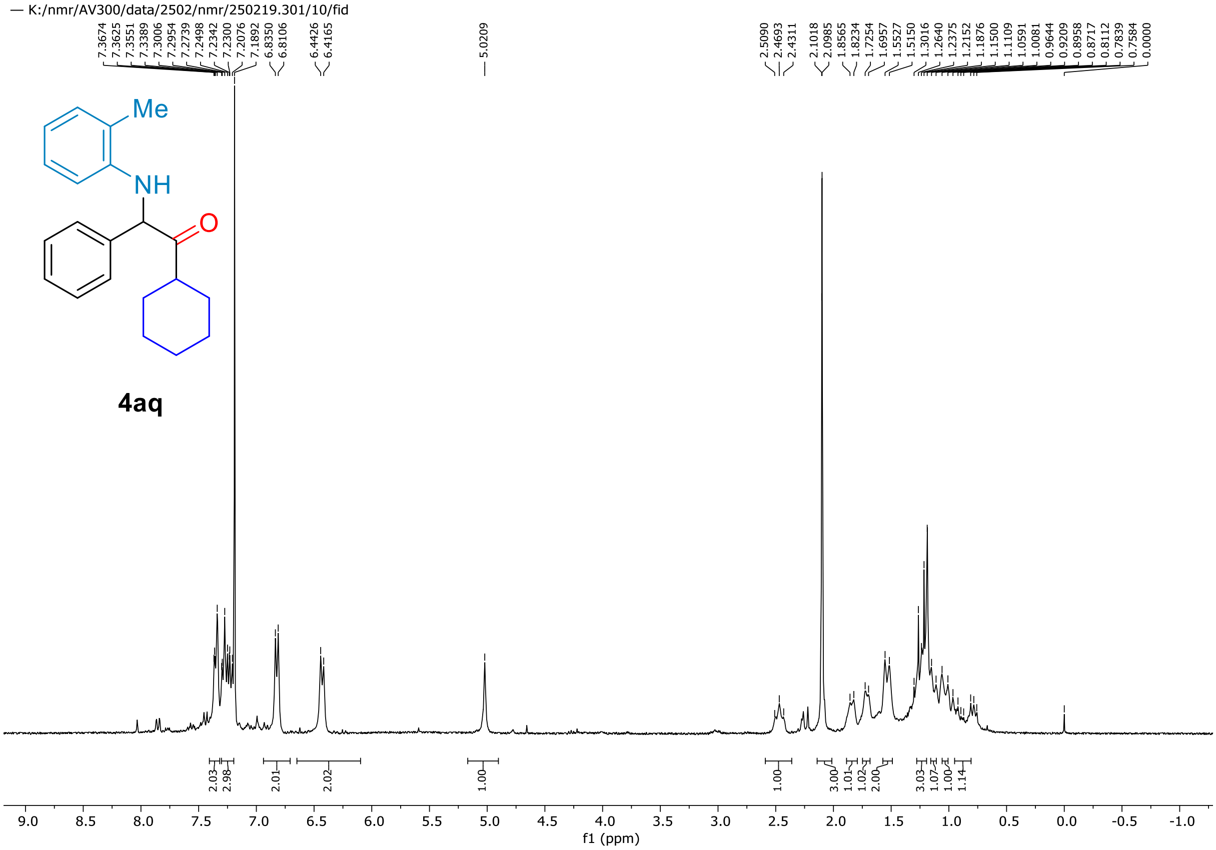

4aq ^{13}C NMR (101 MHz, $CDCl_3$)

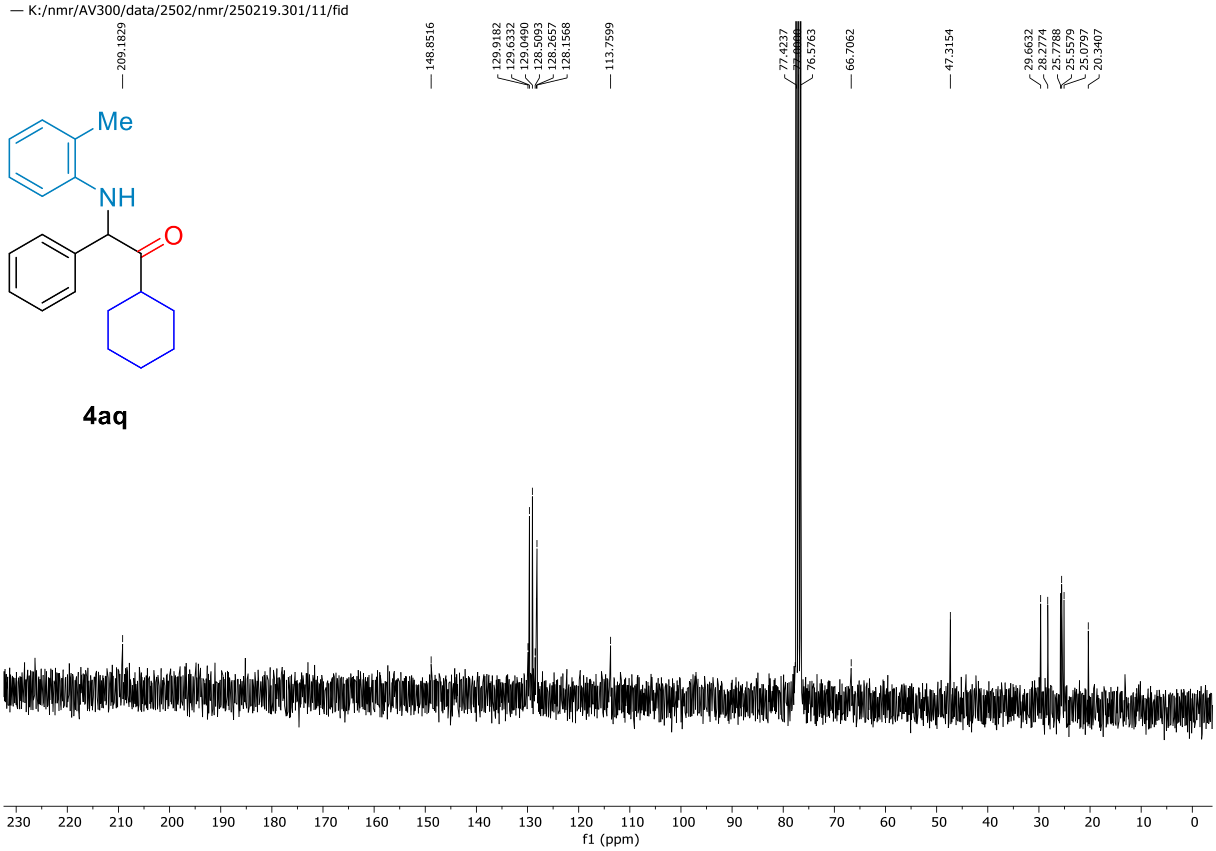

4ar ^{1}H NMR (300 MHz, $CDCl_3$)

— K:/nmr/AV300/data/2502/nmr/250207.306/10/fid

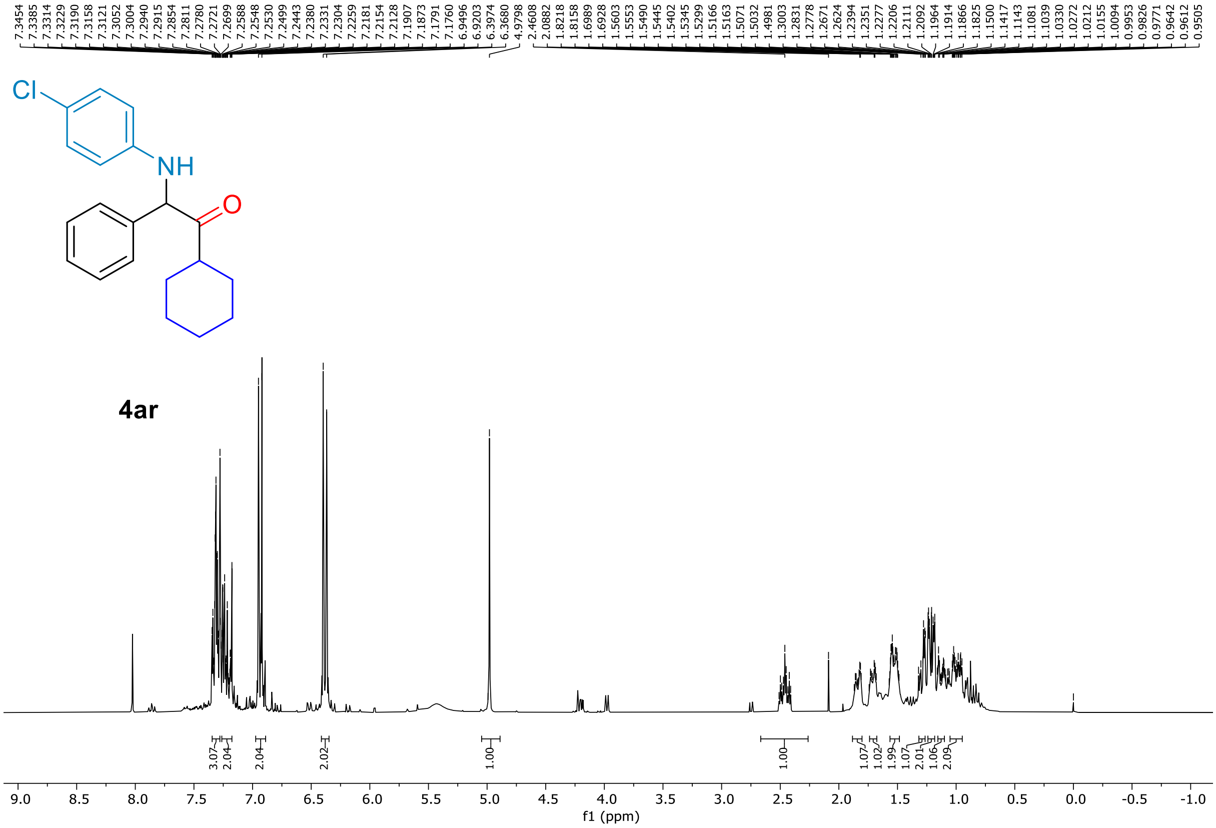

4ar ^{13}C NMR (75 MHz, $CDCl_3$)

— K:/nmr/AV300/data/2502/nmr/250207.306/11/fid

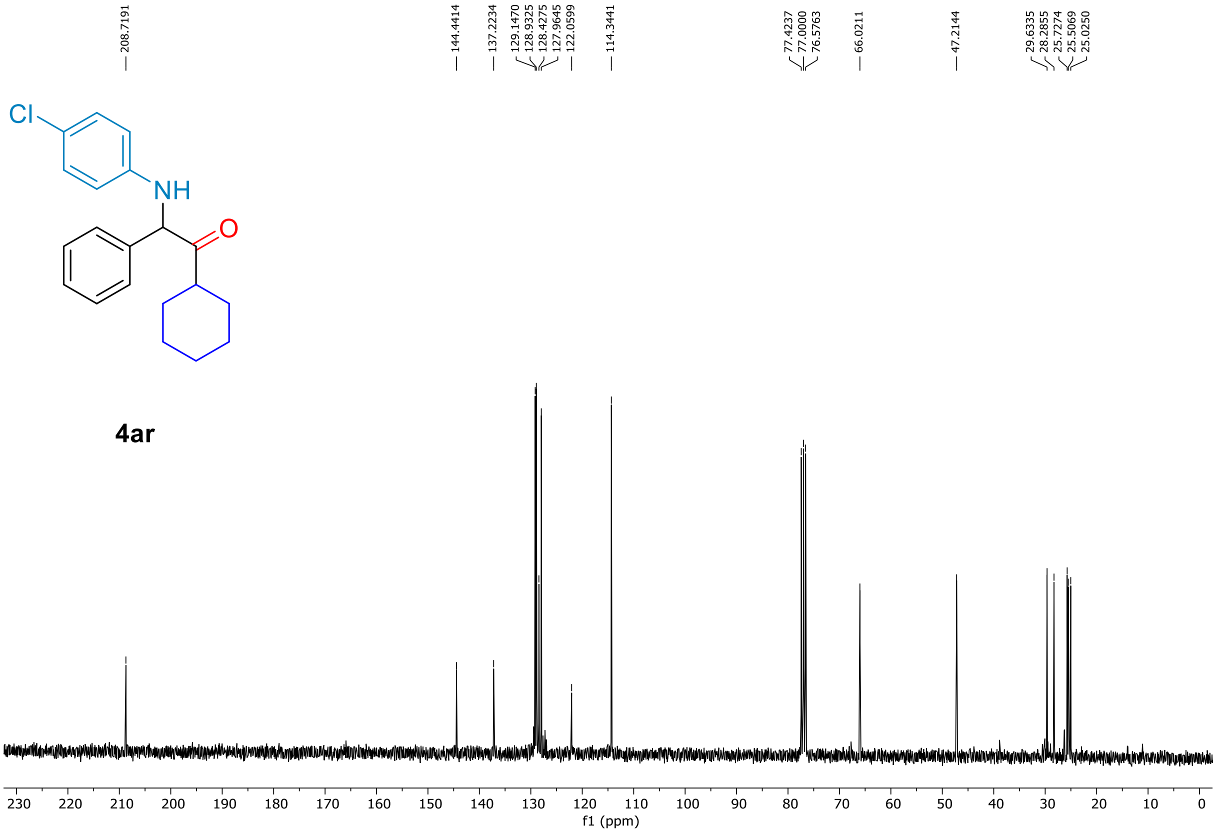

4as ^{1}H NMR (300 MHz, $CDCl_3$)

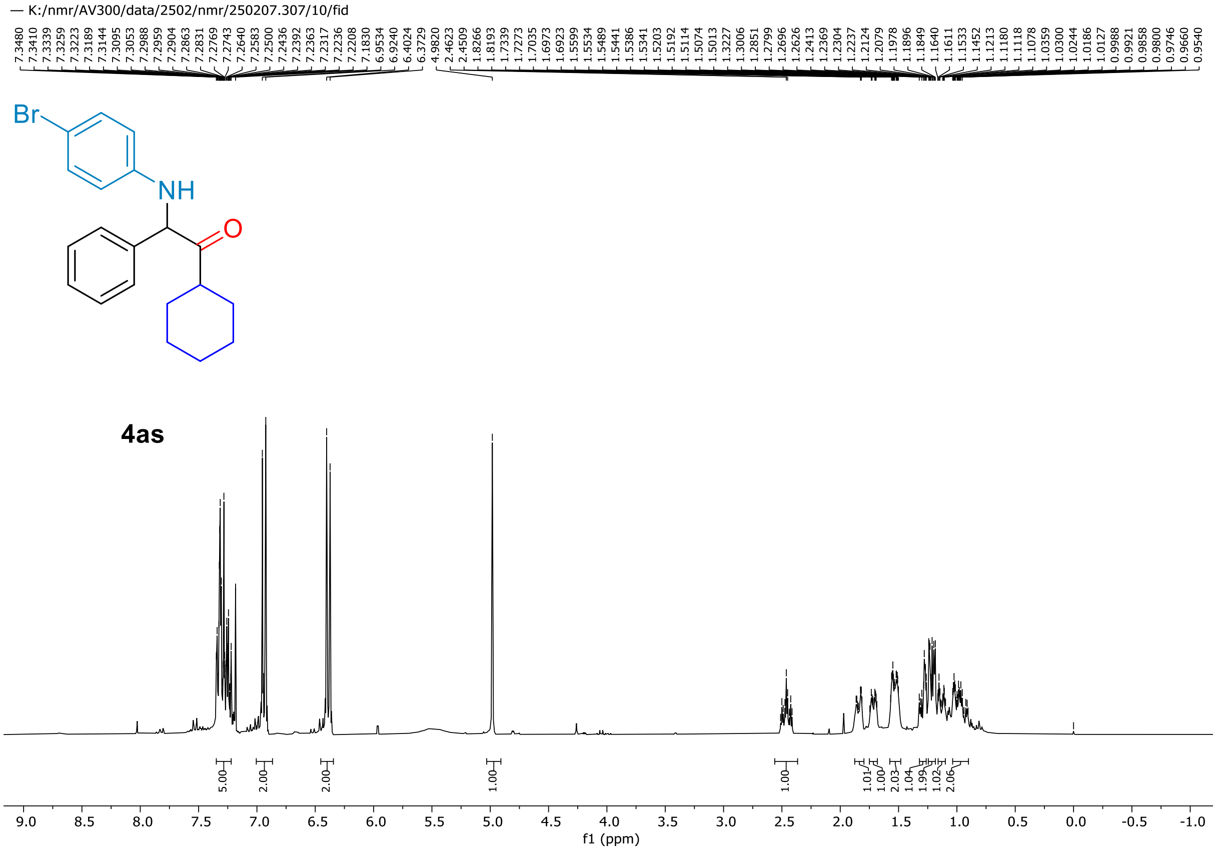

4as ^{13}C NMR (75 MHz, $CDCl_3$)

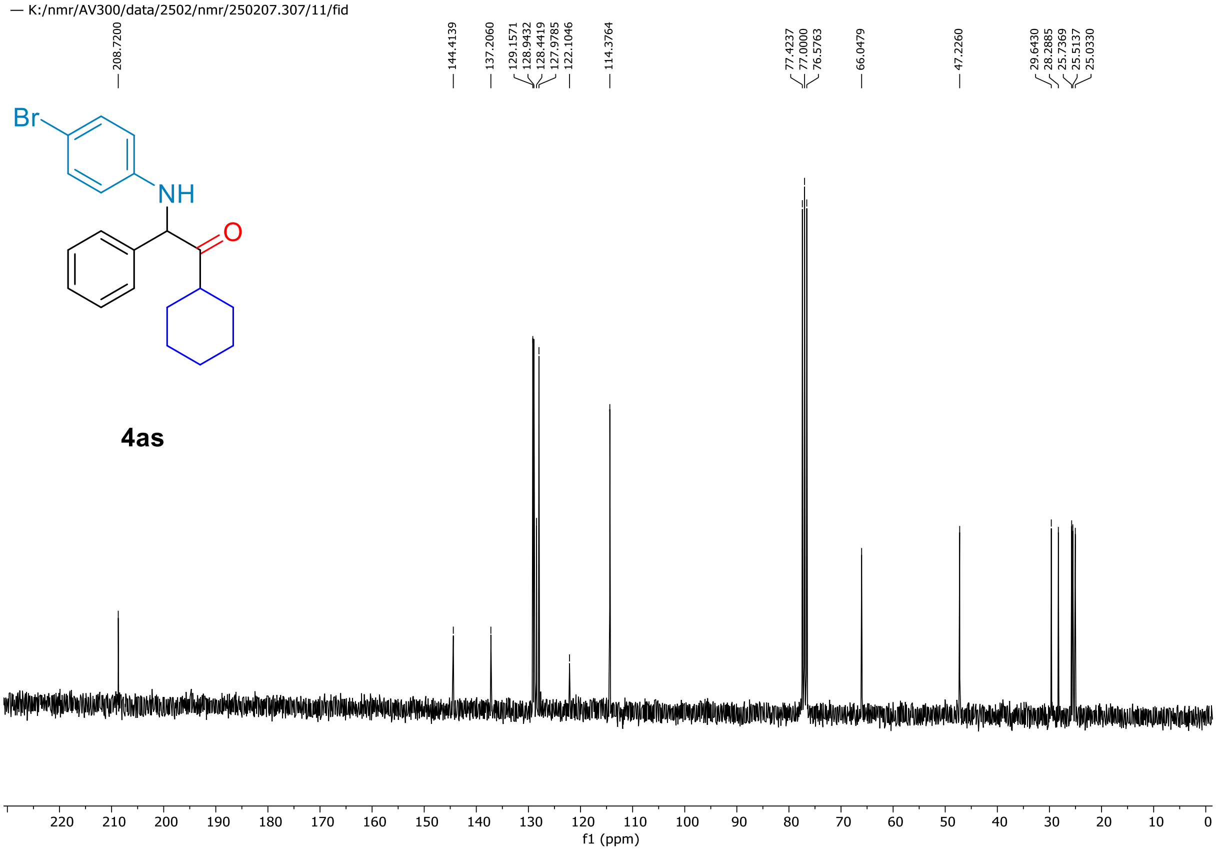

4at ^{1}H NMR (300 MHz, $CDCl_3$)

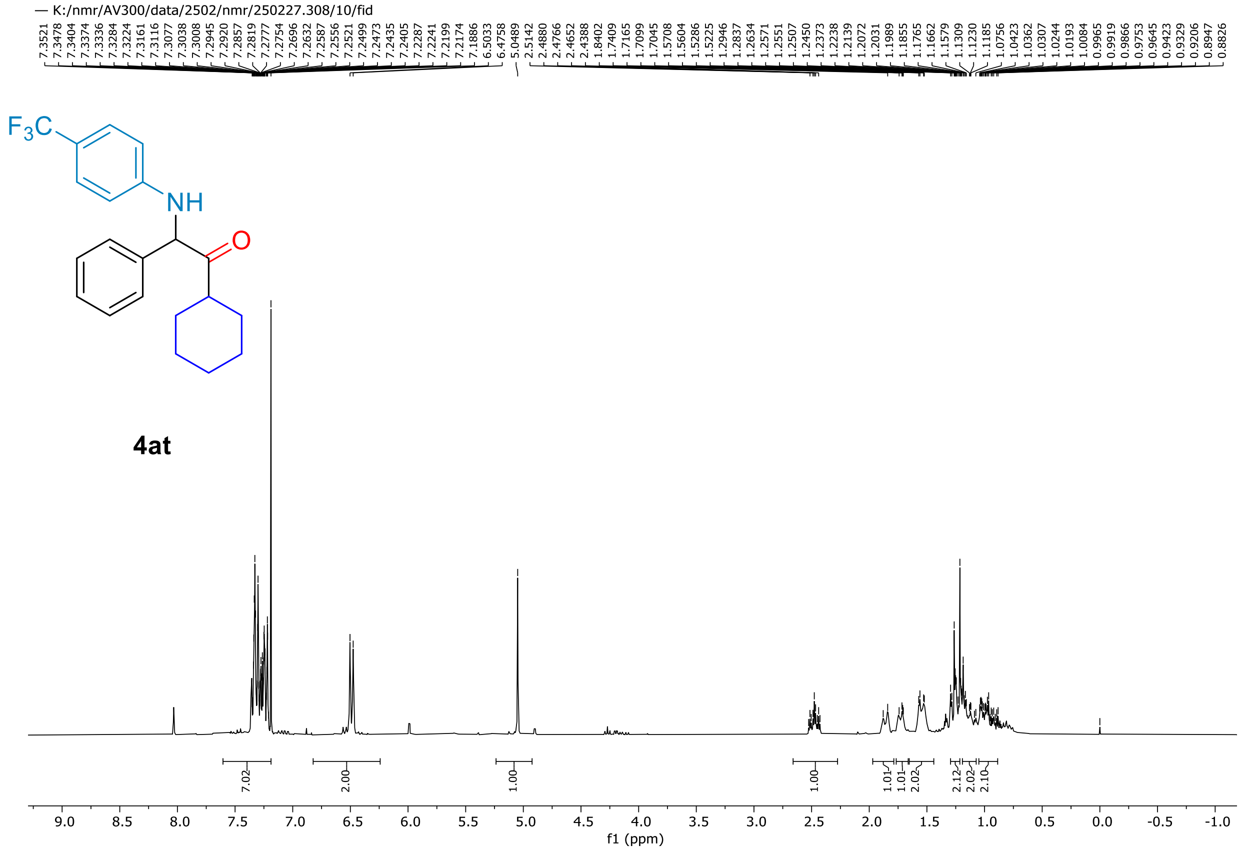

4at ^{13}C NMR (75 MHz, $CDCl_3$)

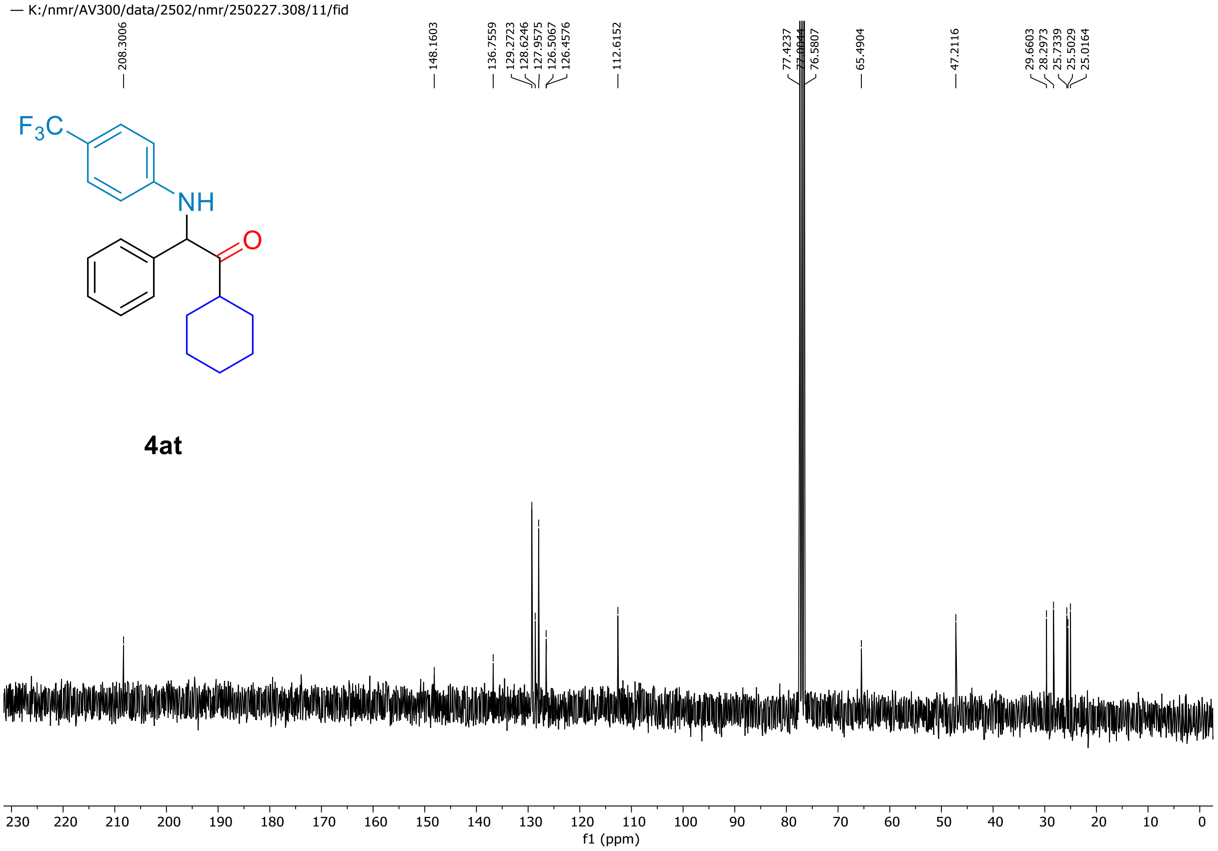

4at ^{19}F NMR (282 MHz, $CDCl_3$)

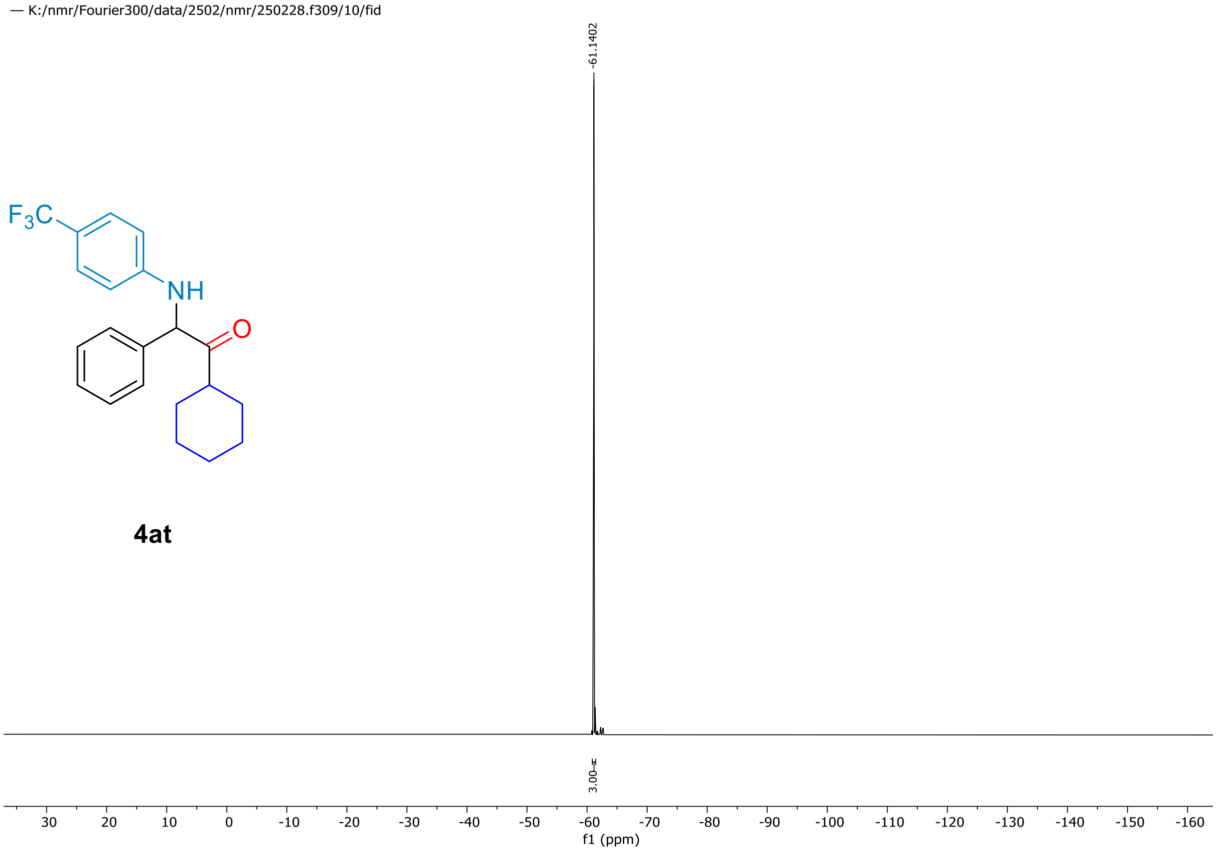

4at

4au ^{1}H NMR (300 MHz, $CDCl_3$)

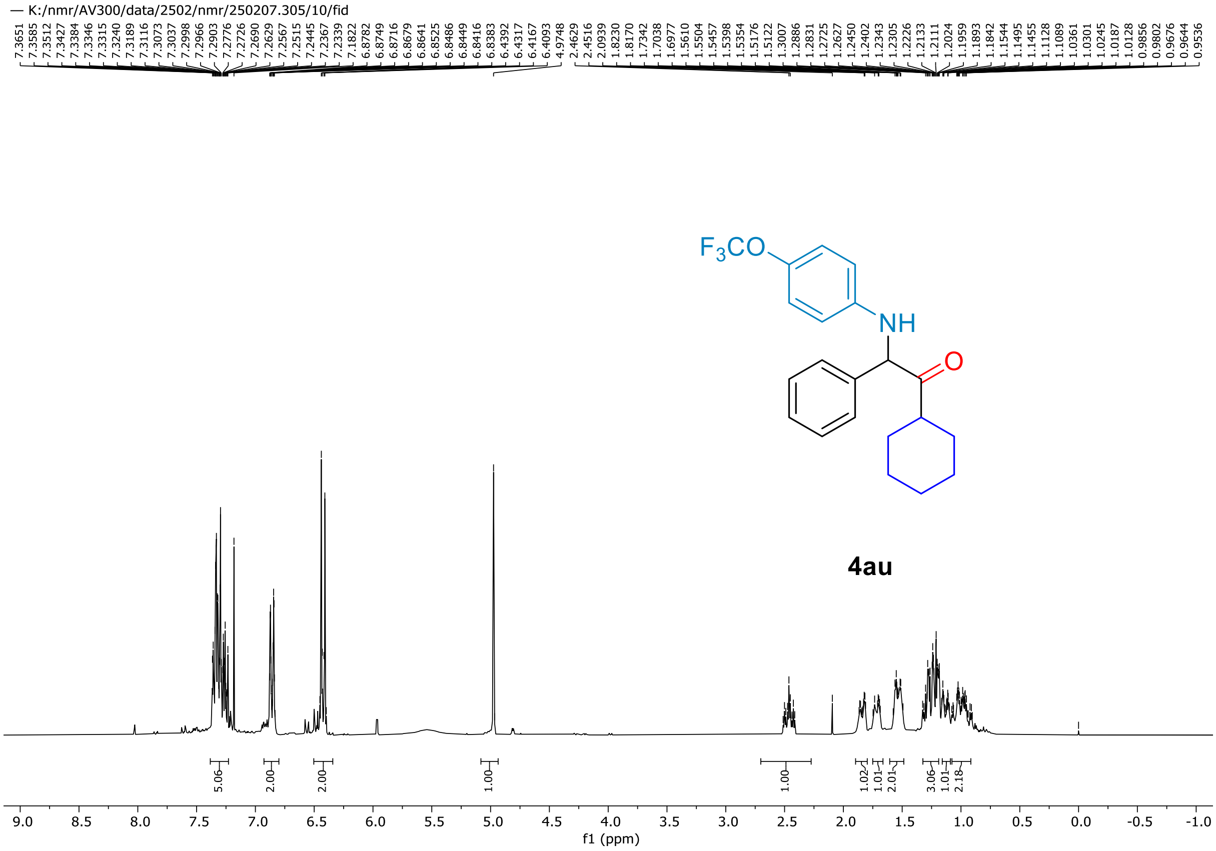

4au ^{13}C NMR (75 MHz, $CDCl_3$)

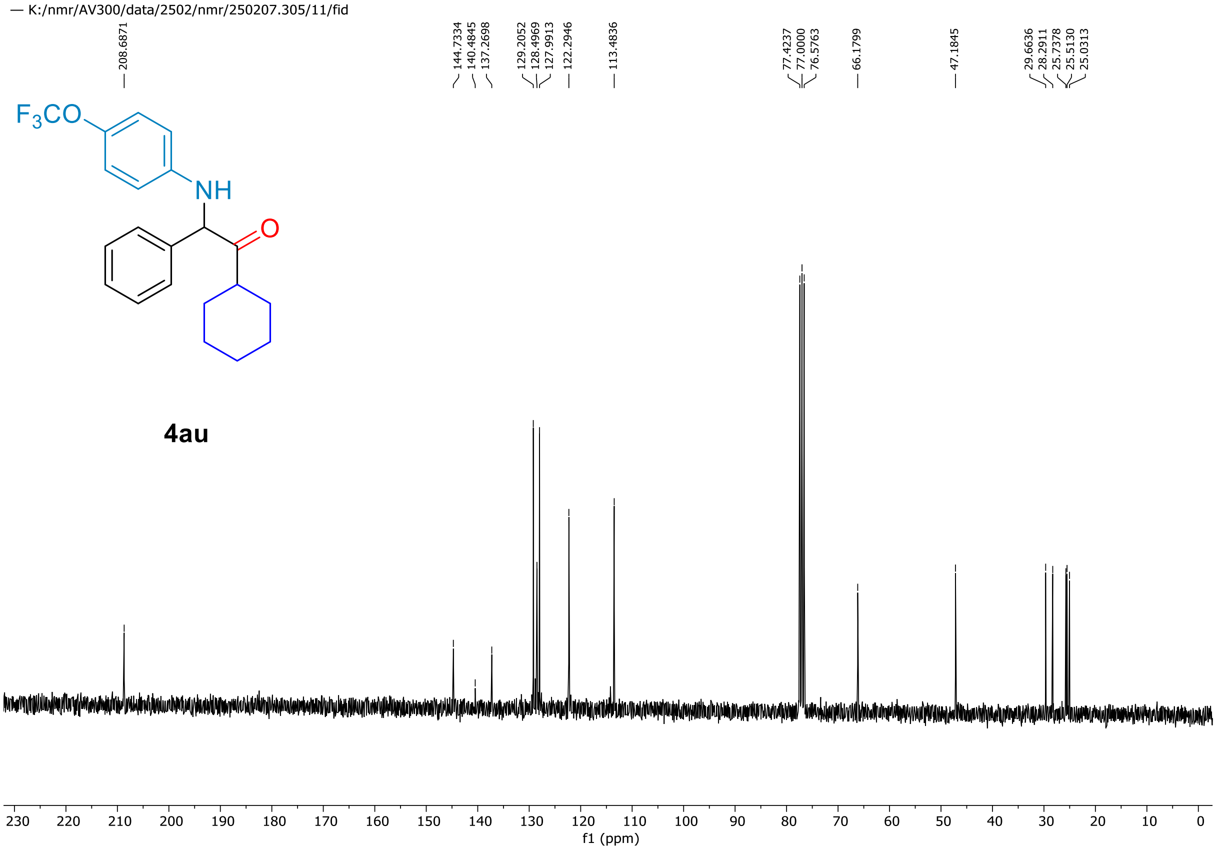

4au ^{19}F NMR (376 MHz, $CDCl_3$)

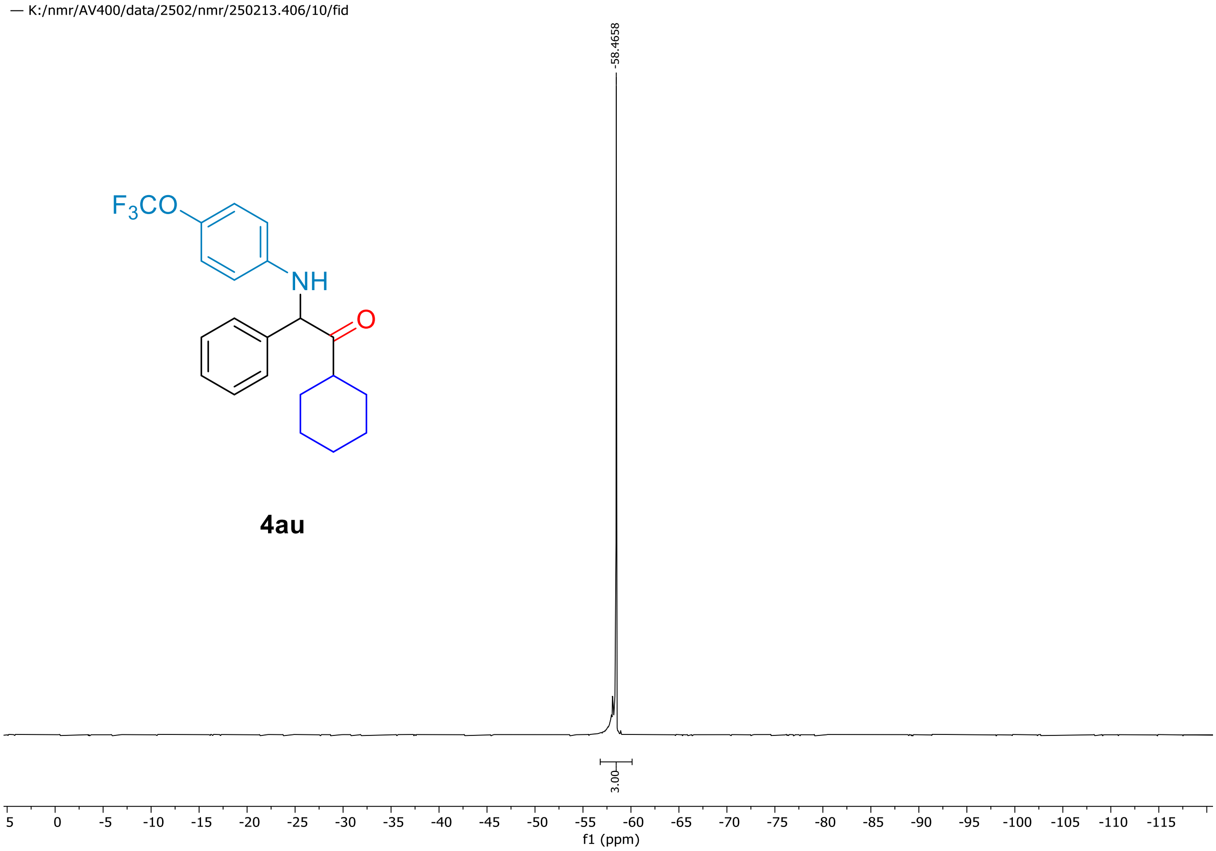

4av ^{1}H NMR (400 MHz, $CDCl_3$)

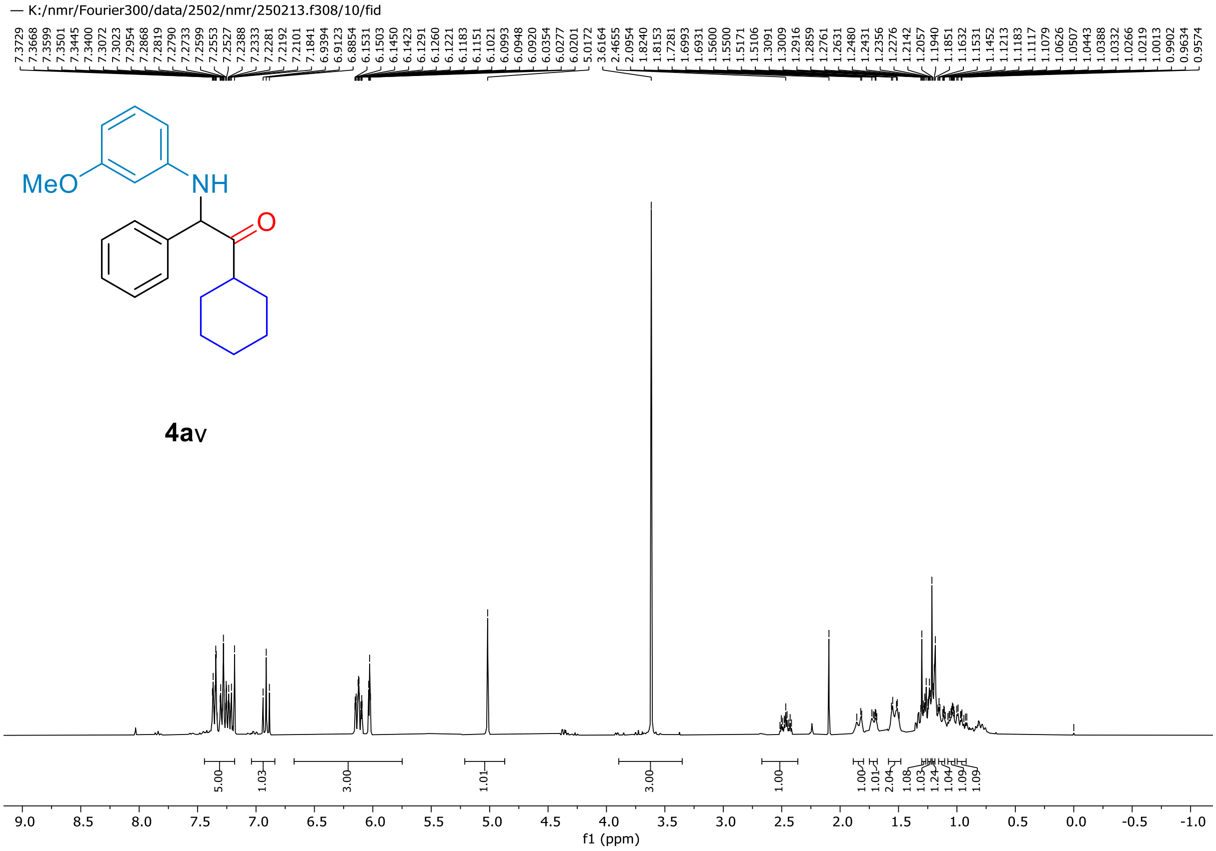

4av ^{13}C NMR (101 MHz, $CDCl_3$)

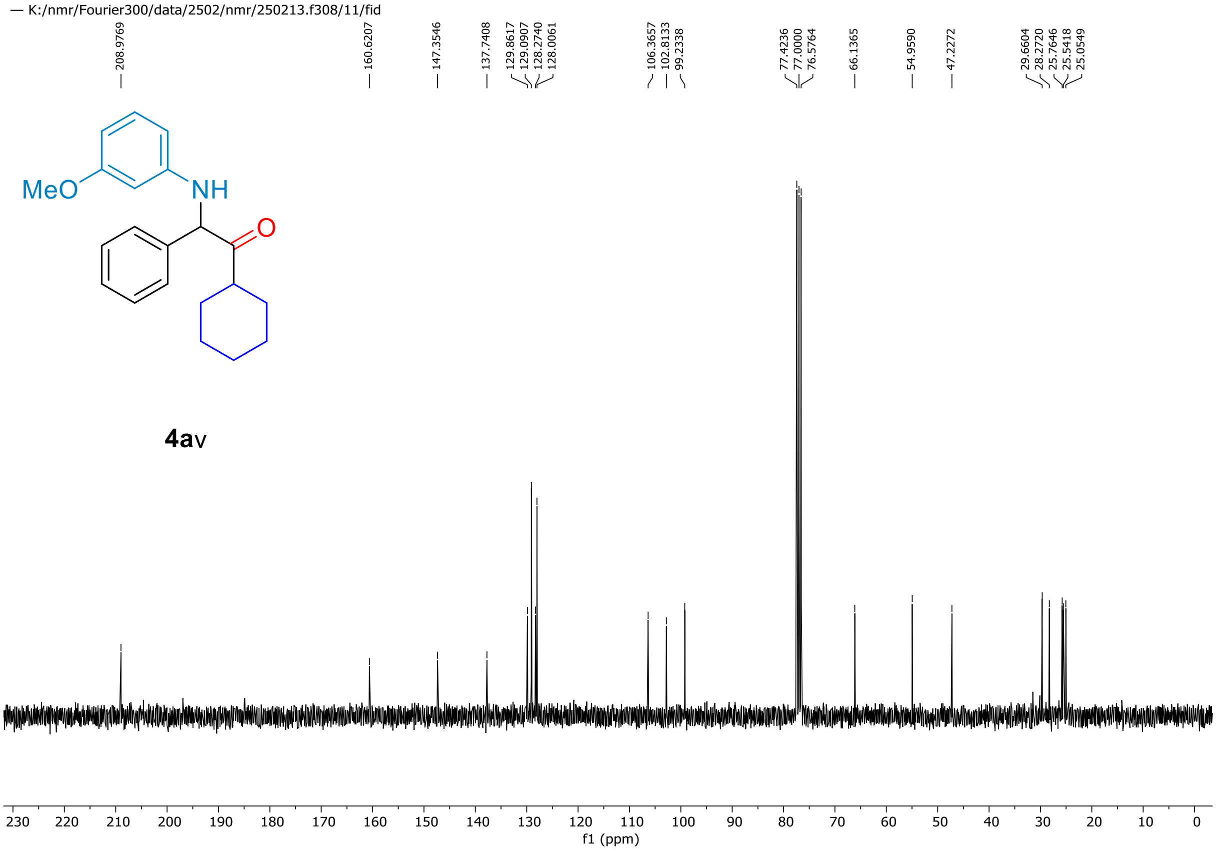

4aw ^{1}H NMR (300 MHz, $CDCl_3$)

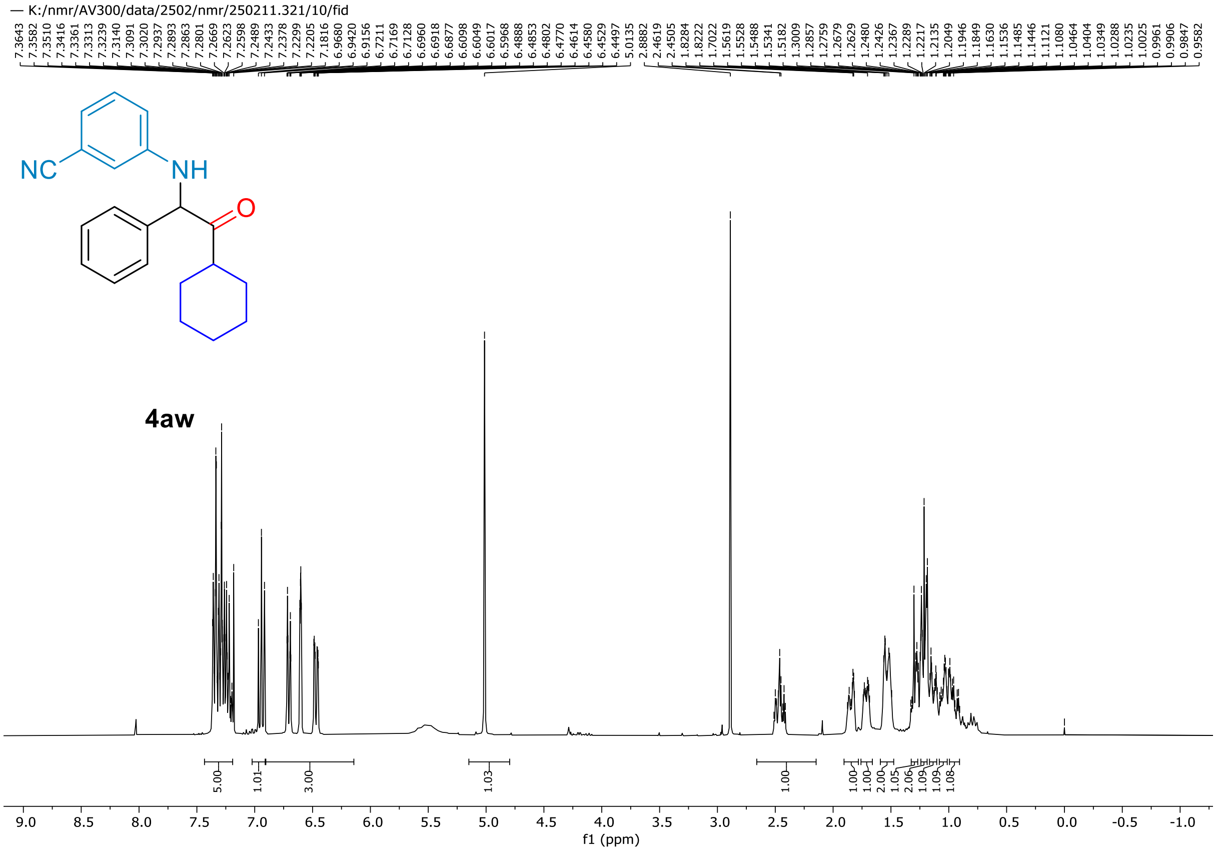

4aw ^{13}C NMR (75 MHz, $CDCl_3$)

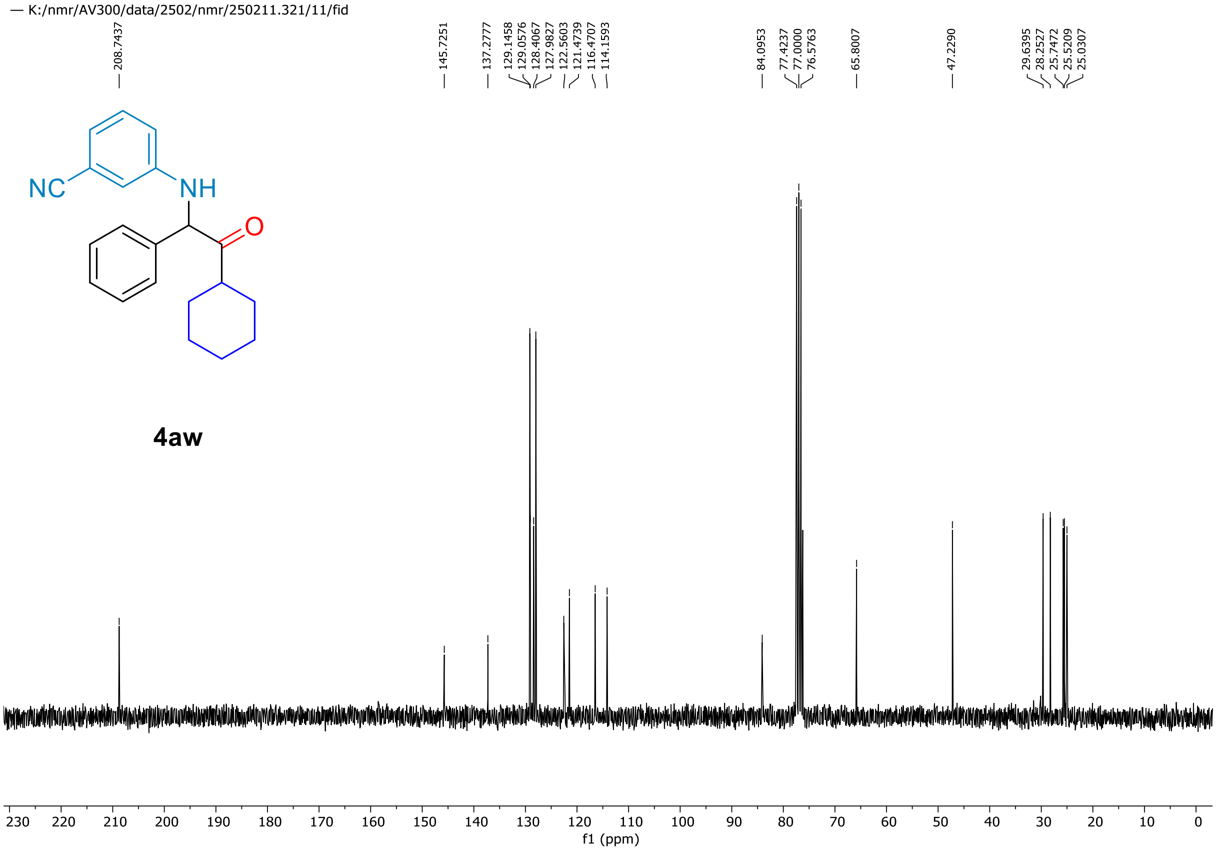

4ax ^{1}H NMR (400 MHz, $CDCl_3$)

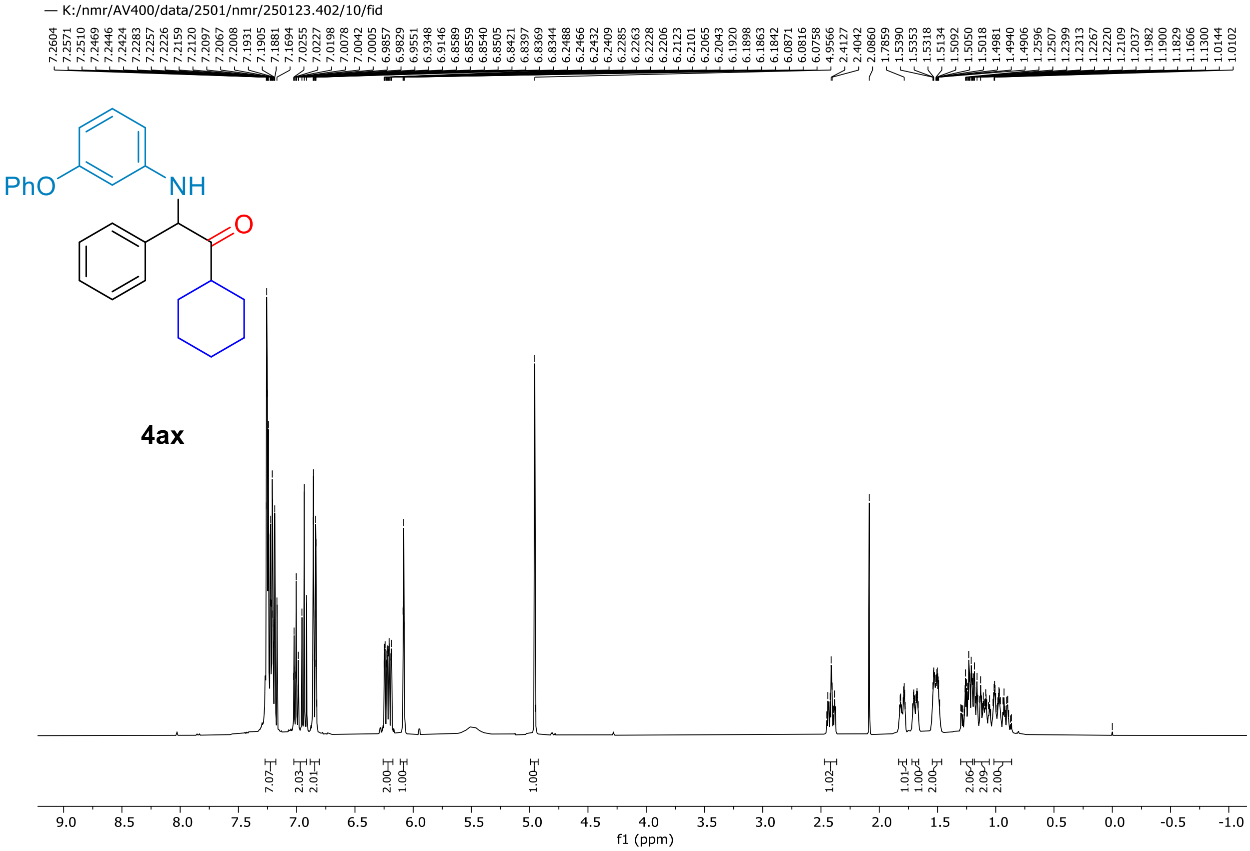

4ax ^{13}C NMR (101 MHz, $CDCl_3$)

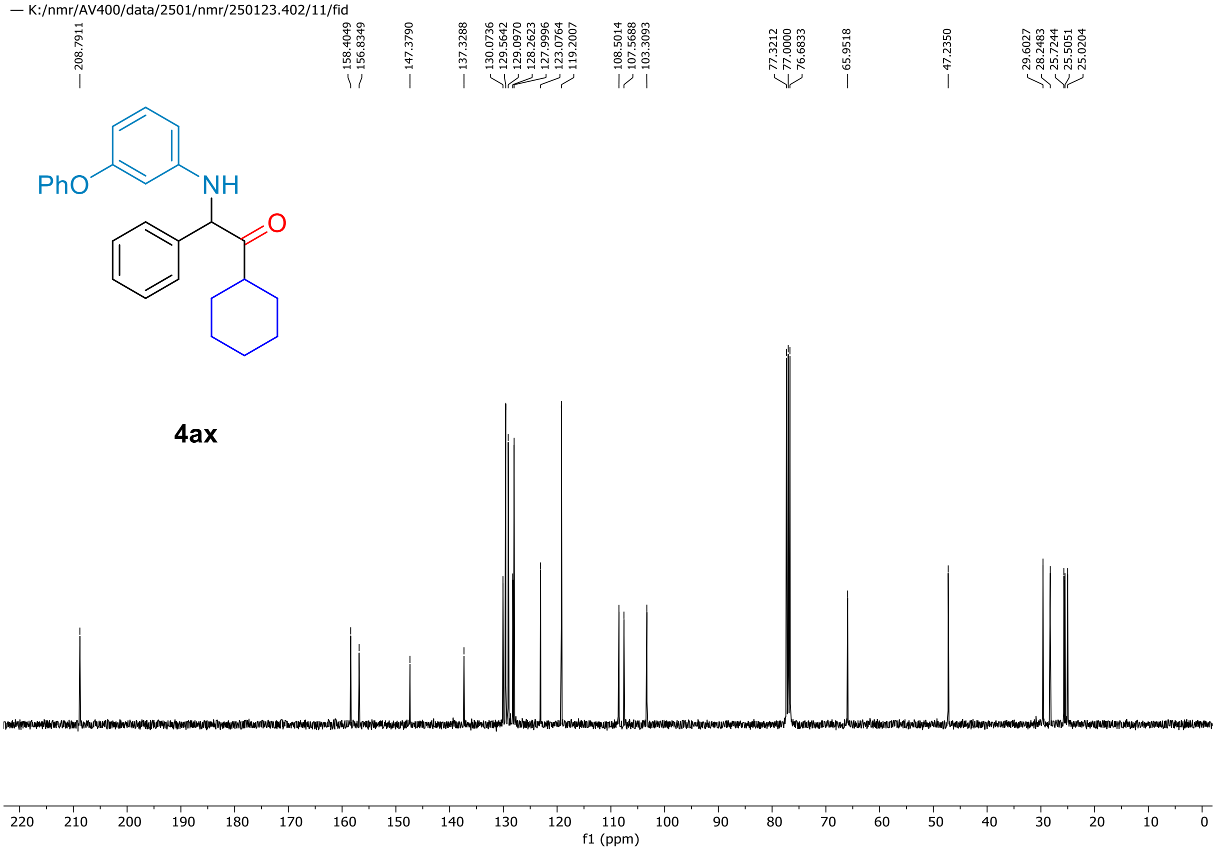

4ay ^{1}H NMR (300 MHz, $CDCl_3$)

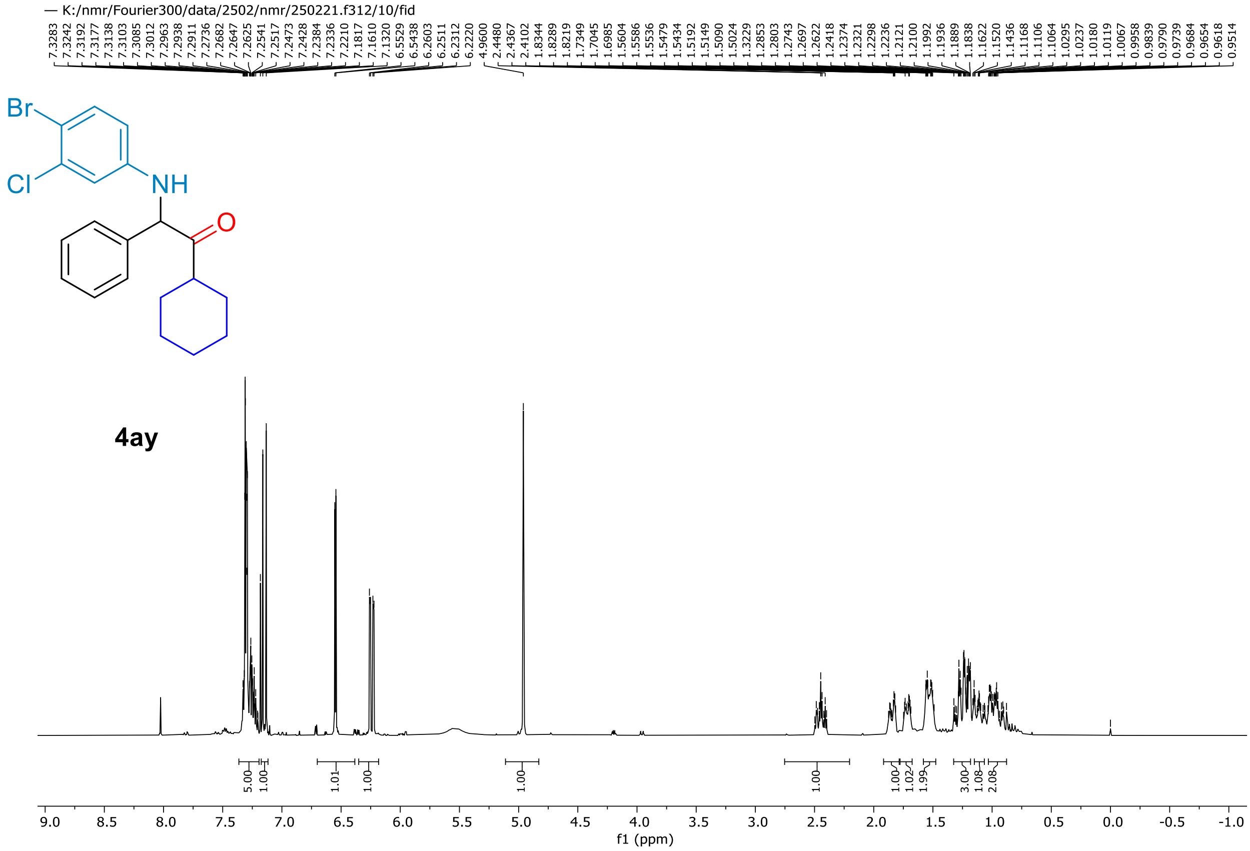

4ay ^{13}C NMR (75 MHz, $CDCl_3$)

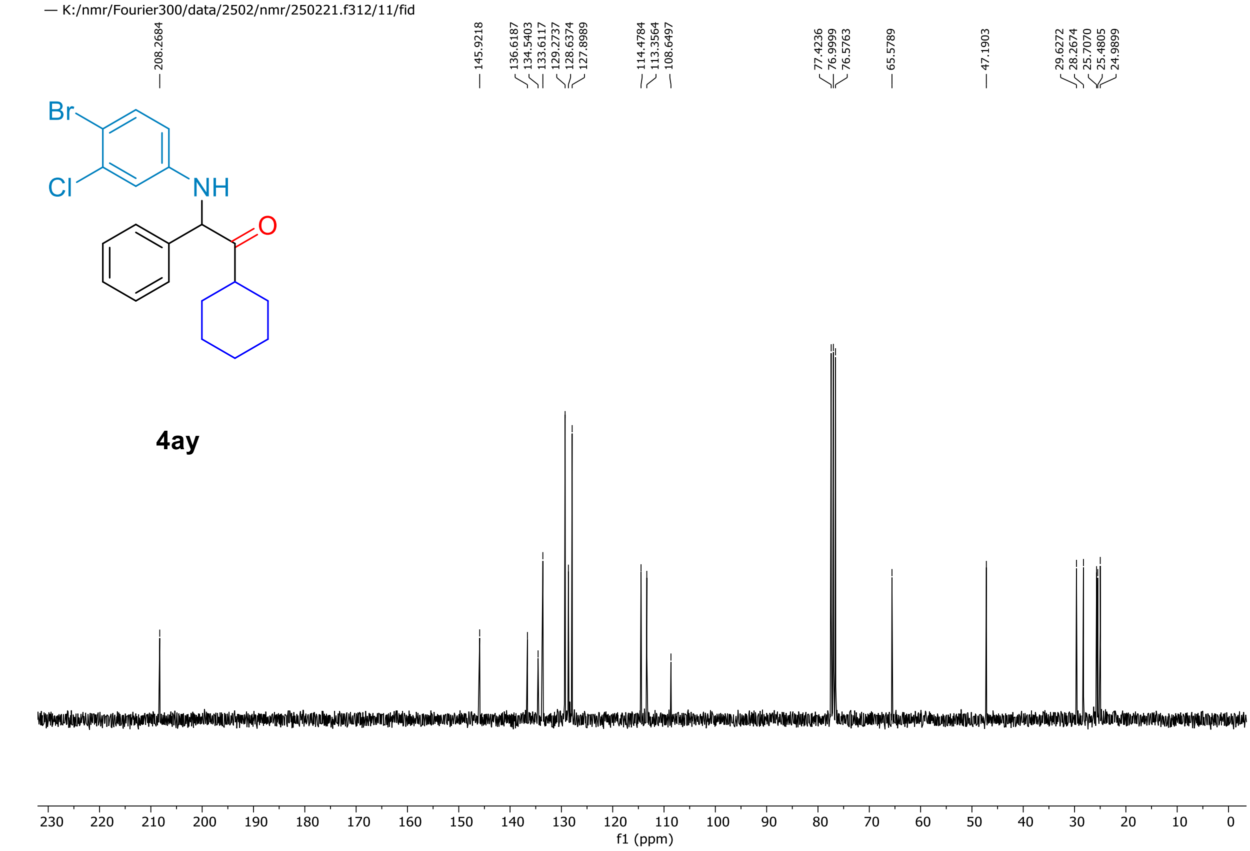

4az ^{1}H NMR (300 MHz, $CDCl_3$)

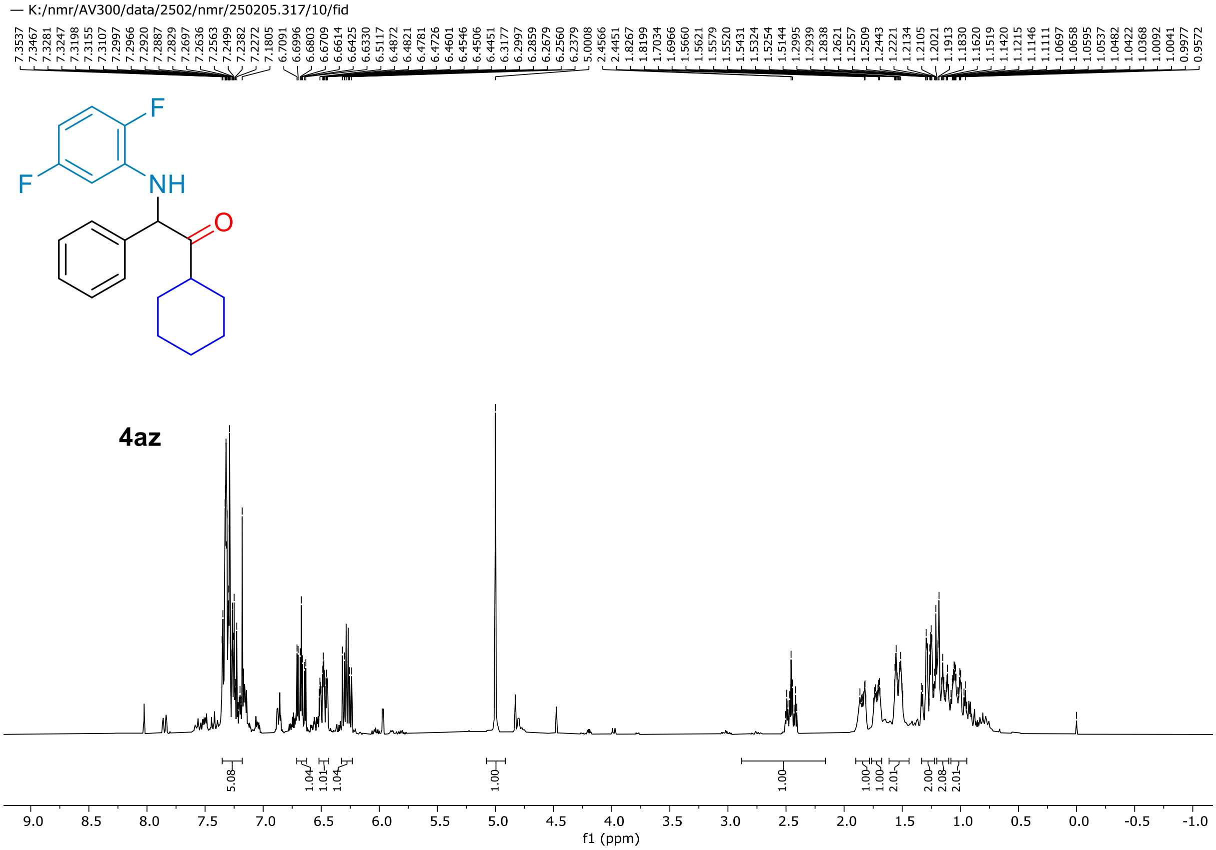

4az ^{13}C NMR (75 MHz, $CDCl_3$)

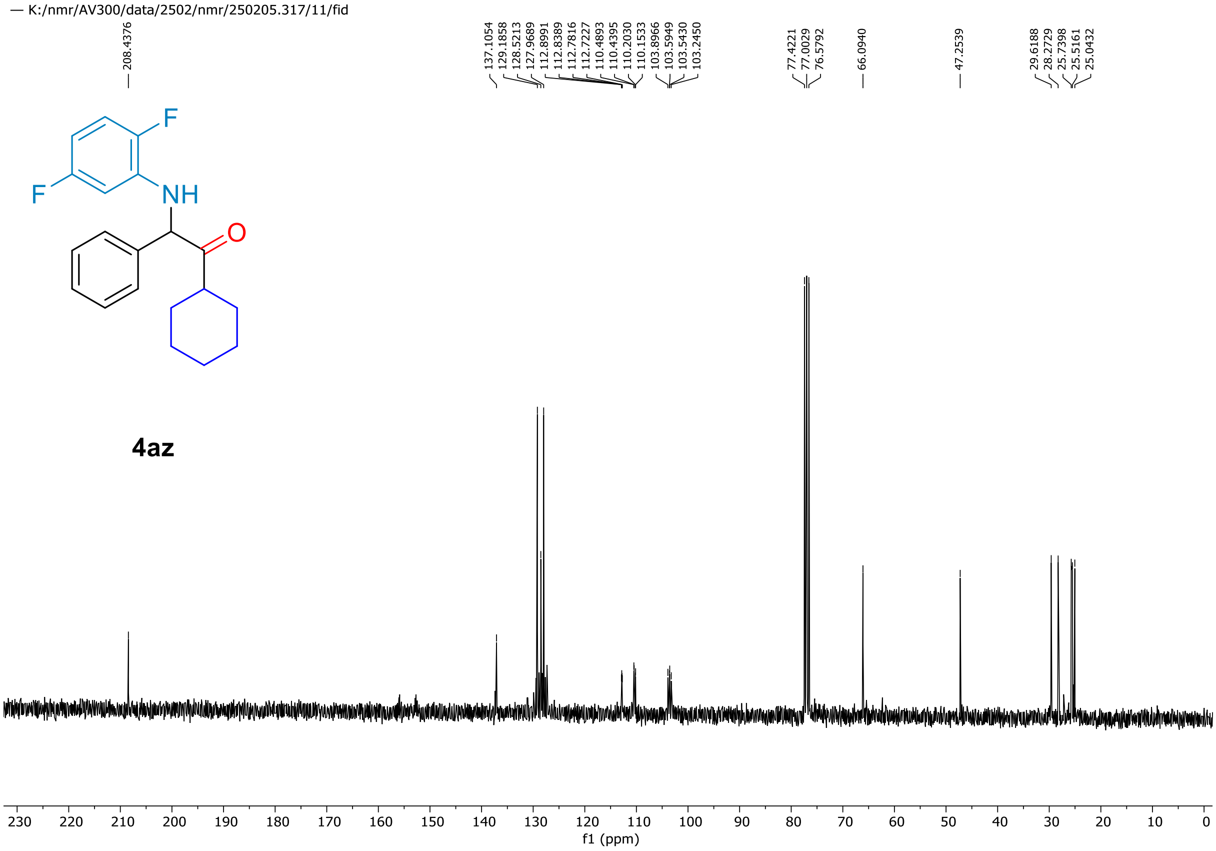

4az ^{19}F NMR (282 MHz, $CDCl_3$)

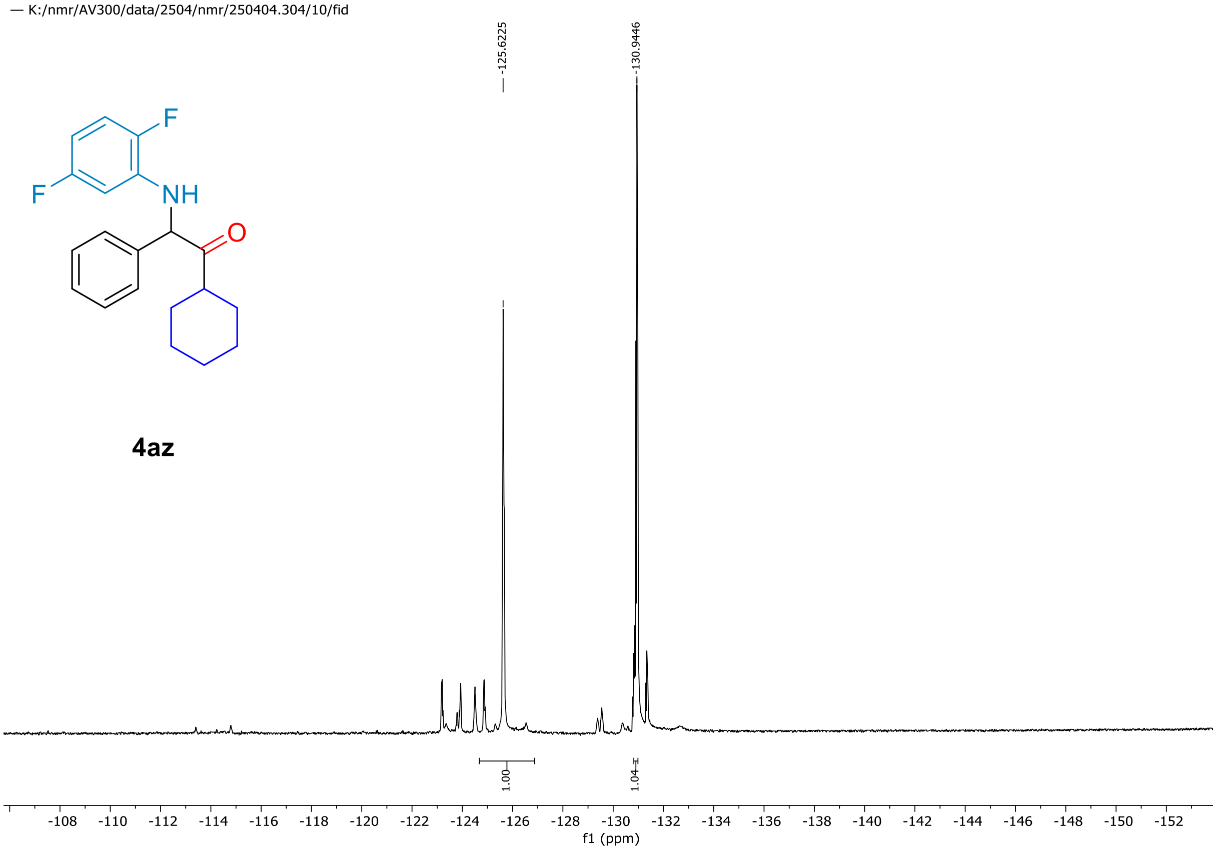

4ba ^{1}H NMR (300 MHz, $CDCl_3$)

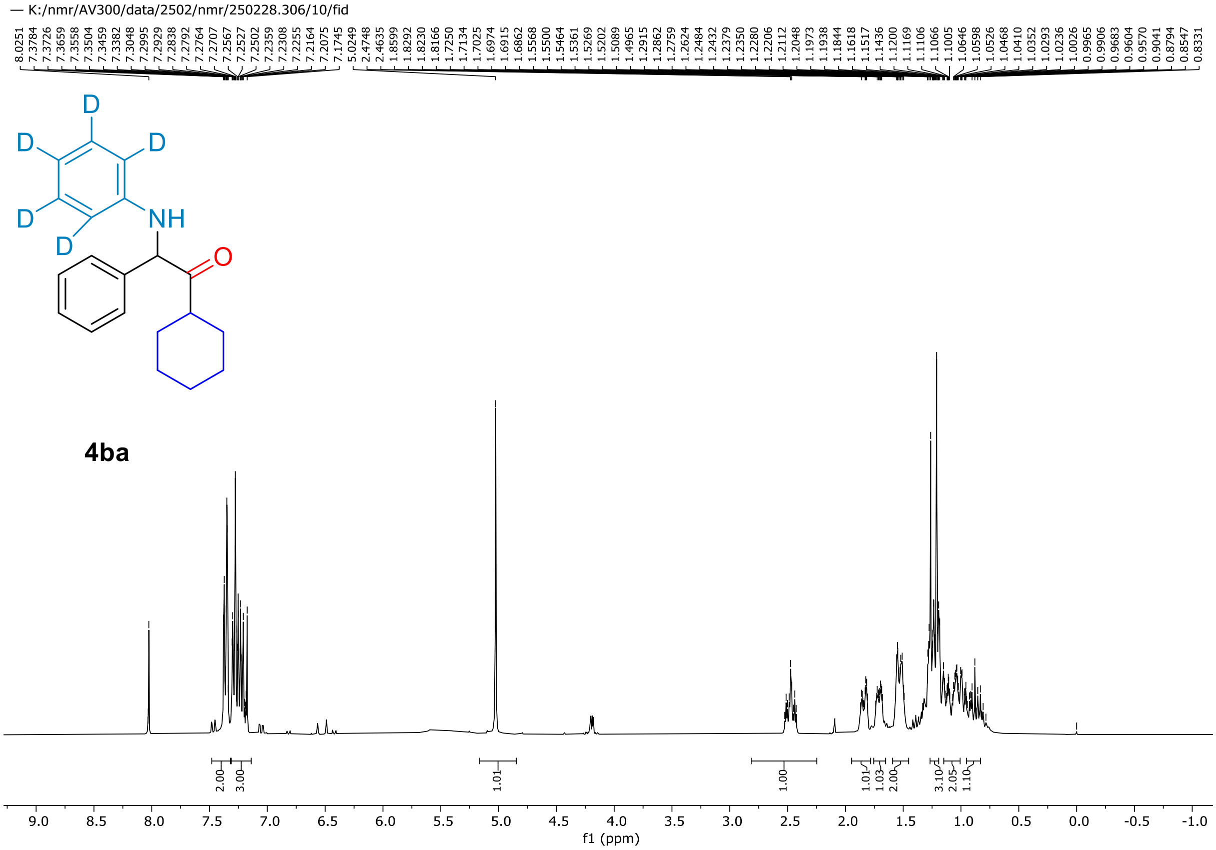

4ba ^{13}C NMR (75 MHz, $CDCl_3$)

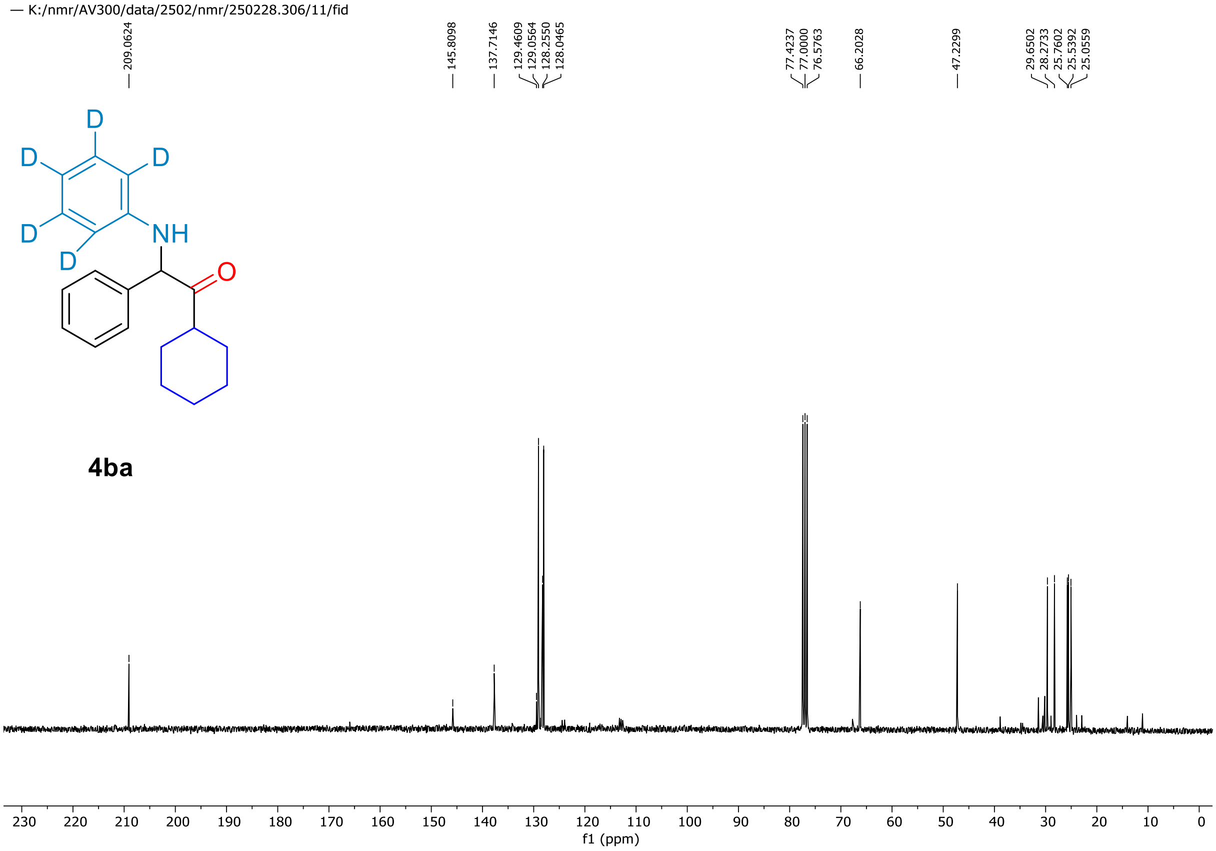

4bb ^1H NMR (400 MHz, $CDCl_3$)

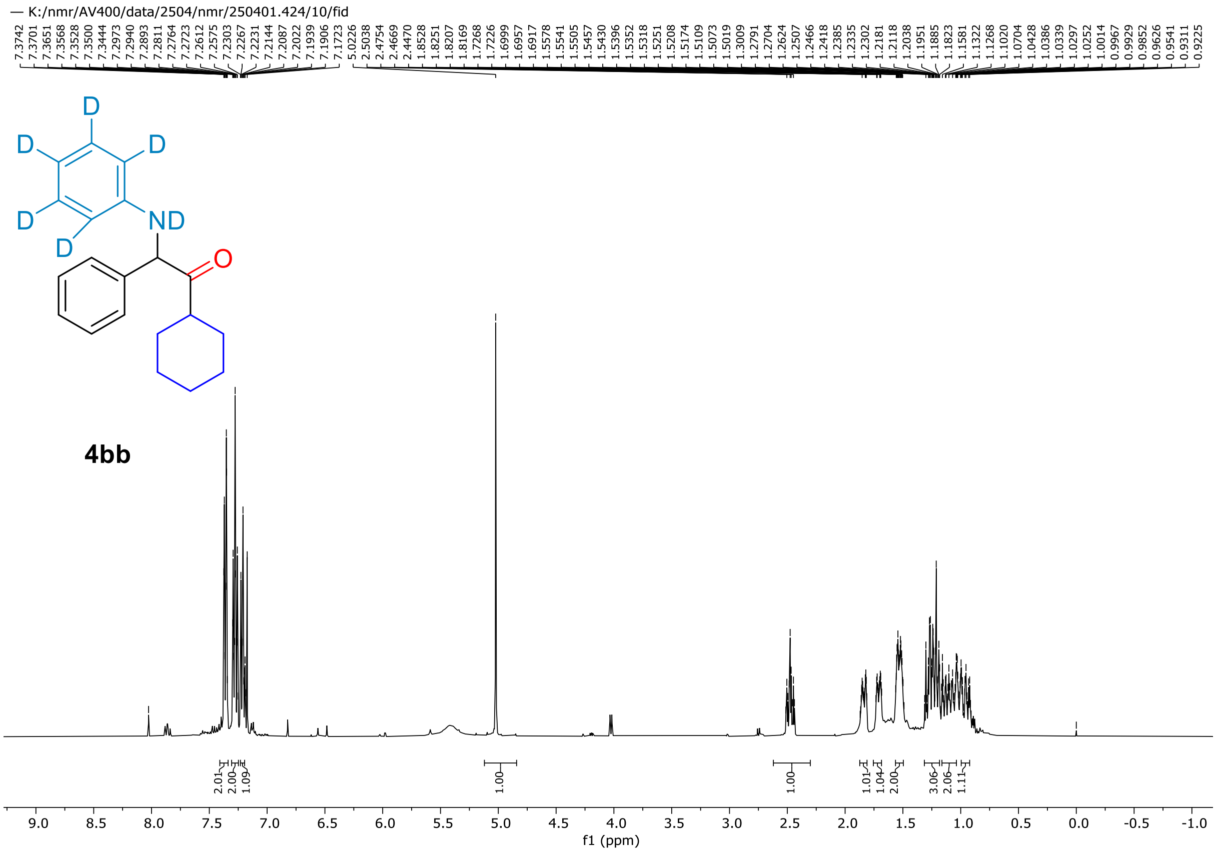

4bb ^{13}C NMR (101 MHz, $CDCl_3$)

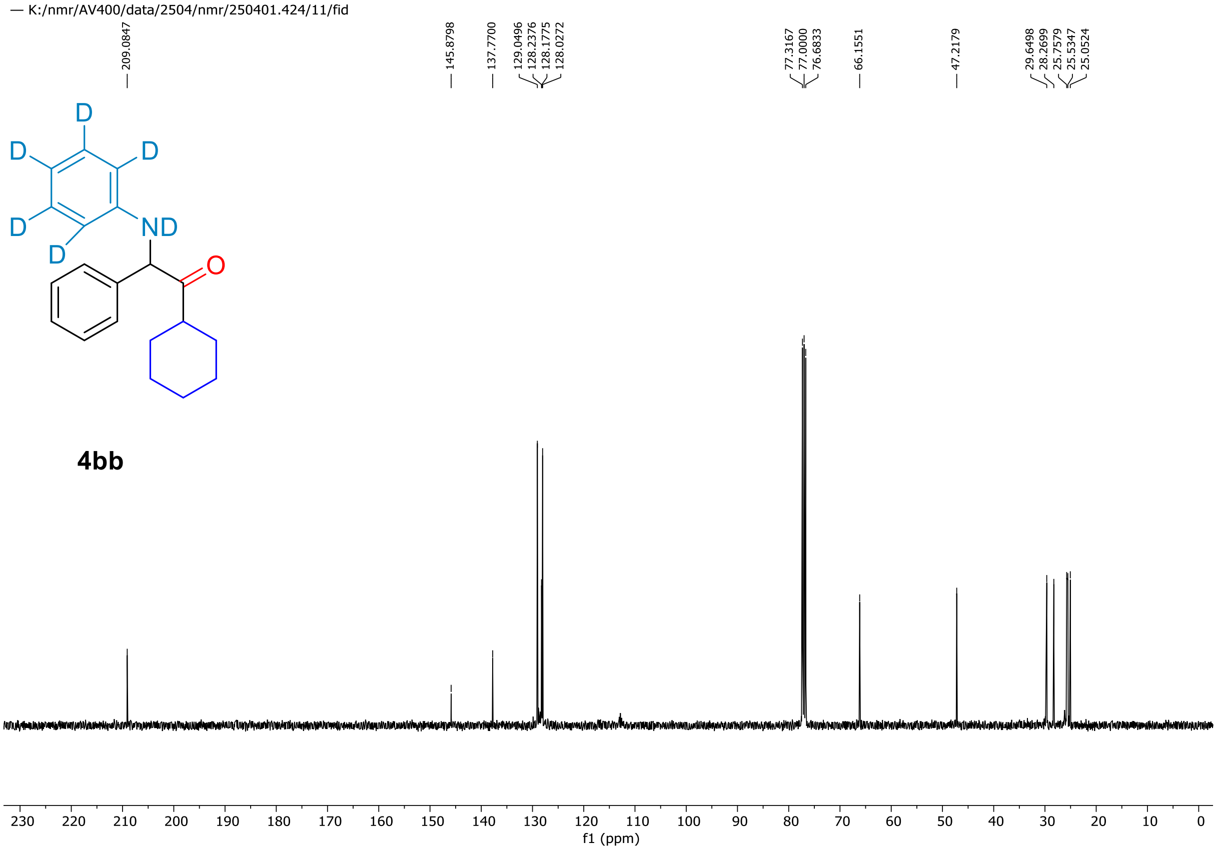

4bc ^{1}H NMR (300 MHz, $CDCl_3$)

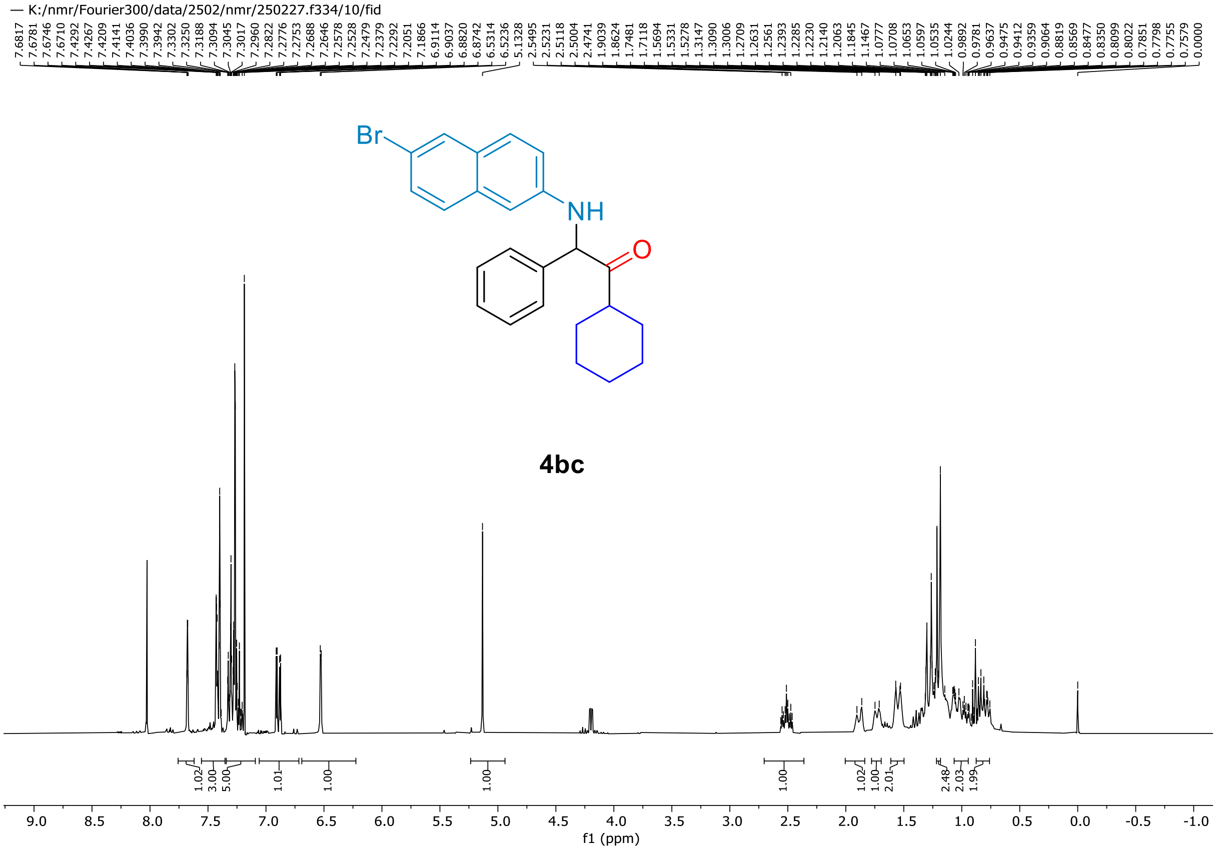

4bc ^{13}C NMR (75 MHz, $CDCl_3$)

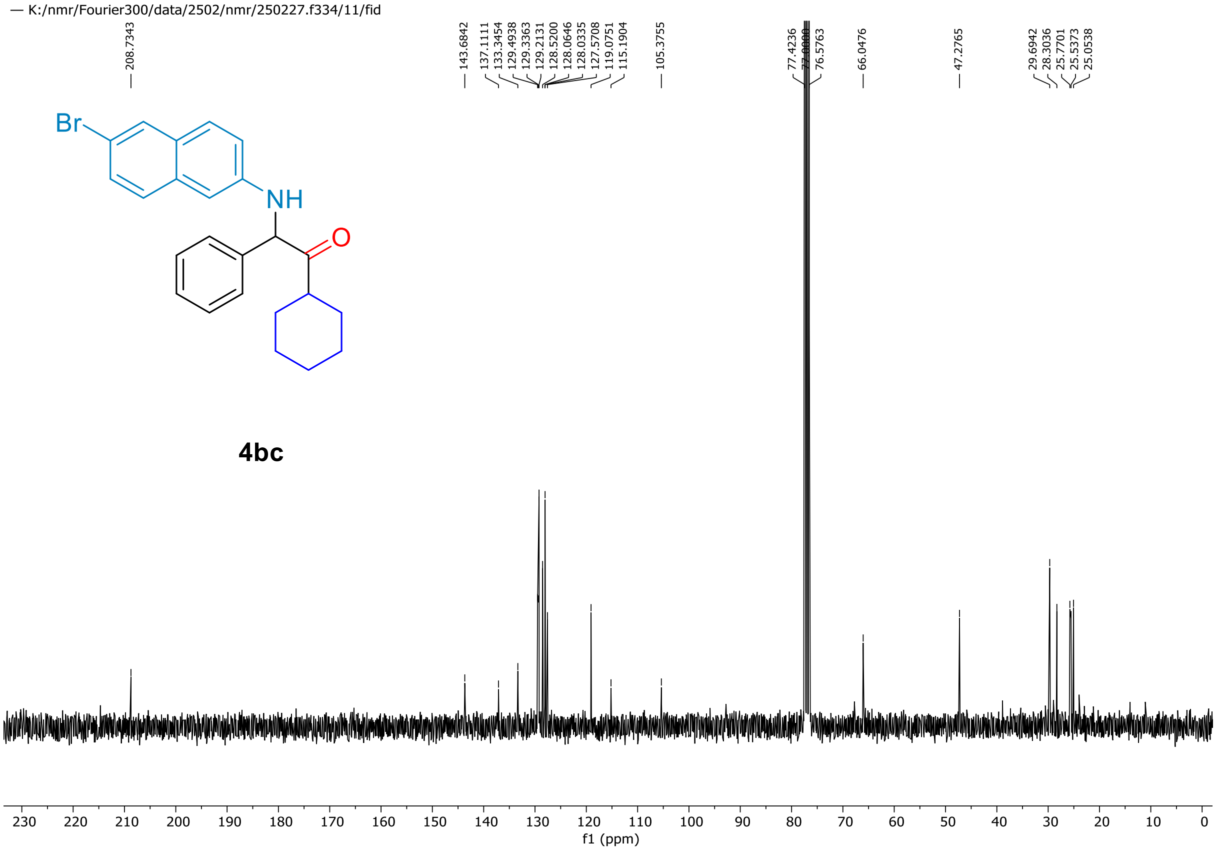

4bd ^{1}H NMR (400 MHz, $CDCl_3$)

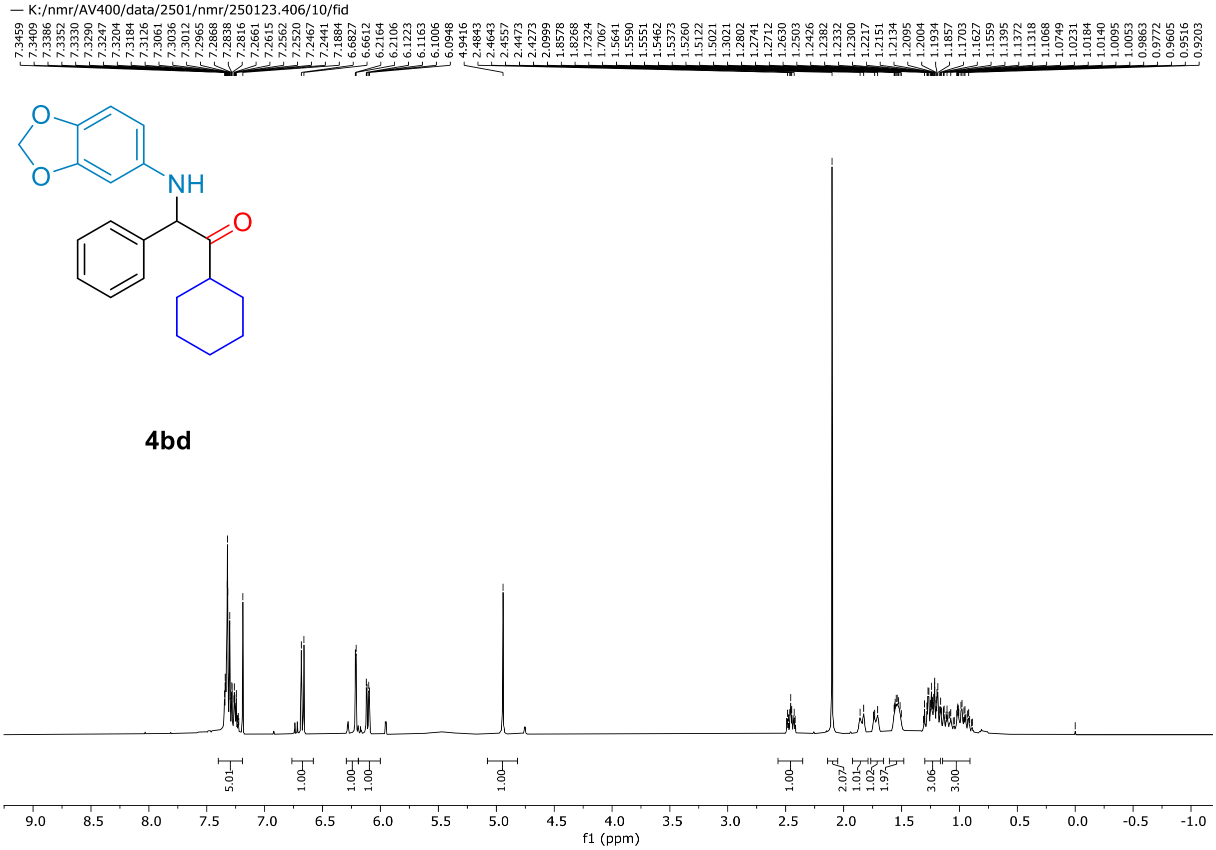

4bd ^{13}C NMR (101 MHz, $CDCl_3$)

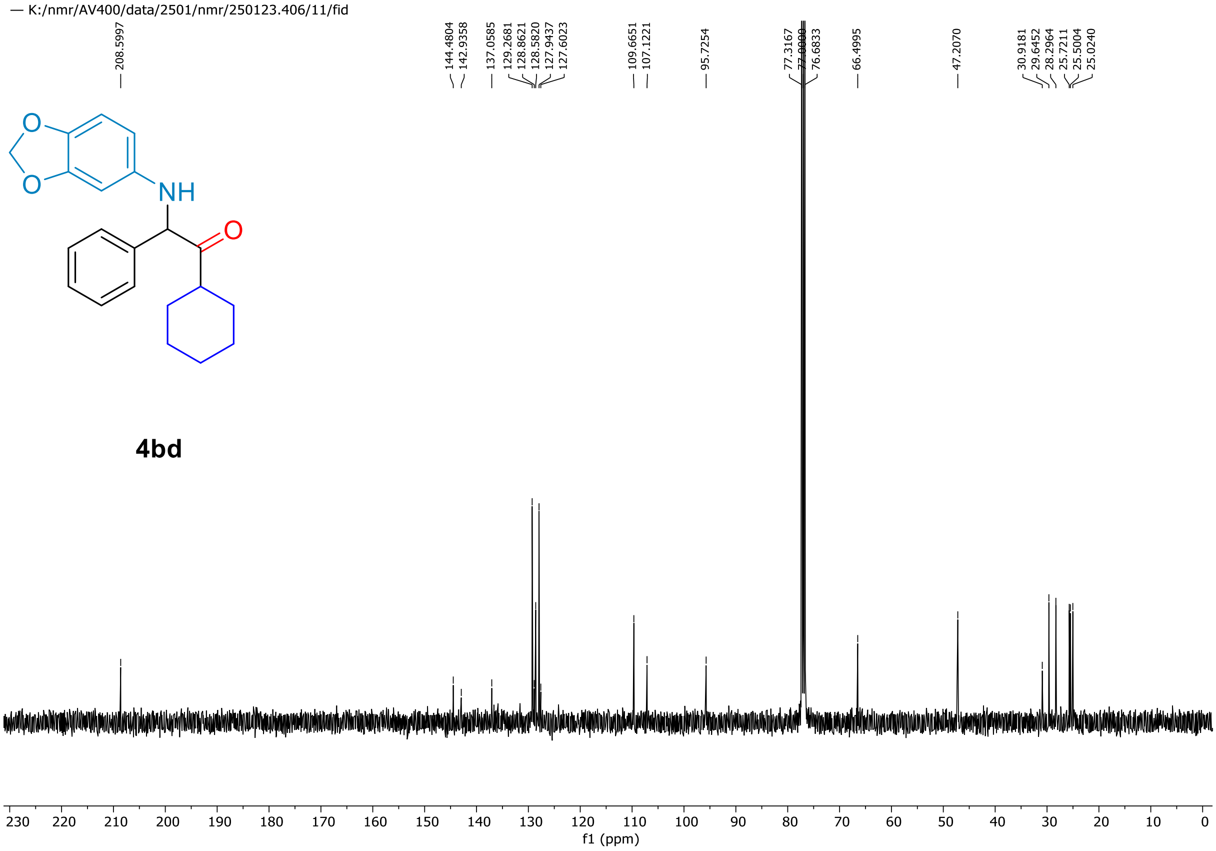

4be ^{1}H NMR (300 MHz, $CDCl_3$)

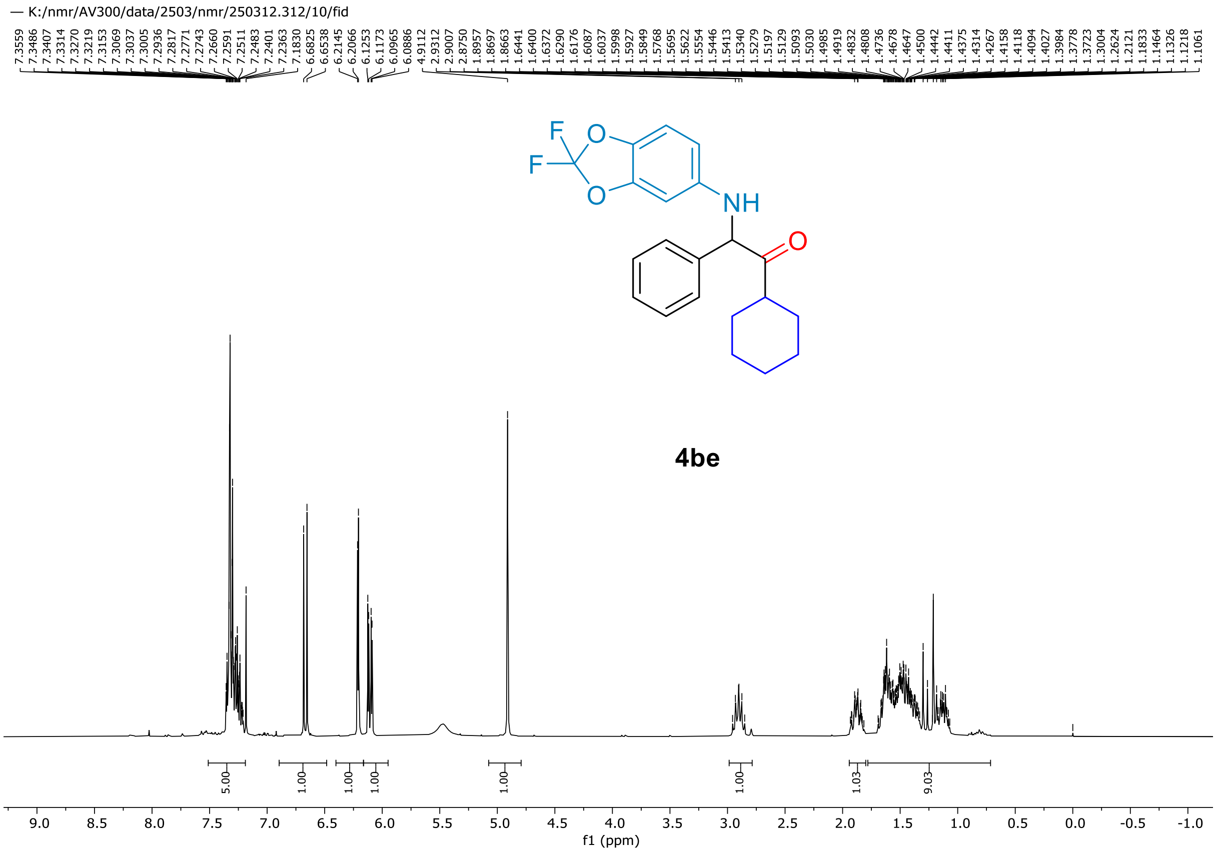

4be ^{13}C NMR (75 MHz, $CDCl_3$)

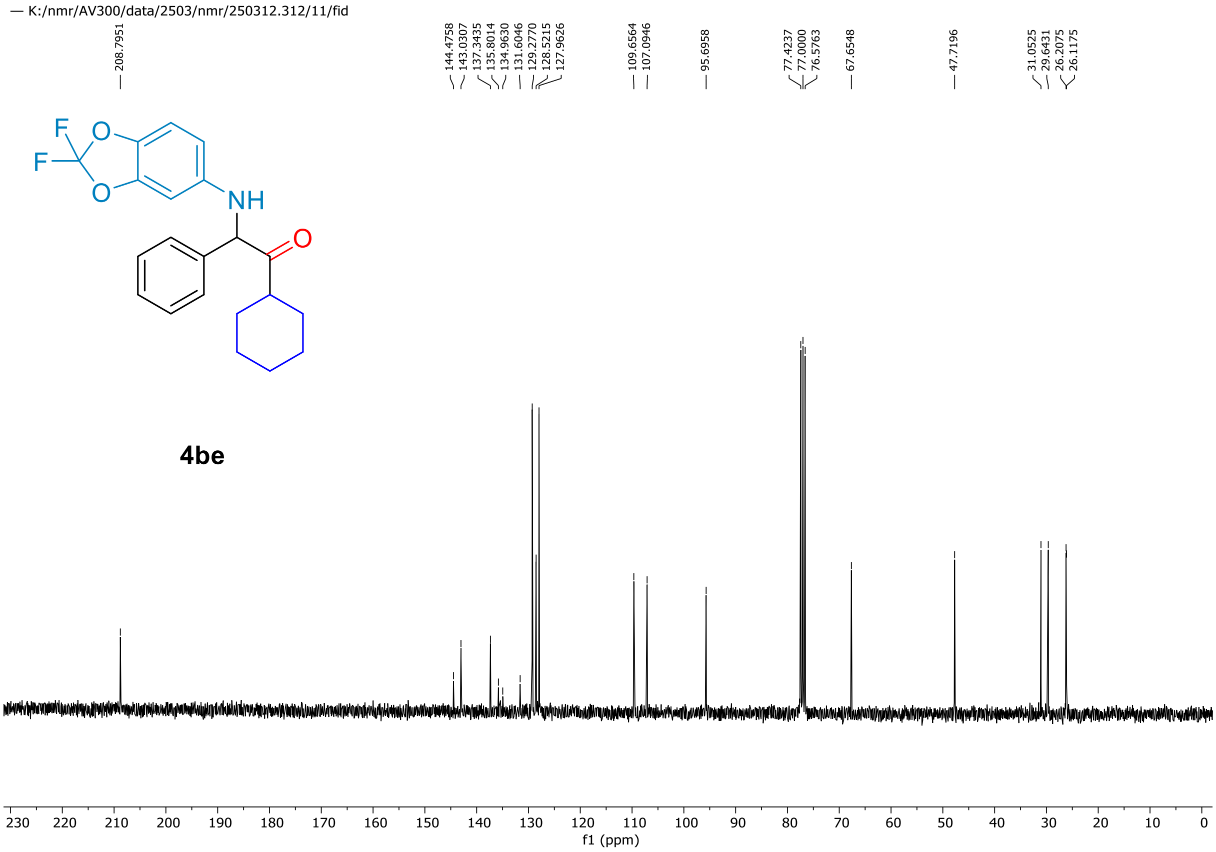

4be ^{13}C NMR (282 MHz, $CDCl_3$)

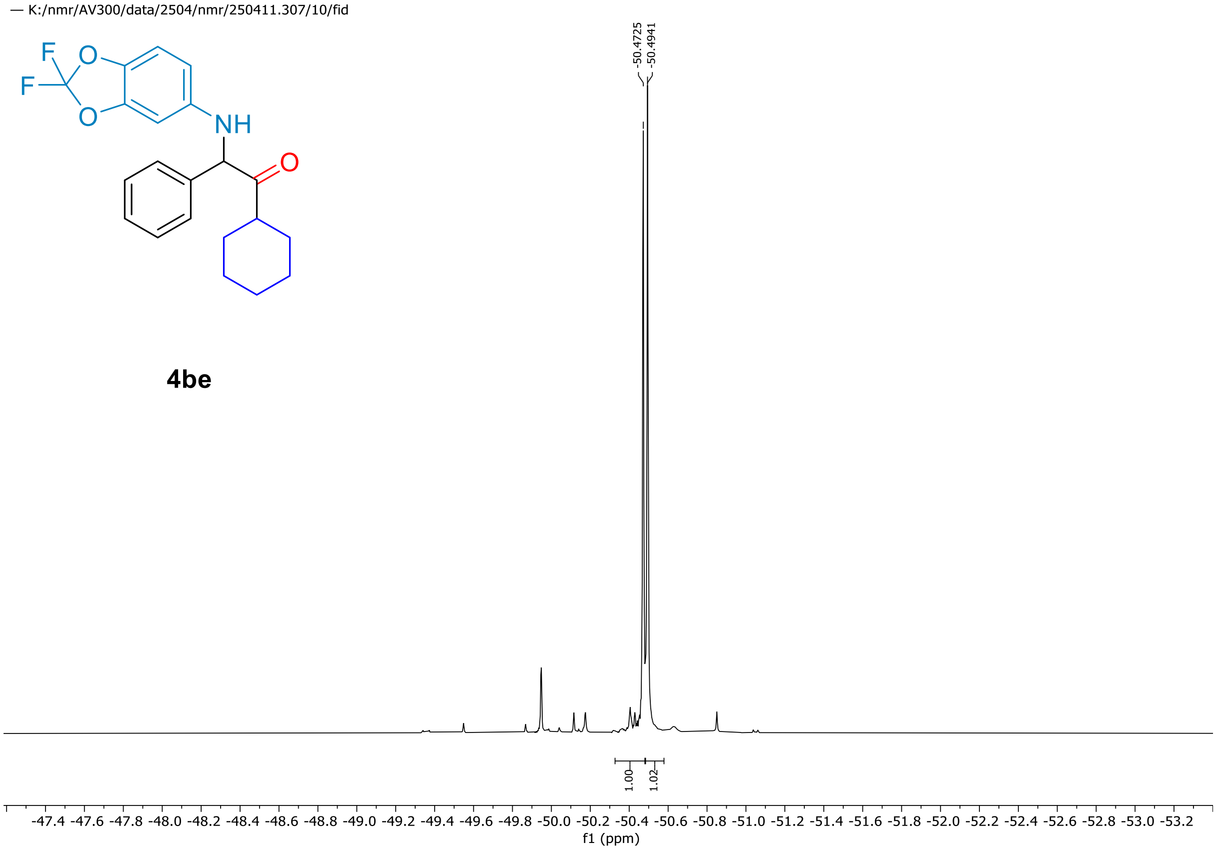

4bf ^{1}H NMR (400 MHz, $CDCl_3$)

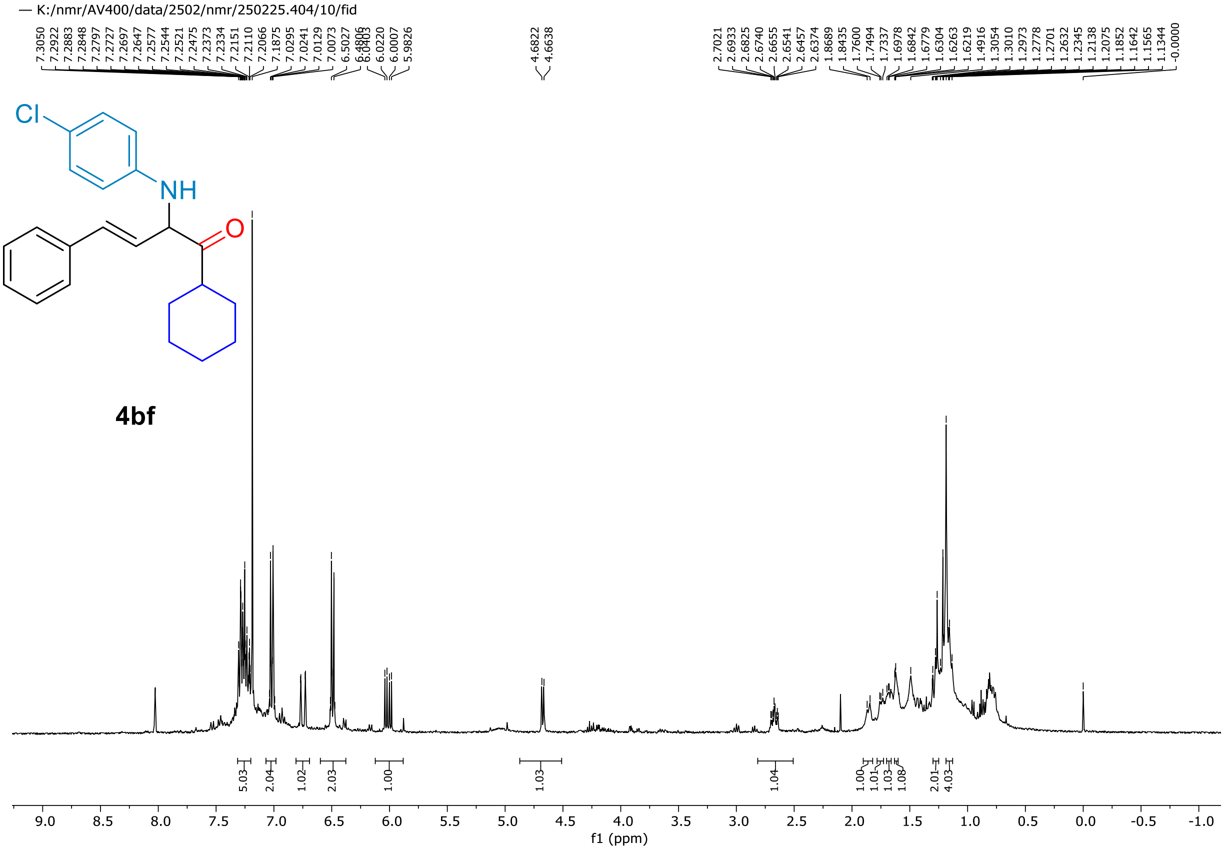

4bf ^{13}C NMR (101 MHz, $CDCl_3$)

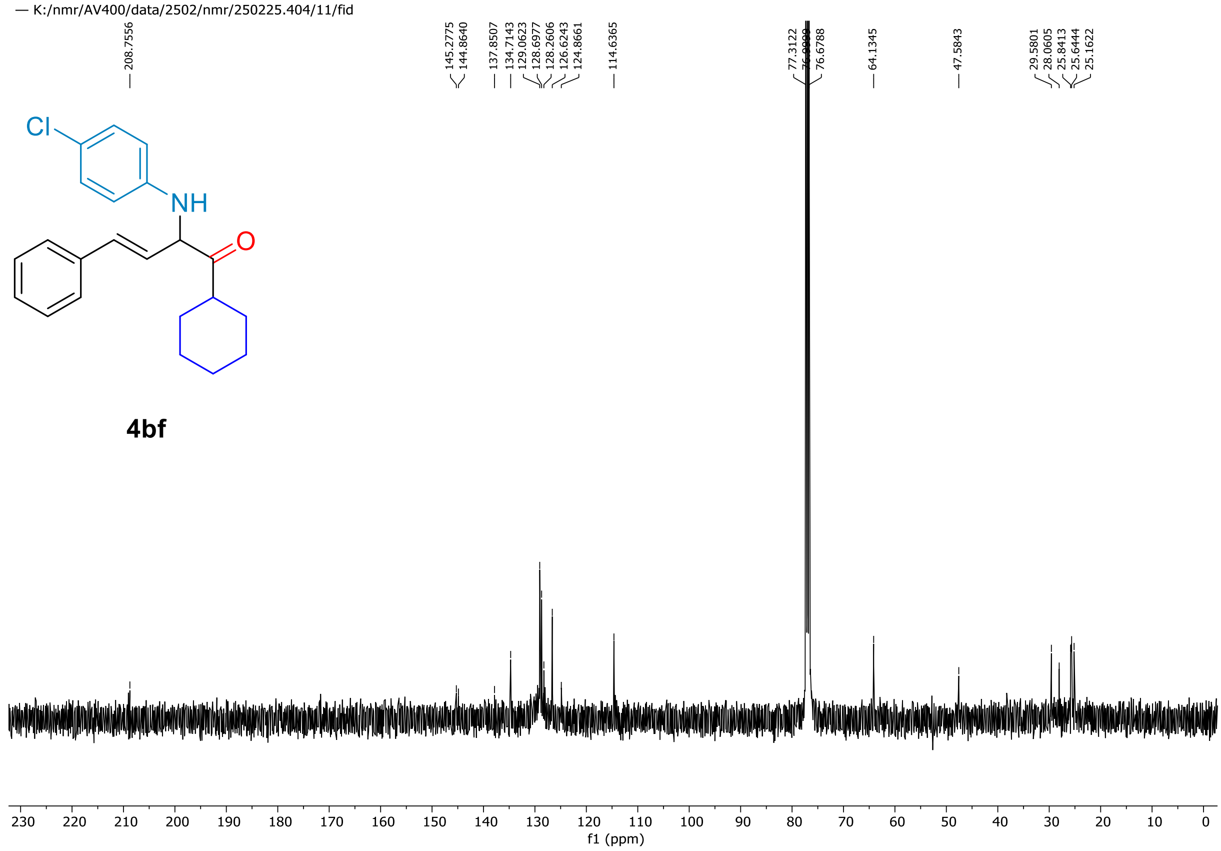

4bg ^{1}H NMR (300 MHz, $CDCl_3$)

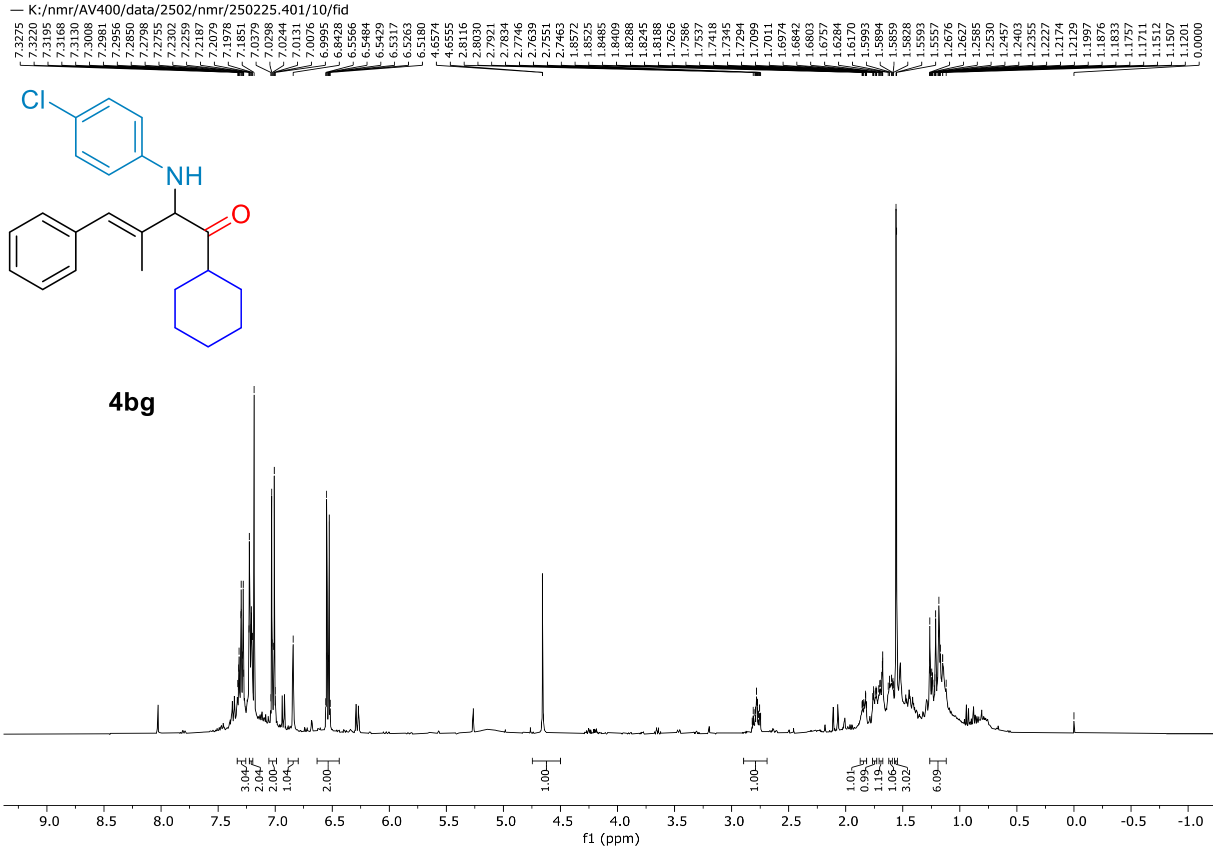

4bg ^{13}C NMR (101 MHz, $CDCl_3$)

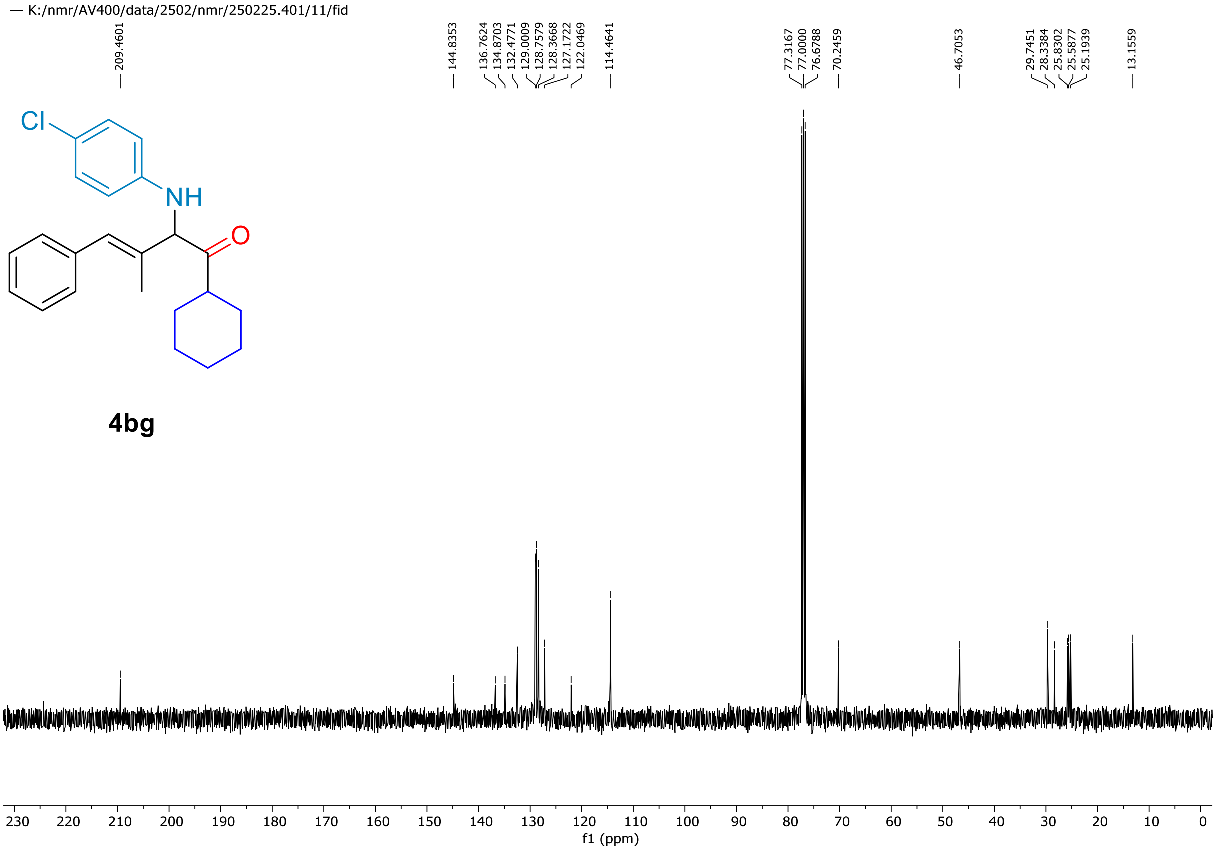

4bh ^{1}H NMR (300 MHz, $CDCl_3$)

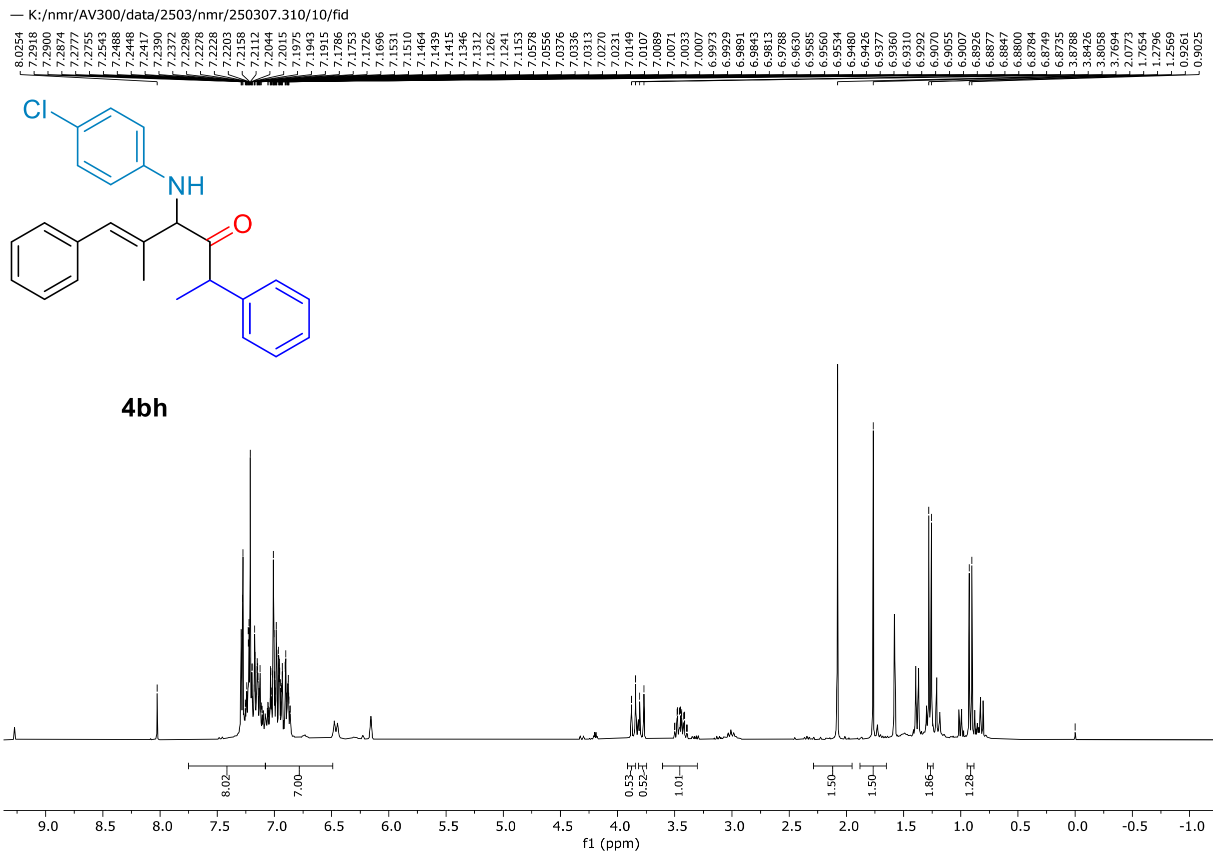

4bh ^{13}C NMR (101 MHz, $CDCl_3$)

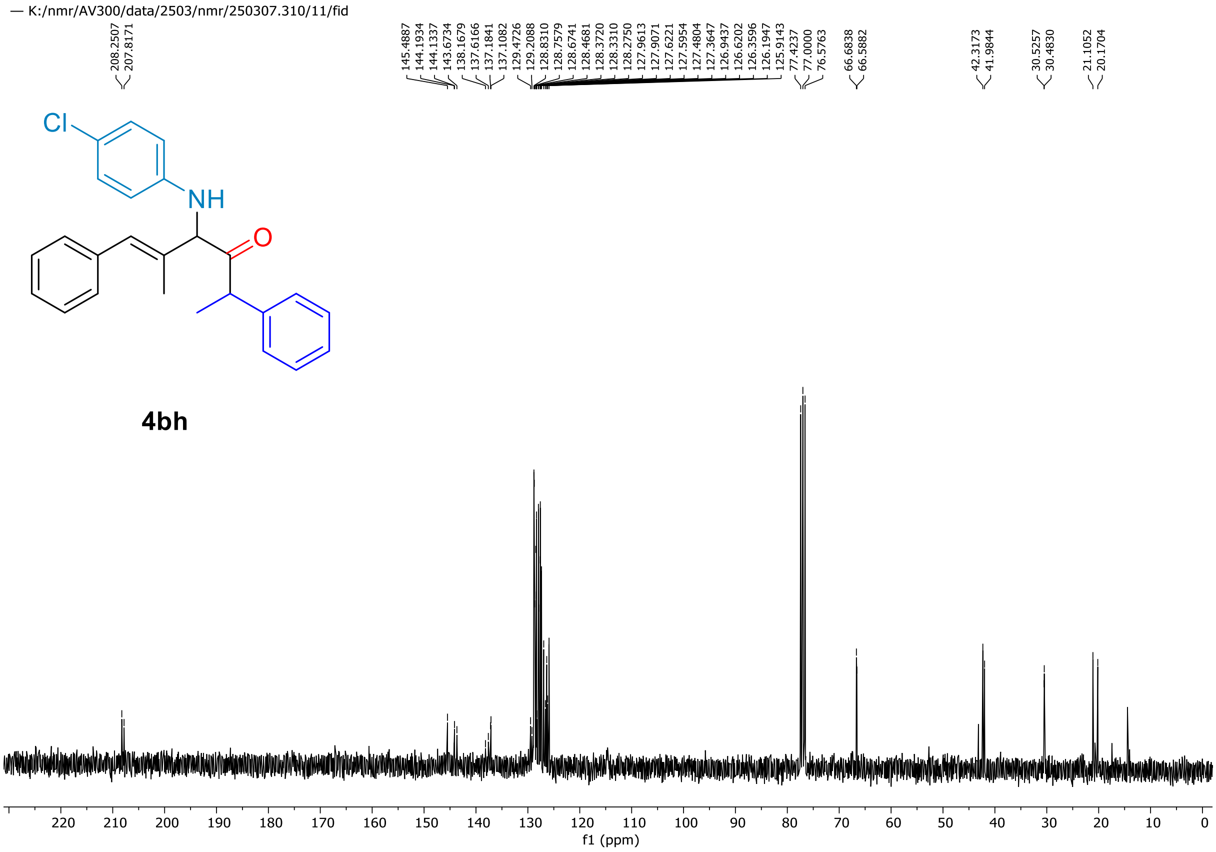

4bi ^{1}H NMR (400 MHz, $CDCl_3$)

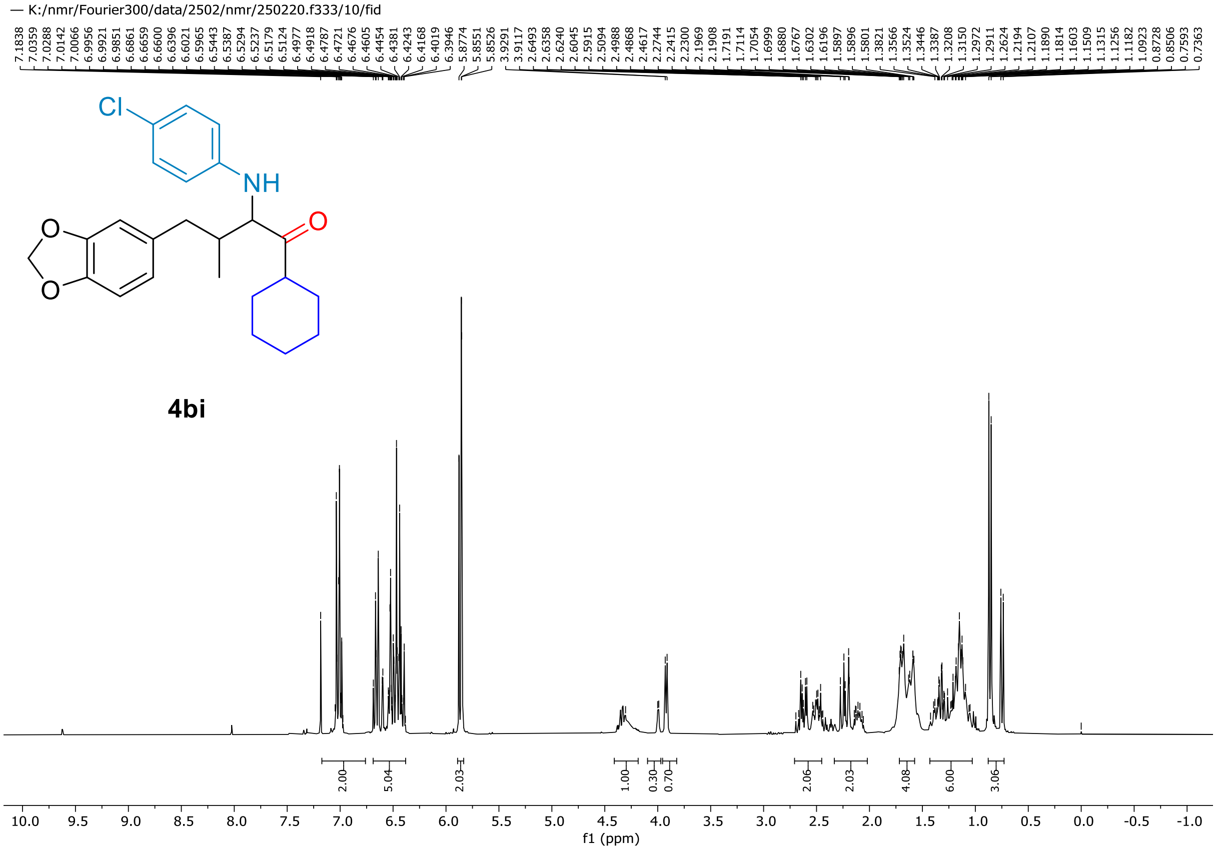

4bi ^{13}C NMR (101 MHz, $CDCl_3$)

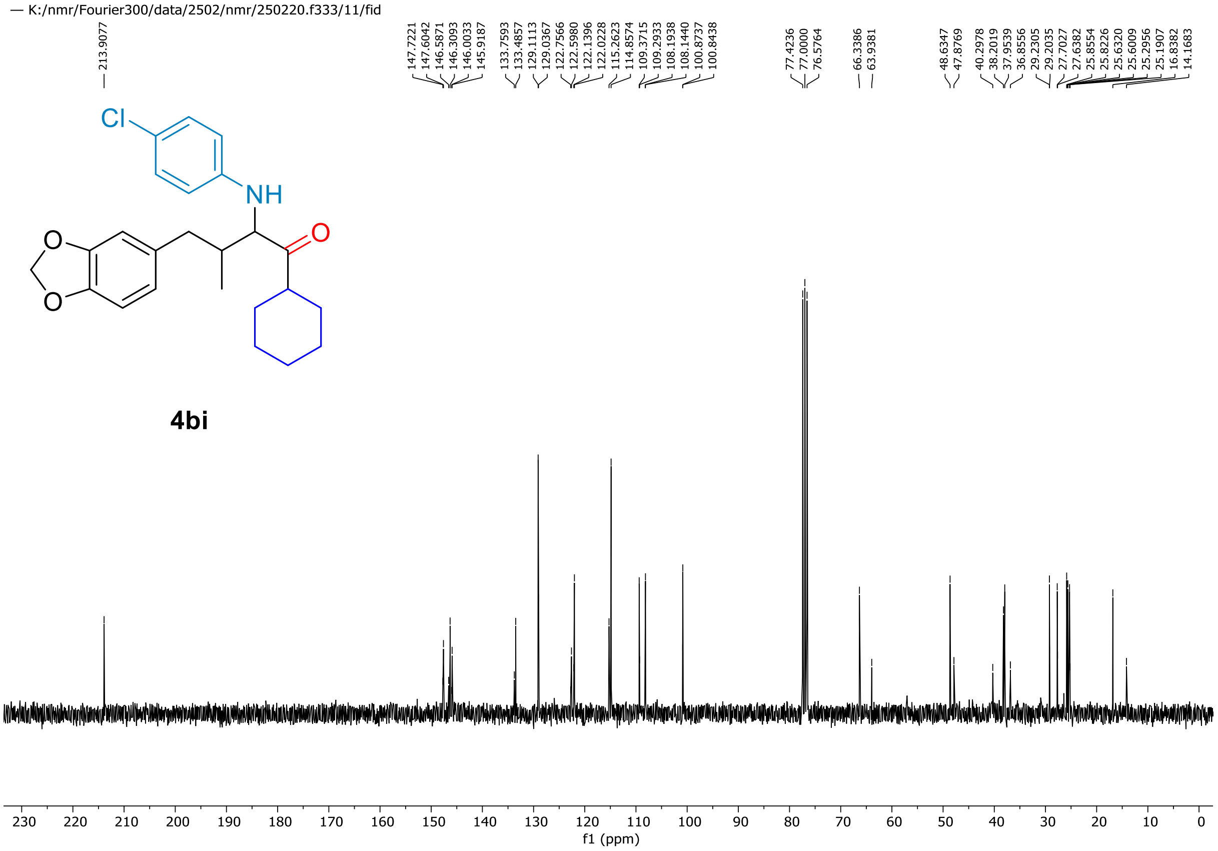

4bj ^{1}H NMR (400 MHz, $CDCl_3$)

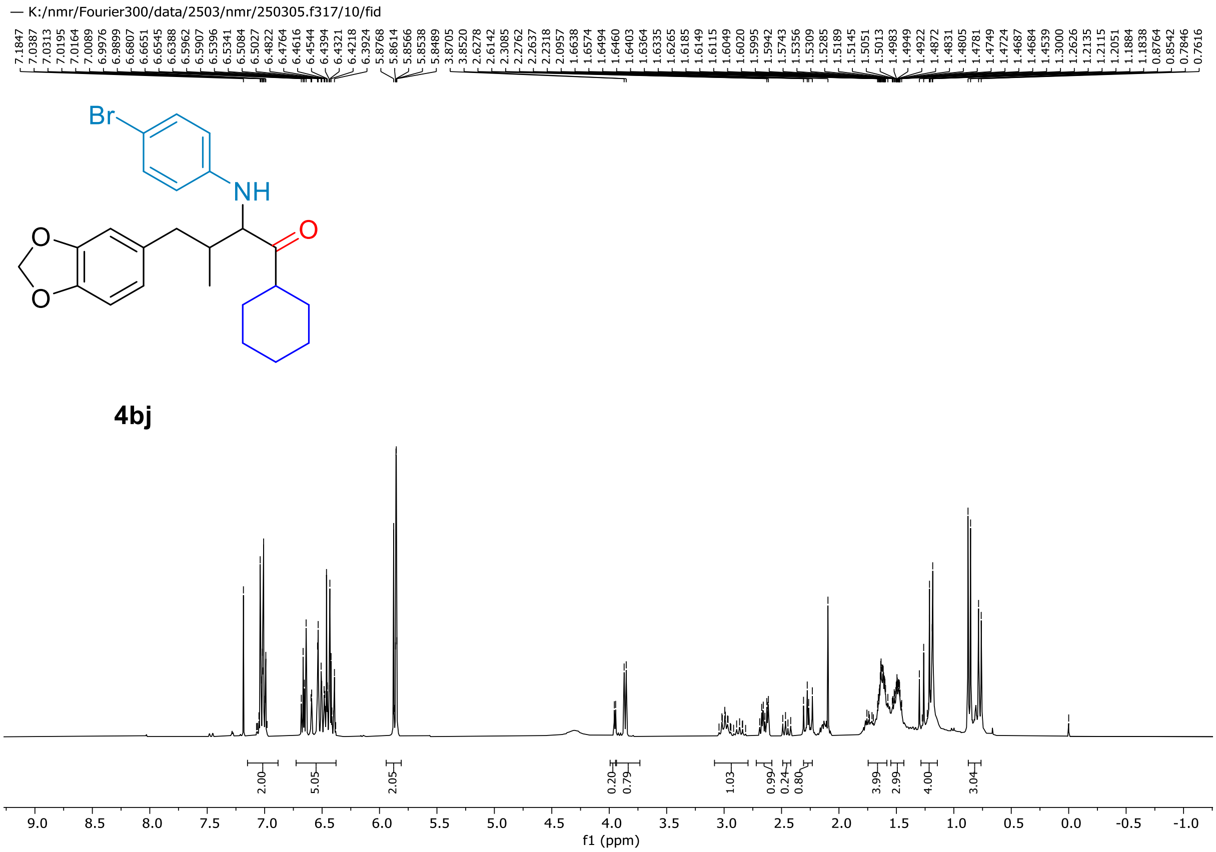

4bj ^{13}C NMR (101 MHz, $CDCl_3$)

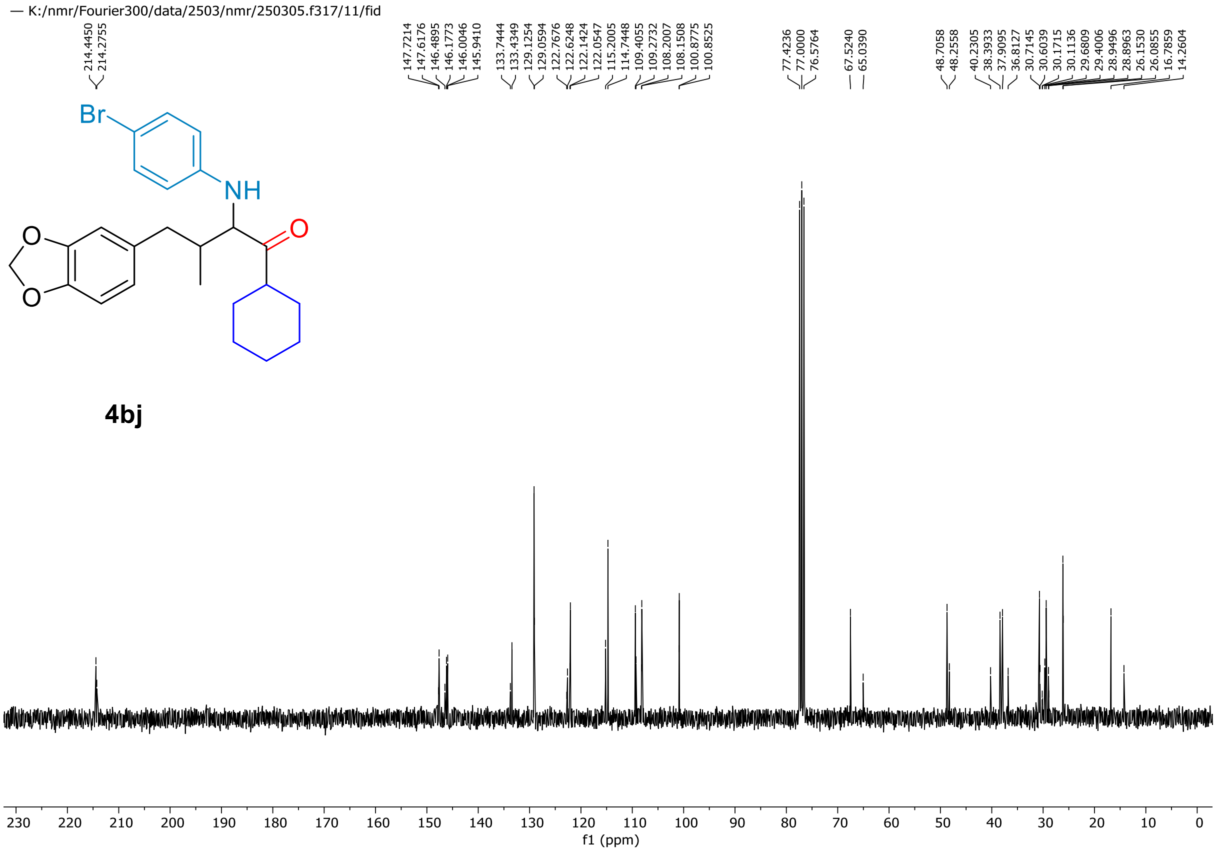

4bk ^{1}H NMR (300 MHz, CDCl$_3$)

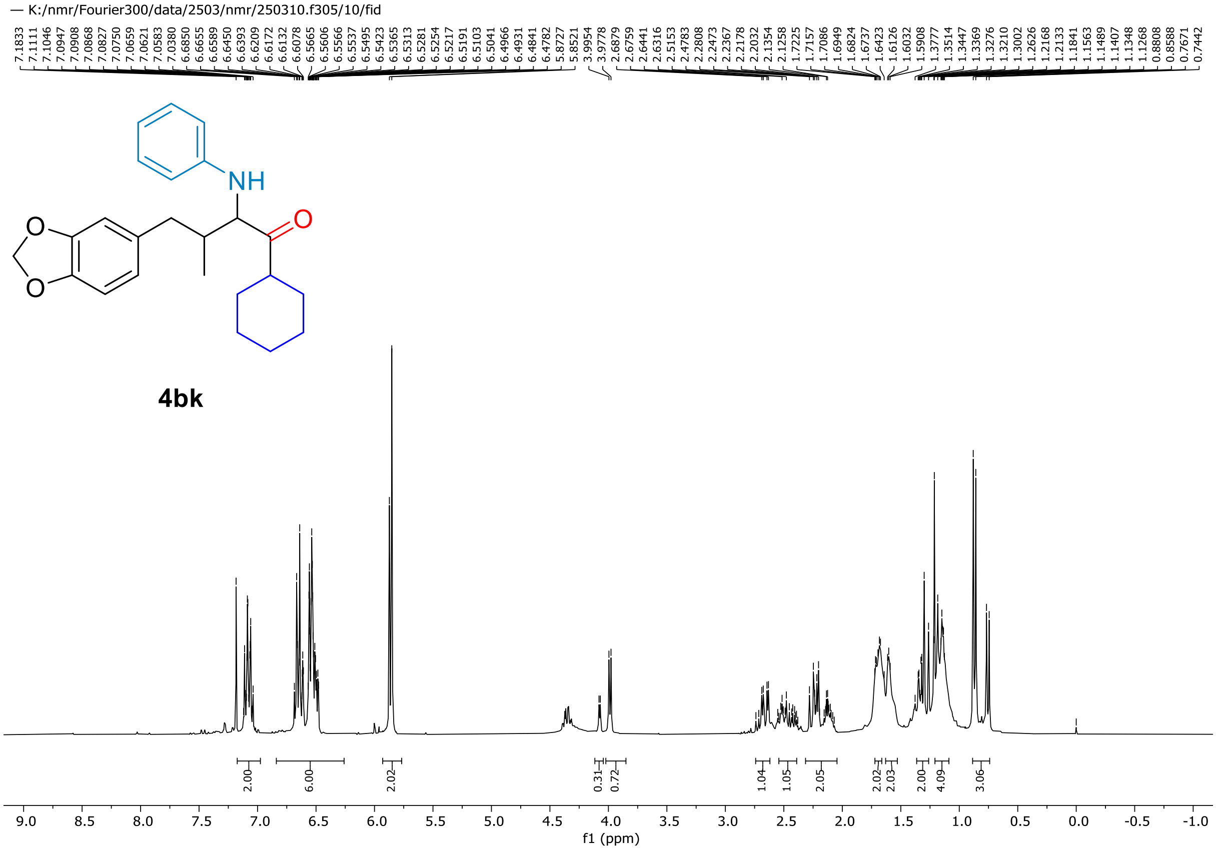

4bk ^{13}C NMR (101 MHz, CDCl$_3$)

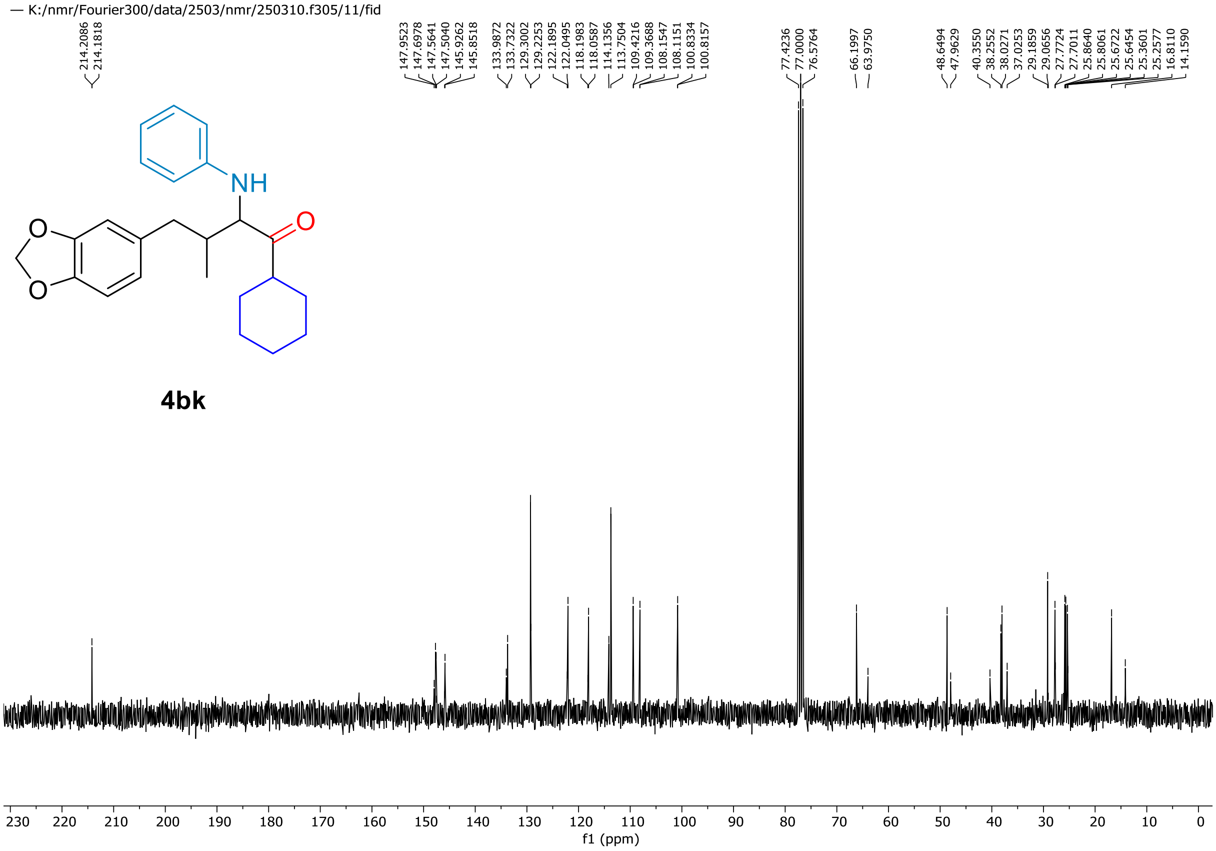

4bl ^{1}H NMR (300 MHz, CDCl$_3$)

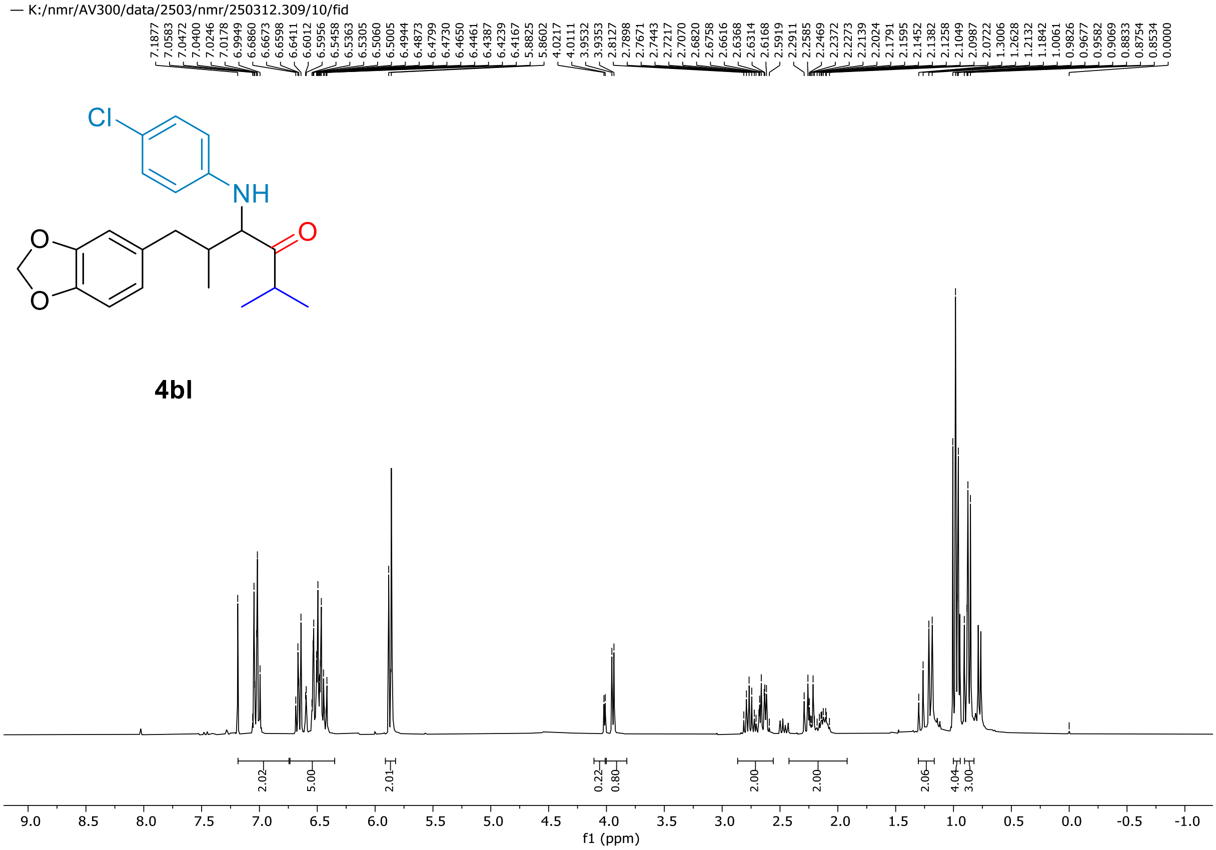

4bl ^{13}C NMR (101 MHz, CDCl$_3$)

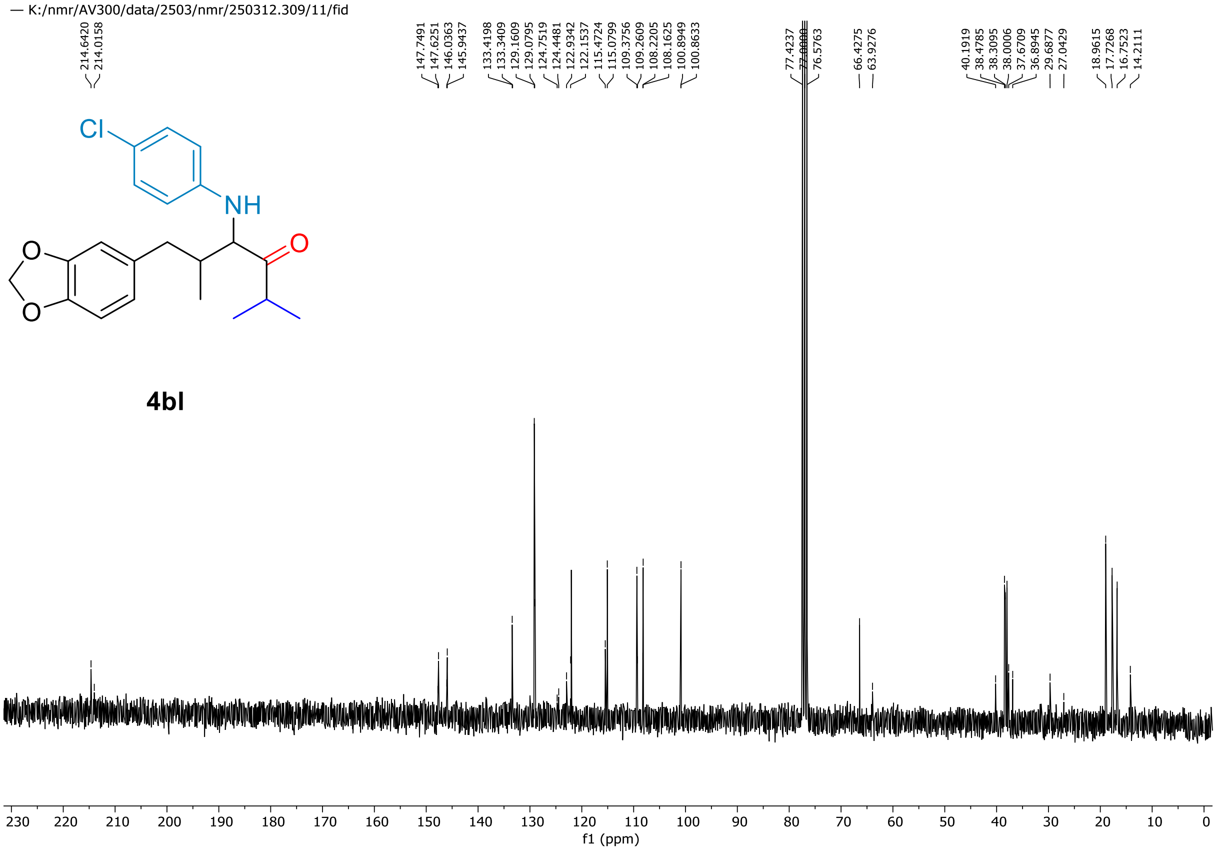

4bm ^{1}H NMR (300 MHz, $CDCl_3$)

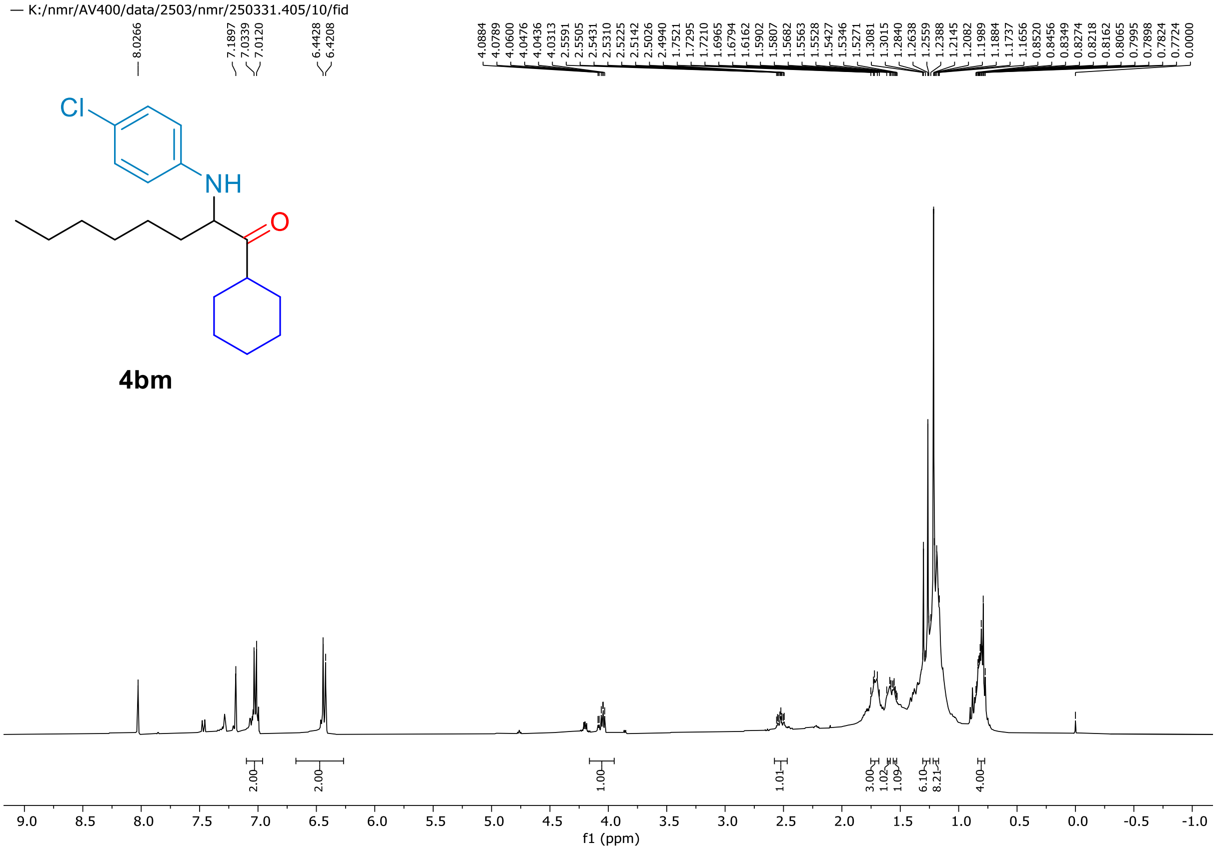

4bm ^{13}C NMR (101 MHz, $CDCl_3$)

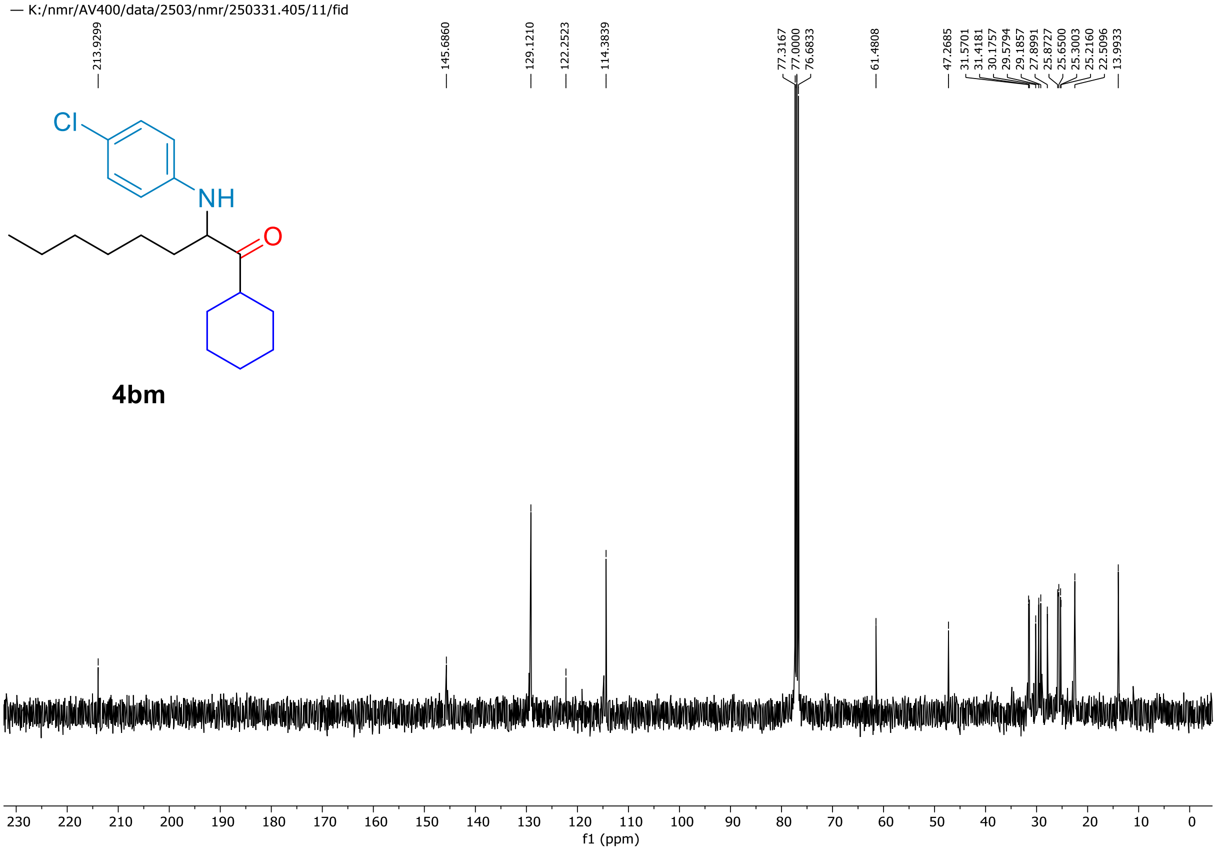

4bn ^{1}H NMR (300 MHz, $CDCl_3$)

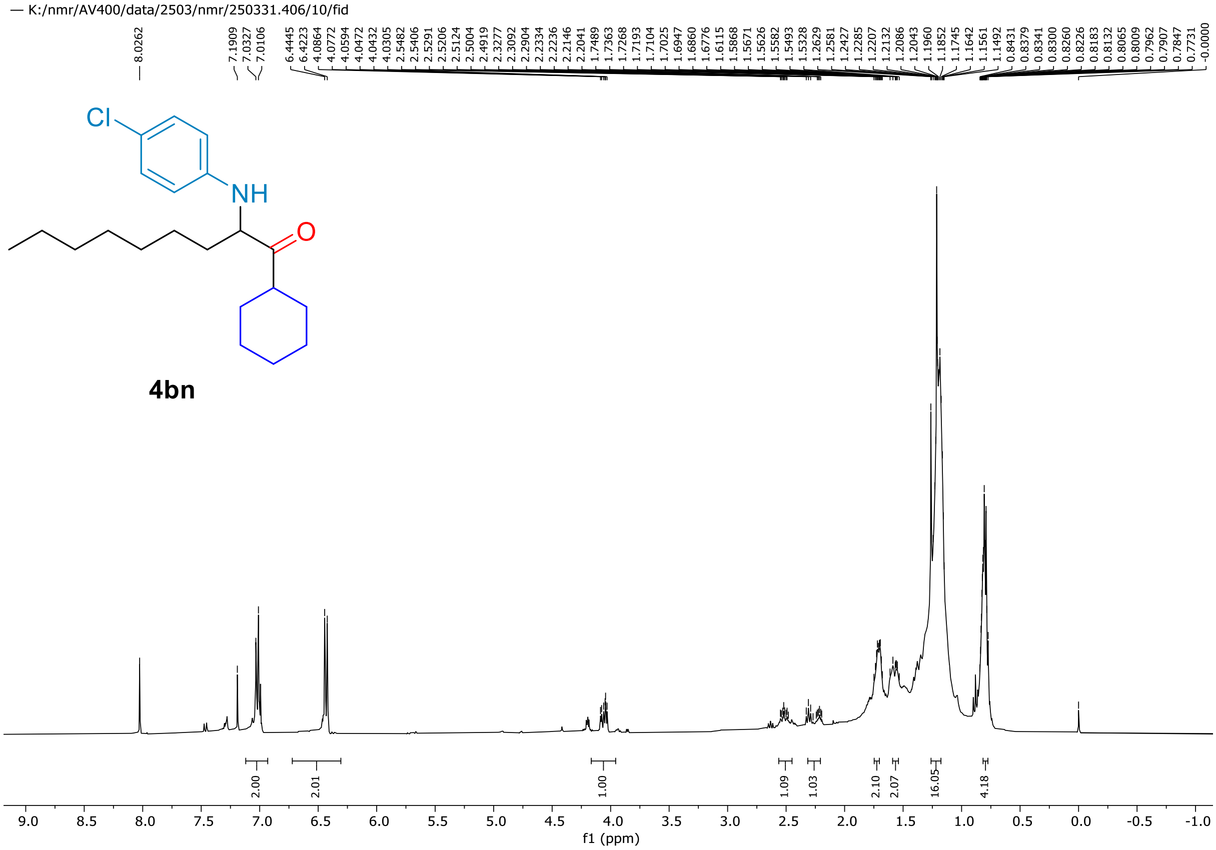

4bn ^{13}C NMR (101 MHz, $CDCl_3$)

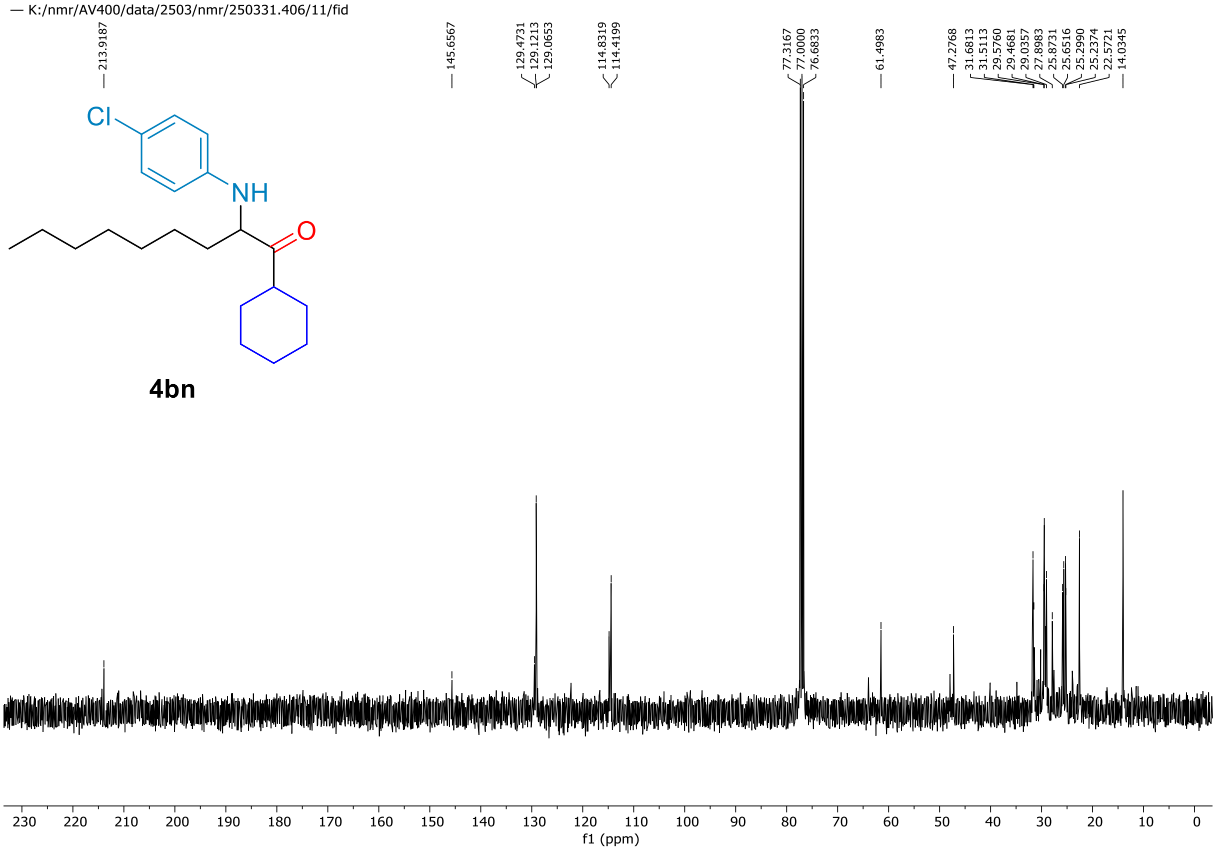

4bo ^{1}H NMR (300 MHz, $CDCl_3$)

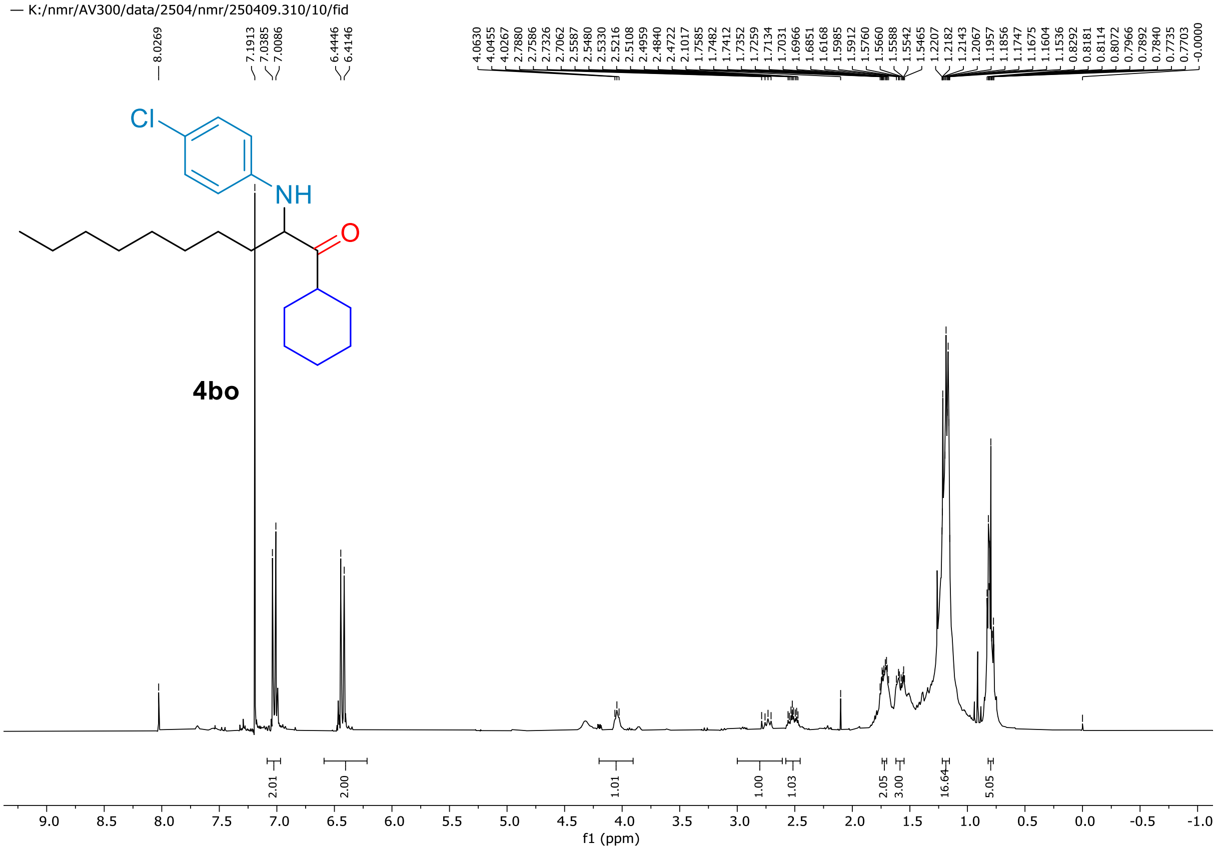

4bo ^{13}C NMR (101 MHz, $CDCl_3$)

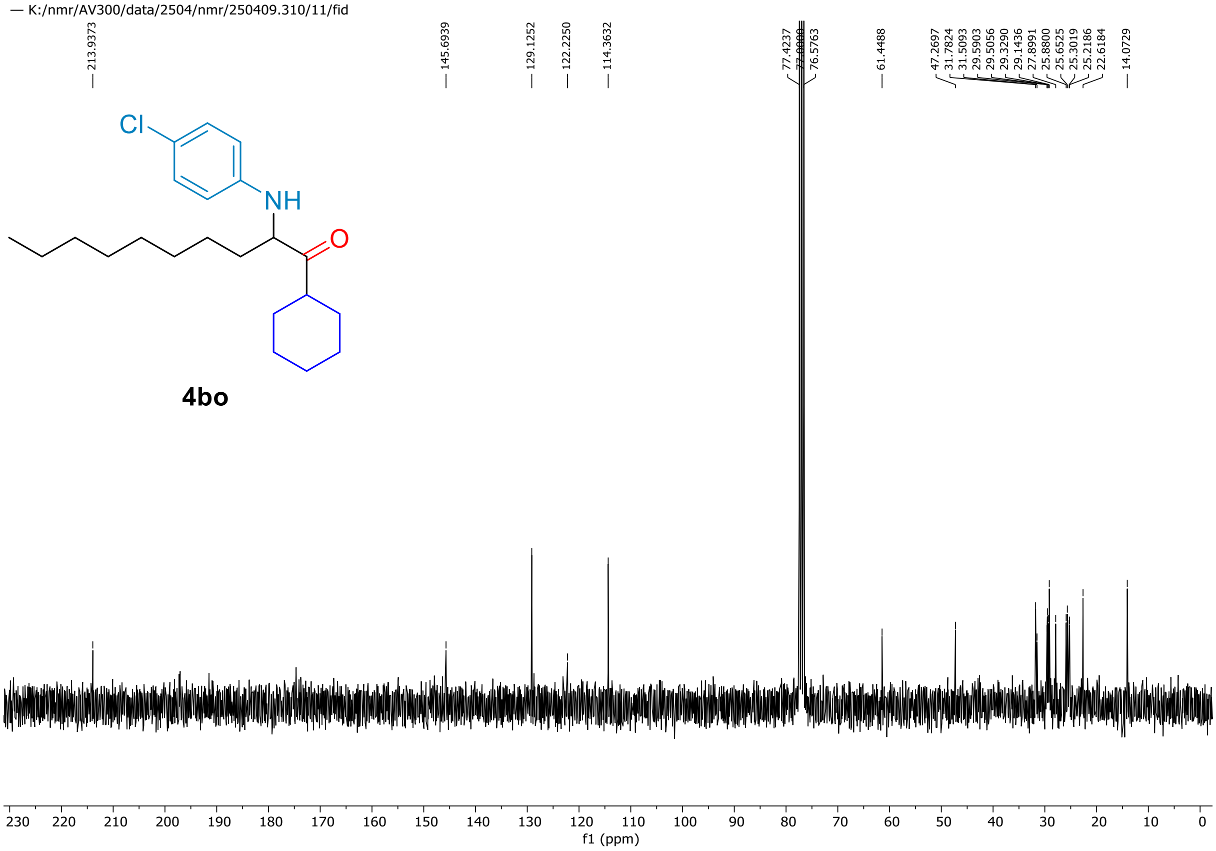

4bp ^{1}H NMR (300 MHz, $CDCl_3$)

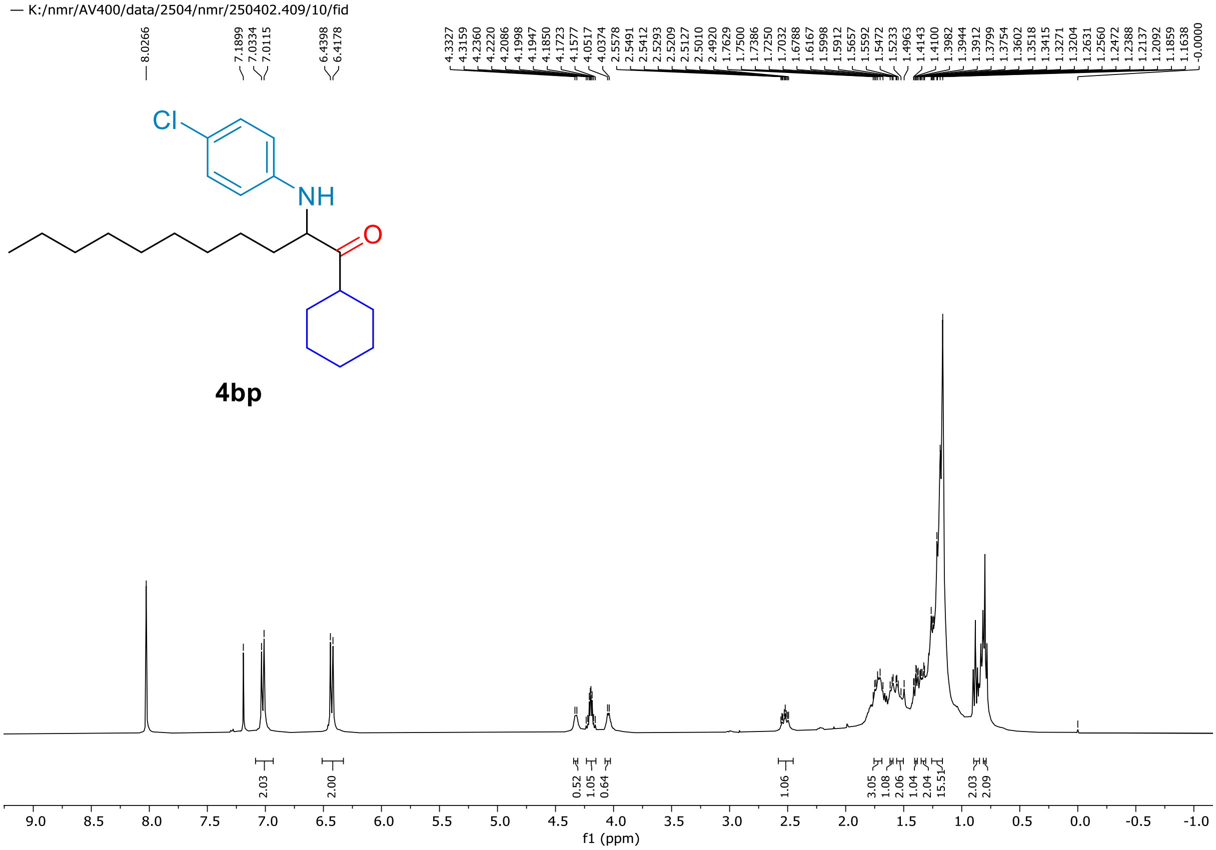

4bp ^{13}C NMR (101 MHz, $CDCl_3$)

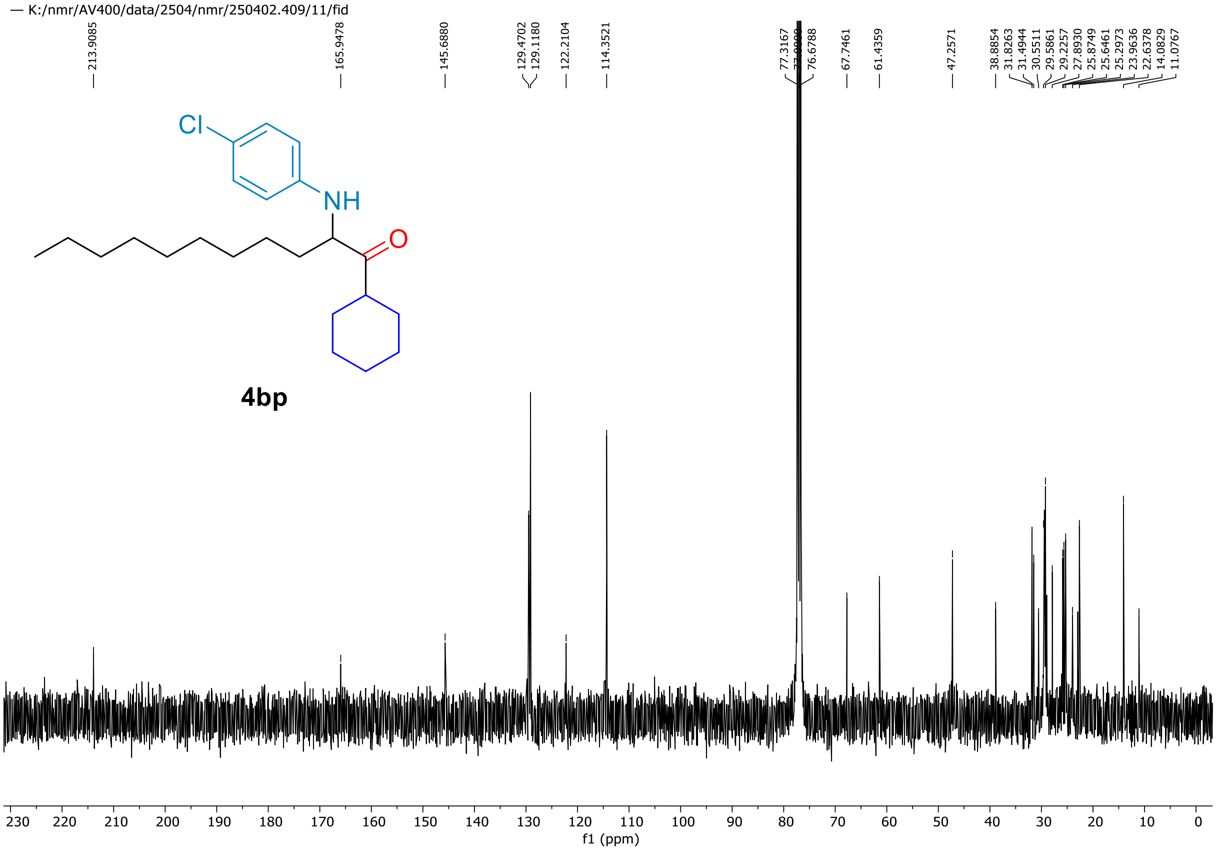

4bq ^{1}H NMR (300 MHz, $CDCl_3$)

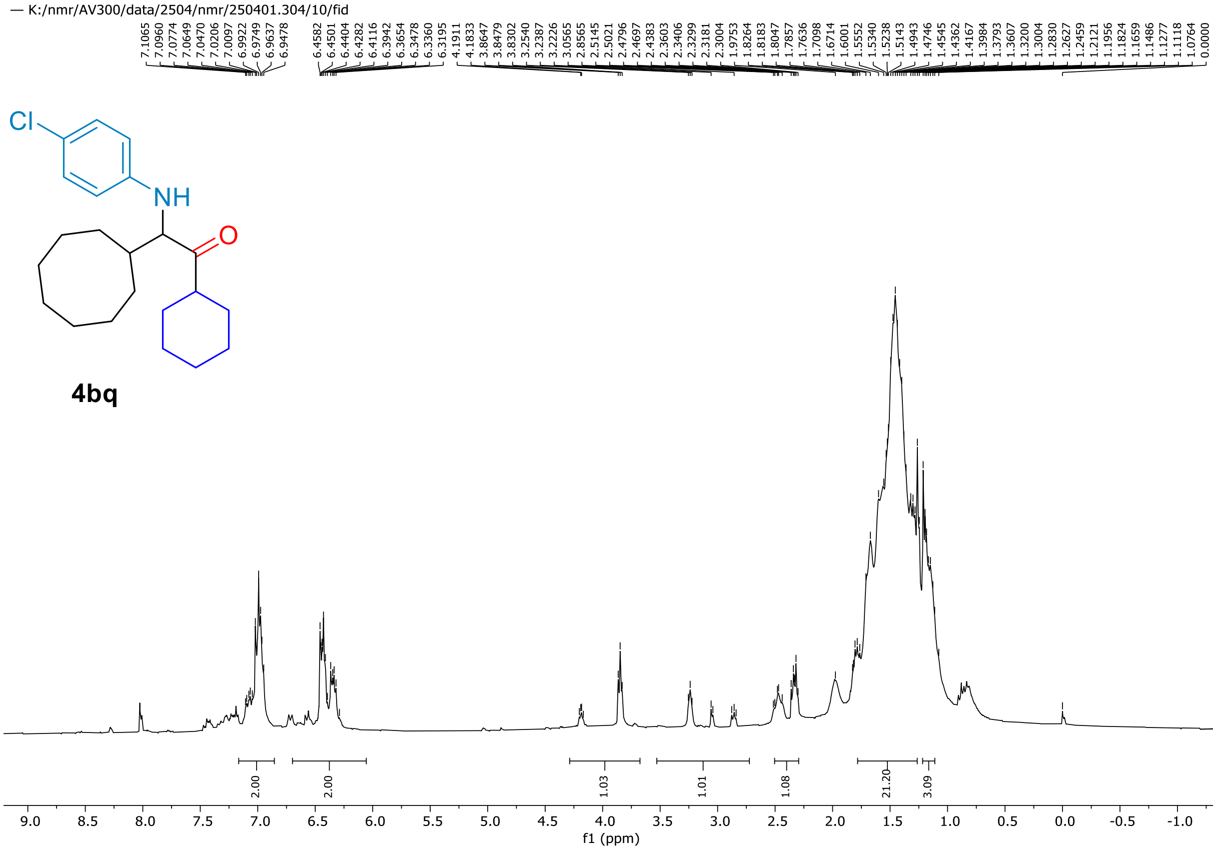

4bq ^{13}C NMR (101 MHz, $CDCl_3$)

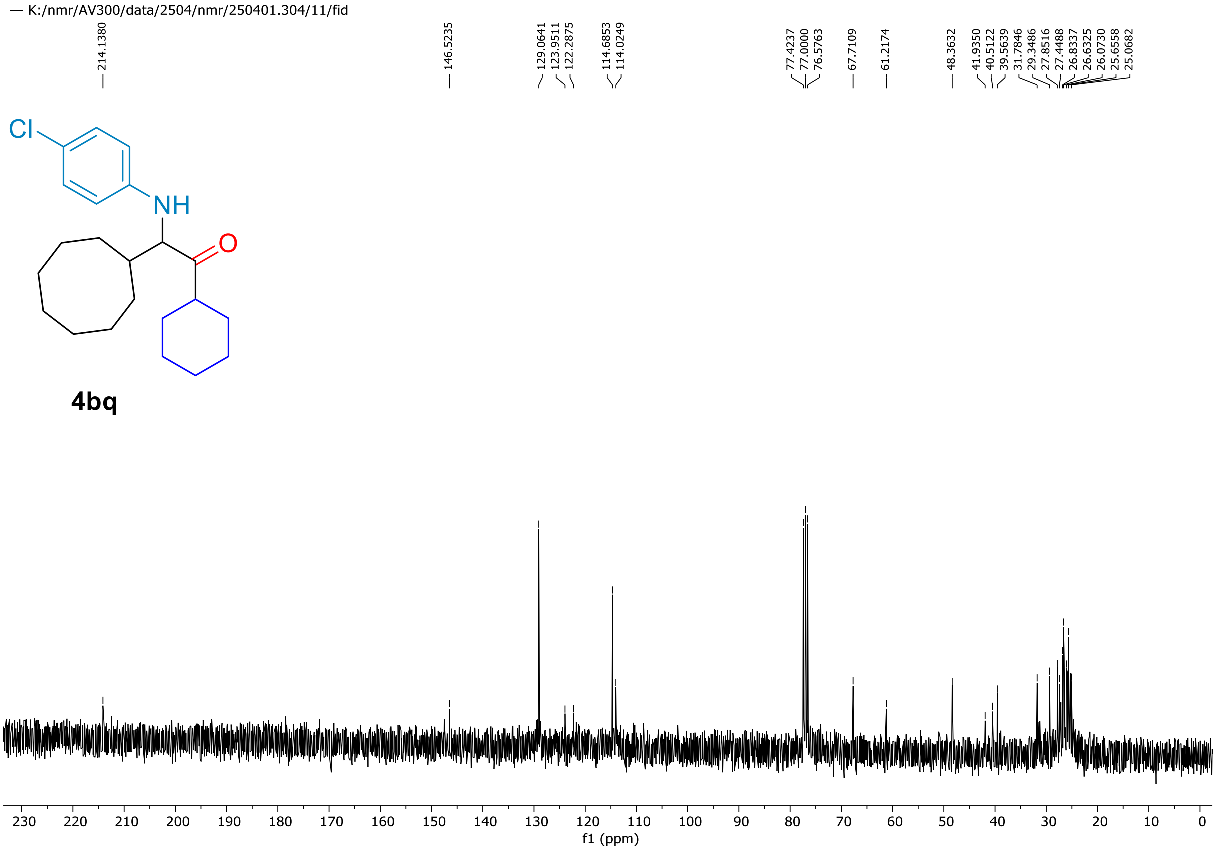

4br ^{1}H NMR (400 MHz, $CDCl_3$)

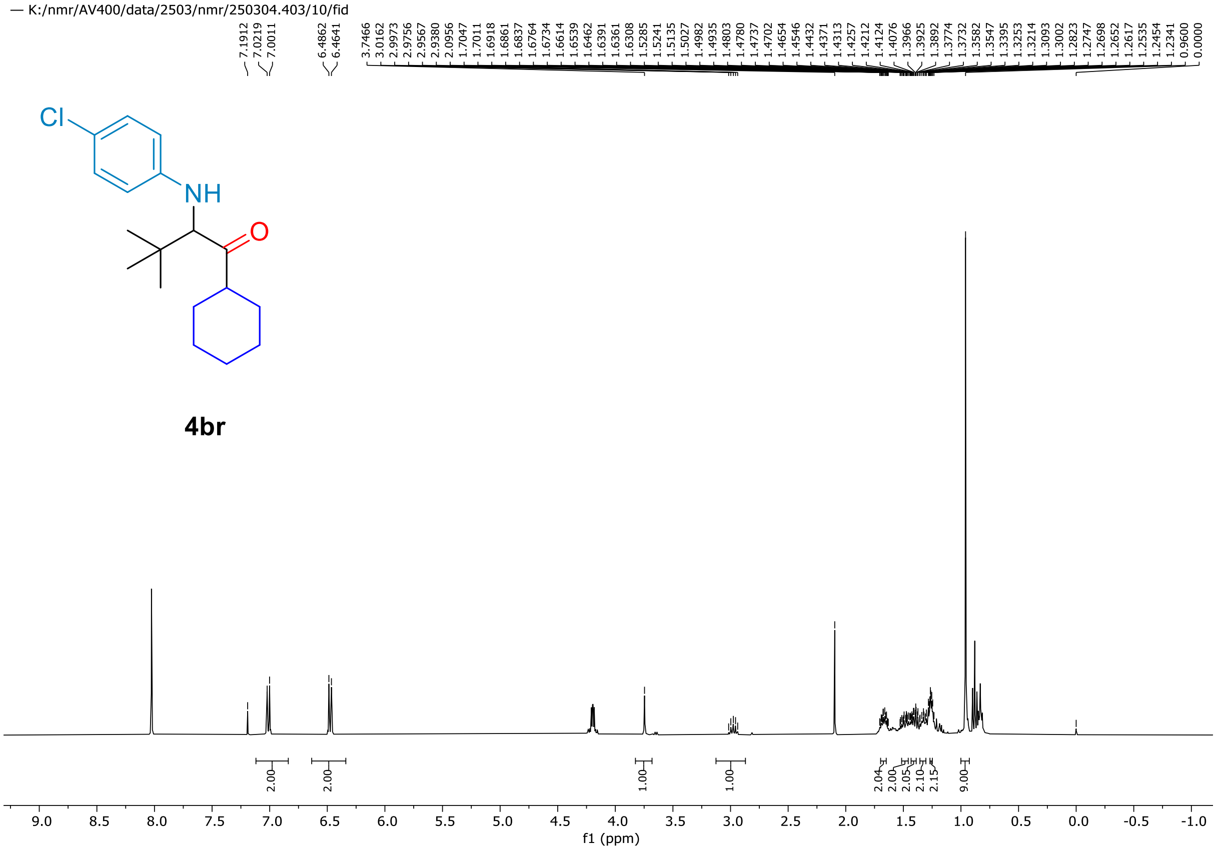

4br ^{13}C NMR (101 MHz, $CDCl_3$)

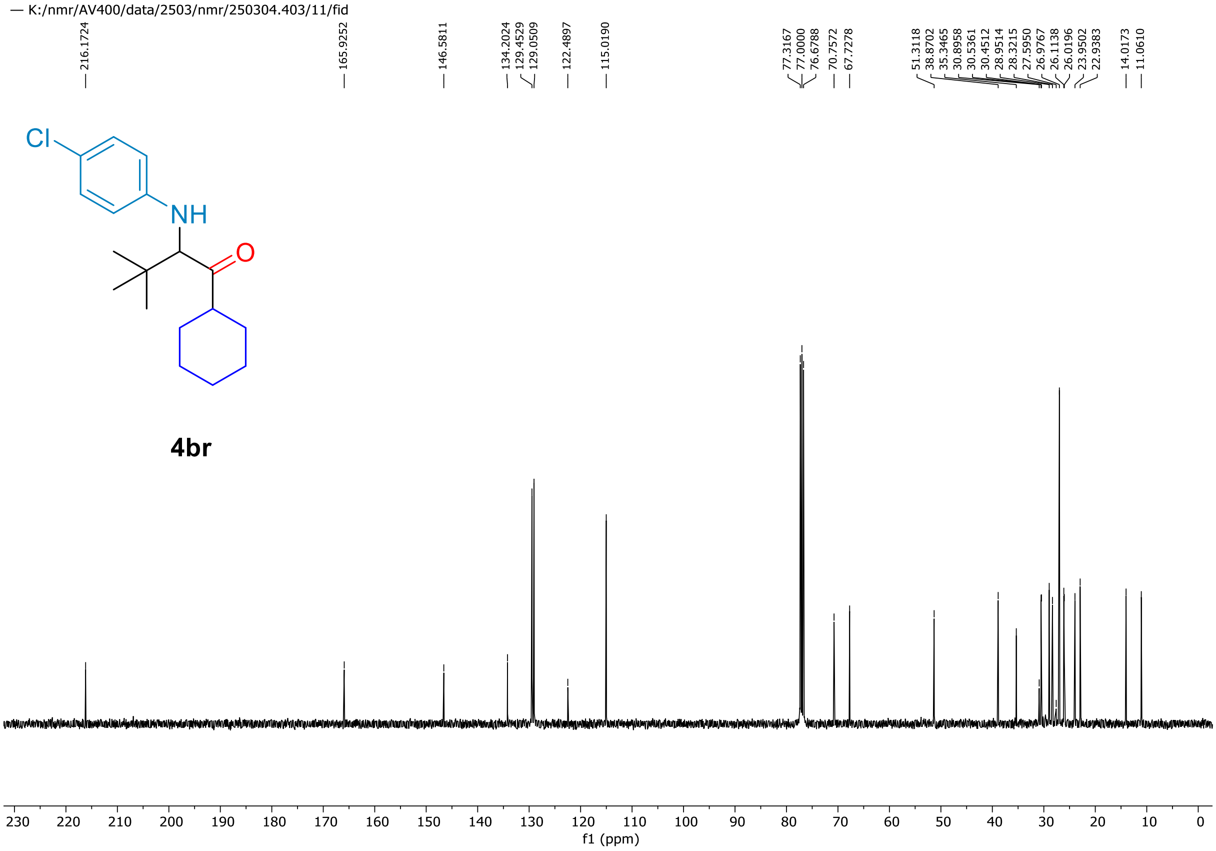

7 ^{1}H NMR (300 MHz, $CDCl_3$)

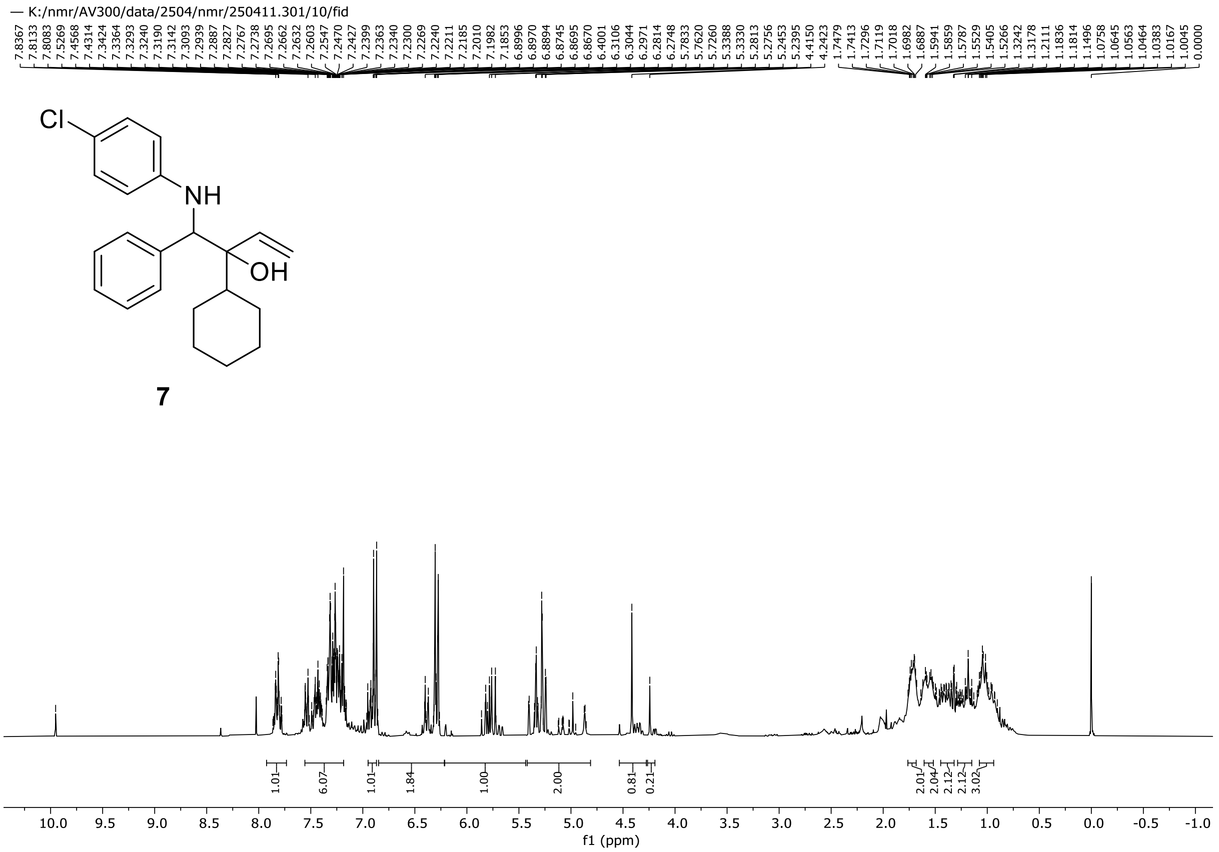

7 ^{13}C NMR (75 MHz, $CDCl_3$)

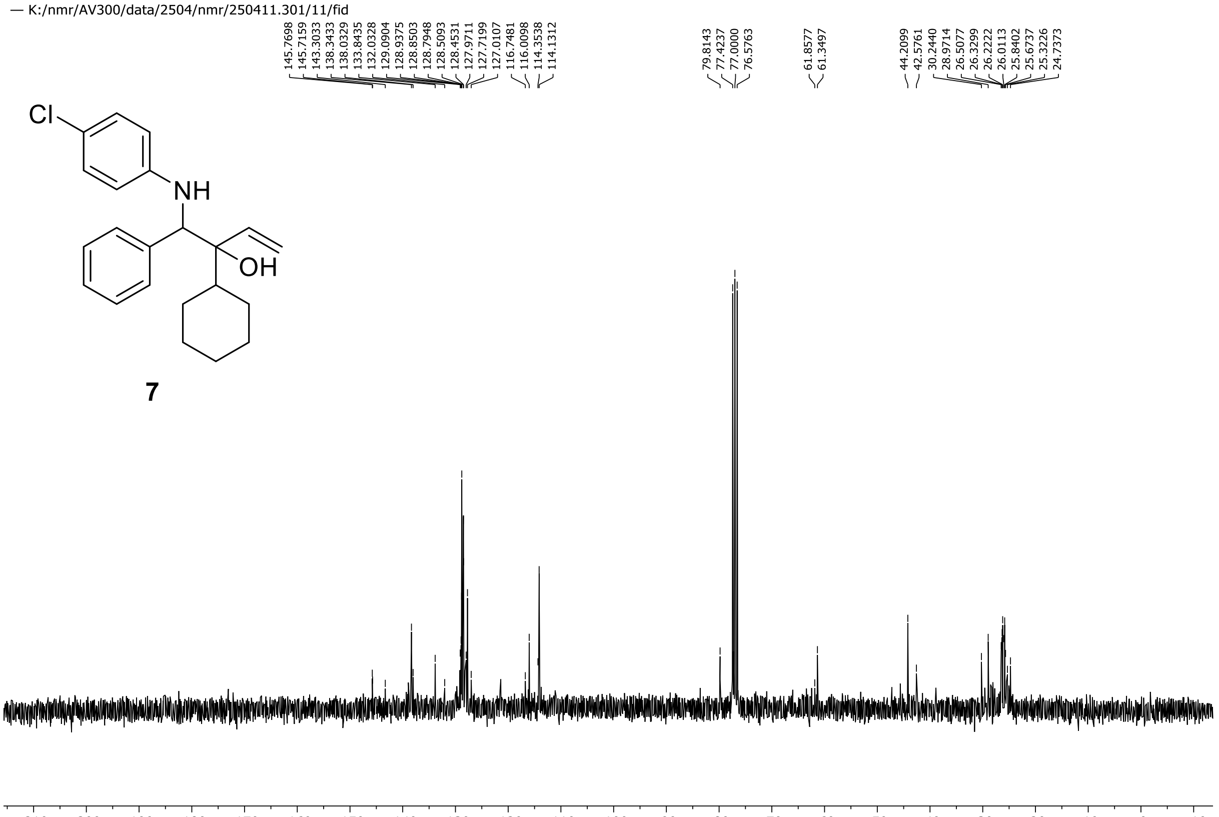

8 ^{1}H NMR (300 MHz, $CDCl_3$)

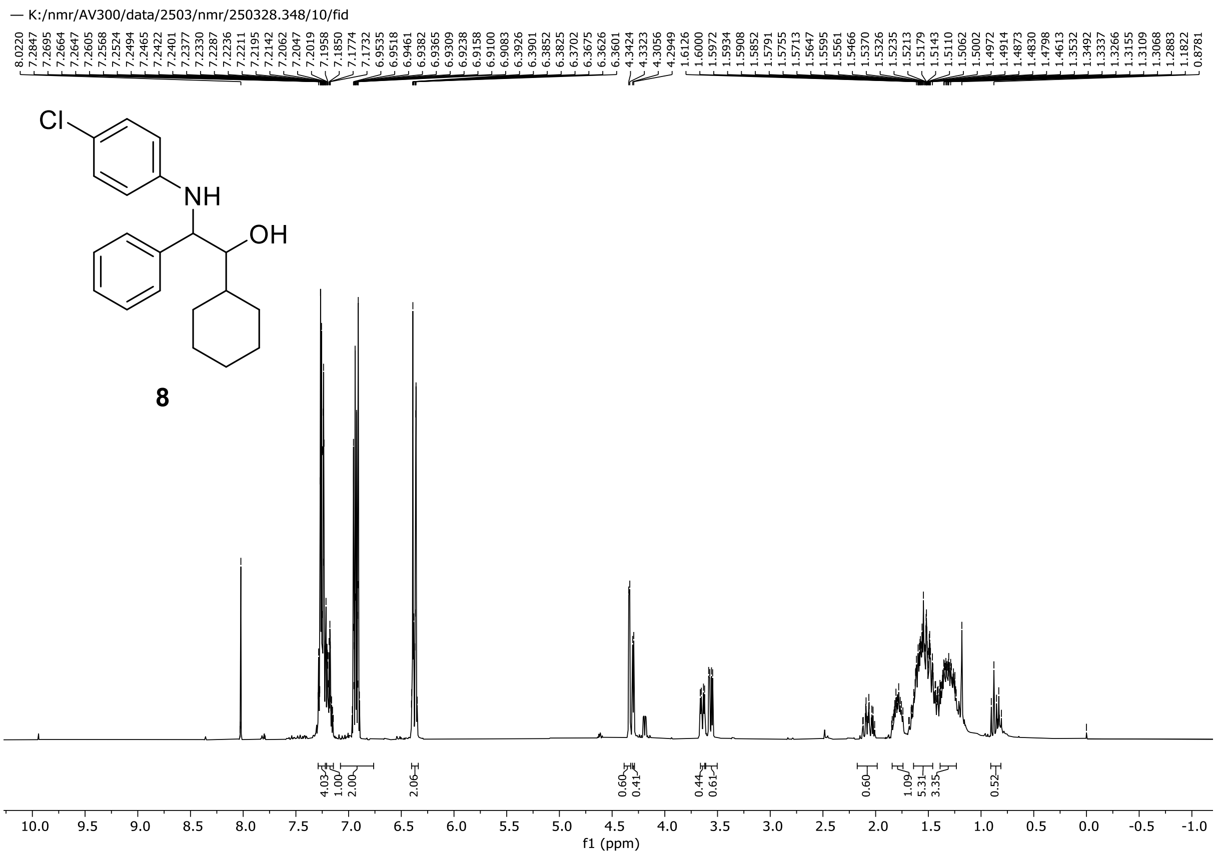

8 ^{13}C NMR (75 MHz, $CDCl_3$)

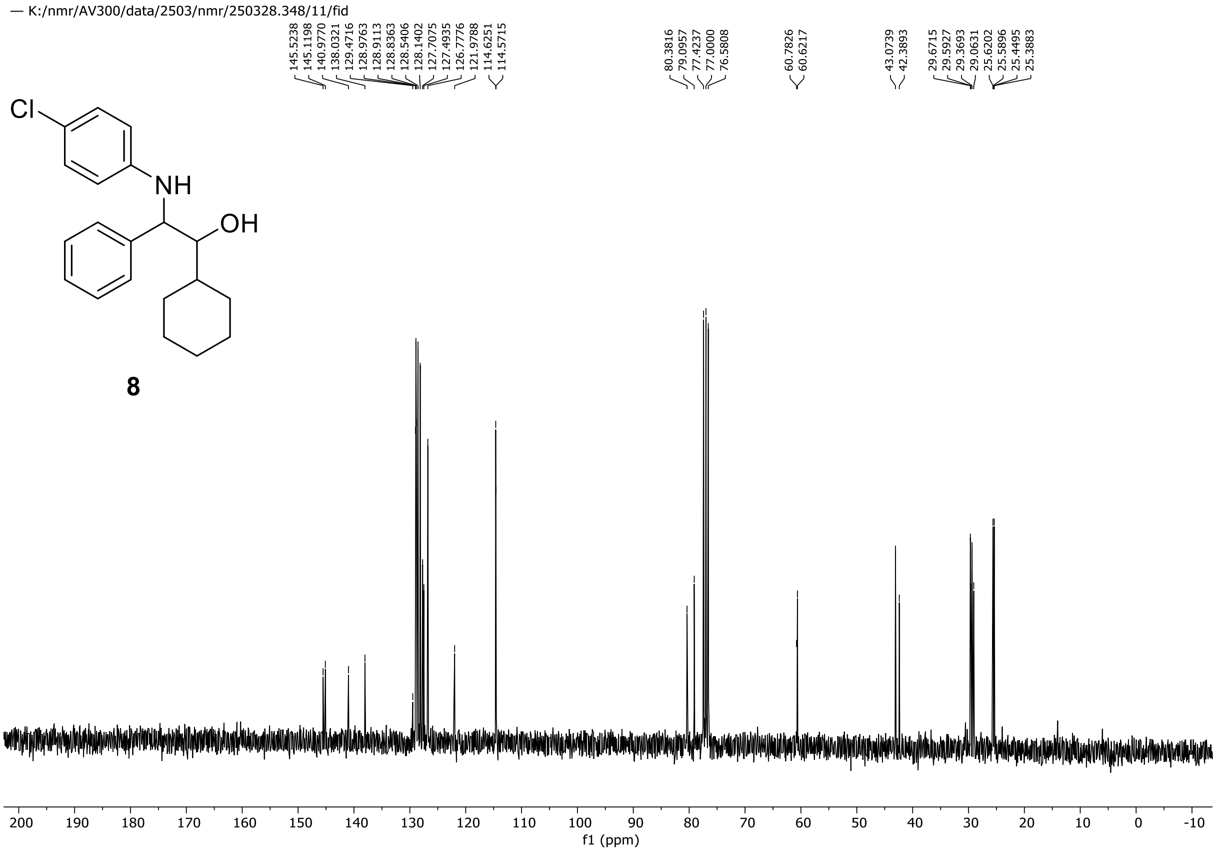

9 ^{1}H NMR (400 MHz, CDCl$_3$)

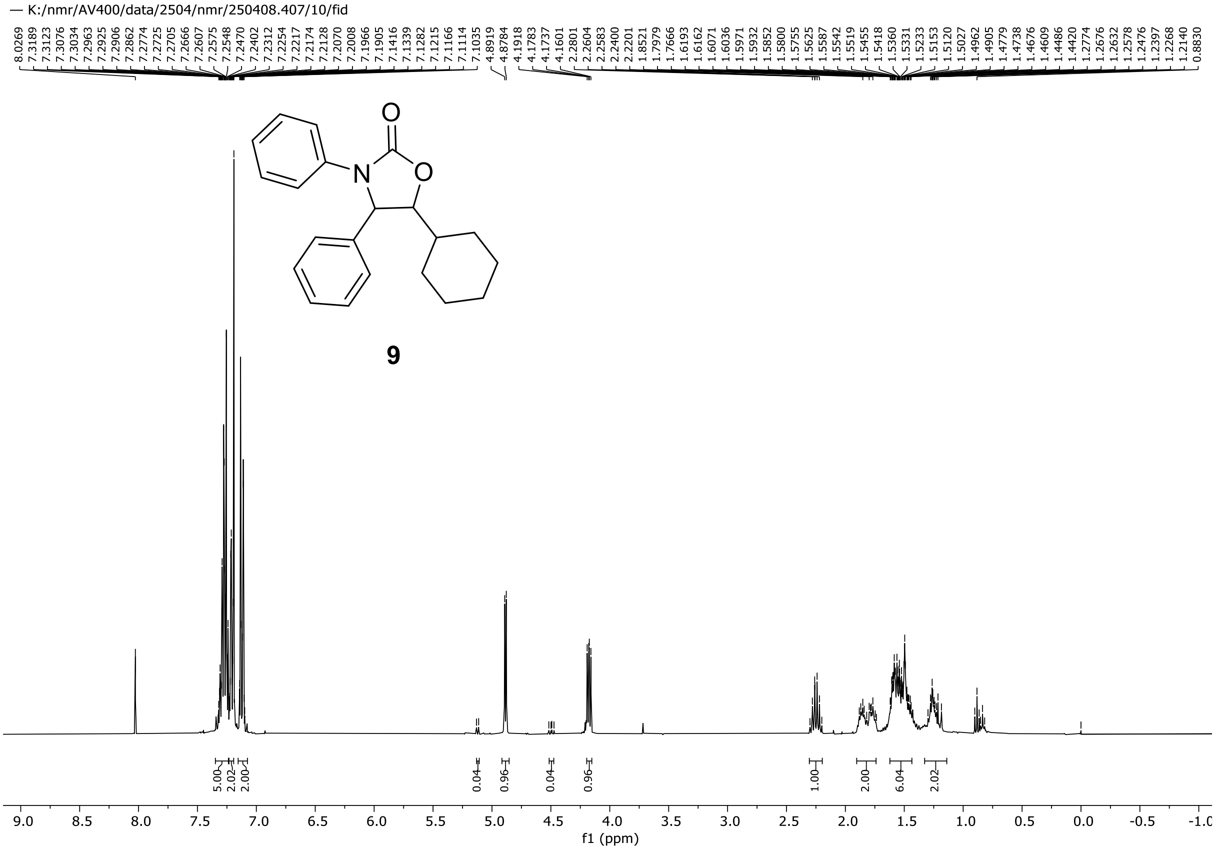

9 ^{13}C NMR (101 MHz, CDCl$_3$)

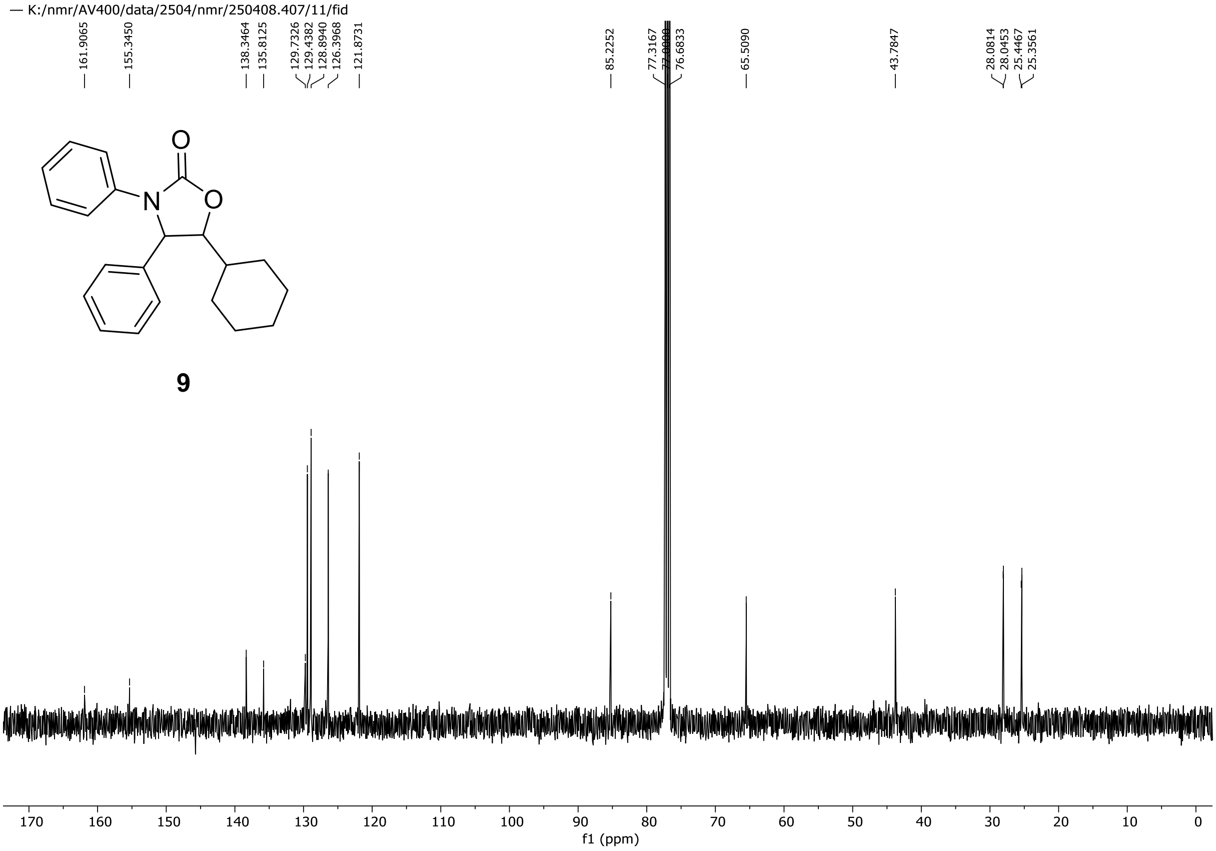

10 ^{1}H NMR (300 MHz, CDCl$_3$)

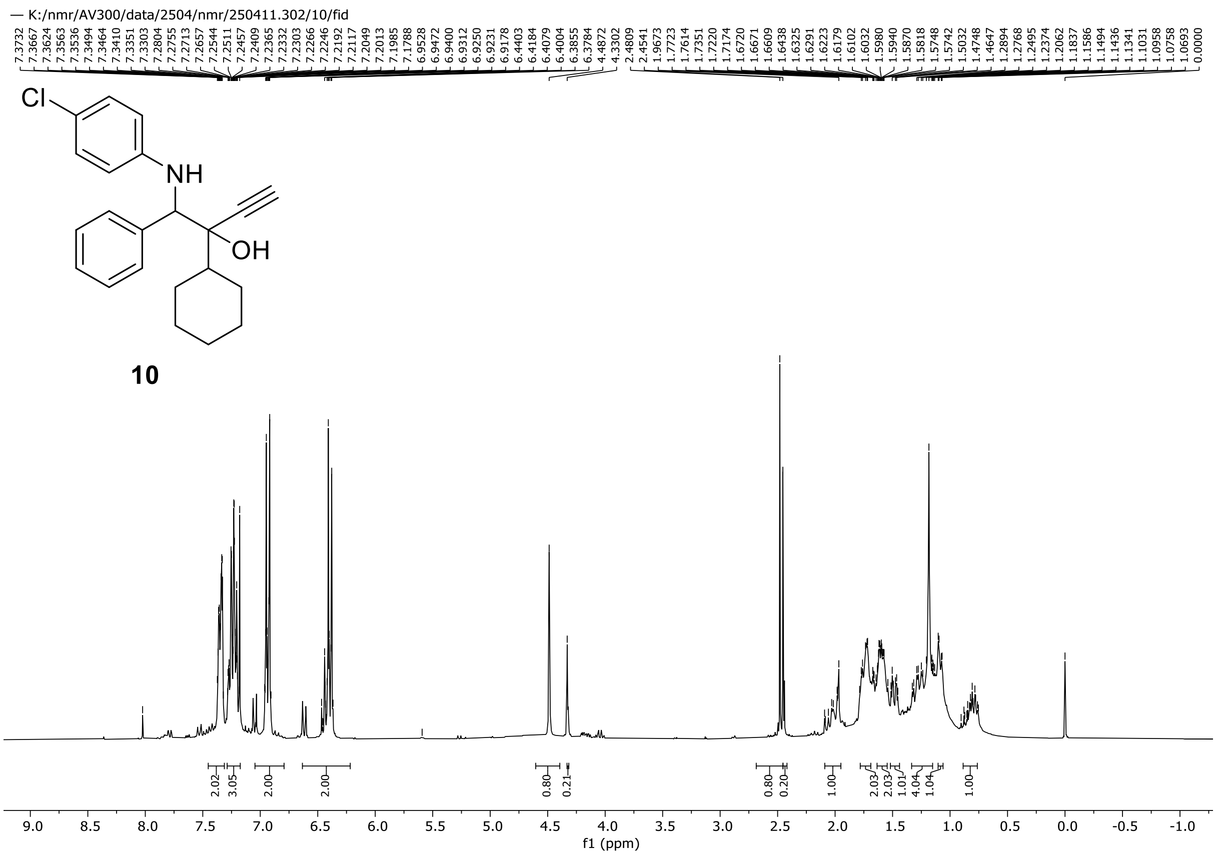

10 ^{13}C NMR (75 MHz, CDCl$_3$)

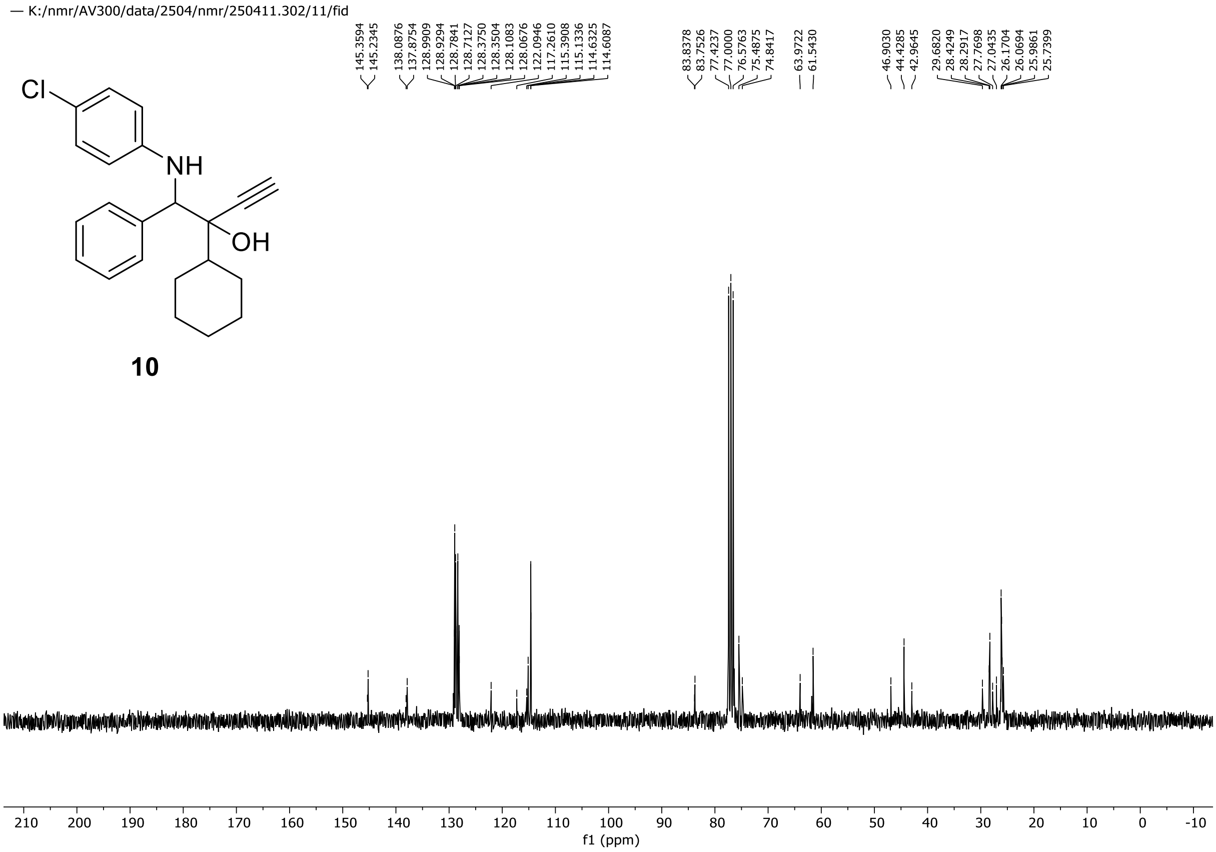

11 ^{1}H NMR (300 MHz, $CDCl_3$)

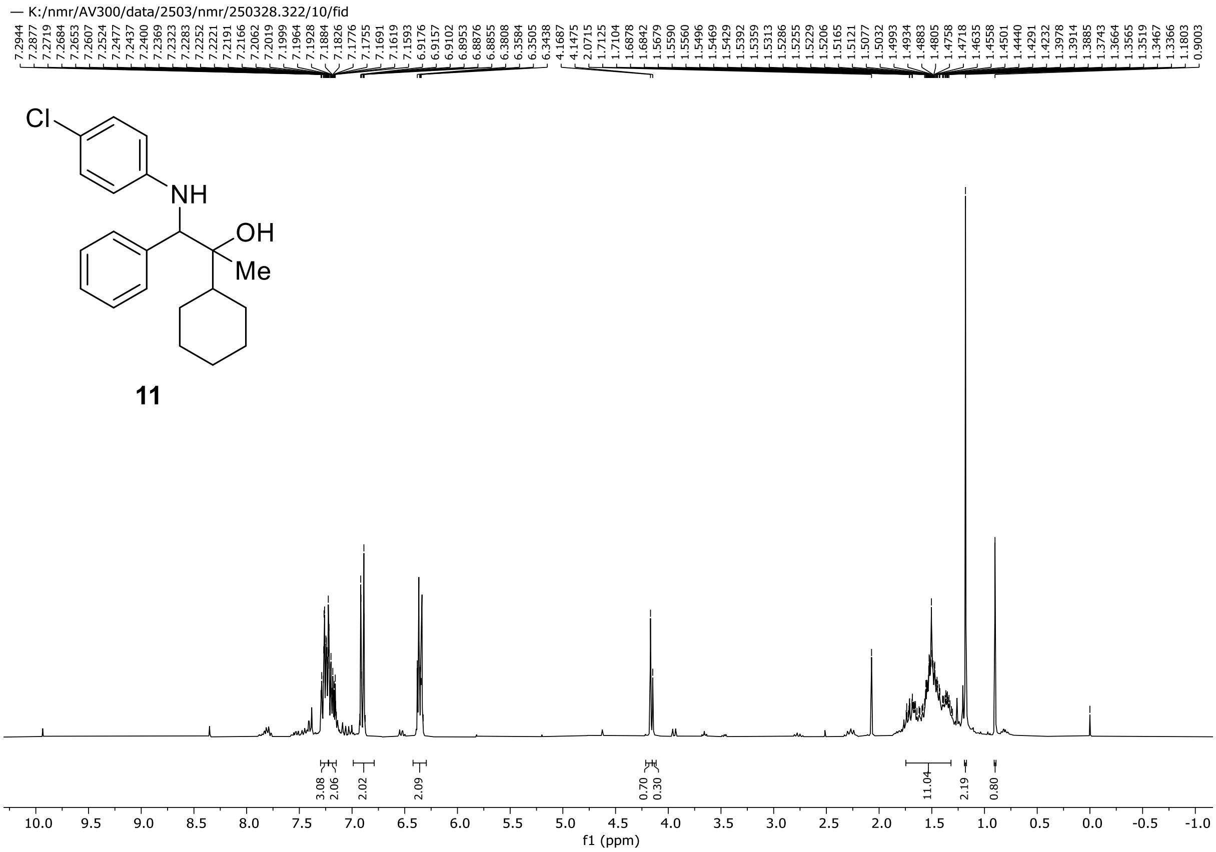

11 ^{13}C NMR (75 MHz, $CDCl_3$)

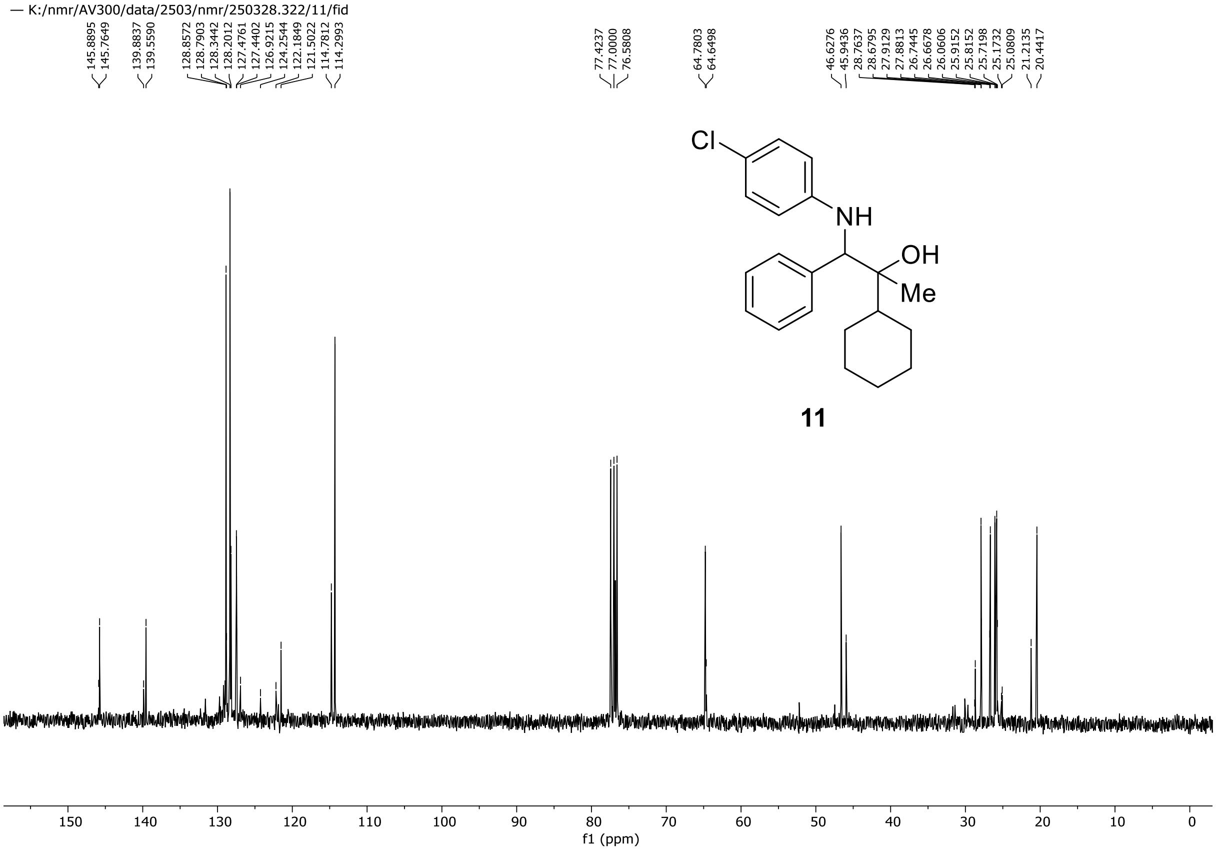

12 ^{1}H NMR (300 MHz, CDCl$_3$)

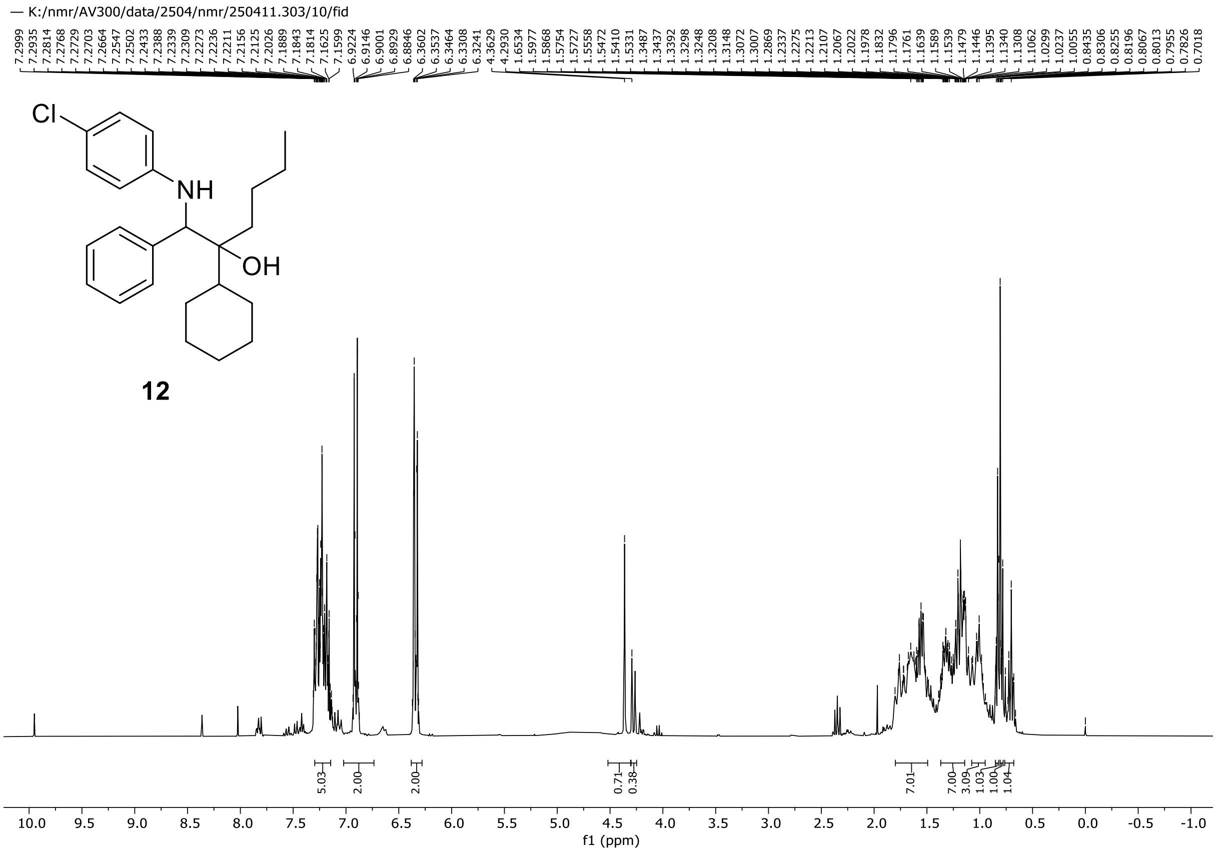

12 ^{13}C NMR (75 MHz, CDCl$_3$)

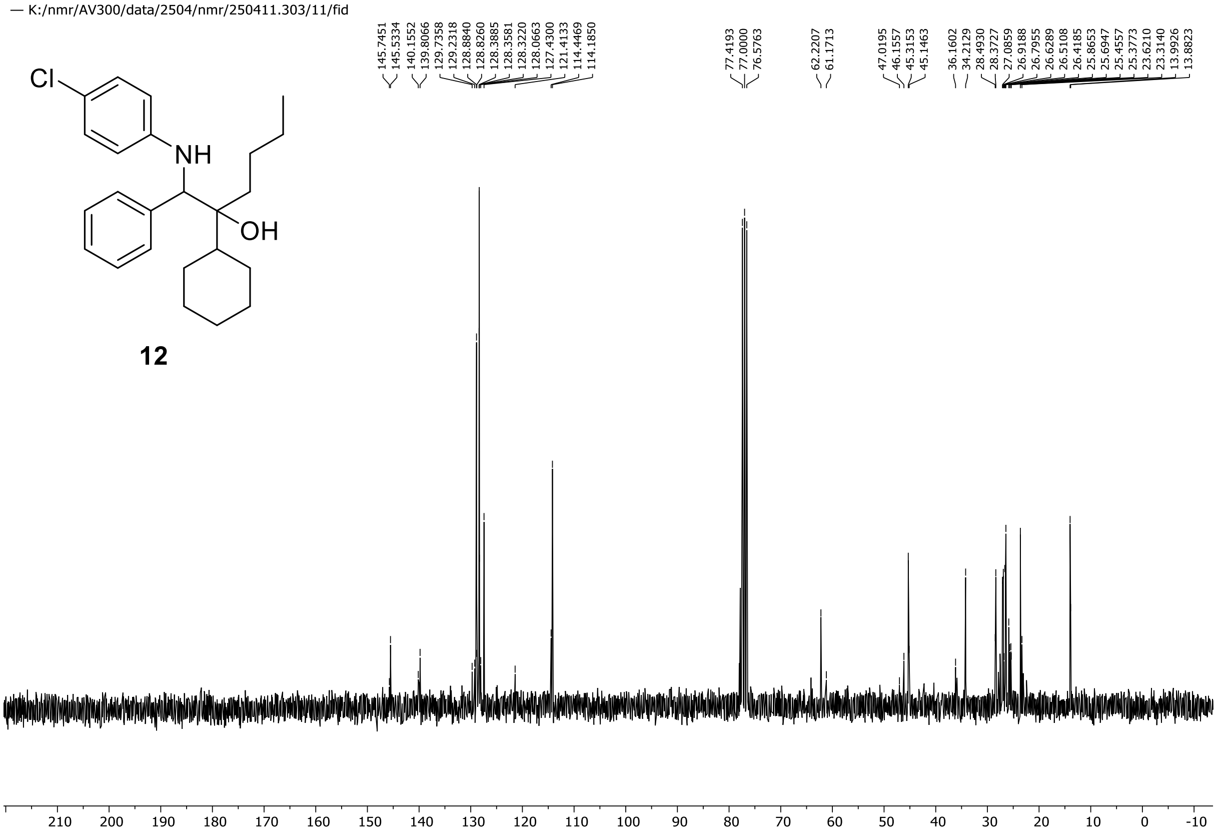

Supplement: SC-016-D5SC04120A-s001 [file SC-016-D5SC04120A-s001.pdf]
